# Evaluating childbirth options for women with obesity: a multi-criteria decision analysis

**DOI:** 10.3389/fgwh.2026.1822385

**Published:** 2026-07-15

**Authors:** Carmen Wyss, Judit Lienert, Evelyne M. Aubry

**Affiliations:** 1 Applied Research and Development, Division of Midwifery, Department of Health Professions, Bern University of Applied Sciences, Bern, Switzerland; 2 Graduate School for Health Sciences, University of Bern, Bern, Switzerland; 3 Decision Analysis Group, Department of Environmental Social Sciences, Swiss Federal Institute of Aquatic Science and Technology (Eawag), Duebendorf, Switzerland

**Keywords:** cesarean birth, childbirth care, maternal obesity, midwifery continuity of care, multi-criteria decision analysis (MCDA), quality of care, stakeholder preferences, vaginal birth

## Abstract

**Background:**

Childbirth in women with obesity (body mass index ≥30 kg/m^2^) has been linked to high medical intervention rates, adverse biomedical and psychosocial outcomes, and increased resource utilization. Person-centered decision-making on high-quality childbirth care should thus account for biopsychosocial and resource-related considerations aligned with individual preferences. This study evaluated childbirth options for women with obesity across obstetric scenarios by integrating multidimensional goals of care and stakeholder preferences with empirical evidence.

**Methods:**

We employed multi-criteria decision analysis (MCDA) using multi-attribute value theory (MAVT). The study population included women with obesity and term, singleton, cephalic pregnancies without compelling indications for cesarean birth. Options encompassed vaginal birth and intrapartum cesarean birth, each with either standard hospital care or continuous midwifery care, and prelabor cesarean birth. Stakeholder-informed goals related to biomedical safety, physiological processes, psychosocial care experience, physical strain for care providers, care setting resource use, and direct healthcare costs. Scenarios accounted for cesarean birth history and maternal comorbidities. We estimated the goal performance of the options using Swiss hospital inpatient data from 22,464 childbirths among women with obesity and assessments from experienced experts. To illustrate how stakeholder preferences may influence person-centered decision-making, we considered four hypothetical extreme and three real preference profiles. All data were aggregated into the options' overall values using a non-additive MAVT model. Sensitivity analyses addressed preference uncertainty. Direct healthcare costs were mapped onto the aggregated value of all other goals in cost-benefit visualizations.

**Results:**

Vaginal birth with standard hospital care or continuous midwifery care achieved similar or higher overall values compared to cesarean birth across a wide range of preference profiles. Prelabor and intrapartum cesarean birth generally achieved lower or comparable overall values, with greater variability depending on stakeholder preferences. Cost-benefit visualizations showed that vaginal birth provided good value at the lowest costs. Findings were largely consistent across obstetric scenarios.

**Conclusion:**

This study highlights the importance of integrating multidimensional goals and stakeholder preferences into decision-making on childbirth options, alongside empirical data. In doing so, vaginal birth with either model of care performed well across the considered scenarios and preference profiles, though the findings should not be interpreted as prescriptive. This MCDA approach offers a novel framework to support evidence-based and person-centered decision-making deliberations on high-quality childbirth care for women with obesity.

## Plain English summary

Women with larger bodies (body mass index of 30 or higher) are more likely to experience medical interventions and complications during childbirth. However, obesity alone is not a medical reason to plan a cesarean birth. Decisions about how to give birth should consider not only medical safety, but also individual goals and preferences related to natural birth processes, the experience of care, the use of healthcare resources, and costs.

In this study, we compared different childbirth options for women with obesity who had no clear medical need for a cesarean birth by taking into account what matters to women and their care providers. The options included vaginal birth and cesarean birth after labor had started, each with either standard hospital care or continuous care from a known midwife, and planned cesarean birth before labor. We used a structured decision-making approach that combined empirical data with individual goals and preferences. Specifically, we analyzed data from nearly 22,500 births in Switzerland together with information about what is most important to women and care providers in childbirth care.

Overall, vaginal birth—whether with standard hospital care or continuous care from a known midwife—performed as well as or better than cesarean birth for most preferences and clinical situations considered in our study. Vaginal birth also generally involved lower healthcare costs. These findings suggest that vaginal birth could often be a good option for women with obesity unless there is a clear medical reason for a cesarean birth. The approach used in this study may help support informed, person-centered discussions about childbirth care between women and their care providers.

## 1 Introduction

Every pregnant woman has the right to high-quality childbirth care that aligns with her goals, preferences, and the best available scientific evidence (1). However, providing such care for women with obesity [body mass index (BMI) ≥30 kg/m^2^] may present unique challenges, including higher rates of medical interventions, obstetric complications, adverse biomedical and psychosocial outcomes, and increased resource utilization (2–6). While current guidelines primarily adopt a clinical perspective aimed at improving outcomes for women with obesity and their children (7–9), decision-making in this context is normatively complex (10) and may not be adequately informed by single clinical outcomes alone. Instead, person-centered, high-quality childbirth care encompasses biomedical, physiological, psychosocial, and resource-related considerations (1, 10, 11), requiring explicit attention to multidimensional goals and individual preferences for trade-offs between them.

At the same time, women with obesity are frequently classified as “high-risk”, a designation that may influence decisions on childbirth care (12). Even though obesity itself is not a medical indication for operative delivery (7, 8), they experience cesarean births at disproportionately high rates (2–4), which may pose additional risks (13) and technical challenges (14) in this population. Notably, biomedical obstetric factors have been found to explain only part of these increased rates, accounting for 18%–57% of prelabor and intrapartum cesarean births in women with obesity (15). This suggests that decision-making on cesarean birth may also be shaped by factors beyond clinical indications, such as biopsychosocial priorities and structural considerations (16, 17). In addition, women with obesity may not be considered eligible for midwifery continuity models of care due to their obstetric risk status (18). Such models have been associated with beneficial biomedical, physiological, and psychosocial childbirth outcomes, along with a trend towards saving resources (19). Nevertheless, evidence on continuous midwifery care for women at higher-risk of complications, including those with obesity, remains limited (19).

These complexities highlight the need for approaches that explicitly consider multidimensional goals of childbirth care, stakeholder preferences, and empirical evidence to support high-quality decision-making on childbirth modes and models of care for women with obesity. To address this critical need, we employed multi-criteria decision analysis (MCDA). MCDA refers to a methodological approach that enables the integration of multiple goals, preferences for achieving them, and empirical data into structured evaluation models (20–23). By making trade-offs between competing goals explicit and allowing for the incorporation of both scientific and preferential uncertainty (20, 24), MCDA has been proposed as a promising approach to inform evidence-based, person-centered decision-making in healthcare [cf. (25)]. Yet, to our knowledge, such integrative frameworks remain scarce in the maternity care context. Therefore, the objective of this study was to explore the application of MCDA to assess the performance of childbirth options for women with obesity across several obstetric scenarios. A sub-objective was to examine how the performance of these options may vary in response to stakeholder preferences. In doing so, this study aimed to contribute a novel framework to facilitate evidence-based and person-centered deliberations on childbirth care for women with obesity.

## 2 Methods

### 2.1 MCDA using multi-attribute value theory (MAVT)

We used multi-attribute value theory (MAVT) to evaluate childbirth options in this MCDA (20). MAVT integrates individual preferences through a multi-attribute value function *v*, which quantifies how well each option achieves multiple goals that are represented by measurable attributes (20, 26).

The MAVT process of modeling the preference parameters begins with a set of options $a=(a_{1},\;\ldots ,\;a_{n})$ and a set of goals, respectively their attributes, *i*. First, single-attribute value functions are required, $v_{i}$, which translate each option's performance on each goal into relative goal achievement values, $v_{i}(a_{i})$. These value functions can have linear or non-linear shapes, depending on individual preferences regarding changes in goal achievement. Typically, the resulting values are normalized to the interval [0, 1], where higher values indicate better alignment with individual preferences (20).

For decisions involving multiple goals, an overall value v(a) is calculated for each option by aggregating the relative goal achievement values $v_{i}(a_{i})$, and the weights $w_{i}$ that are assigned to each goal. These weights represent the individual preferences for making trade-offs between achieving the goals and reflect their relative importance. Most commonly, an additive model is used to calculate the overall values of the options $a=(a_{1},\;\ldots ,\;a_{n})$ as weighted arithmetic mean:

$$v(a)=\;\overset{n}{\underset{i=1}{\sum }}w_{i}\cdot v_{i}(a_{i})$$

where the weights $w_{i}>0$ and $\overset{n}{\underset{i=1}{\sum }}w_{i}=1$ (20). The option with the highest overall value is considered the best-performing choice from a rational decision-making perspective.

The additive model is intuitively easy to understand and widely used (20). However, it implies that an option's poor performance on one goal can be fully compensated by good performance on another goal. This assumption does not necessarily reflect stakeholders' actual preferences, particularly when the achievement of some goals is considered less compensable or even non-negotiable. In such cases, non-additive aggregation that limits compensation between goals may provide a more accurate representation (27).

**Figure 1:**
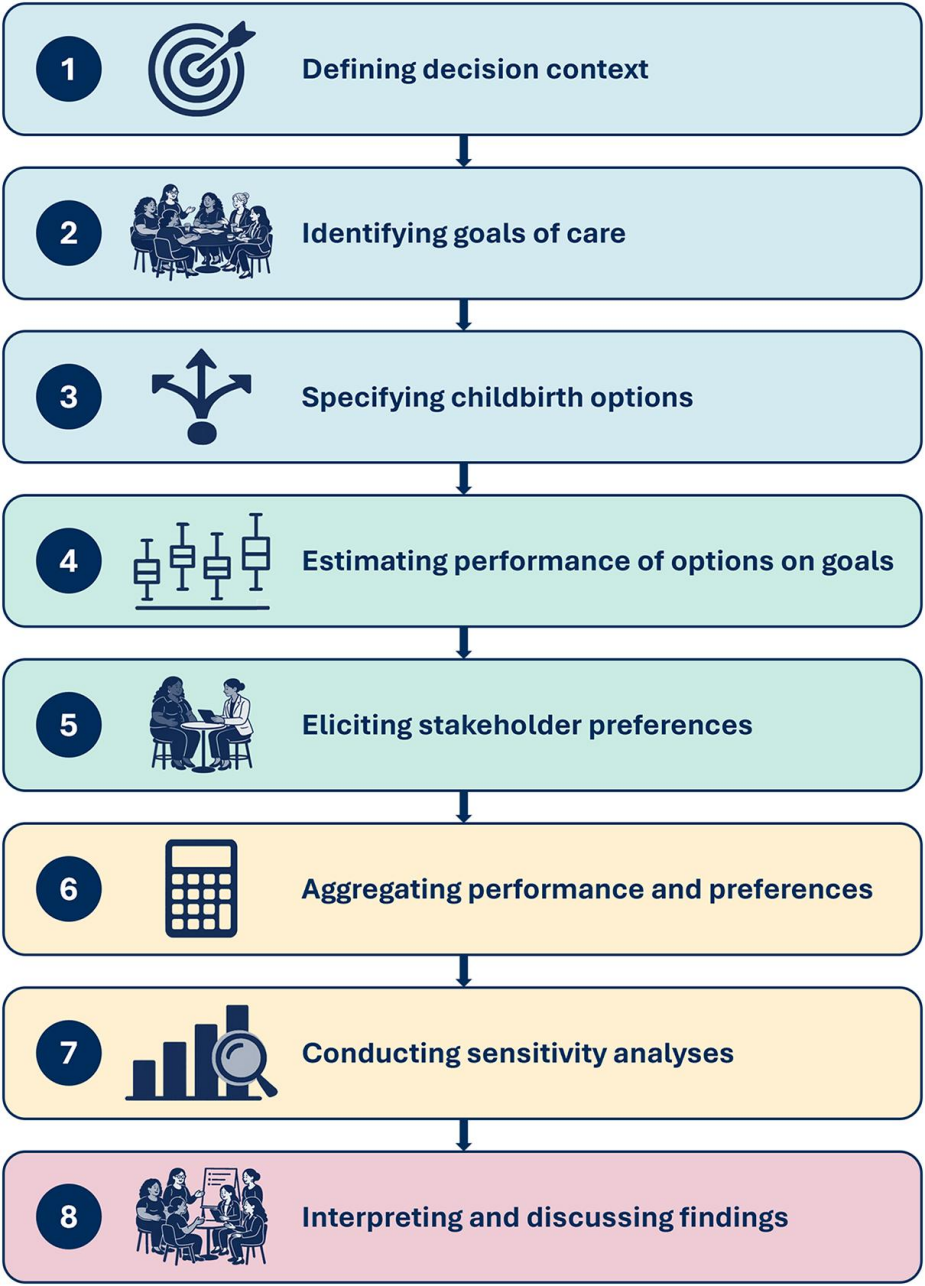
Overview of the MCDA approach used to evaluate childbirth options for women with obesity. MCDA, multi-criteria decision analysis.

Figure 1 illustrates a simplified overview of the MCDA approach employed in this study. We followed a stepwise procedure (20, 22):

1. Defining the decision context, key stakeholders, study population, and relevant obstetric scenarios.
2. Identifying the goals of care and selecting attributes to operationalize them.
3. Specifying the childbirth options.
4. Estimating each option's performance on each goal in each scenario, including uncertainty.
5. Eliciting and quantifying stakeholder preferences for making trade-offs between goals (i.e., weights) and for within-goal changes (i.e., value functions).
6. Calculating the options' overall values for each stakeholder in each scenario, including uncertainty, by aggregating the performance of the options across all goals in each scenario [cf. step 4] and the stakeholder preferences [cf. step 5].
7. Conducting sensitivity analyses on weights, value functions, and the aggregation model.
8. Interpreting overall values, their uncertainty and robustness, and discussing findings.

### 2.2 Decision context, key stakeholders, study population, and obstetric scenarios [cf. 2.1, step 1]

We framed decision-making on childbirth options as a collaborative process involving the pregnant women, midwives, and obstetricians as key stakeholders. In this process, the stakeholders engage in discussions about childbirth care goals and options, with the women making the final decision. Thus, this study was designed to explore a novel approach to support shared deliberations on childbirth care rather than to prescribe formulaic decision-making [cf. (28)].

The study population included women with preconceptional obesity (BMI ≥30kg/m^2^) and singleton pregnancies in cephalic vertex presentation, giving birth at term (37 + 0 to 41 + 6 weeks) in Swiss hospitals. Women with documented respiratory, urological/nephrological, neurological, or coagulation disorders, and cases involving fetal anomalies, fetal congenital malformations, or fetal death before hospital admission were excluded. To focus on genuine choices between options, we also excluded women with medically compelling indications for cesarean birth (Supplementary Material S1-1). To examine how the performance of options may depend on potentially relevant clinical factors (15, 29), we analyzed four obstetric scenarios (Table 1).

**Table 1:** Obstetric scenarios.

Scenario	Cesarean birth history	Comorbidities^a^	Description
1	No	No	No cesarean birth history or comorbidities
2	No	Yes	Comorbidities only
3	Yes	No	Cesarean birth history only
4	Yes	Yes	Both cesarean birth history and comorbidities

a Comorbidities included diabetes mellitus and/or hypertensive disorders.

### 2.3 Goals of care and childbirth options [cf. 2.1, steps 2 and 3]

The goals included in this MCDA were derived from a preceding multi-stakeholder study on fundamental goals of childbirth care for women with obesity employing an embedded mixed-methods design, as detailed in Wyss et al. (10). This earlier study involved women with obesity, midwives, and obstetricians and identified six top-level and eight lower-level goals reflecting stakeholders' underlying values and priorities across biomedical, physiological, psychosocial, and resource-related dimensions of care (10, 30). Conceptually, these goals align with widely recognized pillars of high-quality childbirth care, such as established in World Health Organization frameworks [e.g., (1, 11)]. While the goal set was developed to be comprehensive within the specific decision context, it was not intended to be exhaustive or exclusively relevant for women with obesity. Rather, it represents a structured and stakeholder-informed basis for multidimensional evaluation of key considerations of care quality. Table 2 outlines the identified goals of childbirth care, and their operationalization for this MCDA.

**Table 2:** Goals of childbirth care for women with obesity (10), and their operationalization.

Top-level goal	Lower-level goal	Short label	Type of attribute	Indicator(s)/Expert assessment	Unit (range)
A. High biomedical safety	A1. Low maternal complication rates	Maternal complications (A1)	Constructed attribute/Weighted composite index	Postpartum hemorrhage, obstetric anal sphincter injury, postpartum infection, obstetric wound complication, thromboembolic event	Value (0–1)
	A2. Low neonatal complication rates	Neonatal complications (A2)	Constructed attribute/Weighted composite index	Primary adaptation problem [hypothermia (<35.5 °C), hypoglycemia (<2 mmol/L), respiratory distress syndrome, Apgar score at 5 min <7], advanced neonatal acidosis with arterial umbilical pH <7.1, birth trauma, neonatal infection	Value (0–1)
B. Undisturbed physiological processes	B1. Physiological labor and childbirth processes	Physiological childbirth processes (B1)	Constructed attribute/Weighted composite index	Spontaneous onset of labor, spontaneous progression of labor, childbirth without anesthesia, no iatrogenic obstetric wound, spontaneous birth, vaginal birth	Value (0–1)
	B2. Positive initiation of bonding and breastfeeding after childbirth	Breastfeeding initiation (B2)	Proxy indicator	Exclusive breastfeeding at hospital discharge	% (0–100)
C. Positive psychosocial experience	C1. Positive psychosocial experience of care interactions and events	Psychosocial care experience (C1)	Expert assessment	Expected psychosocial care experience	Scale (0–100)
D. Low physical strain	D1. Low physical strain for care providers	Physical strain (D1)	Expert assessment	Expected physical strain for care providers	Scale (0–10)
E. Low resource use	E1. Low resource use in care setting	Resource use (E1)	Constructed attribute/Clinical indicator scoring system	Gestational age, multiple pregnancy, medical condition, labor interventions, childbirth mode, perineal/vaginal/cervical tear, postpartum hemorrhage, Apgar score at 5 min, birth weight, congenital anomaly, perinatal death, emergency procedures, intensive care needs	Score (5–54)
F. Low costs	F1. Low direct costs to the healthcare system	Direct costs (F1)	Constructed attribute	SwissDRG cost weight	Cost weight (0.553–1.405)

Goals of childbirth care for women with obesity have first been published by Wyss et al. (10).

Five childbirth options were specified based on childbirth modes and models of care (Table 3; Supplementary Material S1-2). We included the childbirth mode because women with obesity often experience prelabor or intrapartum cesarean birth, with biomedical obstetric factors alone not fully explaining this association (15). The model of care was considered due to the suggested benefits of continuous midwifery care on childbirth outcomes (19).

**Table 3:** Characteristics of the childbirth options.

Childbirth option	Short label	Childbirth mode	Model of care		
			Midwifery care during labor/childbirth	Obstetricians at childbirth	Familiarity of women with care providers
Vaginal birth with standard hospital care	VB standard	Vaginal birth	Standard hospital midwifery care	Typically present	Care providers typically unknown
Vaginal birth with continuous midwifery care	VB midwifery	Vaginal birth	One-to-one continuous midwifery care	Not necessarily present	Midwives known from prenatal care; obstetricians typically unknown
Intrapartum cesarean birth with standard hospital care	Intrapartum CB standard	Cesarean birth after onset of active labor and/or rupture of membranes	Standard hospital midwifery care	Perform the cesarean	Care providers typically unknown
Intrapartum cesarean birth with continuous midwifery care	Intrapartum CB midwifery	Cesarean birth after onset of active labor and/or rupture of membranes	One-to-one continuous midwifery care	Perform the cesarean	Midwives known from prenatal care; obstetricians typically unknown
Prelabor cesarean birth	Prelabor CB	Cesarean birth before onset of active labor and rupture of membranes	Typically standard hospital midwifery care	Perform the cesarean	Care providers typically unknown

See Supplementary Material S1-2 for details on the childbirth options.

### 2.4 MCDA inputs: performance estimates [cf. 2.1, step 4]

To implement the MCDA, the performance of each childbirth option on each goal in each scenario was estimated using attributes, which were constructed from one or more underlying indicators where applicable (Table 2). Different methods were applied depending on data availability. We analyzed Swiss hospital inpatient data from 22,464 childbirths between 2005 and 2022 among women meeting the study's inclusion criteria to assess the options' performance on the goals related to maternal and neonatal complications (A1/2), physiological childbirth processes (B1), breastfeeding initiation (B2), resource use (E1), and direct costs (F1) (sample characteristics Supplementary Material S1-3). These data were provided by the Swiss Obstetric Study Group (31). Dataset information are available in Wyss et al. (15).

For the goal on maternal complications (A1), we constructed an attribute by integrating multiple clinically relevant maternal complication indicators into a weighted composite index (“mini-MCDA”), with values ranging from 0 (“worst” composite outcome) to 1 (“best” composite outcome). This approach was used to represent the inherently multidimensional construct of maternal complications, rather than relying on a single proxy outcome. First, we estimated the predicted probabilities and associated uncertainties for postpartum hemorrhage, obstetric anal sphincter injury, postpartum infection, obstetric wound complication, and thromboembolic event (Table 2; Supplementary Material S1-3) for each option in each scenario using logistic regressions on the Swiss hospital inpatient data. We adjusted for maternal age, ethnicity, parity, smoking status, and year of delivery to control for potential confounders. Next, we transformed these probabilities to a 0–1 value scale. To do this, we elicited value functions for each indicator from three experts in structured interviews in August 2023 using the bisection method (20). The experts provided the midvalues for the intervals [*v* = 0; *v* = 1], [*v* = 0; *v* = 0.5], and [*v* = 0.5; *v* = 1], facilitated by a graphical ruler indicating the range from worst to best possible outcomes (32). Consistency of answers was checked, and discrepancies resolved through reiteration (32). The resulting value functions were mostly non-linear with slightly decreasing marginal values. From the same experts, we also elicited weights reflecting the relative importance of indicators using the reverse swing method to account for outcome ranges (32), with consistency checks performed by pairwise comparisons. The value functions and weights were pooled across experts for the weighted composite index. Furthermore, we asked the experts whether the compensation assumption of the additive aggregation model holds [cf. 2.1], using graphical illustrations and simple examples. They all disagreed. Thus, we aggregated the predictions on the maternal complication indicators, value functions, and weights using a non-additive power-mean aggregation with *γ* = 0.2 in the ValueDecisions application (26) [cf. 2.6 for details]. The uncertainty associated with the indicator predictions was transformed into a single uncertainty distribution using 2,000 Monte Carlo simulations. As input into the main MCDA, we used the resulting mean overall value of each option and characterized its aggregated uncertainty as normally distributed with a standard deviation of the 95% confidence interval from the simulations divided by 3.92. For sensitivity analyses, we additionally implemented a model with linear value functions and a model using an additive aggregation with *γ* = 1 [cf. 2.7].

Analogous to the goal on maternal complications (A1), we constructed weighted composite indices (“mini-MCDAs”) for the goals on neonatal complications (A2) and physiological childbirth processes (B1), using the same approach to integrate multiple clinically relevant indicators. For neonatal complications, indicators included primary adaptation problem, advanced neonatal acidosis, birth trauma, and neonatal infection (Table 2; Supplementary Material S1-3). We elicited value functions and weights for these indicators from three experts as outlined above. Indicators of physiological childbirth processes included spontaneous onset of labor, spontaneous progression of labor, childbirth without anesthesia, no iatrogenic obstetric wound, spontaneous birth, and vaginal birth (Table 2; Supplementary Material S1-3). Again, we obtained weights for these indicators from three experts, two of whom also provided value functions. Overall, a total of six purposively selected experts in maternal and neonatal care with at least nine years of professional experience generated the three weighted composite indices (expert characteristics Supplementary Material S1-4). The intention was to draw on informed professional judgments to operationalize the complex constructs of biomedical safety (A1/2) and physiological childbirth processes (B1), which are not directly observable. Experts were exclusively involved in this step because the technical nature of the indicators would likely have made them difficult for non-specialists to interpret and weigh accurately, and to reduce stakeholder burden in terms of cognitive complexity (33) and limited time resources. Such purposive expert elicitation has been an established approach in MCDA modeling, particularly when addressing multi-layered goals [cf. (34)]. To enhance transparency and assess robustness, all inputs and outputs, as well as the sensitivity analyses of the weighted composite indices for the goals on maternal complications (A1), neonatal complications (A2), and physiological childbirth processes (B1) are provided in Supplementary Material S1-5 to S1-16.

The goal on breastfeeding initiation (B2) was operationalized using Swiss hospital inpatient data on exclusive breastfeeding at hospital discharge. We selected this proxy indicator to reflect breastfeeding success after childbirth in line with the underlying goal of care (10) and because early breastfeeding outcomes may be most directly linked to the childbirth options considered in this study. Predicted exclusive breastfeeding probabilities at hospital discharge, ranging from 0% (“worst”) to 100% (“best”), and associated uncertainties were estimated for each option in each scenario using logistic regressions, adjusting for the same confounders as described above.

To operationalize the goal on resource use (E1), we adopted a clinical indicator scoring system inspired by Ball et al. (35) (Supplementary Material S1-17). The research team, in consultation with a clinical expert and a maternity staffing specialist, deemed the scoring system appropriate for reflecting the complexity of childbirth cases and their resource needs in terms of staffing, infrastructure, and equipment, based on key clinical factors (Table 2). The lowest attainable score for cases that met the study's inclusion criteria was 5 (lowest/“best” resource use), and the highest attainable score was 54 (highest/“worst” resource use). The score was calculated for each childbirth in the Swiss hospital inpatient dataset described earlier. We then computed the predicted scores and associated uncertainties for the subgroups of women having had the same childbirth option in each scenario using linear regressions and controlling for potential confounders.

As attribute for the goal on direct costs (F1), we used the SwissDRG cost weights of assigned case groups, which reflect the relative average treatment cost within the Swiss hospital inpatient tariff structure (36). Given that absolute cost estimates are calculated as base rates × cost weights, and that base rates are negotiated at the hospital level and may vary across institutions, these estimates are not directly comparable within the Swiss healthcare system. Therefore, SwissDRG cost weights were used as standardized proxy for comparing relative direct costs across childbirth options. Direct costs to the Swiss healthcare system were considered, as women do not incur cost sharing for standard healthcare from the 13th week of pregnancy until 8 weeks postpartum in Switzerland (37). Due to the lack of diagnosis-related group (DRG) information in the Swiss hospital inpatient data, we conservatively estimated the expected SwissDRG cost weight for each childbirth for a median length of hospital stay by coding the available clinical information from the Swiss Obstetric Study Group (31) according to SwissDRG 12.0/2023 (38). The considered SwissDRG cost weights ranged from 0.553 (lowest/“best”) to 1.405 (highest/“worst”). The predicted average SwissDRG cost weights and associated uncertainties were then calculated for each option in each scenario using linear regressions, again adjusting for the above-mentioned confounders.

In the absence of suitable quantitative data [cf. (22, 24)], expert assessments were used to estimate the options' performance on the psychosocial care experience (C1) and the physical strain for care providers (D1) to enable the inclusion of these relevant but quantitatively understudied childbirth care considerations. Between July and September 2023, five experts with at least nine years of professional experience were purposively recruited based on their clinical and/or research expertise in childbirth care for women with obesity and related domains (expert characteristics Supplementary Material S1-18). The elicitation followed a protocol consistent with established approaches in decision analysis (39) to derive probability distributions of the performance estimates for the selected goals across all childbirth options, but independent of the scenarios. Using a structured questionnaire and the quantile elicitation method (39), the experts provided the 5%, 95%, 50%, 25%, and 75% quantiles on a 0–100 scale for psychosocial care experience (0 = extremely negative/“worst” to 100 = extremely positive/“best”), and on a 0–10 scale for physical strain for care providers (0 = no physical strain/“best” to 10 = maximum physical strain/“worst”). Additionally, they rated their certainty about their estimates using a verbal scale with associated probabilities and graphical representations (“extremely uncertain” = 95% uncertain to “extremely certain” = 5% uncertain) (40, 41). Experts received written instructions, an illustrative example, and were offered optional in-person guidance to support their understanding of the elicitation tasks. To reduce the influence of individual assessments, estimates from all experts were combined to inform the main MCDA. We used a triangular distribution with the mode of the 50% quantiles, the minimum of 5% quantiles, and the maximum of 95% quantiles. Potential variability and uncertainty in expert assessments were addressed by using these conservative probability distributions and propagating them through extensive Monte Carlo simulations.

All statistical analyses of the performance estimates were conducted using Stata/MP 15.1 (42).

### 2.5 MCDA inputs: stakeholder preferences [cf. 2.1, step 5]

To examine how the options' overall performance may vary in response to stakeholder preferences for making trade-offs between the goals, we adopted a two-pronged approach using hypothetical and real stakeholder preferences. First, we tested the sensitivity of the MCDA results to extreme preference profiles of four hypothetical stakeholders [cf. (43)]: The *biomedical safety enthusiast* strongly prioritized minimizing maternal and neonatal risks and somewhat valued low physical strain for care providers, while disregarding the physiological processes, psychosocial care experience, resource use, and direct costs. The *natural birth proponent* emphasized undisturbed physiological processes without completely neglecting biomedical safety and the psychosocial care experience. The *psychosocial experience advocate* prioritized the psychosocial aspects of childbirth care while also valuing biomedical safety, particularly for the child. The *resource utilization pragmatist* focused on care setting resource use, emphasizing minimal staffing, equipment, and infrastructure needs, but still acknowledged the importance of biomedical safety, low physical strain for providers, and low direct costs to sustain resources. Accordingly, we assigned these hypothetical stakeholders extreme weights and assumed linear value functions for the main MCDA model.

To exemplify the impact of actual preferences on the overall performance of options, we elicited preference profiles of three real stakeholders directly involved in childbirth decisions, namely a woman with obesity, a midwife, and an obstetrician (Supplementary Material S1-19). These preferences were intended to be illustrative rather than representative of the broader stakeholder population. Between February and May 2024, facilitated structured interviews were conducted to determine the stakeholders’ value functions and weights, as well as the aggregation model using the same procedures as described for constructing the weighted composite indices with the experts [cf. 2.4]: We elicited value functions using the bisection method (20) for all goals except those operationalized by the weighted composite indices (goals A1/A2/B1). For them, we assumed linear value functions in the main MCDA model, as expert-derived non-linear functions had already been integrated to construct them. Weights were elicited using the reverse swing method (32), and consistency checks were performed using the swing method (20). No inconsistencies were observed. To avoid splitting bias (44), we first assessed top-level goal weights before addressing lower-level goal weights (32). Finally, we asked the stakeholders about their preferences for compensation between goals [cf. 2.1 and 2.4]. All stakeholders disagreed with the compensation assumption of the additive aggregation model. Each interview lasted two to three hours. Throughout, we provided written and visual representations including outcome ranges for each goal and used graphical illustrations to explore compensation.

### 2.6 Aggregation with ValueDecisions [cf. 2.1, step 6]

We implemented the MCDA using MAVT in the ValueDecisions application by Haag et al. (26), an open-source R-based software designed for integrating multidimensional goals, performance estimates, including their uncertainty, and stakeholder preferences. ValueDecisions also facilitated detailed sensitivity analyses of the stakeholder weights, value functions, and the aggregation model (26). After validating performance predictions, weights, and value functions, we aggregated the input data to compute the overall value of each childbirth option across all goals (A1 to F1) for each stakeholder's preferences in each scenario. Given that all real stakeholders disagreed with the compensation assumption of the additive aggregation model, we applied a non-additive power-mean aggregation with *γ* = 0.2. For details on power-mean aggregation, see Haag et al. (45). Compared to additive aggregation (*γ* = 1), lower values of *γ* reduce the degree to which poor performance on one goal can be offset by good performance on another, thereby allowing trade-offs while limiting extreme compensation across goals. This approach had been considered meaningful in other decision-making contexts [e.g., (34, 46)]. We accounted for uncertainty in the options' goal performance estimates by 2,000 Monte Carlo simulations, drawing from the uncertainty distributions of the predictions and propagating this uncertainty to the results. Based on these simulations, we computed the mean, 5%, and 95% quantiles of the options' overall values for each stakeholder in each scenario. Additionally, we used the “x vs. y” functionality in ValueDecisions to visualize the trade-off between the goal on direct costs to the healthcare system (F1) and the aggregated value of all other goals (A1 to E1) (26), as a complementary analysis to illustrate cost-benefit profiles of the options beyond the main MCDA aggregation.

### 2.7 Sensitivity analyses [cf. 2.1, step 7]

Hypothesizing four stakeholder preference profiles with extreme weights served as an initial sensitivity analysis to examine how the MCDA results may respond to highly divergent preferences for making trade-offs between the goals. Additionally, we conducted a series of local sensitivity analyses to assess the robustness of MCDA results to further uncertainty in stakeholder preferences. Sensitivity analyses of real stakeholder weights in ValueDecisions provided a visual representation of how the overall values of the options changed as the weight of a single goal increased/decreased while the weights of other goals were renormalized (26). We also tested the sensitivity of the results to changes of the value function curvature to more concave with *c* = 2 and to linear with *c* = 0. The latter analysis incorporated the sensitivity model predictions of the weighted composite indices (goals A1/A2/B1) which were calculated with linear value functions instead of expert-derived non-linear functions [cf. 2.4] (Supplementary Material S1-7, S1-11, and S1-15). To assess the sensitivity to the aggregation model, we employed the most commonly used additive aggregation with *γ* = 1. In this analysis, we used the sensitivity model predictions of the weighted composite indices (goals A1/A2/B1) which were also aggregated using additive models with *γ* = 1 [cf. 2.4] (Supplementary Material S1-8, S1-12, and S1-16).

### 2.8 Ethics approval and consent to participate

In a clarification of responsibility, the Ethics Committee for Research of the Canton of Bern, Switzerland, declared that the study did not fall within the scope of the Swiss Human Research Act (2011), art. 2, para. 1 (47). The Swiss hospital inpatient data had been anonymized and irreversibly de-identified before being transferred to the researchers, and no new sensitive health-related personal data were collected. The Ethics Committee did not consider the collection of expert assessments and individual preferences to fall under the Swiss Human Research Act (2011), art. 2, para. 1 (47), and therefore waived formal ethical approval (decision BASEC-No. Req-2019-00531). All participating experts and stakeholders received written information about the study and provided informed consent prior to data collection.

## 3 Results

### 3.1 MCDA inputs

#### 3.1.1 Performance predictions

**Figure 2:**
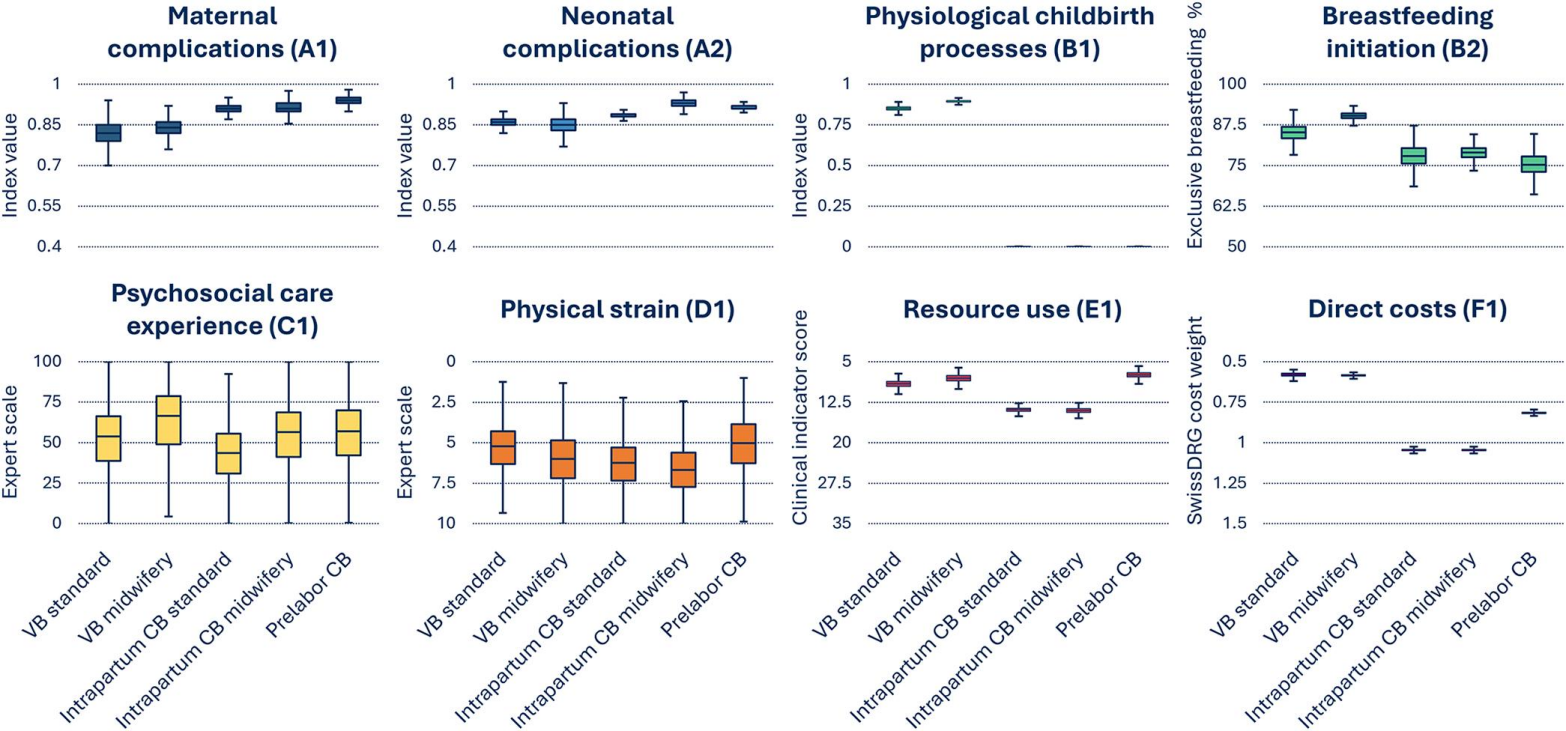
Performance predictions of how well childbirth options achieve the operationalized goals for women with no cesarean birth history or comorbidities (scenario 1). Uncertainty in the predictions was accounted for by 2,000 Monte Carlo simulations. *Y*-axes: Predicted outcomes (attribute levels); ranges approximately scaled to the minimum and maximum performance predictions for each goal; axis values ordered so that higher-positioned boxes represent better goal performance. *X*-axes: Childbirth options; VB standard: Vaginal birth with standard hospital care; VB midwifery: Vaginal birth with continuous midwifery care; Intrapartum CB standard: Intrapartum cesarean birth with standard hospital care; Intrapartum CB midwifery: Intrapartum cesarean birth with continuous midwifery care; Prelabor CB: Prelabor cesarean birth. The boxplots indicate the lower quartile (Q1), median (Q2), and upper quartile (Q3). The whiskers represent the minimum and the maximum values within 1.5 times the interquartile range (IQR; Q3–Q1). Prediction matrices and performance predictions for scenarios 1–4 are provided in Supplementary Material S2-1.

Performance predictions with uncertainty for the eight goal-related attributes indicated trends in goal achievement across the childbirth options (Figure 2, scenario 1; Supplementary Material S2-1). These trends were largely consistent across obstetric scenarios. Stakeholder preferences had not yet been incorporated at this stage. The performance predictions for physiological childbirth processes (B1), resource use (E1), and direct costs (F1) most effectively differentiated between vaginal and cesarean birth options. Both vaginal birth options outperformed all cesarean birth options regarding physiological childbirth processes (B1) and direct costs (F1). In terms of resource use (E1), vaginal birth and prelabor cesarean birth performed similarly well and distinctly better than the intrapartum cesarean birth options. No clear performance differences across goals were observed between the models of standard hospital care and continuous midwifery care within the same childbirth mode, whether vaginal birth or intrapartum cesarean birth.

All options performed comparably on maternal and neonatal complications (A1/A2) in most scenarios. However, in women with both a cesarean birth history and comorbidities (scenario 4), vaginal birth with continuous midwifery care slightly underperformed on maternal and neonatal complications (A1/A2) compared to most cesarean birth options (Supplementary Material S2-1h). Additionally, high uncertainty in the scenario-independent performance predictions for psychosocial care experience (C1) and physical strain (D1) obscured potential differences in the ability of options to achieve these goals. Consequently, the performance predictions for goals on maternal and neonatal complications (A1/A2), psychosocial care experience (B1), and physical strain (D1) did not help discriminate between the options for most scenarios. Omitting these goals in the respective MCDA models could have been methodologically justifiable. However, given the established importance of biomedical safety and the care experience for childbirth decisions (10, 48), the goals were retained in all subsequent MCDA models.

#### 3.1.2 Stakeholder preferences

**Figure 3:**
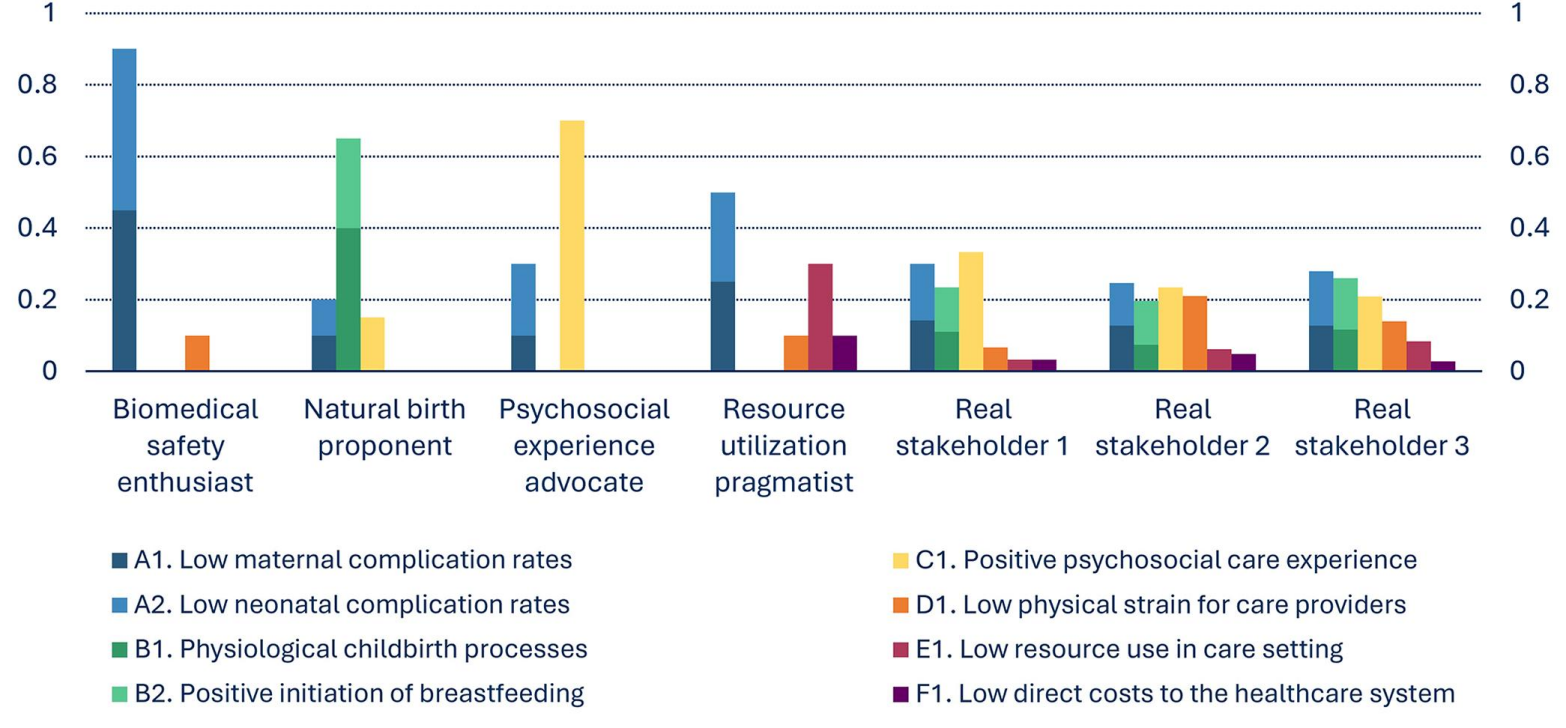
Stakeholder weights assigned to lower-level goals (colors) and top-level goals (bars). Extreme weights of hypothetical stakeholders: biomedical safety enthusiast, natural birth proponent, psychosocial experience advocate, resource utilization pragmatist. Illustrative weights elicited from real stakeholders involved in childbirth decisions: a midwife (real stakeholder 1), an obstetrician (real stakeholder 2), and a woman with obesity (real stakeholder 3), in order of interviews. For each stakeholder, the sum of the weights is defined as 1. Identical stakeholder weights were used to compare scenarios 1–4. Numerical values are provided in Supplementary Material S2-2a.

Hypothetical stakeholders displayed extreme goal weights as intended, whereas real stakeholders assigned more balanced weights across goals (Figure 3; Supplementary Material S2-2a). Real stakeholders tended to prioritize the biomedical, physiological, and psychosocial goals (A1 to C1) over the more structural goals on resources (D1 to F1), though rankings and absolute weights varied. The value functions elicited from real stakeholders were in a few cases linear to mostly slightly concave, with curvature up to a maximum of approximately *c* = 2, indicating decreasing marginal value for most attributes (Supplementary Material S2-2).

### 3.2 MCDA results

#### 3.2.1 Overall values of options

**Figure 4:**
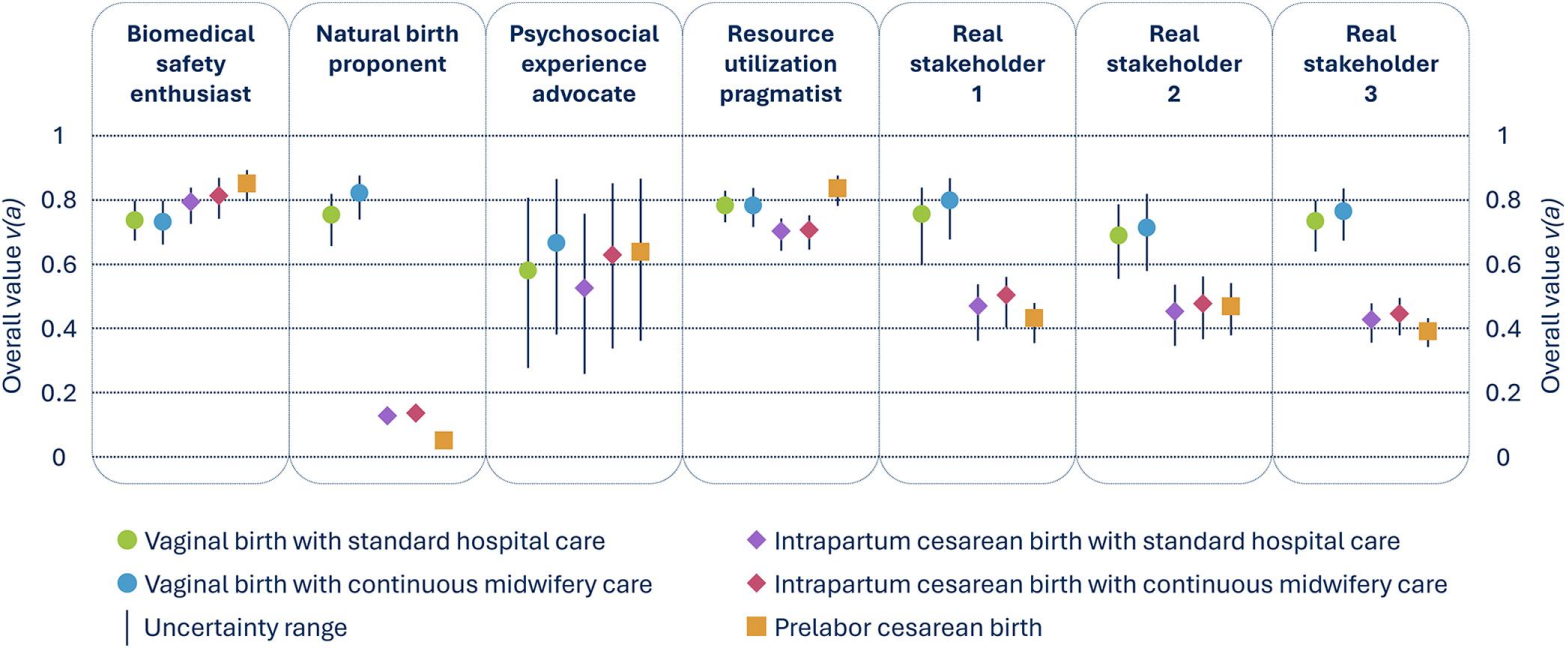
Mean overall values (*y*-axis) for each option (*x*-axis) and each stakeholder (boxes) for women with no cesarean birth history or comorbidities (scenario 1), using a non-additive power-mean aggregation with *γ* = 0.2. Values are normalized to the interval [0, 1], with higher values indicating better overall performance aligned with individual preferences. Uncertainty ranges represent the 5% and 95% quantiles of the overall values (bars) derived from 2,000 Monte Carlo simulations. Non-overlapping uncertainty ranges suggest considerable differences between the overall values of options, whereas overlap indicates that differences should be interpreted with caution. Figures of scenarios 1–4 and corresponding numerical values are provided in Supplementary Material S3-1.

The vaginal birth options consistently performed best and achieved clearly higher overall values compared to the cesarean birth options for all three real stakeholders, and for the hypothetical stakeholder *natural birth proponent*, when aggregating performance predictions and stakeholder preferences (Figure 4, scenario 1). Thereby, higher mean overall values suggested better performance aligned with individual preferences, while non-overlapping uncertainty ranges pointed to considerable differences between the childbirth modes. For the extreme weights of the other hypothetical stakeholders, all childbirth options yielded similarly high overall values. Although their ranking of options varied, the uncertainty ranges for the vaginal and cesarean birth options mostly overlapped, indicating that differences in overall values should be interpreted with caution. Thus, no single childbirth option emerged as clearly best for these hypothetical stakeholders. Additionally, we found no substantial differences in overall performance between the options with standard hospital care and continuous midwifery care within the same childbirth mode, whether vaginal birth or intrapartum cesarean birth, across all stakeholder preferences. These results remained largely consistent across all obstetric scenarios (Supplementary Material S3-1, scenarios 1–4).

A notable exception was observed for women with both a cesarean birth history and comorbidities (scenario 4) under extreme weights for biomedical safety. In this case, prelabor cesarean birth and intrapartum cesarean birth with continuous midwifery care performed marginally better than vaginal birth with continuous midwifery care when accounting for the non-overlapping uncertainty ranges (Supplementary Material S3-1d). However, the absolute differences between the options' overall values were still small [e.g., *ν* = 0.694 (0.634–0.740) for VB midwifery, *ν* = 0.814 (0.747–0.865) for intrapartum CB midwifery, and *ν* = 0.835 (0.779–0.878) for prelabor CB].

#### 3.2.2 Cost-benefit visualizations

**Figure 5:**
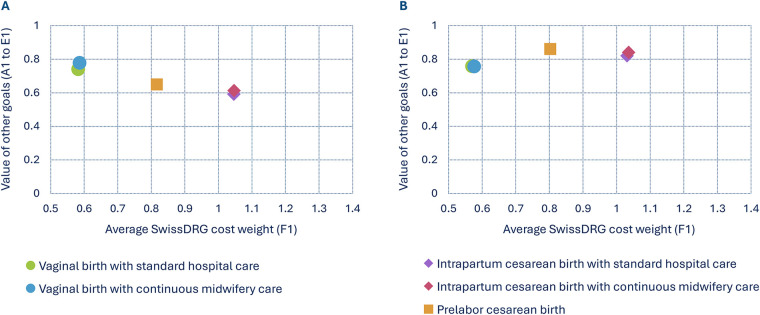
**(A)** Cost-benefit visualization based on the preferences of real stakeholder 3 for women with no cesarean birth history or comorbidities (scenario 1). **(B)** Cost-benefit visualization based on the preferences of hypothetical stakeholder *biomedical safety enthusiast* for women with cesarean birth history but no comorbidities (scenario 3). *X*-axis: Direct costs to the healthcare system represented by average SwissDRG cost weight (goal F1); range scaled to the minimum and maximum SwissDRG cost weight. *Y*-axis: Aggregated value of all other goals (A1 to E1), reflecting benefits and calculated using the MCDA model described in section 2.6; values are normalized to the interval [0, 1], with higher values indicating better overall performance aligned with individual preferences. Figures for all stakeholders across scenarios 1–4 are provided in Supplementary Material S3-2.

For the moderate preferences of all three real stakeholders, the vaginal birth options offered greater benefits—reflected in higher aggregated overall values for goals A1 to E1—compared to the cesarean birth options, while incurring the lowest direct costs to the healthcare system as represented by average SwissDRG cost weights (Figure 5A, real stakeholder 3, scenario 1). Similar results were observed for the hypothetical stakeholder *natural birth proponent*. In contrast, prelabor cesarean birth yielded higher benefits than vaginal birth for the extreme weights of the hypothetical stakeholders *biomedical safety enthusiast* and the *resource utilization pragmatist*, but at substantially increased direct costs (Figure 5B, *biomedical safety enthusiast*, scenario 3). The patterns for all stakeholders remained stable across obstetric scenarios (Supplementary Material S3-2).

#### 3.2.3 Sensitivity analyses

Sensitivity analyses of the real stakeholder weights using ValueDecisions (26) confirmed that the rankings of the highest-value options—the vaginal birth options—would change only in case of extreme weights (Supplementary Material S3-3). Overall values tended to converge with extreme weights, except for the goals related to physiological childbirth processes (B1) and direct costs (F1), for which the vaginal birth options performed markedly better than the cesarean birth options.

Sensitivity analyses on the shape of the value functions and the aggregation model generally supported the results of the main non-additive aggregation model with elicited stakeholder value functions (Supplementary Material S3-4 to S3-6). We observed largely robust patterns across the main model and sensitivity models with stable rankings of options. However, the overlap of the uncertainty ranges varied in some cases. The sensitivity model using additive aggregation resulted in closer overall values of the options, especially for the real stakeholders, leading to less clear distinctions between the vaginal and cesarean birth options for them (Supplementary Material S3-6). Nonetheless, the sensitivity analyses addressing potential uncertainty in preferences indicated that the vaginal birth options remained generally the highest-value options for all three real stakeholders and the hypothetical stakeholder *natural birth proponent* under most model assumptions. The sensitivity results also corroborated that no single childbirth option seemed clearly best for the remaining stakeholders. Again, the patterns of the sensitivity models remained largely consistent across all obstetric scenarios.

## 4 Discussion [cf. 2.1, step 8]

### 4.1 Summary of main findings

To our knowledge, this was the first study employing multi-criteria decision analysis (MCDA) using multi-attribute value theory (MAVT) in a maternity care context. We evaluated childbirth options for women with obesity by explicitly integrating diverse goals of care, empirical data on goal achievement, and individual stakeholder preferences for achieving these goals. This novel approach enabled a multidimensional value assessment that could support evidence-based and person-centered decision-making on high-quality childbirth care.

In the absence of compelling contraindications, vaginal birth with standard hospital care or continuous midwifery care achieved similar or distinctly higher overall values compared to cesarean birth across most considered stakeholder preferences and obstetric scenarios. Conversely, prelabor and intrapartum cesarean birth generally yielded comparable or lower overall values. While variations in preferences for making trade-offs between goals influenced the overall values of the cesarean birth options, they had less impact on the overall values of the vaginal birth options. Options with standard hospital care performed similarly to those with continuous midwifery care within the same childbirth mode. Cost-benefit visualizations further showed that the vaginal birth options provided high or the highest benefits at the lowest costs for all stakeholder preferences. In general, the performance of the childbirth options appeared to be largely consistent across the obstetric scenarios and robust across the sensitivity analyses. These insights may contribute to informing childbirth care decisions for women with obesity. The following discussion will explore their relevance to clinical practice and research, and address key methodological considerations.

### 4.2 Performance of childbirth options

#### 4.2.1 Childbirth modes

Consistent with current guidelines stating that obesity alone is not a medical indication for cesarean birth (7, 8), vaginal birth generally yielded high overall values across the considered scenarios and divergent preference profiles in our MCDA. Prelabor and intrapartum cesarean birth performed comparably well only under specific, extreme preferences. This finding contrasts with the high cesarean birth rates among women with obesity (2–4) that persist in the absence of compelling indications (15) (Supplementary Material S1-3). Building on previous research showing that this link between obesity and cesarean birth may be partly mediated by biomedical obstetric factors (15), we conducted value assessments of childbirth modes across stratified scenarios. Our results indicate that the overall values of the childbirth modes were not primarily dependent on maternal comorbidities and/or the women's cesarean birth history. Thus, these clinical factors might not solely justify decisions to perform a cesarean, unless compelling indications exist. In line with this finding, guidelines for the general birthing population do not specify diabetes mellitus, hypertensive disorders, or cesarean birth history as indications for cesarean birth (49–51), but recommend that maternal preferences should be considered (50, 51). While vaginal birth performed well across a wide range of preferences in our study, one possible exception concerned women with a cesarean birth history, comorbidities, and very strong preferences for biomedical safety. In such cases, a repeat cesarean birth could be a more favorable option than a vaginal birth with continuous midwifery care. Still, the differences in overall values between the childbirth options remained small under these extreme preferences, and no single option clearly outperformed all others [cf. 3.2.1] (Supplementary Material S3-1d). This result may reflect the partial sensitivity of the maternal and neonatal complication indices (goals A1/A2) to the combined presence of both cesarean birth history and comorbidities [cf. 3.1.1]. Similarly, a prior systematic review and meta-analysis suggested better clinical outcomes for planned repeat cesareans in women with BMI ≥40 kg/m^2^ and a previous cesarean birth, and proposed clinical equipoise regarding the childbirth mode for women without cesarean birth history (52). However, when multidimensional goals were considered in our study, vaginal birth generally appeared to achieve comparable or higher overall values, even among women with cesarean birth history in the absence of compelling indications for operative delivery. This insight corresponds with the recommendation that women with obesity who have had a previous cesarean birth should be offered to attempt a vaginal birth, taking into account its benefits and risks, and the women's individual needs (8).

Moreover, our findings suggest that the frequent cesarean births for non-compelling medical reasons among women with obesity (15) (Supplementary Material S1-3) are unlikely to be primarily driven by the women's and care providers' preferences for achieving diverse goals. Instead, they indicate a potential discrepancy between the good performance of vaginal birth options across highly divergent stakeholder preferences in our study and the childbirth decisions often made in clinical practice. This discrepancy might be due to a tendency in obstetric decision-making to focus on biomedical benefits and risks of childbirth modes (“alternative-focused thinking”) rather than a structured evaluation across multidimensional goals and individual preferences (“value-focused thinking”) (53). Yet, even when multiple goals and preferences are acknowledged, they might not be fully reflected in the actual childbirth care women receive. Many additional factors likely influence childbirth practices beyond explicitly stated goals and preferences, such as personal beliefs and attitudes, care philosophies, risk perceptions, clinical skills, convenience, financial incentives, or medicolegal considerations (16, 17, 54, 55). These complexities highlight the challenge of ensuring transparent, evidence-based, and person-centered decisions on childbirth modes and call for further exploration.

#### 4.2.2 Models of care

Adding insights into models of care for women at higher-risk of complications, we found that continuous midwifery care generally performed as well as standard hospital care for women with obesity within the same childbirth mode across the considered scenarios and stakeholder preferences. This finding aligns with a recent Cochrane review which reported at least similarly favorable biomedical, physiological, psychosocial, and economic outcomes for midwifery continuity models of care compared to other models in low-risk and mixed-risk populations (19). Midwifery continuity models are typically provided within an interdisciplinary consultation and referral network as needed (56). Some care may thus be delivered in collaboration with obstetricians or other specialties staff (19, 57). These conditions and our findings support the eligibility of women with obesity for continuous midwifery care, regardless of their obstetric risk status.

However, although previous research has linked midwifery continuity models with more positive care experiences (19, 58), our MCDA results did not show a clear advantage for any childbirth option in this regard. This may be due to the high uncertainty in the experts assessments of the psychosocial care experience (goal C1), likely reflecting its complex and multifaceted nature (59). Given the importance of the childbirth experience to high-quality childbirth care (10, 11, 48), further research is needed to examine how models of care might influence psychosocial outcomes, particularly for women with obesity.

#### 4.2.3 Benefits vs. direct costs

Corresponding to previous research on low-risk pregnancies (60), our cost-benefit visualizations showed that vaginal birth was associated with higher overall values at lower direct costs to the healthcare system for the moderate preferences of the real stakeholders. Even in cases of extreme preferences, both vaginal birth options consistently demonstrated favorable cost-benefit profiles in nearly all of the considered scenarios. These results indicate that vaginal birth may offer good value for money, not only in low-risk populations but potentially also among women at higher-risk for complications, such as women with obesity who may have a cesarean birth history and/or comorbidities. Nevertheless, these findings should be interpreted within the context of the MCDA framework and the use of a relative cost proxy, rather than as results of a formal economic evaluation. In light of the cost sensitivity of the healthcare sector, these findings could still be particularly relevant to ongoing discussions on value-based healthcare (61), and the potential for resource savings by avoiding unnecessary interventions (62).

### 4.3 Robustness of findings across stakeholder preferences

Recognizing that variations in preferences may influence what is considered a high-value childbirth option, we have emphasized the importance of accounting for potential heterogeneity in stakeholder preferences for person-centered decision-making (33). By conceptualizing the MCDA using hypothetical stakeholders with extreme weights, we explored how highly divergent preferences for trade-offs between goals might shift results. Real stakeholders were included to illustrate how actual preferences could fit within this framework, without intending them to be representative. In addition to examining variations in weights, we conducted extensive sensitivity analyses to address uncertainty in preferences for within-goal changes of goal achievement (value functions) and for compensation between goals (aggregation model). While consensus on preferences is not necessary for individual decisions (33), our key finding—that vaginal birth options generally offered high value across varying weights, value function changes, and aggregation models—could potentially contribute to simplifying decision-making on childbirth options for women with obesity.

Notably, the relatively balanced and moderate weights of the three real stakeholders yielded robust results not only for the high-value vaginal birth options, but for all childbirth options in our study. Although only illustrative, these weights may reflect the growing recognition of the biopsychosocial significance of childbirth care (48). In line with earlier research (10), we would expect that many women with obesity, midwives, and obstetricians would similarly weigh the goals of biomedical safety, undisturbed physiological processes, and the psychosocial experience of childbirth care. Other stakeholders such as hospital administrators, health economists, or policymakers might place more emphasis on resource use and costs. Still, it seems unlikely that they would do so at the complete expense of biopsychosocial goals, which could be deemed unethical or immoral [cf. (63, 64)]. This unwillingness of real stakeholders to trade-off substantial biopsychosocial value—known as “taboo trade-offs” or “protected values” (65, 66)—may limit extreme prioritization of resource related goals [cf. (67)]. Yet, even when simulating such extreme preferences, the vaginal birth options continued to perform well in the sensitivity analyses of our study. This robustness suggests that the findings may not be solely driven by the illustrative stakeholder preferences, but rather may reflect largely non-conflicting patterns in the underlying performance of the options across goals. It seems therefore reasonable to assume that even substantial heterogeneity in real stakeholder preferences would produce similar and mostly consistent overall MCDA results. However, the exploratory nature of this study and the very small and non-representative sample of stakeholders preclude population-level generalization. Future research should include larger and more diverse stakeholder samples to validate this hypothesis and to support policy-relevant conclusions.

### 4.4 Implications for clinical practice and research

Providing first empirical insights into the multidimensional value of childbirth options for women with obesity, this study highlights the relevance of supporting childbirth care decisions through alignment with individual goals and preferences. Within the context of our MCDA, avoiding unnecessary cesarean births in women with obesity may be associated with higher overall value in terms of biopsychosocial health outcomes and sustainability of maternity care resources across many obstetric scenarios and stakeholder preferences [cf. (68)]. For example, obesity has been shown to biomedically influence cesarean birth decisions primarily through slower labor progression and cesarean birth history (15). Thus, in the absence of compelling contraindications, allowing more time for labor (8) may be a potential approach to reduce first-time intrapartum cesarean births and, in turn, prevent repeat cesarean births in future pregnancies (15). However, unforeseen severe complications that require emergency interventions can occur during labor, especially among women with cesarean birth history and/or comorbidities (e.g., uterine rupture, eclampsia). Moreover, the model of care provided may influence if, how, and when decisions on cesarean birth are made. Midwifery continuity models have been proposed to increase physiological childbirth (69) and reduce operative deliveries (19). Future studies should therefore compare the value of planned prelabor cesarean birth and anticipated vaginal birth—whether actually achieved or not (52)—for women with obesity across obstetric scenarios and models of care. As many factors beyond those explored in this study may influence how childbirth decisions are made and implemented (16, 17, 54, 55), further research is also needed to better understand these clinical processes. Such work is essential to improve the quality of care provided to women with obesity.

### 4.5 Methodological limitations, strengths, and implications for future MCDA in childbirth care

Future applications of MCDA to childbirth care could be strengthened by refining the model structure, reducing prediction uncertainty, and extending the framework to encompass diverse maternity care contexts. Structurally, the current model may not have fully addressed all relevant goals, although they were based on a preceding multi-stakeholder study (10). For instance, we did not account for potential barriers to accessing continuous midwifery care, such as limited provider availability or the direct costs to women associated with midwives' on-call compensation. As these factors might affect the women's access to certain models of care, they emphasize the need to consider equity in access alongside safety, effectiveness, efficiency, and person-centeredness of care in future models (1). Moreover, structural improvement could be achieved by revising the attributes and indicators used to measure goal achievement. For example, the Swiss hospital inpatient data analyzed in our MCDA were constrained to the period from hospital admission to discharge. Thereby, we overlooked potentially important medium- and long-term consequences of options. This limitation may be particularly salient in the context of cesarean births, given their documented long-term implications for women's and children's health (68), especially for women with obesity and repeat cesarean births (13, 68). Refining goals and including longer time horizons in subsequent MCDA would allow for a more comprehensive evaluation of childbirth options.

In addition, future MCDA would benefit from reducing prediction uncertainty by obtaining more precise data. High uncertainty was evident in expert assessments of the psychosocial care experience (goal C1) and the physical strain for care providers (goal D1) in our study. This uncertainty contributed to substantial variability in the overall values generated by the Monte Carlo simulations. While the ability of MCDA to integrate expert assessments in the absence of more robust data is a recognized strength (22, 24) that enables the inclusion of otherwise unmeasured but relevant goals of care, it can introduce considerable prediction uncertainty and potentially weaken the conclusiveness of findings. Evaluating the options with respect to the psychosocial care experience may have been particularly affected by this limitation, as it was considered a key goal of childbirth care in our study and prior research (10, 48). Furthermore, although we followed a structured elicitation protocol consistent with established approaches in decision analysis (39), expert assessments remain subject to potential bias (44). Thus, they do not obviate the need for prospective empirical evidence on psychosocial care experience and physical strain for care providers. Future studies should aim to generate data on multidimensional outcomes to strengthen decision analyses and inform recommendations for achieving high-quality childbirth care.

Finally, it should be acknowledged that the empirical inputs into this MCDA originate from the Swiss maternity care context, a high-resource setting characterized by routine midwifery involvement, broad access to medical interventions, and mandatory health insurance. These characteristics likely differ across healthcare systems and may limit the direct transferability of the framework to other settings without context-specific adaptation.

However, our study also has notable strengths. We successfully adapted the MCDA approach using the value-focused perspective of MAVT (53) to evaluate childbirth options for women with obesity, offering a preliminary exploration of its possible merits. This approach enabled to reflect the fundamental values of both women and care providers by explicitly incorporating multidimensional goals. These goals, while grounded in stakeholder perspectives (10), also align with established pillars of high-quality childbirth care (1, 11), supporting their conceptual validity. Importantly, no previously identified goals (10) were excluded due to a lack of evidence or data availability (22, 24). Moreover, we scaled the models using both hypothetical and real stakeholder preferences to illustrate how the childbirth options perform from a person-centered perspective. In doing so, the study responded to contemporary standards emphasizing that high-quality care should account for individual goals and preferences (1). Recognizing that high-quality care also depends on scientific evidence and professional knowledge (1), we based our performance predictions on quantitative data from nearly 22,500 childbirths among women with obesity in Switzerland and drew on judgments from experienced experts. At the same time, the MCDA framework itself is adaptable and may be extended to other maternity care settings by incorporating context-specific goals, data, and stakeholder preferences into the model.

Guidelines often recommend considering women's “preferences”, “needs”, or “wishes”, yet they rarely offer guidance for integrating these individual factors into clinical decision-making (8, 9, 70). Here, our MCDA contributes a structured, quantitative approach to identifying person-centered, high-value options in a transparent, consistent, and rational way (20). In this sense, it shifts the focus from potentially conflicting discussions about childbirth options towards value-focused decision-making (53).

## 5 Conclusion

Decision-making on childbirth care should ideally consider multiple factors, including diverse goals, evidence-based information on benefits and risks of options relative to these goals, and individual preferences for achieving them. We employed multi-criteria decision analysis (MCDA) using multi-attribute value theory (MAVT) to evaluate childbirth options for women with obesity by explicitly integrating these factors. Our findings emphasize the importance of incorporating multidimensional goals and stakeholder preferences into decision-making on childbirth modes and models of care, alongside empirical data. Although cesarean birth rates are high among women with obesity, vaginal birth with standard hospital care or continuous midwifery care generally performed well across the considered obstetric scenarios and a wide range of stakeholder preferences in our study. While the findings should not be applied formulaically to prescribe decisions on childbirth care, insights from this exploratory MCDA may help inform clinical and scientific developments in the field. Future research could further strengthen the MCDA with structural refinements, prospective data inputs, and context extensions. By promoting transparency, consistency, and person-centeredness, this study presents a novel framework for supporting collaborative and deliberative decision-making on high-quality childbirth care for women with obesity that aligns with individual goals, preferences, and available scientific evidence.

## Data Availability

The datasets presented in this study can be found in the OLOS open research data repository (71). The Supplementary Material accompanying this article provides additional data and information to support the interpretation and reproducibility of the findings.
