# Supplementary material for: Evaluating childbirth options for women with obesity: a multi-criteria decision analysis
Source: Front Glob Womens Health. 2026 Jul 15;7:1822385. doi: 10.3389/fgwh.2026.1822385 (PMC13415362; doi:10.3389/fgwh.2026.1822385)
Supplement: Supplementary file 1 [file Datasheet1.pdf]

Evaluating childbirth options for women with obesity: a multi-criteria decision analysis

Supplementary Material

Authors and affiliations

Carmen Wyss,[1,2]* PhD, RM, [1]Applied Research and Development, Division of Midwifery, Department of Health Professions, Bern University of Applied Sciences, Bern, Switzerland. [2]Graduate School for Health Sciences, University of Bern, Bern, Switzerland. E-mail: carmen.wyss-zuercher@gmx.ch.

Judit Lienert,[3] PD, PhD, [3]Decision Analysis Group, Department of Environmental Social Sciences, Swiss Federal Institute of Aquatic Science and Technology (Eawag), Duebendorf, Switzerland. E-mail: judit.lienert@eawag.ch.

Evelyne M. Aubry,[1] PhD, [1]Applied Research and Development, Division of Midwifery, Department of Health Professions, Bern University of Applied Sciences, Bern, Switzerland. E-mail: evelyne.aubry@bfh.ch.

* Corresponding author

Contents

S1. Methods 6
S1-1. Study's inclusion and exclusion criteria 6
S1-2. Childbirth options 7
S1-2a. Childbirth options 7
S1-2b. Models of care 7
S1-3. Sample characteristics of the Swiss hospital inpatient data, Swiss Obstetric Study Group (8) 8
S1-4. Characteristics of experts who provided value functions and weights for weighted composite indices 9
S1-5. Inputs to the weighted composite index of maternal complication indicators 10
S1-5a. Prediction matrix for maternal complication indicators, scenario 1 10
S1-5b. Prediction matrix for maternal complication indicators, scenario 2 11
S1-5c. Prediction matrix for maternal complication indicators, scenario 3 12
S1-5d. Prediction matrix for maternal complication indicators, scenario 4 13
S1-5e. Preference matrix for maternal complication indicators, all scenarios 14
S1-6. Main model: Outputs of the weighted composite index of maternal complication indicators with expert value functions and γ=0.2 15
S1-6a. Overall values of options for maternal complication indicators with expert value functions and γ=0.2, scenario 1 15
S1-6b. Overall values of options for maternal complication indicators with expert value functions and γ=0.2, scenario 2 16
S1-6c. Overall values of options for maternal complication indicators with expert value functions and γ=0.2, scenario 3 17
S1-6d. Overall values of options for maternal complication indicators with expert value functions and γ=0.2, scenario 4 18
S1-7. Sensitivity analyses: Outputs of the weighted composite index of maternal complication indicators with linear value functions and γ=0.2 19
S1-7a. Overall values of options for maternal complication indicators with linear value functions and γ=0.2, scenario 1 19
S1-7b. Overall values of options for maternal complication indicators with linear value functions and γ=0.2, scenario 2 20
S1-7c. Overall values of options for maternal complication indicators with linear value functions and γ=0.2, scenario 3 21
S1-7d. Overall values of options for maternal complication indicators with linear value functions and γ=0.2, scenario 4 22
S1-8. Sensitivity analyses: Outputs of the weighted composite index of maternal complication indicators with expert value functions and γ=1 23
S1-8a. Overall values of options for maternal complication indicators with expert value functions and γ=1, scenario 1 23
S1-8b. Overall values of options for maternal complication indicators with expert value functions and γ=1, scenario 2 24
S1-8c. Overall values of options for maternal complication indicators with expert value functions and γ=1, scenario 3 25
S1-8d. Overall values of options for maternal complication indicators with expert value functions and γ=1, scenario 4 26
S1-9. Inputs to the weighted composite index of neonatal complication indicators 27
S1-9a. Prediction matrix for neonatal complication indicators, scenario 1 27

S1-9b. Prediction matrix for neonatal complication indicators, scenario 2 28
S1-9c. Prediction matrix for neonatal complication indicators, scenario 3 29
S1-9d. Prediction matrix for neonatal complication indicators, scenario 4 30
S1-9e. Preference matrix for neonatal complication indicators, all scenarios 31
S1-10. Main model: Outputs of the weighted composite index of neonatal complication indicators with expert value functions and γ=0.2 32
S1-10a. Overall values of options for neonatal complication indicators with expert value functions and γ=0.2, scenario 1 32
S1-10b. Overall values of options for neonatal complication indicators with expert value functions and γ=0.2, scenario 2 33
S1-10c. Overall values of options for neonatal complication indicators with expert value functions and γ=0.2, scenario 3 34
S1-10d. Overall values of options for neonatal complication indicators with expert value functions and γ=0.2, scenario 4 35
S1-11. Sensitivity analyses: Outputs of the weighted composite index of neonatal complication indicators with linear value functions and γ=0.2 36
S1-11a. Overall values of options for neonatal complication indicators with linear value functions and γ=0.2, scenario 1 36
S1-11b. Overall values of options for neonatal complication indicators with linear value functions and γ=0.2, scenario 2 37
S1-11c. Overall values of options for neonatal complication indicators with linear value functions and γ=0.2, scenario 3 38
S1-11d. Overall values of options for neonatal complication indicators with linear value functions and γ=0.2, scenario 4 39
S1-12. Sensitivity analyses: Outputs of the weighted composite index of neonatal complication indicators with expert value functions and γ=1 40
S1-12a. Overall values of options for neonatal complication indicators with expert value functions and γ=1, scenario 1 40
S1-12b. Overall values of options for neonatal complication indicators with expert value functions and γ=1, scenario 2 41
S1-12c. Overall values of options for neonatal complication indicators with expert value functions and γ=1, scenario 3 42
S1-12d. Overall values of options for neonatal complication indicators with expert value functions and γ=1, scenario 4 43
S1-13. Inputs to the weighted composite index of physiological childbirth indicators 44
S1-13a. Prediction matrix for physiological childbirth indicators, scenario 1 44
S1-13b. Prediction matrix for physiological childbirth indicators, scenario 2 45
S1-13c. Prediction matrix for physiological childbirth indicators, scenario 3 46
S1-13d. Prediction matrix for physiological childbirth indicators, scenario 4 47
S1-13e. Preference matrix for physiological childbirth indicators, all scenarios 48
S1-14. Main model: Outputs of the weighted composite index of physiological childbirth indicators with expert value functions and γ=0.2 49
S1-14a. Overall values of options for physiological childbirth indicators with expert value functions and γ=0.2, scenario 1 49
S1-14b. Overall values of options for physiological childbirth indicators with expert value functions and γ=0.2, scenario 2 50

S1-14c. Overall values of options for physiological childbirth indicators with expert value functions and γ=0.2, scenario 3 51
S1-14d. Overall values of options for physiological childbirth indicators with expert value functions and γ=0.2, scenario 4 52
S1-15. Sensitivity analyses: Outputs of the weighted composite index of physiological childbirth indicators with linear value functions and γ=0.2 53
S1-15a. Overall values of options for physiological childbirth indicators with linear value functions and γ=0.2, scenario 1 53
S1-15b. Overall values of options for physiological childbirth indicators with linear value functions and γ=0.2, scenario 2 54
S1-15c. Overall values of options for physiological childbirth indicators with linear value functions and γ=0.2, scenario 3 55
S1-15d. Overall values of options for physiological childbirth indicators with linear value functions and γ=0.2, scenario 4 56
S1-16. Sensitivity analyses: Outputs of the weighted composite index of physiological childbirth indicators with expert value functions and γ=1 57
S1-16a. Overall values of options for physiological childbirth indicators with expert value functions and γ=1, scenario 1 57
S1-16b. Overall values of options for physiological childbirth indicators with expert value functions and γ=1, scenario 2 58
S1-16c. Overall values of options for physiological childbirth indicators with expert value functions and γ=1, scenario 3 59
S1-16d. Overall values of options for physiological childbirth indicators with expert value functions and γ=1, scenario 4 60
S1-17. Clinical indicator scoring system for resource use in intrapartum care, inspired by (10) 61
S1-18. Characteristics of experts who provided performance estimates for the goals on psychosocial care experience and physical strain for care providers 62
S1-19. Characteristics of real stakeholders who provided value functions and weights for the main MCDA 63
S2. MCDA inputs 64
S2-1. Options' goal performance predictions 64
S2-1a. Prediction matrix, scenario 1 64
S2-1b. Performance predictions with uncertainty, scenario 1 66
S2-1c. Prediction matrix, scenario 2 67
S2-1d. Performance predictions with uncertainty, scenario 2 69
S2-1e. Prediction matrix, scenario 3 70
S2-1f. Performance predictions with uncertainty, scenario 3 72
S2-1g. Prediction matrix, scenario 4 73
S2-1h. Performance predictions with uncertainty, scenario 4 75
S2-2. Stakeholder preferences 76
S2-2a. Preference matrix, all scenarios 76
S2-2b. Value functions of real stakeholders 78
S3. MCDA results 79
S3-1. Main model: Overall values of childbirth options with stakeholder value functions and γ=0.2 79
S3-1a. Overall values of childbirth options with stakeholder value functions and γ=0.2, scenario 1 80
S3-1b. Overall values of childbirth options with stakeholder value functions and γ=0.2, scenario 2 81
S3-1c. Overall values of childbirth options with stakeholder value functions and γ=0.2, scenario 3 82
S3-1d. Overall values of childbirth options with stakeholder value functions and γ=0.2, scenario 4 83

S3-2. Cost-benefit visualizations 84
S3-2a. Cost-benefit visualizations, scenario 1 84
S3-2b. Cost-benefit visualizations, scenario 2 85
S3-2c. Cost-benefit visualizations, scenario 3 86
S3-2d. Cost-benefit visualizations, scenario 4 87
S3-3. Sensitivity analyses: Real stakeholder weights 88
S3-3a. Sensitivity analyses of real stakeholder weights, scenario 1 88
S3-3b. Sensitivity analyses of real stakeholder weights, scenario 2 89
S3-3c. Sensitivity analyses of real stakeholder weights, scenario 3 90
S3-3d. Sensitivity analyses of real stakeholder weights, scenario 4 91
S3-4. Sensitivity analyses: Overall values of childbirth options with value functions curvature c=2 and γ=0.2 92
S3-4a. Overall values of childbirth options with value functions curvature c=2 and γ=0.2, scenario 1 93
S3-4b. Overall values of childbirth options with value functions curvature c=2 and γ=0.2, scenario 2 94
S3-4c. Overall values of childbirth options with value functions curvature c=2 and γ=0.2, scenario 3 95
S3-4d. Overall values of childbirth options with value functions curvature c=2 and γ=0.2, scenario 4 96
S3-5. Sensitivity analyses: Overall values of childbirth options with linear value functions and γ=0.2 97
S3-5a. Overall values of childbirth options with linear value functions and γ=0.2, scenario 1 98
S3-5b. Overall values of childbirth options with linear value functions and γ=0.2, scenario 2 99
S3-5c. Overall values of childbirth options with linear value functions and γ=0.2, scenario 3 100
S3-5d. Overall values of childbirth options with linear value functions and γ=0.2, scenario 4 101
S3-6. Sensitivity analyses: Overall values of childbirth options with stakeholder value functions and γ=1 102
S3-6a. Overall values of childbirth options with stakeholder value functions and γ=1, scenario 1 103
S3-6b. Overall values of childbirth options with stakeholder value functions and γ=1, scenario 2 104
S3-6c. Overall values of childbirth options with stakeholder value functions and γ=1, scenario 3 105
S3-6d. Overall values of childbirth options with stakeholder value functions and γ=1, scenario 4 106
References 107

S1. Methods

S1-1. Study's inclusion and exclusion criteria

Inclusion criteria	Exclusion criteria
• Women with…<br>– preconceptional BMI ≥ 30kg/m^2<br>– singleton pregnancy with cephalic vertex presentation at term<br>– giving birth between 37+0 and 41+6 gestational weeks<br>• Childbirth in a Swiss hospital	• Known maternal respiratory, uro-/nephrological, neurological, coagulation disorder<br>• Known fetal anomalies or congenital malformations<br>• Fetal death before hospital admission<br>• Potentially compelling indication for cesarean birth before and/or during labor: Placenta previa, placental abruption, uterine rupture, umbilical cord prolapse, eclampsia, HELLP syndrome, amnion infection syndrome, HIV seroprevalence, HSV infection, diagnosed cephalopelvic disproportion, pathological CTG pattern (1-3)<br>• Extreme emergency cesarean delivery due to a life-threatening situation for the child and/or the mother

S1-2. Childbirth options

S1-2a. Childbirth options

	Standard hospital model of care	Midwifery continuity model of care
**Vaginal birth**	(a) *Vaginal birth with standard hospital care*: The woman intends and has a spontaneous or instrumental vaginal birth. During labor and childbirth, she primarily receives care from hospital midwives on duty. An obstetrician is usually present at childbirth. In most cases, the woman does not know the midwives and obstetrician(s) before admission to the hospital.	(b) *Vaginal birth with continuous midwifery care*: The woman intends and has a spontaneous or instrumental vaginal birth. During labor and childbirth, she receives one-to-one care from an attending midwife or a small group of midwives who have provided prenatal care. An obstetrician may or may not be present at childbirth. The woman knows the midwife/midwives before admission to the hospital but usually does not know the obstetrician(s).
**Intrapartum cesarean birth**	(c) *Intrapartum cesarean birth with standard hospital care*: The woman has a cesarean birth after the onset of active labor and/or rupture of the membranes without compelling indication (cf. S1-1). She primarily receives care from hospital midwives on duty. Obstetricians perform the cesarean. In most cases, the woman does not know the midwives and obstetrician(s) before admission to the hospital.	(d) *Intrapartum cesarean birth with continuous midwifery care*: The woman has a cesarean birth after the onset of active labor and/or rupture of the membranes without compelling indication (cf. S1-1). She receives one-to-one care from an attending midwife or a small group of midwives who have provided prenatal care. Obstetricians perform the cesarean. The woman knows the midwife/midwives before admission to the hospital but usually does not know the obstetrician(s).
**Prelabor cesarean birth**	(e) *Prelabor cesarean birth*: The woman has a cesarean birth before the onset of active labor and rupture of the membranes without compelling indication (cf. S1-1). During the cesarean, she usually receives care from a midwife and the surgical team. Obstetricians perform the cesarean. The woman usually does not know the care providers before admission to the hospital.	

S1-2b. Models of care

Swiss standard model of care	Midwifery continuity model of care
**Pregnancy**<br>• Prenatal care is primarily provided by obstetricians and gynecologists in private practice or in a hospital setting.<br><br>**Labor and childbirth**<br>• Labor and childbirth usually take place in public or private hospitals.<br>• Intrapartum care is primarily provided by hospital midwives, but it is obstetrician-led; meaning that midwives provide the actual care, but obstetricians have overall responsibility (4).<br>• An obstetrician is usually present at childbirth (4) but not necessarily during labor.<br>• Obstetricians on duty for labor and childbirth are usually not the same ones who provided prenatal care.<br>• Midwives and obstetricians attending labor and childbirth in hospitals are usually unknown to the women.<br><br>**Postpartum**<br>• Early postpartum care in hospitals is primarily provided by nurses and midwives, but it is obstetrician-led; meaning that nurses and midwives provide the actual care, but obstetricians have overall responsibility.<br>• After discharge from the hospital, postpartum care is usually provided in the family's home by an independent midwife until 56 days after childbirth.<br>• The midwife who provides postpartum care is usually not the same midwife who attended the childbirth or provided early postpartum care in the hospital.	**Pregnancy, labor and childbirth, and postpartum**<br>• Typically, a philosophy of normality of pregnancy and childbirth and a belief in a woman's natural ability to experience childbirth without the need for routine intervention (4, 5).<br>• Continuous care throughout the childbearing cycle by a known midwife or a small group of known midwives, including: Assessing women's and families' physical, psychological, and social well-being; Providing personalized information, education and prenatal care; Providing care and support during labor, childbirth and the immediate postpartum; Providing support during the postpartum and breastfeeding period; Minimizing avoidable interventions; Identifying the need for, referring women to, and coordinating specialty care when needed; Advocating for the women and navigating their care (4, 5).<br>• Midwives are the leading professionals in planning, organizing, and providing care for women from early pregnancy through the postpartum period (4).<br>• Prenatal, intrapartum, and/or postpartum care may be co-provided in collaboration with obstetricians or other medical professionals as needed (4, 6).
→ Differences between the models are often characterized by: Variations in childbirth philosophy and focus; the relationship between the woman (and her partner) and the maternity care provider; the setting of care; the utilization of obstetric interventions; and the goals of care (4, 7).	

S1-3. Sample characteristics of the Swiss hospital inpatient data, Swiss Obstetric Study Group (8)

Variable [m]	Statistic	Total sample (N=22,464)	Scenario 1 (n=13,700)	Scenario 2 (n=4,125)	Scenario 3 (n=3,379)	Scenario 4 (n=1,260)
**Scenarios**						
Comorbidities (diabetes mellitus and/or hypertensive disorder)[a] [0]	n (%)	5385 (23.97)	0 (0.00)	4125 (100.00)	0 (0.00)	1260 (100.00)
History of cesarean birth [0]	n (%)	4639 (20.65)	0 (0.00)	0 (0.00)	3379 (100.00)	1260 (100.00)
**Options**						
Vaginal birth [42]	n (%)	14637 (65.28)	10917 (79.86)	2899 (70.36)	659 (19.52)	162 (12.90)
Any cesarean birth [42]	n (%)	7785 (34.72)	2753 (20.14)	1221 (29.64)	2717 (80.48)	1094 (87.10)
Intrapartum cesarean birth [49]	n (%)	2776 (12.38)	1518 (11.10)	615 (14.94)	465 (13.79)	178 (14.17)
Prelabor cesarean birth [49]	n (%)	5002 (22.32)	1235 (9.03)	603 (14.65)	2248 (66.67)	916 (72.93)
Continuous midwifery care [23]	n (%)	403 (1.80)	254 (1.86)	72 (1.75)	56 (1.66)	21 (1.67)
**Maternal complication indicators**						
Postpartum hemorrhage [54]	n (%)	1060 (4.73)	720 (5.27)	235 (5.71)	73 (2.17)	32 (2.55)
Obstetric anal sphincter injury[b] (total sample) [2]	n (%)	449 (2.00)	319 (2.33)	99 (2.40)	26 (0.77)	5 (0.40)
Obstetric anal sphincter injury[b] (subgroup with vaginal birth) [2]	n (%)	446 (3.05)	316 (2.90)	99 (3.41)	26 (3.95)	5 (3.09)
Postpartum infection[c] [0]	n (%)	215 (0.96)	135 (0.99)	46 (1.12)	21 (0.62)	13 (1.03)
Obstetric wound complication[d] [0]	n (%)	131 (0.58)	59 (0.43)	30 (0.73)	32 (0.95)	10 (0.79)
Thromboembolic event[e] [0]	n (%)	9 (0.04)	6 (0.04)	1 (0.02)	2 (0.06)	0 (0.00)
**Neonatal complication indicators**						
Primary adaptation problem[f][0]	n (%)	1229 (5.47)	621 (4.53)	312 (7.56)	203 (6.01)	93 (7.38)
Advanced neonatal acidosis[g] (total sample) [1258]	n (%)	675 (3.18)	431 (3.35)	160 (4.12)	60 (1.85)	24 (1.99)
Advanced neonatal acidosis[g] (subsample with labor) [1022]	n (%)	607 (3.69)	417 (3.56)	148 (4.48)	32 (2.97)	10 (3.02)
Birth trauma[h] (total sample) [0]	n (%)	146 (0.65)	114 (0.83)	28 (0.68)	3 (0.09)	1 (0.08)
Birth trauma[h] (subgroup with vaginal birth) [0]	n (%)	142 (0.97)	111 (1.02)	28 (0.97)	3 (0.46)	0 (0.00)
Neonatal infection[i] [0]	n (%)	32 (0.14)	19 (0.14)	8 (0.19)	5 (0.15)	0 (0.00)
**Physiological childbirth indicators**						
Spontaneous onset of labor[j] [40]	n (%)	11148 (49.71)	8667 (63.37)	1432 (34.77)	851 (25.24)	198 (15.75)
Spontaneous progression of labor[k] [34]	n (%)	10984 (48.97)	7756 (56.68)	2185 (53.07)	789 (23.38)	254 (20.22)
Childbirth without anesthesia[l] [0]	n (%)	9122 (40.61)	6959 (50.80)	1707 (41.38)	368 (10.89)	88 (6.98)
No iatrogenic obstetric wound[m] [31]	n (%)	12114 (54.00)	8975 (65.60)	2465 (59.84)	536 (15.88)	138 (10.99)
Spontaneous birth [35]	n (%)	12902 (57.52)	9638 (70.50)	2551 (61.92)	573 (16.96)	140 (11.11)
**Breastfeeding initiation**						
Exclusive breastfeeding at hospital discharge [418]	n (%)	17938 (81.37)	11187 (83.60)	3171 (78.03)	2647 (79.01)	933 (74.64)
**Resource use in care setting**						
Clinical indicator score[n] [330]	mean (sd)	10.57 (4.12)	9.43 (3.48)	15.15 (3.55)	8.56 (2.83)	13.41 (2.63)
**Direct costs to healthcare system**						
SwissDRG cost weight [42]	mean (sd)	0.70 (0.18)	0.66 (0.17)	0.73 (0.22)	0.79 (0.14)	0.87 (0.15)
**Obstetric confounders**						
Maternal age (in years) [4]	mean (sd)	31.01 (5.07)	30.28 (5.04)	31.59 (5.13)	32.35 (4.60)	33.37 (4.65)
Primiparity [2]	n (%)	9288 (41.35)	6989 (51.02)	2299 (55.73)	0 (0.00)	0 (0.00)
White ethnicity [2]	n (%)	21192 (94.35)	13051 (95.27)	3870 (93.82)	3153 (93.34)	1118 (88.73)
Smoking status [0]	n (%)	2468 (10.99)	1472 (10.74)	507 (12.29)	349 (10.33)	140 (11.11)

Note: Sample characteristics of the Swiss hospital inpatient data from 22,464 childbirths between 2005 and 2022 that met the study's inclusion and exclusion criteria, Swiss Obstetric Study Group (8). [m]: number of missing values; n: number of observations; %: percentage; sd: standard deviation. [a] Comorbidities: Diabetes mellitus type I/II, gestational diabetes mellitus, chronic hypertension, pregnancy-induced hypertension, preeclampsia (excl. eclampsia). [b] Obstetric anal sphincter injury: 3rd/4th degree perineal tear. [c] Postpartum infection: Endometritis, urinary tract infection, maternal septicemia, maternal septic shock. [d] Obstetric wound complication: Obstetric wound hematoma, obstetric perineal wound dehiscence, postoperative wound dehiscence, postoperative wound infection. [e] Thromboembolic event: Deep venous thrombosis, pulmonary embolism. [f] Primary adaptation problem: Hypothermia (<35.5°C), hypoglycemia (<2mmol/l), respiratory distress syndrome, Apgar score at 5min. <7. [g] Advanced neonatal acidosis: Arterial umbilical pH <7.1. [h] Birth trauma: Paresis, clavicle fracture, other skeleton injury, cephalohematoma. [i] Neonatal infection: Postnatal infection, postnatal septicemia. [j] Spontaneous onset of labor: No induction of labor, no prelabor cesarean birth. [k] Spontaneous progression of labor: No augmentation of labor, no prelabor cesarean birth. [l] Childbirth without anesthesia: No epidural, spinal, and/or general anesthesia. [m] No iatrogenic obstetric wound: No episiotomy, no cesarean birth wound. [n] Intrapartum clinical indicator score: cf. S1-17.

S1-4. Characteristics of experts who provided value functions and weights for weighted composite indices

Characteristic [m]	Statistic	N = 6
Age in years [0]	range (mean; median)	34.00-55.00 (42.83; 41.00)
Self-reported female gender [0]	n (%)	6 (100.00)
Number of children [0]	range (mean; median)	0.00-5.00 (2.17; 2.00)
Self-reported obesity [0]	n (%)	1 (16.67)
Professional experience in years [0]	range (mean; median)	9.00-31.00 (19.33; 17.50)
Number of clients with obesity per year [1]	range (mean; median)	6.00-100.00 (34.20; 20.00)
Self-reported field of expertise[a]		General maternity care<br>Maternal obesity<br>Midwife-led maternity care<br>Neonatal care<br>Public health<br>Salutogenic maternity care

Note: [m]: number of missing values; n: number of observations; %: percentage. [a] Multiple answers possible.

S1-5. Inputs to the weighted composite index of maternal complication indicators

S1-5a. Prediction matrix for maternal complication indicators, scenario 1

Indicator	Unit	Option	Prediction	Minimum	Maximum	Uncertainty distribution	Expectation value (mean)	Standard deviation
Postpartum hemorrhage	%	a_VB_shc	5.897	0.5	12.5	normal	5.897	1.337
Postpartum hemorrhage	%	b_VB_cmc	5.948	0.5	12.5	normal	5.948	1.304
Postpartum hemorrhage	%	c_ICB_shc	3.176	0.5	12.5	normal	3.176	0.728
Postpartum hemorrhage	%	d_ICB_cmc	2.122	0.5	12.5	normal	2.122	0.486
Postpartum hemorrhage	%	e_PCB	1.889	0.5	12.5	normal	1.889	0.429
Obstetric anal sphincter injury	%	a_VB_shc	2.917	0	9.0	normal	2.917	1.699
Obstetric anal sphincter injury	%	b_VB_cmc	1.574	0	9.0	normal	1.574	0.953
Obstetric anal sphincter injury	%	c_ICB_shc	0	0	9.0	none	NA	NA
Obstetric anal sphincter injury	%	d_ICB_cmc	0	0	9.0	none	NA	NA
Obstetric anal sphincter injury	%	e_PCB	0	0	9.0	none	NA	NA
Postpartum infection	%	a_VB_shc	0.960	0.001	15.0	normal	0.960	0.751
Postpartum infection	%	b_VB_cmc	0.337	0.001	15.0	normal	0.337	0.223
Postpartum infection	%	c_ICB_shc	1.428	0.001	15.0	normal	1.428	1.054
Postpartum infection	%	d_ICB_cmc	3.458	0.001	15.0	normal	3.458	2.752
Postpartum infection	%	e_PCB	0.782	0.001	15.0	normal	0.782	0.633
Obstetric wound complication	%	a_VB_shc	0.333	0.001	5.0	normal	0.333	0.218
Obstetric wound complication	%	b_VB_cmc	0.608	0.001	5.0	normal	0.608	0.406
Obstetric wound complication	%	c_ICB_shc	0.847	0.001	5.0	normal	0.847	0.458
Obstetric wound complication	%	d_ICB_cmc	0.847*	0.001	5.0	normal	0.847*	0.458*
Obstetric wound complication	%	e_PCB	0.771	0.001	5.0	normal	0.771	0.492
Thromboembolic event	%	a_VB_shc	0.032	0.001	4.0	normal	0.032	0.070
Thromboembolic event	%	b_VB_cmc	0.032*	0.001	4.0	normal	0.032*	0.070*
Thromboembolic event	%	c_ICB_shc	0.095	0.001	4.0	normal	0.095	0.212
Thromboembolic event	%	d_ICB_cmc	0.095*	0.001	4.0	normal	0.095*	0.212*
Thromboembolic event	%	e_PCB	0.096	0.001	4.0	normal	0.096	0.205

Note: Adapted from the ValueDecisions' predictions template (9). Options: [a_VB_shc] Vaginal birth with standard hospital care; [b_VB_cmc] Vaginal birth with continuous midwifery care; [c_ICB_shc] Intrapartum cesarean birth with standard hospital care; [d_ICB_cmc] Intrapartum cesarean birth with continuous midwifery care; [e_PCB] Prelabor cesarean birth; NA: not applicable. * Approximate estimates based on women with the same childbirth mode in this scenario, irrespective of the model of care, as no events occurred with this option.

S1-5b. Prediction matrix for maternal complication indicators, scenario 2

Indicator	Unit	Option	Prediction	Minimum	Maximum	Uncertainty distribution	Expectation value (mean)	Standard deviation
Postpartum hemorrhage	%	a_VB_shc	6.877	0.5	12.5	normal	6.877	1.369
Postpartum hemorrhage	%	b_VB_cmc	7.082	0.5	12.5	normal	7.082	1.366
Postpartum hemorrhage	%	c_ICB_shc	3.605	0.5	12.5	normal	3.605	0.757
Postpartum hemorrhage	%	d_ICB_cmc	2.137	0.5	12.5	normal	2.137	0.499
Postpartum hemorrhage	%	e_PCB	2.146	0.5	12.5	normal	2.146	0.449
Obstetric anal sphincter injury	%	a_VB_shc	3.447	0	9.0	normal	3.447	2.078
Obstetric anal sphincter injury	%	b_VB_cmc	1.871	0	9.0	normal	1.871	1.130
Obstetric anal sphincter injury	%	c_ICB_shc	0	0	9.0	none	NA	NA
Obstetric anal sphincter injury	%	d_ICB_cmc	0	0	9.0	none	NA	NA
Obstetric anal sphincter injury	%	e_PCB	0	0	9.0	none	NA	NA
Postpartum infection	%	a_VB_shc	1.003	0.001	15.0	normal	1.003	0.831
Postpartum infection	%	b_VB_cmc	0.367	0.001	15.0	normal	0.367	0.310
Postpartum infection	%	c_ICB_shc	1.637	0.001	15.0	normal	1.637	1.221
Postpartum infection	%	d_ICB_cmc	5.843	0.001	15.0	normal	5.843	3.464
Postpartum infection	%	e_PCB	0.956	0.001	15.0	normal	0.956	0.756
Obstetric wound complication	%	a_VB_shc	0.482	0.001	5.0	normal	0.482	0.335
Obstetric wound complication	%	b_VB_cmc	0.862	0.001	5.0	normal	0.862	0.587
Obstetric wound complication	%	c_ICB_shc	1.327	0.001	5.0	normal	1.327	0.746
Obstetric wound complication	%	d_ICB_cmc	1.327*	0.001	5.0	normal	1.327*	0.746*
Obstetric wound complication	%	e_PCB	1.307	0.001	5.0	normal	1.307	0.804
Thromboembolic event	%	a_VB_shc	0.016	0.001	4.0	normal	0.016	0.037
Thromboembolic event	%	b_VB_cmc	0.016*	0.001	4.0	normal	0.016*	0.037*
Thromboembolic event	%	c_ICB_shc	0.041	0.001	4.0	normal	0.041	0.081
Thromboembolic event	%	d_ICB_cmc	0.041*	0.001	4.0	normal	0.041*	0.081*
Thromboembolic event	%	e_PCB	0.050	0.001	4.0	normal	0.050	0.100

Note: Adapted from the ValueDecisions' predictions template (9). Options: [a_VB_shc] Vaginal birth with standard hospital care; [b_VB_cmc] Vaginal birth with continuous midwifery care; [c_ICB_shc] Intrapartum cesarean birth with standard hospital care; [d_ICB_cmc] Intrapartum cesarean birth with continuous midwifery care; [e_PCB] Prelabor cesarean birth; NA: not applicable. * Approximate estimates based on women with the same childbirth mode in this scenario, irrespective of the model of care, as no events occurred with this option.

S1-5c. Prediction matrix for maternal complication indicators, scenario 3

Indicator	Unit	Option	Prediction	Minimum	Maximum	Uncertainty distribution	Expectation value (mean)	Standard deviation
Postpartum hemorrhage	%	a_VB_shc	4.562	0.5	12.5	normal	4.562	0.977
Postpartum hemorrhage	%	b_VB_cmc	4.868	0.5	12.5	normal	4.868	0.949
Postpartum hemorrhage	%	c_ICB_shc	2.407	0.5	12.5	normal	2.407	0.533
Postpartum hemorrhage	%	d_ICB_cmc	1.484	0.5	12.5	normal	1.484	0.322
Postpartum hemorrhage	%	e_PCB	1.422	0.5	12.5	normal	1.422	0.314
Obstetric anal sphincter injury	%	a_VB_shc	3.991	0	9.0	normal	3.991	1.935
Obstetric anal sphincter injury	%	b_VB_cmc	2.310	0	9.0	normal	2.310	0.998
Obstetric anal sphincter injury	%	c_ICB_shc	0	0	9.0	none	NA	NA
Obstetric anal sphincter injury	%	d_ICB_cmc	0	0	9.0	none	NA	NA
Obstetric anal sphincter injury	%	e_PCB	0	0	9.0	none	NA	NA
Postpartum infection	%	a_VB_shc	0.667	0.001	15.0	normal	0.667	0.483
Postpartum infection	%	b_VB_cmc	0.223	0.001	15.0	normal	0.223	0.165
Postpartum infection	%	c_ICB_shc	0.923	0.001	15.0	normal	0.923	0.680
Postpartum infection	%	d_ICB_cmc	3.021	0.001	15.0	normal	3.021	1.491
Postpartum infection	%	e_PCB	0.541	0.001	15.0	normal	0.541	0.402
Obstetric wound complication	%	a_VB_shc	0.500	0.001	5.0	normal	0.500	0.313
Obstetric wound complication	%	b_VB_cmc	0.853	0.001	5.0	normal	0.853	0.510
Obstetric wound complication	%	c_ICB_shc	1.068	0.001	5.0	normal	1.068	0.552
Obstetric wound complication	%	d_ICB_cmc	1.068*	0.001	5.0	normal	1.068*	0.552*
Obstetric wound complication	%	e_PCB	1.059	0.001	5.0	normal	1.059	0.627
Thromboembolic event	%	a_VB_shc	0.023	0.001	4.0	normal	0.023	0.048
Thromboembolic event	%	b_VB_cmc	0.023*	0.001	4.0	normal	0.023*	0.048*
Thromboembolic event	%	c_ICB_shc	0.075	0.001	4.0	normal	0.075	0.143
Thromboembolic event	%	d_ICB_cmc	0.075*	0.001	4.0	normal	0.075*	0.143*
Thromboembolic event	%	e_PCB	0.067	0.001	4.0	normal	0.067	0.142

Note: Adapted from the ValueDecisions' predictions template (9). Options: [a_VB_shc] Vaginal birth with standard hospital care; [b_VB_cmc] Vaginal birth with continuous midwifery care; [c_ICB_shc] Intrapartum cesarean birth with standard hospital care; [d_ICB_cmc] Intrapartum cesarean birth with continuous midwifery care; [e_PCB] Prelabor cesarean birth; NA: not applicable. * Approximate estimates based on women with the same childbirth mode in this scenario, irrespective of the model of care, as no events occurred with this option

S1-5d. Prediction matrix for maternal complication indicators, scenario 4

Indicator	Unit	Option	Prediction	Minimum	Maximum	Uncertainty distribution	Expectation value (mean)	Standard deviation
Postpartum hemorrhage	%	a_VB_shc	5.956	0.5	12.5	normal	5.956	1.145
Postpartum hemorrhage	%	b_VB_cmc	5.617	0.5	12.5	normal	5.617	0.597
Postpartum hemorrhage	%	c_ICB_shc	3.050	0.5	12.5	normal	3.050	0.608
Postpartum hemorrhage	%	d_ICB_cmc	1.890	0.5	12.5	normal	1.890	0.258
Postpartum hemorrhage	%	e_PCB	1.858	0.5	12.5	normal	1.858	0.369
Obstetric anal sphincter injury	%	a_VB_shc	3.092	0	9.0	normal	3.092	1.772
Obstetric anal sphincter injury	%	b_VB_cmc	2.853	0	9.0	normal	2.853	0.345
Obstetric anal sphincter injury	%	c_ICB_shc	0	0	9.0	none	NA	NA
Obstetric anal sphincter injury	%	d_ICB_cmc	0	0	9.0	none	NA	NA
Obstetric anal sphincter injury	%	e_PCB	0	0	9.0	none	NA	NA
Postpartum infection	%	a_VB_shc	1.048	0.001	15.0	normal	1.048	0.763
Postpartum infection	%	b_VB_cmc	0.529	0.001	15.0	normal	0.529	0.161
Postpartum infection	%	c_ICB_shc	1.572	0.001	15.0	normal	1.572	1.059
Postpartum infection	%	d_ICB_cmc	3.430	0.001	15.0	normal	3.430	1.018
Postpartum infection	%	e_PCB	0.918	0.001	15.0	normal	0.918	0.740
Obstetric wound complication	%	a_VB_shc	0.361	0.001	5.0	normal	0.361	0.250
Obstetric wound complication	%	b_VB_cmc	1.146	0.001	5.0	normal	1.146	0.141
Obstetric wound complication	%	c_ICB_shc	0.913	0.001	5.0	normal	0.913	0.527
Obstetric wound complication	%	d_ICB_cmc	0.913[a]	0.001	5.0	normal	0.913[a]	0.527[a]
Obstetric wound complication	%	e_PCB	0.853	0.001	5.0	normal	0.853	0.519
Thromboembolic event	%	a_VB_shc	0.016[b]	0.001	4.0	normal	0.016[b]	0.037[b]
Thromboembolic event	%	b_VB_cmc	0.016[c]	0.001	4.0	normal	0.016[c]	0.037[c]
Thromboembolic event	%	c_ICB_shc	0.041[b]	0.001	4.0	normal	0.041[b]	0.081[b]
Thromboembolic event	%	d_ICB_cmc	0.041[c]	0.001	4.0	normal	0.041[c]	0.081[c]
Thromboembolic event	%	e_PCB	0.050[b]	0.001	4.0	normal	0.050[b]	0.100[b]

Note: Adapted from the ValueDecisions' predictions template (9). Options: [a_VB_shc] Vaginal birth with standard hospital care; [b_VB_cmc] Vaginal birth with continuous midwifery care; [c_ICB_shc] Intrapartum cesarean birth with standard hospital care; [d_ICB_cmc] Intrapartum cesarean birth with continuous midwifery care; [e_PCB] Prelabor cesarean birth; NA: not applicable. [a] Approximate estimates based on women with the same childbirth mode in this scenario, irrespective of the model of care, as no events occurred with this option. [b] Approximate estimates based on women with comorbidities, irrespective of cesarean birth history, as no events occurred with these options in scenario 4. [c] Approximate estimates for women with comorbidities and the same childbirth mode, irrespective of cesarean birth history and the model of care, as no events occurred with these options in scenario 4.

S1-5e. Preference matrix for maternal complication indicators, all scenarios

Expert	Indicator	Unit	Worst	Best	Shape of value function	Indicator levels	Values of indicator levels	Global weight of indicator
E1	Postpartum hemorrhage	%	12.5	0.5	interpolate	12.50/10.40/8.00/5.00/0.50	0/0.25/0.5/0.75/1	0.210
E1	Obstetric anal sphincter injury	%	9.0	0	interpolate	9.00/7.50/5.50/3.15/0.00	0/0.25/0.5/0.75/1	0.247
E1	Postpartum infection	%	15.0	0.001	interpolate	15.00/12.30/9.00/5.50/0.001	0/0.25/0.5/0.75/1	0.136
E1	Obstetric wound complication	%	5.0	0.001	interpolate	5.00/4.10/3.00/1.75/0.001	0/0.25/0.5/0.75/1	0.235
E1	Thromboembolic event	%	4.0	0.001	interpolate	4.00/3.30/2.50/1.50/0.001	0/0.25/0.5/0.75/1	0.173
E2	Postpartum hemorrhage	%	12.5	0.5	interpolate	12.50/10.00/7.00/4.00/0.50	0/0.25/0.5/0.75/1	0.345
E2	Obstetric anal sphincter injury	%	9.0	0	interpolate	9.00/7.00/5.00/3.00/0.00	0/0.25/0.5/0.75/1	0.241
E2	Postpartum infection	%	15.0	0.001	interpolate	15.00/13.00/10.00/6.00/0.001	0/0.25/0.5/0.75/1	0.155
E2	Obstetric wound complication	%	5.0	0.001	interpolate	5.00/4.50/4.00/2.00/0.001	0/0.25/0.5/0.75/1	0.121
E2	Thromboembolic event	%	4.0	0.001	interpolate	4.00/3.00/2.00/1.00/0.001	0/0.25/0.5/0.75/1	0.138
E3	Postpartum hemorrhage	%	12.5	0.5	interpolate	12.50/10.50/8.00/5.00/0.50	0/0.25/0.5/0.75/1	0.244
E3	Obstetric anal sphincter injury	%	9.0	0	interpolate	9.00/7.50/5.50/3.40/0.00	0/0.25/0.5/0.75/1	0.146
E3	Postpartum infection	%	15.0	0.001	interpolate	15.00/13.00/10.00/6.00/0.001	0/0.25/0.5/0.75/1	0.220
E3	Obstetric wound complication	%	5.0	0.001	interpolate	5.00/4.25/3.00/1.50/0.001	0/0.25/0.5/0.75/1	0.171
E3	Thromboembolic event	%	4.0	0.001	interpolate	4.00/3.50/2.50/1.50/0.001	0/0.25/0.5/0.75/1	0.220
E_pooled	Postpartum hemorrhage	%	12.5	0.5	interpolate	12.50/10.30/7.67/4.67/0.50	0/0.25/0.5/0.75/1	0.266
E_pooled	Obstetric anal sphincter injury	%	9.0	0	interpolate	9.00/7.33/5.33/3.18/0.00	0/0.25/0.5/0.75/1	0.211
E_pooled	Postpartum infection	%	15.0	0.001	interpolate	15.00/12.77/9.67/5.83/0.001	0/0.25/0.5/0.75/1	0.170
E_pooled	Obstetric wound complication	%	5.0	0.001	interpolate	5.00/4.28/3.33/1.75/0.001	0/0.25/0.5/0.75/1	0.176
E_pooled	Thromboembolic event	%	4.0	0.001	interpolate	4.00/3.27/2.33/1.33/0.001	0/0.25/0.5/0.75/1	0.177

Note: Adapted from the ValueDecisions' preferences template (9).

S1-6. Main model: Outputs of the weighted composite index of maternal complication indicators with expert value functions and γ=0.2

S1-6a. Overall values of options for maternal complication indicators with expert value functions and γ=0.2, scenario 1

Expert	Option	Mean overall value	5% quantile of overall value	95% quantile of overall value	Minimum	Maximum
E1	a_VB_shc	0.833	0.736	0.908	0.561	0.95
E1	b_VB_cmc	0.86	0.801	0.912	0.711	0.954
E1	c_ICB_shc	0.921	0.889	0.95	0.838	0.979
E1	d_ICB_cmc	0.917	0.868	0.954	0.754	0.991
E1	e_PCB	0.943	0.913	0.97	0.868	0.994
E2	a_VB_shc	0.757	0.634	0.857	0.469	0.928
E2	b_VB_cmc	0.789	0.701	0.869	0.6	0.943
E2	c_ICB_shc	0.9	0.862	0.935	0.818	0.962
E2	d_ICB_cmc	0.911	0.866	0.946	0.755	0.99
E2	e_PCB	0.939	0.915	0.963	0.893	0.995
E3	a_VB_shc	0.849	0.768	0.91	0.658	0.945
E3	b_VB_cmc	0.864	0.803	0.917	0.714	0.954
E3	c_ICB_shc	0.914	0.882	0.944	0.842	0.976
E3	d_ICB_cmc	0.905	0.847	0.949	0.699	0.989
E3	e_PCB	0.94	0.912	0.965	0.875	0.993
E_pooled	a_VB_shc	0.816	0.716	0.894	0.561	0.943
E_pooled	b_VB_cmc	0.841	0.772	0.903	0.68	0.951
E_pooled	c_ICB_shc	0.914	0.884	0.944	0.84	0.973
E_pooled	d_ICB_cmc	0.912	0.862	0.951	0.737	0.99
E_pooled	e_PCB	0.942	0.917	0.966	0.884	0.994

Note: Overall values for each option and each expert using elicited expert value functions and a non-additive aggregation model with γ=0.2 for scenario 1: 0 (highest/"worst" complication rates) to 1 (lowest/"best" complication rates). Uncertainty in the predictions was accounted for by 2,000 Monte Carlo simulations. Options: [a_VB_shc] Vaginal birth with standard hospital care; [b_VB_cmc] Vaginal birth with continuous midwifery care; [c_ICB_shc] Intrapartum cesarean birth with standard hospital care; [d_ICB_cmc] Intrapartum cesarean birth with continuous midwifery care; [e_PCB] Prelabor cesarean birth.

S1-6b. Overall values of options for maternal complication indicators with expert value functions and γ=0.2, scenario 2

Expert	Option	Mean overall value	5% quantile of overall value	95% quantile of overall value	Minimum	Maximum
E1	a_VB_shc	0.787	0.655	0.883	0.361	0.958
E1	b_VB_cmc	0.82	0.746	0.889	0.615	0.938
E1	c_ICB_shc	0.896	0.838	0.941	0.709	0.973
E1	d_ICB_cmc	0.874	0.783	0.942	0.553	0.984
E1	e_PCB	0.918	0.853	0.963	0.74	0.99
E2	a_VB_shc	0.698	0.551	0.814	0.317	0.924
E2	b_VB_cmc	0.737	0.638	0.83	0.46	0.921
E2	c_ICB_shc	0.882	0.839	0.922	0.802	0.965
E2	d_ICB_cmc	0.883	0.807	0.937	0.525	0.977
E2	e_PCB	0.926	0.896	0.954	0.855	0.993
E3	a_VB_shc	0.808	0.709	0.884	0.517	0.953
E3	b_VB_cmc	0.824	0.746	0.891	0.584	0.938
E3	c_ICB_shc	0.893	0.848	0.935	0.765	0.969
E3	d_ICB_cmc	0.86	0.756	0.936	0.408	0.98
E3	e_PCB	0.92	0.873	0.959	0.797	0.991
E_pooled	a_VB_shc	0.767	0.64	0.863	0.394	0.947
E_pooled	b_VB_cmc	0.797	0.713	0.873	0.552	0.935
E_pooled	c_ICB_shc	0.894	0.851	0.933	0.764	0.969
E_pooled	d_ICB_cmc	0.874	0.784	0.939	0.493	0.981
E_pooled	e_PCB	0.924	0.88	0.959	0.805	0.992

Note: Overall values for each option and each expert using elicited expert value functions and a non-additive aggregation model with γ=0.2 for scenario 2: 0 (highest/"worst" complication rates) to 1 (lowest/"best" complication rates). Uncertainty in the predictions was accounted for by 2,000 Monte Carlo simulations. Options: [a_VB_shc] Vaginal birth with standard hospital care; [b_VB_cmc] Vaginal birth with continuous midwifery care; [c_ICB_shc] Intrapartum cesarean birth with standard hospital care; [d_ICB_cmc] Intrapartum cesarean birth with continuous midwifery care; [e_PCB] Prelabor cesarean birth.

S1-6c. Overall values of options for maternal complication indicators with expert value functions and γ=0.2, scenario 3

Expert	Option	Mean overall value	5% quantile of overall value	95% quantile of overall value	Minimum	Maximum
E1	a_VB_shc	0.823	0.698	0.908	0.411	0.955
E1	b_VB_cmc	0.863	0.81	0.912	0.748	0.95
E1	c_ICB_shc	0.926	0.887	0.958	0.834	0.981
E1	d_ICB_cmc	0.925	0.885	0.959	0.819	0.983
E1	e_PCB	0.94	0.895	0.975	0.825	0.992
E2	a_VB_shc	0.767	0.63	0.871	0.39	0.939
E2	b_VB_cmc	0.813	0.747	0.875	0.68	0.929
E2	c_ICB_shc	0.922	0.893	0.949	0.865	0.974
E2	d_ICB_cmc	0.932	0.906	0.957	0.864	0.979
E2	e_PCB	0.95	0.928	0.971	0.9	0.989
E3	a_VB_shc	0.856	0.775	0.915	0.578	0.959
E3	b_VB_cmc	0.877	0.832	0.918	0.784	0.947
E3	c_ICB_shc	0.924	0.891	0.955	0.856	0.976
E3	d_ICB_cmc	0.918	0.881	0.953	0.829	0.98
E3	e_PCB	0.942	0.906	0.972	0.863	0.99
E_pooled	a_VB_shc	0.819	0.706	0.9	0.457	0.953
E_pooled	b_VB_cmc	0.855	0.802	0.903	0.746	0.941
E_pooled	c_ICB_shc	0.926	0.896	0.954	0.864	0.977
E_pooled	d_ICB_cmc	0.926	0.894	0.957	0.849	0.98
E_pooled	e_PCB	0.945	0.913	0.972	0.875	0.99

Note: Overall values for each option and each expert using elicited expert value functions and a non-additive aggregation model with γ=0.2 for scenario 3: 0 (highest/"worst" complication rates) to 1 (lowest/"best" complication rates). Uncertainty in the predictions was accounted for by 2,000 Monte Carlo simulations. Options: [a_VB_shc] Vaginal birth with standard hospital care; [b_VB_cmc] Vaginal birth with continuous midwifery care; [c_ICB_shc] Intrapartum cesarean birth with standard hospital care; [d_ICB_cmc] Intrapartum cesarean birth with continuous midwifery care; [e_PCB] Prelabor cesarean birth.

S1-6d. Overall values of options for maternal complication indicators with expert value functions and γ=0.2, scenario 4

Expert	Option	Mean overall value	5% quantile of overall value	95% quantile of overall value	Minimum	Maximum
E1	a_VB_shc	0.827	0.727	0.901	0.483	0.955
E1	b_VB_cmc	0.832	0.806	0.855	0.774	0.871
E1	c_ICB_shc	0.922	0.889	0.952	0.824	0.976
E1	d_ICB_cmc	0.925	0.893	0.954	0.835	0.974
E1	e_PCB	0.943	0.911	0.971	0.844	0.986
E2	a_VB_shc	0.749	0.632	0.846	0.419	0.922
E2	b_VB_cmc	0.776	0.738	0.812	0.69	0.845
E2	c_ICB_shc	0.905	0.874	0.937	0.832	0.958
E2	d_ICB_cmc	0.924	0.903	0.944	0.88	0.963
E2	e_PCB	0.941	0.919	0.963	0.892	0.983
E3	a_VB_shc	0.844	0.771	0.904	0.609	0.948
E3	b_VB_cmc	0.85	0.824	0.873	0.789	0.888
E3	c_ICB_shc	0.916	0.884	0.946	0.845	0.972
E3	d_ICB_cmc	0.917	0.885	0.945	0.843	0.966
E3	e_PCB	0.941	0.911	0.967	0.867	0.983
E_pooled	a_VB_shc	0.81	0.713	0.887	0.502	0.941
E_pooled	b_VB_cmc	0.823	0.794	0.851	0.756	0.871
E_pooled	c_ICB_shc	0.917	0.888	0.944	0.854	0.969
E_pooled	d_ICB_cmc	0.924	0.896	0.948	0.858	0.967
E_pooled	e_PCB	0.943	0.917	0.967	0.877	0.982

Note: Overall values for each option and each expert using elicited expert value functions and a non-additive aggregation model with γ=0.2 for scenario 4: 0 (highest/"worst" complication rates) to 1 (lowest/"best" complication rates). Uncertainty in the predictions was accounted for by 2,000 Monte Carlo simulations. Options: [a_VB_shc] Vaginal birth with standard hospital care; [b_VB_cmc] Vaginal birth with continuous midwifery care; [c_ICB_shc] Intrapartum cesarean birth with standard hospital care; [d_ICB_cmc] Intrapartum cesarean birth with continuous midwifery care; [e_PCB] Prelabor cesarean birth.

S1-7. Sensitivity analyses: Outputs of the weighted composite index of maternal complication indicators with linear value functions and γ=0.2

S1-7a. Overall values of options for maternal complication indicators with linear value functions and γ=0.2, scenario 1

Expert	Option	Mean overall value	5% quantile of overall value	95% quantile of overall value	Minimum	Maximum
E1	a_VB_shc	0.766	0.656	0.863	0.418	0.936
E1	b_VB_cmc	0.805	0.733	0.871	0.624	0.936
E1	c_ICB_shc	0.884	0.838	0.928	0.792	0.96
E1	d_ICB_cmc	0.879	0.818	0.932	0.729	0.974
E1	e_PCB	0.917	0.874	0.956	0.813	0.987
E2	a_VB_shc	0.712	0.598	0.825	0.386	0.913
E2	b_VB_cmc	0.756	0.662	0.84	0.533	0.93
E2	c_ICB_shc	0.873	0.826	0.918	0.787	0.954
E2	d_ICB_cmc	0.878	0.816	0.929	0.745	0.965
E2	e_PCB	0.921	0.889	0.952	0.846	0.987
E3	a_VB_shc	0.783	0.699	0.864	0.544	0.927
E3	b_VB_cmc	0.811	0.74	0.876	0.626	0.941
E3	c_ICB_shc	0.876	0.831	0.92	0.785	0.954
E3	d_ICB_cmc	0.86	0.779	0.924	0.686	0.971
E3	e_PCB	0.917	0.88	0.952	0.832	0.983
E_pooled	a_VB_shc	0.753	0.652	0.85	0.446	0.925
E_pooled	b_VB_cmc	0.791	0.713	0.861	0.601	0.935
E_pooled	c_ICB_shc	0.878	0.833	0.921	0.789	0.954
E_pooled	d_ICB_cmc	0.872	0.806	0.927	0.72	0.969
E_pooled	e_PCB	0.918	0.882	0.952	0.835	0.986

Note: Overall values for each option and each expert using linear value functions and a non-additive aggregation model with γ=0.2 for scenario 1: 0 (highest/"worst" complication rates) to 1 (lowest/"best" complication rates). Uncertainty in the predictions was accounted for by 2,000 Monte Carlo simulations. Options: [a_VB_shc] Vaginal birth with standard hospital care; [b_VB_cmc] Vaginal birth with continuous midwifery care; [c_ICB_shc] Intrapartum cesarean birth with standard hospital care; [d_ICB_cmc] Intrapartum cesarean birth with continuous midwifery care; [e_PCB] Prelabor cesarean birth.

S1-7b. Overall values of options for maternal complication indicators with linear value functions and γ=0.2, scenario 2

Expert	Option	Mean overall value	5% quantile of overall value	95% quantile of overall value	Minimum	Maximum
E1	a_VB_shc	0.717	0.571	0.83	0.321	0.936
E1	b_VB_cmc	0.755	0.668	0.838	0.563	0.909
E1	c_ICB_shc	0.85	0.777	0.912	0.643	0.959
E1	d_ICB_cmc	0.828	0.724	0.916	0.516	0.976
E1	e_PCB	0.883	0.804	0.946	0.688	0.986
E2	a_VB_shc	0.657	0.509	0.779	0.298	0.907
E2	b_VB_cmc	0.695	0.592	0.796	0.428	0.9
E2	c_ICB_shc	0.847	0.792	0.899	0.72	0.954
E2	d_ICB_cmc	0.835	0.738	0.914	0.489	0.97
E2	e_PCB	0.899	0.851	0.94	0.777	0.991
E3	a_VB_shc	0.74	0.632	0.829	0.47	0.927
E3	b_VB_cmc	0.762	0.679	0.841	0.534	0.913
E3	c_ICB_shc	0.849	0.789	0.904	0.689	0.954
E3	d_ICB_cmc	0.803	0.671	0.907	0.372	0.97
E3	e_PCB	0.891	0.83	0.941	0.733	0.988
E_pooled	a_VB_shc	0.704	0.571	0.811	0.358	0.924
E_pooled	b_VB_cmc	0.737	0.646	0.825	0.506	0.907
E_pooled	c_ICB_shc	0.848	0.787	0.904	0.683	0.956
E_pooled	d_ICB_cmc	0.822	0.712	0.912	0.456	0.972
E_pooled	e_PCB	0.891	0.828	0.941	0.733	0.988

Note: Overall values for each option and each expert using linear value functions and a non-additive aggregation model with γ=0.2 for scenario 2: 0 (highest/"worst" complication rates) to 1 (lowest/"best" complication rates). Uncertainty in the predictions was accounted for by 2,000 Monte Carlo simulations. Options: [a_VB_shc] Vaginal birth with standard hospital care; [b_VB_cmc] Vaginal birth with continuous midwifery care; [c_ICB_shc] Intrapartum cesarean birth with standard hospital care; [d_ICB_cmc] Intrapartum cesarean birth with continuous midwifery care; [e_PCB] Prelabor cesarean birth.

S1-7c. Overall values of options for maternal complication indicators with linear value functions and γ=0.2, scenario 3

Expert	Option	Mean overall value	5% quantile of overall value	95% quantile of overall value	Minimum	Maximum
E1	a_VB_shc	0.759	0.619	0.864	0.371	0.935
E1	b_VB_cmc	0.802	0.733	0.869	0.661	0.925
E1	c_ICB_shc	0.892	0.839	0.938	0.781	0.972
E1	d_ICB_cmc	0.891	0.837	0.941	0.768	0.974
E1	e_PCB	0.914	0.855	0.963	0.779	0.988
E2	a_VB_shc	0.728	0.596	0.838	0.371	0.925
E2	b_VB_cmc	0.773	0.701	0.844	0.636	0.906
E2	c_ICB_shc	0.898	0.859	0.934	0.822	0.965
E2	d_ICB_cmc	0.903	0.863	0.941	0.804	0.973
E2	e_PCB	0.931	0.897	0.962	0.851	0.986
E3	a_VB_shc	0.799	0.708	0.874	0.538	0.941
E3	b_VB_cmc	0.824	0.767	0.878	0.711	0.922
E3	c_ICB_shc	0.895	0.852	0.934	0.806	0.966
E3	d_ICB_cmc	0.884	0.831	0.934	0.758	0.972
E3	e_PCB	0.922	0.876	0.96	0.819	0.984
E_pooled	a_VB_shc	0.762	0.641	0.859	0.422	0.933
E_pooled	b_VB_cmc	0.799	0.734	0.863	0.669	0.916
E_pooled	c_ICB_shc	0.895	0.851	0.935	0.805	0.966
E_pooled	d_ICB_cmc	0.892	0.844	0.937	0.785	0.971
E_pooled	e_PCB	0.922	0.876	0.961	0.82	0.986

Note: Overall values for each option and each expert using linear value functions and a non-additive aggregation model with γ=0.2 for scenario 3: 0 (highest/"worst" complication rates) to 1 (lowest/"best" complication rates). Uncertainty in the predictions was accounted for by 2,000 Monte Carlo simulations. Options: [a_VB_shc] Vaginal birth with standard hospital care; [b_VB_cmc] Vaginal birth with continuous midwifery care; [c_ICB_shc] Intrapartum cesarean birth with standard hospital care; [d_ICB_cmc] Intrapartum cesarean birth with continuous midwifery care; [e_PCB] Prelabor cesarean birth.

S1-7d. Overall values of options for maternal complication indicators with linear value functions and γ=0.2, scenario 4

Expert	Option	Mean overall value	5% quantile of overall value	95% quantile of overall value	Minimum	Maximum
E1	a_VB_shc	0.761	0.647	0.854	0.423	0.931
E1	b_VB_cmc	0.759	0.728	0.789	0.693	0.813
E1	c_ICB_shc	0.886	0.838	0.929	0.767	0.964
E1	d_ICB_cmc	0.891	0.845	0.932	0.776	0.961
E1	e_PCB	0.917	0.871	0.957	0.795	0.979
E2	a_VB_shc	0.709	0.592	0.812	0.393	0.898
E2	b_VB_cmc	0.728	0.689	0.766	0.639	0.801
E2	c_ICB_shc	0.877	0.837	0.918	0.788	0.948
E2	d_ICB_cmc	0.894	0.861	0.925	0.819	0.951
E2	e_PCB	0.922	0.891	0.952	0.853	0.976
E3	a_VB_shc	0.781	0.698	0.855	0.549	0.921
E3	b_VB_cmc	0.786	0.756	0.815	0.717	0.838
E3	c_ICB_shc	0.88	0.836	0.922	0.79	0.957
E3	d_ICB_cmc	0.879	0.836	0.919	0.78	0.95
E3	e_PCB	0.918	0.88	0.954	0.823	0.975
E_pooled	a_VB_shc	0.75	0.645	0.839	0.452	0.917
E_pooled	b_VB_cmc	0.757	0.724	0.789	0.685	0.817
E_pooled	c_ICB_shc	0.881	0.838	0.921	0.79	0.956
E_pooled	d_ICB_cmc	0.888	0.847	0.924	0.791	0.953
E_pooled	e_PCB	0.919	0.881	0.953	0.823	0.975

Note: Overall values for each option and each expert using linear value functions and a non-additive aggregation model with γ=0.2 for scenario 4: 0 (highest/"worst" complication rates) to 1 (lowest/"best" complication rates). Uncertainty in the predictions was accounted for by 2,000 Monte Carlo simulations. Options: [a_VB_shc] Vaginal birth with standard hospital care; [b_VB_cmc] Vaginal birth with continuous midwifery care; [c_ICB_shc] Intrapartum cesarean birth with standard hospital care; [d_ICB_cmc] Intrapartum cesarean birth with continuous midwifery care; [e_PCB] Prelabor cesarean birth.

S1-8. Sensitivity analyses: Outputs of the weighted composite index of maternal complication indicators with expert value functions and γ=1

S1-8a. Overall values of options for maternal complication indicators with expert value functions and γ=1, scenario 1

Expert	Option	Mean overall value	5% quantile of overall value	95% quantile of overall value	Minimum	Maximum
E1	a_VB_shc	0.847	0.771	0.912	0.601	0.95
E1	b_VB_cmc	0.872	0.821	0.918	0.765	0.96
E1	c_ICB_shc	0.923	0.892	0.951	0.846	0.971
E1	d_ICB_cmc	0.919	0.877	0.954	0.836	0.982
E1	e_PCB	0.945	0.918	0.969	0.874	0.987
E2	a_VB_shc	0.777	0.683	0.865	0.495	0.932
E2	b_VB_cmc	0.811	0.742	0.877	0.681	0.944
E2	c_ICB_shc	0.903	0.867	0.936	0.83	0.964
E2	d_ICB_cmc	0.913	0.872	0.946	0.823	0.977
E2	e_PCB	0.941	0.917	0.962	0.894	0.982
E3	a_VB_shc	0.861	0.803	0.914	0.674	0.948
E3	b_VB_cmc	0.877	0.828	0.92	0.779	0.964
E3	c_ICB_shc	0.916	0.884	0.944	0.846	0.968
E3	d_ICB_cmc	0.907	0.855	0.948	0.775	0.975
E3	e_PCB	0.942	0.916	0.965	0.88	0.985
E_pooled	a_VB_shc	0.831	0.756	0.899	0.591	0.944
E_pooled	b_VB_cmc	0.857	0.801	0.907	0.746	0.957
E_pooled	c_ICB_shc	0.916	0.886	0.944	0.851	0.967
E_pooled	d_ICB_cmc	0.914	0.87	0.95	0.813	0.978
E_pooled	e_PCB	0.944	0.92	0.965	0.888	0.985

Note: Overall values for each option and each expert using elicited expert value functions and an additive aggregation model with γ=1 for scenario 1: 0 (highest/"worst" complication rates) to 1 (lowest/"best" complication rates). Uncertainty in the predictions was accounted for by 2,000 Monte Carlo simulations. Options: [a_VB_shc] Vaginal birth with standard hospital care; [b_VB_cmc] Vaginal birth with continuous midwifery care; [c_ICB_shc] Intrapartum cesarean birth with standard hospital care; [d_ICB_cmc] Intrapartum cesarean birth with continuous midwifery care; [e_PCB] Prelabor cesarean birth.

S1-8b. Overall values of options for maternal complication indicators with expert value functions and γ=1, scenario 2

Expert	Option	Mean overall value	5% quantile of overall value	95% quantile of overall value	Minimum	Maximum
E1	a_VB_shc	0.808	0.712	0.889	0.611	0.959
E1	b_VB_cmc	0.836	0.775	0.894	0.698	0.939
E1	c_ICB_shc	0.901	0.851	0.943	0.771	0.973
E1	d_ICB_cmc	0.886	0.816	0.944	0.74	0.984
E1	e_PCB	0.922	0.868	0.964	0.797	0.991
E2	a_VB_shc	0.73	0.616	0.827	0.51	0.926
E2	b_VB_cmc	0.766	0.693	0.84	0.609	0.923
E2	c_ICB_shc	0.887	0.848	0.924	0.817	0.965
E2	d_ICB_cmc	0.891	0.834	0.938	0.762	0.977
E2	e_PCB	0.928	0.9	0.955	0.865	0.993
E3	a_VB_shc	0.828	0.754	0.89	0.684	0.954
E3	b_VB_cmc	0.842	0.785	0.897	0.722	0.94
E3	c_ICB_shc	0.898	0.859	0.936	0.809	0.969
E3	d_ICB_cmc	0.872	0.794	0.938	0.696	0.98
E3	e_PCB	0.924	0.885	0.959	0.83	0.991
E_pooled	a_VB_shc	0.792	0.696	0.871	0.603	0.948
E_pooled	b_VB_cmc	0.818	0.755	0.88	0.687	0.936
E_pooled	c_ICB_shc	0.899	0.861	0.935	0.804	0.969
E_pooled	d_ICB_cmc	0.885	0.818	0.94	0.74	0.981
E_pooled	e_PCB	0.927	0.89	0.959	0.833	0.992

Note: Overall values for each option and each expert using elicited expert value functions and an additive aggregation model with γ=1 for scenario 2: 0 (highest/"worst" complication rates) to 1 (lowest/"best" complication rates). Uncertainty in the predictions was accounted for by 2,000 Monte Carlo simulations. Options: [a_VB_shc] Vaginal birth with standard hospital care; [b_VB_cmc] Vaginal birth with continuous midwifery care; [c_ICB_shc] Intrapartum cesarean birth with standard hospital care; [d_ICB_cmc] Intrapartum cesarean birth with continuous midwifery care; [e_PCB] Prelabor cesarean birth.

S1-8c. Overall values of options for maternal complication indicators with expert value functions and γ=1, scenario 3

Expert	Option	Mean overall value	5% quantile of overall value	95% quantile of overall value	Minimum	Maximum
E1	a_VB_shc	0.84	0.75	0.911	0.672	0.956
E1	b_VB_cmc	0.868	0.818	0.915	0.761	0.951
E1	c_ICB_shc	0.928	0.893	0.959	0.852	0.981
E1	d_ICB_cmc	0.927	0.891	0.96	0.845	0.983
E1	e_PCB	0.943	0.904	0.975	0.854	0.992
E2	a_VB_shc	0.788	0.689	0.876	0.601	0.94
E2	b_VB_cmc	0.823	0.765	0.879	0.709	0.929
E2	c_ICB_shc	0.924	0.896	0.95	0.87	0.975
E2	d_ICB_cmc	0.934	0.909	0.957	0.872	0.979
E2	e_PCB	0.951	0.93	0.971	0.906	0.989
E3	a_VB_shc	0.869	0.81	0.918	0.749	0.959
E3	b_VB_cmc	0.884	0.843	0.92	0.799	0.949
E3	c_ICB_shc	0.927	0.897	0.955	0.87	0.976
E3	d_ICB_cmc	0.921	0.888	0.954	0.841	0.98
E3	e_PCB	0.944	0.914	0.972	0.879	0.99
E_pooled	a_VB_shc	0.836	0.753	0.903	0.677	0.953
E_pooled	b_VB_cmc	0.862	0.813	0.907	0.761	0.943
E_pooled	c_ICB_shc	0.928	0.9	0.955	0.874	0.977
E_pooled	d_ICB_cmc	0.929	0.899	0.957	0.86	0.98
E_pooled	e_PCB	0.947	0.919	0.973	0.886	0.99

Note: Overall values for each option and each expert using elicited expert value functions and an additive aggregation model with γ=1 for scenario 3: 0 (highest/"worst" complication rates) to 1 (lowest/"best" complication rates). Uncertainty in the predictions was accounted for by 2,000 Monte Carlo simulations. Options: [a_VB_shc] Vaginal birth with standard hospital care; [b_VB_cmc] Vaginal birth with continuous midwifery care; [c_ICB_shc] Intrapartum cesarean birth with standard hospital care; [d_ICB_cmc] Intrapartum cesarean birth with continuous midwifery care; [e_PCB] Prelabor cesarean birth.

S1-8d. Overall values of options for maternal complication indicators with expert value functions and γ=1, scenario 4

Expert	Option	Mean overall value	5% quantile of overall value	95% quantile of overall value	Minimum	Maximum
E1	a_VB_shc	0.84	0.76	0.905	0.649	0.956
E1	b_VB_cmc	0.838	0.815	0.859	0.786	0.873
E1	c_ICB_shc	0.925	0.893	0.953	0.843	0.977
E1	d_ICB_cmc	0.928	0.898	0.955	0.849	0.974
E1	e_PCB	0.944	0.915	0.971	0.862	0.986
E2	a_VB_shc	0.77	0.673	0.854	0.582	0.923
E2	b_VB_cmc	0.787	0.755	0.819	0.717	0.848
E2	c_ICB_shc	0.908	0.879	0.938	0.841	0.958
E2	d_ICB_cmc	0.926	0.906	0.945	0.884	0.963
E2	e_PCB	0.943	0.921	0.963	0.897	0.983
E3	a_VB_shc	0.857	0.8	0.908	0.731	0.95
E3	b_VB_cmc	0.857	0.835	0.877	0.809	0.891
E3	c_ICB_shc	0.919	0.888	0.947	0.86	0.972
E3	d_ICB_cmc	0.919	0.89	0.946	0.855	0.967
E3	e_PCB	0.943	0.915	0.968	0.882	0.983
E_pooled	a_VB_shc	0.826	0.746	0.891	0.657	0.944
E_pooled	b_VB_cmc	0.831	0.806	0.856	0.775	0.874
E_pooled	c_ICB_shc	0.919	0.892	0.946	0.86	0.969
E_pooled	d_ICB_cmc	0.926	0.9	0.949	0.867	0.968
E_pooled	e_PCB	0.945	0.92	0.968	0.888	0.982

Note: Overall values for each option and each expert using elicited expert value functions and an additive aggregation model with γ=1 for scenario 4: 0 (highest/"worst" complication rates) to 1 (lowest/"best" complication rates). Uncertainty in the predictions was accounted for by 2,000 Monte Carlo simulations. Options: [a_VB_shc] Vaginal birth with standard hospital care; [b_VB_cmc] Vaginal birth with continuous midwifery care; [c_ICB_shc] Intrapartum cesarean birth with standard hospital care; [d_ICB_cmc] Intrapartum cesarean birth with continuous midwifery care; [e_PCB] Prelabor cesarean birth.

S1-9. Inputs to the weighted composite index of neonatal complication indicators

S1-9a. Prediction matrix for neonatal complication indicators, scenario 1

Indicator	Unit	Option	Prediction	Minimum	Maximum	Uncertainty distribution	Expectation value (mean)	Standard deviation
Primary adaptation problem	%	a_VB_shc	4.265	0.001	17.0	normal	4.265	0.481
Primary adaptation problem	%	b_VB_cmc	0.600	0.001	17.0	normal	0.600	0.048
Primary adaptation problem	%	c_ICB_shc	5.859	0.001	17.0	normal	5.859	0.695
Primary adaptation problem	%	d_ICB_cmc	1.663	0.001	17.0	normal	1.663	0.08
Primary adaptation problem	%	e_PCB	5.866	0.001	17.0	normal	5.866	0.664
Advanced neonatal acidosis	%	a_VB_shc	3.664	0.001	14.0	normal	3.664	1.053
Advanced neonatal acidosis	%	b_VB_cmc	5.636	0.001	14.0	normal	5.636	1.575
Advanced neonatal acidosis	%	c_ICB_shc	2.287	0.001	14.0	normal	2.287	0.398
Advanced neonatal acidosis	%	d_ICB_cmc	1.850	0.001	14.0	normal	1.850	0.396
Advanced neonatal acidosis	%	e_PCB	1.394	0.001	14.0	normal	1.394	0.345
Birth trauma	%	a_VB_shc	1.027	0.001	4.0	normal	1.027	0.331
Birth trauma	%	b_VB_cmc	0.718	0.001	4.0	normal	0.718	0.216
Birth trauma	%	c_ICB_shc	0.086	0.001	4.0	normal	0.086	0.027
Birth trauma	%	d_ICB_cmc	0.086*	0.001	4.0	normal	0.086*	0.027*
Birth trauma	%	e_PCB	0.032	0.001	4.0	normal	0.032	0.01
Neonatal infection	%	a_VB_shc	0.125	0.001	2.0	normal	0.125	0.059
Neonatal infection	%	b_VB_cmc	0.321	0.001	2.0	normal	0.321	0.148
Neonatal infection	%	c_ICB_shc	0.244	0.001	2.0	normal	0.244	0.121
Neonatal infection	%	d_ICB_cmc	0.244*	0.001	2.0	normal	0.244*	0.121*
Neonatal infection	%	e_PCB	0.105	0.001	2.0	normal	0.105	0.052

Note: Adapted from the ValueDecisions' predictions template (9). Options: [a_VB_shc] Vaginal birth with standard hospital care; [b_VB_cmc] Vaginal birth with continuous midwifery care; [c_ICB_shc] Intrapartum cesarean birth with standard hospital care; [d_ICB_cmc] Intrapartum cesarean birth with continuous midwifery care; [e_PCB] Prelabor cesarean birth.
* Approximate estimates based on women with the same childbirth mode in this scenario, irrespective of the model of care, as no events occurred with this option.

S1-9b. Prediction matrix for neonatal complication indicators, scenario 2

Indicator	Unit	Option	Prediction	Minimum	Maximum	Uncertainty distribution	Expectation value (mean)	Standard deviation
Primary adaptation problem	%	a_VB_shc	6.880	0.001	17.0	normal	6.880	0.814
Primary adaptation problem	%	b_VB_cmc	0.987	0.001	17.0	normal	0.987	0.09
Primary adaptation problem	%	c_ICB_shc	9.226	0.001	17.0	normal	9.226	1.065
Primary adaptation problem	%	d_ICB_cmc	2.628	0.001	17.0	normal	2.628	0.186
Primary adaptation problem	%	e_PCB	9.320	0.001	17.0	normal	9.320	1.133
Advanced neonatal acidosis	%	a_VB_shc	4.739	0.001	14.0	normal	4.739	1.424
Advanced neonatal acidosis	%	b_VB_cmc	7.400	0.001	14.0	normal	7.400	2.047
Advanced neonatal acidosis	%	c_ICB_shc	2.983	0.001	14.0	normal	2.983	0.537
Advanced neonatal acidosis	%	d_ICB_cmc	2.508	0.001	14.0	normal	2.508	0.525
Advanced neonatal acidosis	%	e_PCB	1.887	0.001	14.0	normal	1.887	0.471
Birth trauma	%	a_VB_shc	0.948	0.001	4.0	normal	0.948	0.319
Birth trauma	%	b_VB_cmc	0.657	0.001	4.0	normal	0.657	0.223
Birth trauma	%	c_ICB_shc	0.082	0.001	4.0	normal	0.082	0.024
Birth trauma	%	d_ICB_cmc	0.082*	0.001	4.0	normal	0.082*	0.024*
Birth trauma	%	e_PCB	0.032	0.001	4.0	normal	0.032	0.011
Neonatal infection	%	a_VB_shc	0.167	0.001	2.0	normal	0.167	0.082
Neonatal infection	%	b_VB_cmc	0.438	0.001	2.0	normal	0.438	0.239
Neonatal infection	%	c_ICB_shc	0.352	0.001	2.0	normal	0.352	0.171
Neonatal infection	%	d_ICB_cmc	0.352*	0.001	2.0	normal	0.352*	0.171*
Neonatal infection	%	e_PCB	0.150	0.001	2.0	normal	0.150	0.074

Note: Adapted from the ValueDecisions' predictions template (9). Options: [a_VB_shc] Vaginal birth with standard hospital care; [b_VB_cmc] Vaginal birth with continuous midwifery care; [c_ICB_shc] Intrapartum cesarean birth with standard hospital care; [d_ICB_cmc] Intrapartum cesarean birth with continuous midwifery care; [e_PCB] Prelabor cesarean birth.
* Approximate estimates based on women with the same childbirth mode in this scenario, irrespective of the model of care, as no events occurred with this option.

S1-9c. Prediction matrix for neonatal complication indicators, scenario 3

Indicator	Unit	Option	Prediction	Minimum	Maximum	Uncertainty distribution	Expectation value (mean)	Standard deviation
Primary adaptation problem	%	a_VB_shc	4.659	0.001	17.0	normal	4.659	0.586
Primary adaptation problem	%	b_VB_cmc	0.670	0.001	17.0	normal	0.670	0.082
Primary adaptation problem	%	c_ICB_shc	6.412	0.001	17.0	normal	6.412	0.836
Primary adaptation problem	%	d_ICB_cmc	1.759	0.001	17.0	normal	1.759	0.074
Primary adaptation problem	%	e_PCB	6.351	0.001	17.0	normal	6.351	0.758
Advanced neonatal acidosis	%	a_VB_shc	3.597	0.001	14.0	normal	3.597	0.937
Advanced neonatal acidosis	%	b_VB_cmc	5.846	0.001	14.0	normal	5.846	1.240
Advanced neonatal acidosis	%	c_ICB_shc	2.039	0.001	14.0	normal	2.039	0.370
Advanced neonatal acidosis	%	d_ICB_cmc	1.884	0.001	14.0	normal	1.884	0.049
Advanced neonatal acidosis	%	e_PCB	1.298	0.001	14.0	normal	1.298	0.284
Birth trauma	%	a_VB_shc	0.396	0.001	4.0	normal	0.396	0.130
Birth trauma	%	b_VB_cmc	0.271	0.001	4.0	normal	0.271	0.086
Birth trauma	%	c_ICB_shc	0.032	0.001	4.0	normal	0.032	0.011
Birth trauma	%	d_ICB_cmc	0.032*	0.001	4.0	normal	0.032*	0.011*
Birth trauma	%	e_PCB	0.012	0.001	4.0	normal	0.012	0.004
Neonatal infection	%	a_VB_shc	0.139	0.001	2.0	normal	0.139	0.064
Neonatal infection	%	b_VB_cmc	0.320	0.001	2.0	normal	0.320	0.160
Neonatal infection	%	c_ICB_shc	0.276	0.001	2.0	normal	0.276	0.135
Neonatal infection	%	d_ICB_cmc	0.276*	0.001	2.0	normal	0.276*	0.135*
Neonatal infection	%	e_PCB	0.125	0.001	2.0	normal	0.125	0.058

Note: Adapted from the ValueDecisions' predictions template (9). Options: [a_VB_shc] Vaginal birth with standard hospital care; [b_VB_cmc] Vaginal birth with continuous midwifery care; [c_ICB_shc] Intrapartum cesarean birth with standard hospital care; [d_ICB_cmc] Intrapartum cesarean birth with continuous midwifery care; [e_PCB] Prelabor cesarean birth.
* Approximate estimates based on women with the same childbirth mode in this scenario, irrespective of the model of care, as no events occurred with this option.

S1-9d. Prediction matrix for neonatal complication indicators, scenario 4

Indicator	Unit	Option	Prediction	Minimum	Maximum	Uncertainty distribution	Expectation value (mean)	Standard deviation
Primary adaptation problem	%	a_VB_shc	5.669	0.001	17.0	normal	5.669	0.843
Primary adaptation problem	%	b_VB_cmc	0.760	0.001	17.0	normal	0.760	0.024
Primary adaptation problem	%	c_ICB_shc	7.682	0.001	17.0	normal	7.682	1.206
Primary adaptation problem	%	d_ICB_cmc	2.089	0.001	17.0	normal	2.089	0.033
Primary adaptation problem	%	e_PCB	7.606	0.001	17.0	normal	7.606	1.046
Advanced neonatal acidosis	%	a_VB_shc	3.857	0.001	14.0	normal	3.857	1.173
Advanced neonatal acidosis	%	b_VB_cmc	7.480	0.001	14.0	normal	7.480	0.099
Advanced neonatal acidosis	%	c_ICB_shc	2.333	0.001	14.0	normal	2.333	0.444
Advanced neonatal acidosis	%	d_ICB_cmc	1.779	0.001	14.0	normal	1.779	0.480
Advanced neonatal acidosis	%	e_PCB	1.473	0.001	14.0	normal	1.473	0.343
Birth trauma	%	a_VB_shc	0.487	0.001	4.0	normal	0.487	0.172
Birth trauma	%	b_VB_cmc	0.409	0.001	4.0	normal	0.409	0.052
Birth trauma	%	c_ICB_shc	0.042	0.001	4.0	normal	0.042	0.014
Birth trauma	%	d_ICB_cmc	0.042[a]	0.001	4.0	normal	0.042[a]	0.014[a]
Birth trauma	%	e_PCB	0.015	0.001	4.0	normal	0.015	0.005
Neonatal infection	%	a_VB_shc	0.167[b]	0.001	2.0	normal	0.167[b]	0.082[b]
Neonatal infection	%	b_VB_cmc	0.438[b]	0.001	2.0	normal	0.438[b]	0.239[b]
Neonatal infection	%	c_ICB_shc	0.352[b]	0.001	2.0	normal	0.352[b]	0.171[b]
Neonatal infection	%	d_ICB_cmc	0.352[c]	0.001	2.0	normal	0.352[c]	0.171[c]
Neonatal infection	%	e_PCB	0.150[b]	0.001	2.0	normal	0.150[b]	0.074[b]

Note: Adapted from the ValueDecisions' predictions template (9). Options: [a_VB_shc] Vaginal birth with standard hospital care; [b_VB_cmc] Vaginal birth with continuous midwifery care; [c_ICB_shc] Intrapartum cesarean birth with standard hospital care; [d_ICB_cmc] Intrapartum cesarean birth with continuous midwifery care; [e_PCB] Prelabor cesarean birth. [a] Approximate estimates based on women with the same childbirth mode in this scenario, irrespective of the model of care, as no events occurred with this option. [b] Approximate estimates based on women with comorbidities, irrespective of cesarean birth history, as no events occurred with these options in scenario 4. [c] Approximate estimates for women with comorbidities and the same childbirth mode, irrespective of cesarean birth history and the model of care, as no events occurred with this option in scenario 4.

S1-9e. Preference matrix for neonatal complication indicators, all scenarios

Expert	Indicator	Unit	Worst	Best	Shape of value function	Indicator levels	Values of indicator levels	Global weight of indicator
E1	Primary adaptation problem	%	17.0	0.001	interpolate	17.00/14.20/10.50/6.00/0.001	0/0.25/0.5/0.75/1	0.279
E1	Advanced neonatal acidosis	%	14.0	0.001	interpolate	14.00/11.50/8.50/4.80/0.001	0/0.25/0.5/0.75/1	0.328
E1	Birth trauma	%	4.0	0.001	interpolate	4.00/3.30/2.50/1.50/0.001	0/0.25/0.5/0.75/1	0.164
E1	Neonatal infection	%	2.0	0.001	interpolate	2.00/1.65/1.25/0.75/0.001	0/0.25/0.5/0.75/1	0.230
E2	Primary adaptation problem	%	17.0	0.001	interpolate	17.00/15.00/12.00/8.00/0.001	0/0.25/0.5/0.75/1	0.233
E2	Advanced neonatal acidosis	%	14.0	0.001	interpolate	14.00/12.00/9.00/5.00/0.001	0/0.25/0.5/0.75/1	0.333
E2	Birth trauma	%	4.0	0.001	interpolate	4.00/3.00/2.00/1.00/0.001	0/0.25/0.5/0.75/1	0.133
E2	Neonatal infection	%	2.0	0.001	interpolate	2.00/1.80/1.50/0.80/0.001	0/0.25/0.5/0.75/1	0.300
E6	Primary adaptation problem	%	17.0	0.001	interpolate	17.00/15.50/12.00/7.00/0.001	0/0.25/0.5/0.75/1	0.250
E6	Advanced neonatal acidosis	%	14.0	0.001	interpolate	14.00/12.50/10.00/5.50/0.001	0/0.25/0.5/0.75/1	0.313
E6	Birth trauma	%	4.0	0.001	interpolate	4.00/3.25/2.50/1.50/0.001	0/0.25/0.5/0.75/1	0.156
E6	Neonatal infection	%	2.0	0.001	interpolate	2.00/1.70/1.30/0.85/0.001	0/0.25/0.5/0.75/1	0.281
E_pooled	Primary adaptation problem	%	17.0	0.001	interpolate	17.00/14.90/11.50/7.00/0.001	0/0.25/0.5/0.75/1	0.254
E_pooled	Advanced neonatal acidosis	%	14.0	0.001	interpolate	14.00/12.00/9.17/5.10/0.001	0/0.25/0.5/0.75/1	0.325
E_pooled	Birth trauma	%	4.0	0.001	interpolate	4.00/3.18/2.33/1.33/0.001	0/0.25/0.5/0.75/1	0.151
E_pooled	Neonatal infection	%	2.0	0.001	interpolate	2.00/1.72/1.35/0.80/0.001	0/0.25/0.5/0.75/1	0.270

Note: Adapted from the ValueDecisions' preferences template (9).

S1-10. Main model: Outputs of the weighted composite index of neonatal complication indicators with expert value functions and γ=0.2

S1-10a. Overall values of options for neonatal complication indicators with expert value functions and γ=0.2, scenario 1

Expert	Option	Mean overall value	5% quantile of overall value	95% quantile of overall value	Minimum	Maximum
E1	a_VB_shc	0.848	0.808	0.884	0.762	0.92
E1	b_VB_cmc	0.839	0.768	0.899	0.663	0.97
E1	c_ICB_shc	0.867	0.842	0.891	0.81	0.915
E1	d_ICB_cmc	0.927	0.908	0.945	0.886	0.961
E1	e_PCB	0.894	0.872	0.912	0.85	0.937
E2	a_VB_shc	0.858	0.819	0.897	0.771	0.931
E2	b_VB_cmc	0.837	0.771	0.895	0.674	0.97
E2	c_ICB_shc	0.892	0.869	0.913	0.851	0.931
E2	d_ICB_cmc	0.93	0.909	0.951	0.882	0.968
E2	e_PCB	0.921	0.906	0.935	0.891	0.951
E6	a_VB_shc	0.871	0.838	0.902	0.797	0.932
E6	b_VB_cmc	0.862	0.808	0.91	0.732	0.974
E6	c_ICB_shc	0.89	0.869	0.91	0.849	0.93
E6	d_ICB_cmc	0.935	0.917	0.953	0.893	0.969
E6	e_PCB	0.915	0.9	0.93	0.883	0.948
E_pooled	a_VB_shc	0.86	0.824	0.895	0.778	0.928
E_pooled	b_VB_cmc	0.847	0.785	0.902	0.69	0.972
E_pooled	c_ICB_shc	0.885	0.862	0.906	0.839	0.926
E_pooled	d_ICB_cmc	0.931	0.912	0.95	0.887	0.967
E_pooled	e_PCB	0.912	0.896	0.927	0.877	0.946

Note: Overall values for each option and each expert using elicited expert value functions and a non-additive aggregation model with γ=0.2 for scenario 1: 0 (highest/"worst" complication rates) to 1 (lowest/"best" complication rates). Uncertainty in the predictions was accounted for by 2,000 Monte Carlo simulations. Options: [a_VB_shc] Vaginal birth with standard hospital care; [b_VB_cmc] Vaginal birth with continuous midwifery care; [c_ICB_shc] Intrapartum cesarean birth with standard hospital care; [d_ICB_cmc] Intrapartum cesarean birth with continuous midwifery care; [e_PCB] Prelabor cesarean birth.

S1-10b. Overall values of options for neonatal complication indicators with expert value functions and γ=0.2, scenario 2

Expert	Option	Mean overall value	5% quantile of overall value	95% quantile of overall value	Minimum	Maximum
E1	a_VB_shc	0.788	0.73	0.841	0.654	0.884
E1	b_VB_cmc	0.773	0.65	0.869	0.379	0.942
E1	c_ICB_shc	0.787	0.742	0.829	0.681	0.873
E1	d_ICB_cmc	0.896	0.869	0.922	0.83	0.949
E1	e_PCB	0.818	0.775	0.857	0.718	0.903
E2	a_VB_shc	0.817	0.763	0.866	0.702	0.91
E2	b_VB_cmc	0.776	0.661	0.866	0.386	0.94
E2	c_ICB_shc	0.832	0.79	0.87	0.737	0.909
E2	d_ICB_cmc	0.902	0.871	0.932	0.833	0.958
E2	e_PCB	0.868	0.832	0.901	0.784	0.93
E6	a_VB_shc	0.827	0.782	0.869	0.727	0.907
E6	b_VB_cmc	0.811	0.718	0.885	0.439	0.948
E6	c_ICB_shc	0.828	0.79	0.864	0.744	0.903
E6	d_ICB_cmc	0.908	0.881	0.934	0.84	0.958
E6	e_PCB	0.858	0.826	0.89	0.784	0.923
E_pooled	a_VB_shc	0.813	0.762	0.861	0.698	0.903
E_pooled	b_VB_cmc	0.788	0.675	0.874	0.399	0.944
E_pooled	c_ICB_shc	0.817	0.776	0.856	0.723	0.898
E_pooled	d_ICB_cmc	0.903	0.875	0.93	0.835	0.955
E_pooled	e_PCB	0.849	0.813	0.884	0.763	0.92

Note: Overall values for each option and each expert using elicited expert value functions and a non-additive aggregation model with γ=0.2 for scenario 2: 0 (highest/"worst" complication rates) to 1 (lowest/"best" complication rates). Uncertainty in the predictions was accounted for by 2,000 Monte Carlo simulations. Options: [a_VB_shc] Vaginal birth with standard hospital care; [b_VB_cmc] Vaginal birth with continuous midwifery care; [c_ICB_shc] Intrapartum cesarean birth with standard hospital care; [d_ICB_cmc] Intrapartum cesarean birth with continuous midwifery care; [e_PCB] Prelabor cesarean birth.

S1-10c. Overall values of options for neonatal complication indicators with expert value functions and γ=0.2, scenario 3

Expert	Option	Mean overall value	5% quantile of overall value	95% quantile of overall value	Minimum	Maximum
E1	a_VB_shc	0.86	0.827	0.89	0.775	0.921
E1	b_VB_cmc	0.845	0.786	0.897	0.701	0.928
E1	c_ICB_shc	0.861	0.83	0.889	0.797	0.918
E1	d_ICB_cmc	0.925	0.907	0.941	0.893	0.947
E1	e_PCB	0.887	0.863	0.909	0.84	0.932
E2	a_VB_shc	0.877	0.846	0.907	0.799	0.934
E2	b_VB_cmc	0.846	0.79	0.897	0.715	0.929
E2	c_ICB_shc	0.889	0.863	0.913	0.828	0.93
E2	d_ICB_cmc	0.928	0.907	0.948	0.89	0.955
E2	e_PCB	0.918	0.903	0.933	0.886	0.948
E6	a_VB_shc	0.883	0.856	0.909	0.818	0.934
E6	b_VB_cmc	0.869	0.822	0.91	0.771	0.938
E6	c_ICB_shc	0.886	0.86	0.91	0.827	0.929
E6	d_ICB_cmc	0.933	0.915	0.951	0.9	0.957
E6	e_PCB	0.91	0.893	0.927	0.876	0.945
E_pooled	a_VB_shc	0.875	0.845	0.903	0.8	0.93
E_pooled	b_VB_cmc	0.854	0.801	0.902	0.729	0.932
E_pooled	c_ICB_shc	0.881	0.854	0.906	0.82	0.926
E_pooled	d_ICB_cmc	0.929	0.91	0.947	0.895	0.953
E_pooled	e_PCB	0.907	0.889	0.924	0.869	0.943

Note: Overall values for each option and each expert using elicited expert value functions and a non-additive aggregation model with γ=0.2 for scenario 3: 0 (highest/"worst" complication rates) to 1 (lowest/"best" complication rates). Uncertainty in the predictions was accounted for by 2,000 Monte Carlo simulations. Options: [a_VB_shc] Vaginal birth with standard hospital care; [b_VB_cmc] Vaginal birth with continuous midwifery care; [c_ICB_shc] Intrapartum cesarean birth with standard hospital care; [d_ICB_cmc] Intrapartum cesarean birth with continuous midwifery care; [e_PCB] Prelabor cesarean birth.

S1-10d. Overall values of options for neonatal complication indicators with expert value functions and γ=0.2, scenario 4

Expert	Option	Mean overall value	5% quantile of overall value	95% quantile of overall value	Minimum	Maximum
E1	a_VB_shc	0.838	0.793	0.879	0.728	0.924
E1	b_VB_cmc	0.788	0.756	0.812	0.675	0.822
E1	c_ICB_shc	0.827	0.784	0.869	0.722	0.904
E1	d_ICB_cmc	0.915	0.89	0.94	0.858	0.961
E1	e_PCB	0.859	0.824	0.893	0.773	0.917
E2	a_VB_shc	0.86	0.819	0.898	0.753	0.938
E2	b_VB_cmc	0.79	0.756	0.82	0.691	0.831
E2	c_ICB_shc	0.864	0.826	0.898	0.77	0.93
E2	d_ICB_cmc	0.919	0.889	0.947	0.857	0.969
E2	e_PCB	0.9	0.873	0.922	0.834	0.939
E6	a_VB_shc	0.866	0.831	0.9	0.78	0.937
E6	b_VB_cmc	0.823	0.791	0.849	0.689	0.859
E6	c_ICB_shc	0.859	0.822	0.894	0.773	0.922
E6	d_ICB_cmc	0.924	0.898	0.949	0.87	0.968
E6	e_PCB	0.89	0.862	0.915	0.828	0.934
E_pooled	a_VB_shc	0.856	0.817	0.893	0.756	0.934
E_pooled	b_VB_cmc	0.802	0.769	0.829	0.689	0.839
E_pooled	c_ICB_shc	0.852	0.811	0.889	0.757	0.919
E_pooled	d_ICB_cmc	0.92	0.893	0.946	0.862	0.967
E_pooled	e_PCB	0.885	0.854	0.912	0.815	0.931

Note: Overall values for each option and each expert using elicited expert value functions and a non-additive aggregation model with γ=0.2 for scenario 4: 0 (highest/"worst" complication rates) to 1 (lowest/"best" complication rates). Uncertainty in the predictions was accounted for by 2,000 Monte Carlo simulations. Options: [a_VB_shc] Vaginal birth with standard hospital care; [b_VB_cmc] Vaginal birth with continuous midwifery care; [c_ICB_shc] Intrapartum cesarean birth with standard hospital care; [d_ICB_cmc] Intrapartum cesarean birth with continuous midwifery care; [e_PCB] Prelabor cesarean birth.

S1-11. Sensitivity analyses: Outputs of the weighted composite index of neonatal complication indicators with linear value functions and γ=0.2

S1-11a. Overall values of options for neonatal complication indicators with linear value functions and γ=0.2, scenario 1

Expert	Option	Mean overall value	5% quantile of overall value	95% quantile of overall value	Minimum	Maximum
E1	a_VB_shc	0.783	0.732	0.834	0.676	0.886
E1	b_VB_cmc	0.778	0.695	0.856	0.587	0.957
E1	c_ICB_shc	0.812	0.778	0.844	0.743	0.879
E1	d_ICB_cmc	0.896	0.868	0.923	0.833	0.946
E1	e_PCB	0.848	0.821	0.874	0.794	0.91
E2	a_VB_shc	0.796	0.744	0.847	0.686	0.896
E2	b_VB_cmc	0.772	0.685	0.853	0.574	0.959
E2	c_ICB_shc	0.819	0.783	0.854	0.751	0.886
E2	d_ICB_cmc	0.891	0.858	0.924	0.813	0.95
E2	e_PCB	0.861	0.836	0.885	0.81	0.916
E6	a_VB_shc	0.793	0.742	0.843	0.688	0.891
E6	b_VB_cmc	0.779	0.696	0.856	0.591	0.958
E6	c_ICB_shc	0.818	0.783	0.852	0.751	0.885
E6	d_ICB_cmc	0.894	0.863	0.924	0.82	0.95
E6	e_PCB	0.858	0.832	0.883	0.806	0.914
E_pooled	a_VB_shc	0.791	0.739	0.841	0.684	0.891
E_pooled	b_VB_cmc	0.776	0.693	0.855	0.584	0.958
E_pooled	c_ICB_shc	0.816	0.781	0.85	0.751	0.883
E_pooled	d_ICB_cmc	0.894	0.863	0.923	0.822	0.948
E_pooled	e_PCB	0.856	0.83	0.881	0.803	0.914

Note: Overall values for each option and each expert using linear value functions and a non-additive aggregation model with γ=0.2 for scenario 1: 0 (highest/"worst" complication rates) to 1 (lowest/"best" complication rates). Uncertainty in the predictions was accounted for by 2,000 Monte Carlo simulations. Options: [a_VB_shc] Vaginal birth with standard hospital care; [b_VB_cmc] Vaginal birth with continuous midwifery care; [c_ICB_shc] Intrapartum cesarean birth with standard hospital care; [d_ICB_cmc] Intrapartum cesarean birth with continuous midwifery care; [e_PCB] Prelabor cesarean birth.

S1-11b. Overall values of options for neonatal complication indicators with linear value functions and γ=0.2, scenario 2

Expert	Option	Mean overall value	5% quantile of overall value	95% quantile of overall value	Minimum	Maximum
E1	a_VB_shc	0.708	0.64	0.775	0.559	0.834
E1	b_VB_cmc	0.701	0.57	0.813	0.336	0.916
E1	c_ICB_shc	0.713	0.658	0.765	0.59	0.823
E1	d_ICB_cmc	0.852	0.811	0.89	0.763	0.928
E1	e_PCB	0.756	0.706	0.804	0.646	0.86
E2	a_VB_shc	0.725	0.655	0.793	0.577	0.853
E2	b_VB_cmc	0.691	0.557	0.809	0.322	0.918
E2	c_ICB_shc	0.727	0.671	0.779	0.599	0.841
E2	d_ICB_cmc	0.846	0.797	0.892	0.734	0.931
E2	e_PCB	0.779	0.734	0.822	0.678	0.874
E6	a_VB_shc	0.721	0.655	0.786	0.579	0.845
E6	b_VB_cmc	0.702	0.573	0.814	0.344	0.916
E6	c_ICB_shc	0.724	0.668	0.776	0.596	0.836
E6	d_ICB_cmc	0.85	0.804	0.894	0.744	0.931
E6	e_PCB	0.772	0.725	0.817	0.667	0.87
E_pooled	a_VB_shc	0.718	0.65	0.784	0.571	0.844
E_pooled	b_VB_cmc	0.698	0.568	0.812	0.335	0.917
E_pooled	c_ICB_shc	0.722	0.665	0.774	0.595	0.833
E_pooled	d_ICB_cmc	0.849	0.804	0.892	0.747	0.93
E_pooled	e_PCB	0.769	0.721	0.814	0.664	0.868

Note: Overall values for each option and each expert using linear value functions and a non-additive aggregation model with γ=0.2 for scenario 2: 0 (highest/"worst" complication rates) to 1 (lowest/"best" complication rates). Uncertainty in the predictions was accounted for by 2,000 Monte Carlo simulations. Options: [a_VB_shc] Vaginal birth with standard hospital care; [b_VB_cmc] Vaginal birth with continuous midwifery care; [c_ICB_shc] Intrapartum cesarean birth with standard hospital care; [d_ICB_cmc] Intrapartum cesarean birth with continuous midwifery care; [e_PCB] Prelabor cesarean birth.

S1-11c. Overall values of options for neonatal complication indicators with linear value functions and γ=0.2, scenario 3

Expert	Option	Mean overall value	5% quantile of overall value	95% quantile of overall value	Minimum	Maximum
E1	a_VB_shc	0.801	0.757	0.845	0.698	0.887
E1	b_VB_cmc	0.787	0.717	0.853	0.631	0.898
E1	c_ICB_shc	0.803	0.763	0.842	0.716	0.883
E1	d_ICB_cmc	0.893	0.866	0.917	0.843	0.926
E1	e_PCB	0.84	0.811	0.869	0.784	0.904
E2	a_VB_shc	0.81	0.766	0.854	0.705	0.895
E2	b_VB_cmc	0.778	0.703	0.848	0.619	0.898
E2	c_ICB_shc	0.811	0.768	0.852	0.711	0.882
E2	d_ICB_cmc	0.887	0.852	0.919	0.824	0.929
E2	e_PCB	0.853	0.826	0.881	0.8	0.911
E6	a_VB_shc	0.81	0.767	0.852	0.71	0.893
E6	b_VB_cmc	0.787	0.716	0.854	0.636	0.901
E6	c_ICB_shc	0.81	0.768	0.849	0.713	0.883
E6	d_ICB_cmc	0.89	0.858	0.921	0.831	0.93
E6	e_PCB	0.849	0.821	0.877	0.796	0.91
E_pooled	a_VB_shc	0.807	0.763	0.851	0.704	0.892
E_pooled	b_VB_cmc	0.784	0.711	0.852	0.629	0.899
E_pooled	c_ICB_shc	0.808	0.767	0.848	0.713	0.883
E_pooled	d_ICB_cmc	0.89	0.859	0.919	0.832	0.928
E_pooled	e_PCB	0.847	0.819	0.875	0.793	0.908

Note: Overall values for each option and each expert using linear value functions and a non-additive aggregation model with γ=0.2 for scenario 3: 0 (highest/"worst" complication rates) to 1 (lowest/"best" complication rates). Uncertainty in the predictions was accounted for by 2,000 Monte Carlo simulations. Options: [a_VB_shc] Vaginal birth with standard hospital care; [b_VB_cmc] Vaginal birth with continuous midwifery care; [c_ICB_shc] Intrapartum cesarean birth with standard hospital care; [d_ICB_cmc] Intrapartum cesarean birth with continuous midwifery care; [e_PCB] Prelabor cesarean birth.

S1-11d. Overall values of options for neonatal complication indicators with linear value functions and γ=0.2, scenario 4

Expert	Option	Mean overall value	5% quantile of overall value	95% quantile of overall value	Minimum	Maximum
E1	a_VB_shc	0.772	0.714	0.827	0.641	0.891
E1	b_VB_cmc	0.718	0.674	0.754	0.589	0.767
E1	c_ICB_shc	0.762	0.706	0.813	0.633	0.863
E1	d_ICB_cmc	0.879	0.84	0.915	0.797	0.945
E1	e_PCB	0.806	0.763	0.847	0.708	0.881
E2	a_VB_shc	0.783	0.726	0.839	0.647	0.901
E2	b_VB_cmc	0.705	0.649	0.751	0.543	0.767
E2	c_ICB_shc	0.771	0.716	0.825	0.645	0.873
E2	d_ICB_cmc	0.871	0.823	0.915	0.771	0.948
E2	e_PCB	0.823	0.783	0.86	0.738	0.893
E6	a_VB_shc	0.781	0.727	0.836	0.653	0.898
E6	b_VB_cmc	0.718	0.664	0.761	0.563	0.776
E6	c_ICB_shc	0.769	0.714	0.823	0.642	0.87
E6	d_ICB_cmc	0.875	0.83	0.916	0.78	0.947
E6	e_PCB	0.818	0.777	0.856	0.728	0.89
E_pooled	a_VB_shc	0.778	0.723	0.834	0.647	0.897
E_pooled	b_VB_cmc	0.714	0.662	0.755	0.565	0.77
E_pooled	c_ICB_shc	0.767	0.712	0.82	0.64	0.868
E_pooled	d_ICB_cmc	0.875	0.831	0.916	0.783	0.947
E_pooled	e_PCB	0.815	0.775	0.855	0.724	0.888

Note: Overall values for each option and each expert using linear value functions and a non-additive aggregation model with γ=0.2 for scenario 4: 0 (highest/"worst" complication rates) to 1 (lowest/"best" complication rates). Uncertainty in the predictions was accounted for by 2,000 Monte Carlo simulations. Options: [a_VB_shc] Vaginal birth with standard hospital care; [b_VB_cmc] Vaginal birth with continuous midwifery care; [c_ICB_shc] Intrapartum cesarean birth with standard hospital care; [d_ICB_cmc] Intrapartum cesarean birth with continuous midwifery care; [e_PCB] Prelabor cesarean birth.

S1-12. Sensitivity analyses: Outputs of the weighted composite index of neonatal complication indicators with expert value functions and γ=1

S1-12a. Overall values of options for neonatal complication indicators with expert value functions and γ=1, scenario 1

Expert	Option	Mean overall value	5% quantile of overall value	95% quantile of overall value	Minimum	Maximum
E1	a_VB_shc	0.85	0.812	0.885	0.769	0.92
E1	b_VB_cmc	0.847	0.788	0.901	0.716	0.97
E1	c_ICB_shc	0.871	0.847	0.893	0.822	0.917
E1	d_ICB_cmc	0.927	0.909	0.946	0.888	0.962
E1	e_PCB	0.898	0.878	0.915	0.858	0.938
E2	a_VB_shc	0.862	0.825	0.898	0.784	0.931
E2	b_VB_cmc	0.844	0.787	0.897	0.715	0.97
E2	c_ICB_shc	0.893	0.87	0.915	0.852	0.931
E2	d_ICB_cmc	0.93	0.91	0.951	0.884	0.968
E2	e_PCB	0.923	0.909	0.937	0.894	0.951
E6	a_VB_shc	0.873	0.841	0.903	0.803	0.933
E6	b_VB_cmc	0.867	0.82	0.912	0.761	0.974
E6	c_ICB_shc	0.892	0.871	0.913	0.853	0.931
E6	d_ICB_cmc	0.936	0.917	0.954	0.895	0.969
E6	e_PCB	0.918	0.903	0.932	0.888	0.949
E_pooled	a_VB_shc	0.863	0.827	0.897	0.786	0.929
E_pooled	b_VB_cmc	0.854	0.8	0.904	0.73	0.972
E_pooled	c_ICB_shc	0.887	0.865	0.908	0.847	0.927
E_pooled	d_ICB_cmc	0.932	0.913	0.95	0.889	0.967
E_pooled	e_PCB	0.914	0.899	0.929	0.882	0.947

Note: Overall values for each option and each expert using elicited expert value functions and an additive aggregation model with γ=1 for scenario 1: 0 (highest/"worst" complication rates) to 1 (lowest/"best" complication rates). Uncertainty in the predictions was accounted for by 2,000 Monte Carlo simulations. Options: [a_VB_shc] Vaginal birth with standard hospital care; [b_VB_cmc] Vaginal birth with continuous midwifery care; [c_ICB_shc] Intrapartum cesarean birth with standard hospital care; [d_ICB_cmc] Intrapartum cesarean birth with continuous midwifery care; [e_PCB] Prelabor cesarean birth.

S1-12b. Overall values of options for neonatal complication indicators with expert value functions and γ=1, scenario 2

Expert	Option	Mean overall value	5% quantile of overall value	95% quantile of overall value	Minimum	Maximum
E1	a_VB_shc	0.794	0.739	0.845	0.675	0.889
E1	b_VB_cmc	0.793	0.704	0.873	0.585	0.942
E1	c_ICB_shc	0.8	0.761	0.837	0.711	0.88
E1	d_ICB_cmc	0.897	0.871	0.923	0.837	0.949
E1	e_PCB	0.835	0.801	0.867	0.763	0.907
E2	a_VB_shc	0.821	0.771	0.868	0.72	0.912
E2	b_VB_cmc	0.792	0.706	0.87	0.57	0.941
E2	c_ICB_shc	0.838	0.799	0.873	0.75	0.912
E2	d_ICB_cmc	0.903	0.873	0.932	0.838	0.958
E2	e_PCB	0.876	0.845	0.905	0.81	0.932
E6	a_VB_shc	0.83	0.787	0.871	0.74	0.91
E6	b_VB_cmc	0.823	0.749	0.887	0.611	0.948
E6	c_ICB_shc	0.836	0.802	0.869	0.759	0.907
E6	d_ICB_cmc	0.909	0.883	0.935	0.846	0.958
E6	e_PCB	0.869	0.842	0.895	0.811	0.926
E_pooled	a_VB_shc	0.818	0.769	0.863	0.715	0.906
E_pooled	b_VB_cmc	0.804	0.72	0.878	0.588	0.944
E_pooled	c_ICB_shc	0.826	0.788	0.861	0.741	0.902
E_pooled	d_ICB_cmc	0.904	0.876	0.931	0.841	0.956
E_pooled	e_PCB	0.86	0.831	0.89	0.795	0.923

Note: Overall values for each option and each expert using elicited expert value functions and an additive aggregation model with γ=1 for scenario 2: 0 (highest/"worst" complication rates) to 1 (lowest/"best" complication rates). Uncertainty in the predictions was accounted for by 2,000 Monte Carlo simulations. Options: [a_VB_shc] Vaginal birth with standard hospital care; [b_VB_cmc] Vaginal birth with continuous midwifery care; [c_ICB_shc] Intrapartum cesarean birth with standard hospital care; [d_ICB_cmc] Intrapartum cesarean birth with continuous midwifery care; [e_PCB] Prelabor cesarean birth.

S1-12c. Overall values of options for neonatal complication indicators with expert value functions and γ=1, scenario 3

Expert	Option	Mean overall value	5% quantile of overall value	95% quantile of overall value	Minimum	Maximum
E1	a_VB_shc	0.862	0.831	0.892	0.785	0.922
E1	b_VB_cmc	0.854	0.804	0.9	0.747	0.93
E1	c_ICB_shc	0.865	0.837	0.893	0.804	0.919
E1	d_ICB_cmc	0.925	0.909	0.942	0.895	0.948
E1	e_PCB	0.892	0.871	0.912	0.851	0.934
E2	a_VB_shc	0.879	0.85	0.908	0.808	0.935
E2	b_VB_cmc	0.854	0.804	0.899	0.751	0.931
E2	c_ICB_shc	0.891	0.866	0.915	0.83	0.931
E2	d_ICB_cmc	0.929	0.908	0.949	0.892	0.955
E2	e_PCB	0.92	0.906	0.934	0.889	0.949
E6	a_VB_shc	0.885	0.86	0.91	0.825	0.935
E6	b_VB_cmc	0.874	0.833	0.912	0.79	0.939
E6	c_ICB_shc	0.889	0.864	0.912	0.831	0.93
E6	d_ICB_cmc	0.934	0.915	0.951	0.902	0.957
E6	e_PCB	0.914	0.898	0.929	0.881	0.946
E_pooled	a_VB_shc	0.877	0.849	0.904	0.808	0.93
E_pooled	b_VB_cmc	0.862	0.815	0.904	0.764	0.934
E_pooled	c_ICB_shc	0.884	0.857	0.908	0.823	0.926
E_pooled	d_ICB_cmc	0.93	0.911	0.948	0.897	0.954
E_pooled	e_PCB	0.91	0.894	0.926	0.875	0.944

Note: Overall values for each option and each expert using elicited expert value functions and an additive aggregation model with γ=1 for scenario 3: 0 (highest/"worst" complication rates) to 1 (lowest/"best" complication rates). Uncertainty in the predictions was accounted for by 2,000 Monte Carlo simulations. Options: [a_VB_shc] Vaginal birth with standard hospital care; [b_VB_cmc] Vaginal birth with continuous midwifery care; [c_ICB_shc] Intrapartum cesarean birth with standard hospital care; [d_ICB_cmc] Intrapartum cesarean birth with continuous midwifery care; [e_PCB] Prelabor cesarean birth.

S1-12d. Overall values of options for neonatal complication indicators with expert value functions and γ=1, scenario 4

Expert	Option	Mean overall value	5% quantile of overall value	95% quantile of overall value	Minimum	Maximum
E1	a_VB_shc	0.841	0.799	0.881	0.744	0.925
E1	b_VB_cmc	0.804	0.772	0.831	0.706	0.84
E1	c_ICB_shc	0.836	0.797	0.873	0.743	0.906
E1	d_ICB_cmc	0.916	0.892	0.941	0.863	0.961
E1	e_PCB	0.869	0.838	0.898	0.805	0.92
E2	a_VB_shc	0.862	0.824	0.899	0.766	0.939
E2	b_VB_cmc	0.804	0.768	0.835	0.709	0.847
E2	c_ICB_shc	0.868	0.832	0.901	0.78	0.932
E2	d_ICB_cmc	0.92	0.891	0.947	0.861	0.969
E2	e_PCB	0.904	0.879	0.925	0.852	0.941
E6	a_VB_shc	0.868	0.835	0.901	0.789	0.938
E6	b_VB_cmc	0.832	0.801	0.861	0.716	0.871
E6	c_ICB_shc	0.864	0.831	0.897	0.786	0.925
E6	d_ICB_cmc	0.925	0.9	0.949	0.874	0.968
E6	e_PCB	0.896	0.871	0.918	0.847	0.936
E_pooled	a_VB_shc	0.859	0.822	0.895	0.769	0.935
E_pooled	b_VB_cmc	0.815	0.781	0.844	0.713	0.854
E_pooled	c_ICB_shc	0.857	0.821	0.892	0.771	0.922
E_pooled	d_ICB_cmc	0.921	0.895	0.946	0.866	0.967
E_pooled	e_PCB	0.891	0.864	0.915	0.836	0.933

Note: Overall values for each option and each expert using elicited expert value functions and an additive aggregation model with γ=1 for scenario 4: 0 (highest/"worst" complication rates) to 1 (lowest/"best" complication rates). Uncertainty in the predictions was accounted for by 2,000 Monte Carlo simulations. Options: [a_VB_shc] Vaginal birth with standard hospital care; [b_VB_cmc] Vaginal birth with continuous midwifery care; [c_ICB_shc] Intrapartum cesarean birth with standard hospital care; [d_ICB_cmc] Intrapartum cesarean birth with continuous midwifery care; [e_PCB] Prelabor cesarean birth.

S1-13. Inputs to the weighted composite index of physiological childbirth indicators

S1-13a. Prediction matrix for physiological childbirth indicators, scenario 1

Indicator	Unit	Option	Prediction	Minimum	Maximum	Uncertainty distribution	Expectation value (mean)	Standard deviation
Spontaneous onset of labor	%	a_VB_shc	71.352	0	99.99	normal	71.352	1.236
Spontaneous onset of labor	%	b_VB_cmc	78.592	0	99.99	normal	78.592	0.813
Spontaneous onset of labor	%	c_ICB_shc	56.713	0	99.99	normal	56.713	1.338
Spontaneous onset of labor	%	d_ICB_cmc	59.875	0	99.99	normal	59.875	1.076
Spontaneous onset of labor	%	e_PCB	0	0	99.99	none	NA	NA
Spontaneous progression of labor	%	a_VB_shc	61.888	0	99.99	normal	61.888	9.68
Spontaneous progression of labor	%	b_VB_cmc	67.395	0	99.99	normal	67.395	9.187
Spontaneous progression of labor	%	c_ICB_shc	64.664	0	99.99	normal	64.664	5.679
Spontaneous progression of labor	%	d_ICB_cmc	69.461	0	99.99	normal	69.461	6.561
Spontaneous progression of labor	%	e_PCB	0	0	99.99	none	NA	NA
Childbirth without anesthesia	%	a_VB_shc	63.344	0	99.99	normal	63.344	10.619
Childbirth without anesthesia	%	b_VB_cmc	76.579	0	99.99	normal	76.579	8.114
Childbirth without anesthesia	%	c_ICB_shc	0	0	99.99	none	NA	NA
Childbirth without anesthesia	%	d_ICB_cmc	0	0	99.99	none	NA	NA
Childbirth without anesthesia	%	e_PCB	0	0	99.99	none	NA	NA
No iatrogenic obstetric wound	%	a_VB_shc	82.058	0	99.99	normal	82.058	12.916
No iatrogenic obstetric wound	%	b_VB_cmc	89.295	0	99.99	normal	89.295	8.914
No iatrogenic obstetric wound	%	c_ICB_shc	0	0	99.99	none	NA	NA
No iatrogenic obstetric wound	%	d_ICB_cmc	0	0	99.99	none	NA	NA
No iatrogenic obstetric wound	%	e_PCB	0	0	99.99	none	NA	NA
Spontaneous birth	%	a_VB_shc	88.192	0	99.99	normal	88.192	10.345
Spontaneous birth	%	b_VB_cmc	92.843	0	99.99	normal	92.843	6.762
Spontaneous birth	%	c_ICB_shc	0	0	99.99	none	NA	NA
Spontaneous birth	%	d_ICB_cmc	0	0	99.99	none	NA	NA
Spontaneous birth	%	e_PCB	0	0	99.99	none	NA	NA
Vaginal birth	%	a_VB_shc	100	0	100	none	NA	NA
Vaginal birth	%	b_VB_cmc	100	0	100	none	NA	NA
Vaginal birth	%	c_ICB_shc	0	0	100	none	NA	NA
Vaginal birth	%	d_ICB_cmc	0	0	100	none	NA	NA
Vaginal birth	%	e_PCB	0	0	100	none	NA	NA

Note: Adapted from the ValueDecisions' predictions template (9). Options: [a_VB_shc] Vaginal birth with standard hospital care; [b_VB_cmc] Vaginal birth with continuous midwifery care; [c_ICB_shc] Intrapartum cesarean birth with standard hospital care; [d_ICB_cmc] Intrapartum cesarean birth with continuous midwifery care; [e_PCB] Prelabor cesarean birth; NA: not applicable.

S1-13b. Prediction matrix for physiological childbirth indicators, scenario 2

Indicator	Unit	Option	Prediction	Minimum	Maximum	Uncertainty distribution	Expectation value (mean)	Standard deviation
Spontaneous onset of labor	%	a_VB_shc	43.184	0	99.99	normal	43.184	1.56
Spontaneous onset of labor	%	b_VB_cmc	52.683	0	99.99	normal	52.683	1.25
Spontaneous onset of labor	%	c_ICB_shc	28.557	0	99.99	normal	28.557	1.088
Spontaneous onset of labor	%	d_ICB_cmc	31.087	0	99.99	normal	31.087	0.448
Spontaneous onset of labor	%	e_PCB	0	0	99.99	none	NA	NA
Spontaneous progression of labor	%	a_VB_shc	61.764	0	99.99	normal	61.764	10.347
Spontaneous progression of labor	%	b_VB_cmc	66.526	0	99.99	normal	66.526	9.756
Spontaneous progression of labor	%	c_ICB_shc	63.885	0	99.99	normal	63.885	5.519
Spontaneous progression of labor	%	d_ICB_cmc	65.431	0	99.99	normal	65.431	6.975
Spontaneous progression of labor	%	e_PCB	0	0	99.99	none	NA	NA
Childbirth without anesthesia	%	a_VB_shc	58.564	0	99.99	normal	58.564	11.758
Childbirth without anesthesia	%	b_VB_cmc	71.729	0	99.99	normal	71.729	9.034
Childbirth without anesthesia	%	c_ICB_shc	0	0	99.99	none	NA	NA
Childbirth without anesthesia	%	d_ICB_cmc	0	0	99.99	none	NA	NA
Childbirth without anesthesia	%	e_PCB	0	0	99.99	none	NA	NA
No iatrogenic obstetric wound	%	a_VB_shc	84.968	0	99.99	normal	84.968	11.627
No iatrogenic obstetric wound	%	b_VB_cmc	91.046	0	99.99	normal	91.046	7.916
No iatrogenic obstetric wound	%	c_ICB_shc	0	0	99.99	none	NA	NA
No iatrogenic obstetric wound	%	d_ICB_cmc	0	0	99.99	none	NA	NA
No iatrogenic obstetric wound	%	e_PCB	0	0	99.99	none	NA	NA
Spontaneous birth	%	a_VB_shc	87.935	0	99.99	normal	87.935	10.681
Spontaneous birth	%	b_VB_cmc	92.494	0	99.99	normal	92.494	6.928
Spontaneous birth	%	c_ICB_shc	0	0	99.99	none	NA	NA
Spontaneous birth	%	d_ICB_cmc	0	0	99.99	none	NA	NA
Spontaneous birth	%	e_PCB	0	0	99.99	none	NA	NA
Vaginal birth	%	a_VB_shc	100	0	100	none	NA	NA
Vaginal birth	%	b_VB_cmc	100	0	100	none	NA	NA
Vaginal birth	%	c_ICB_shc	0	0	100	none	NA	NA
Vaginal birth	%	d_ICB_cmc	0	0	100	none	NA	NA
Vaginal birth	%	e_PCB	0	0	100	none	NA	NA

Note: Adapted from the ValueDecisions' predictions template (9). Options: [a_VB_shc] Vaginal birth with standard hospital care; [b_VB_cmc] Vaginal birth with continuous midwifery care; [c_ICB_shc] Intrapartum cesarean birth with standard hospital care; [d_ICB_cmc] Intrapartum cesarean birth with continuous midwifery care; [e_PCB] Prelabor cesarean birth; NA: not applicable.

S1-13c. Prediction matrix for physiological childbirth indicators, scenario 3

Indicator	Unit	Option	Prediction	Minimum	Maximum	Uncertainty distribution	Expectation value (mean)	Standard deviation
Spontaneous onset of labor	%	a_VB_shc	80.479	0	99.99	normal	80.479	0.929
Spontaneous onset of labor	%	b_VB_cmc	85.743	0	99.99	normal	85.743	0.514
Spontaneous onset of labor	%	c_ICB_shc	68.625	0	99.99	normal	68.625	1.122
Spontaneous onset of labor	%	d_ICB_cmc	71.164	0	99.99	normal	71.164	0.156
Spontaneous onset of labor	%	e_PCB	0	0	99.99	none	NA	NA
Spontaneous progression of labor	%	a_VB_shc	67.953	0	99.99	normal	67.953	8.165
Spontaneous progression of labor	%	b_VB_cmc	72.022	0	99.99	normal	72.022	6.665
Spontaneous progression of labor	%	c_ICB_shc	73.166	0	99.99	normal	73.166	4.956
Spontaneous progression of labor	%	d_ICB_cmc	72.851	0	99.99	normal	72.851	2.122
Spontaneous progression of labor	%	e_PCB	0	0	99.99	none	NA	NA
Childbirth without anesthesia	%	a_VB_shc	55.502	0	99.99	normal	55.502	10.774
Childbirth without anesthesia	%	b_VB_cmc	67.949	0	99.99	normal	67.949	8.377
Childbirth without anesthesia	%	c_ICB_shc	0	0	99.99	none	NA	NA
Childbirth without anesthesia	%	d_ICB_cmc	0	0	99.99	none	NA	NA
Childbirth without anesthesia	%	e_PCB	0	0	99.99	none	NA	NA
No iatrogenic obstetric wound	%	a_VB_shc	81.110	0	99.99	normal	81.110	12.506
No iatrogenic obstetric wound	%	b_VB_cmc	89.360	0	99.99	normal	89.360	8.023
No iatrogenic obstetric wound	%	c_ICB_shc	0	0	99.99	none	NA	NA
No iatrogenic obstetric wound	%	d_ICB_cmc	0	0	99.99	none	NA	NA
No iatrogenic obstetric wound	%	e_PCB	0	0	99.99	none	NA	NA
Spontaneous birth	%	a_VB_shc	86.822	0	99.99	normal	86.822	8.944
Spontaneous birth	%	b_VB_cmc	91.511	0	99.99	normal	91.511	5.390
Spontaneous birth	%	c_ICB_shc	0	0	99.99	none	NA	NA
Spontaneous birth	%	d_ICB_cmc	0	0	99.99	none	NA	NA
Spontaneous birth	%	e_PCB	0	0	99.99	none	NA	NA
Vaginal birth	%	a_VB_shc	100	0	100	none	NA	NA
Vaginal birth	%	b_VB_cmc	100	0	100	none	NA	NA
Vaginal birth	%	c_ICB_shc	0	0	100	none	NA	NA
Vaginal birth	%	d_ICB_cmc	0	0	100	none	NA	NA
Vaginal birth	%	e_PCB	0	0	100	none	NA	NA

Note: Adapted from the ValueDecisions' predictions template (9). Options: [a_VB_shc] Vaginal birth with standard hospital care; [b_VB_cmc] Vaginal birth with continuous midwifery care; [c_ICB_shc] Intrapartum cesarean birth with standard hospital care; [d_ICB_cmc] Intrapartum cesarean birth with continuous midwifery care; [e_PCB] Prelabor cesarean birth; NA: not applicable.

S1-13d. Prediction matrix for physiological childbirth indicators, scenario 4

Indicator	Unit	Option	Prediction	Minimum	Maximum	Uncertainty distribution	Expectation value (mean)	Standard deviation
Spontaneous onset of labor	%	a_VB_shc	66.009	0	99.99	normal	66.009	1.436
Spontaneous onset of labor	%	b_VB_cmc	73.880	0	99.99	normal	73.880	0.147
Spontaneous onset of labor	%	c_ICB_shc	50.862	0	99.99	normal	50.862	1.241
Spontaneous onset of labor	%	d_ICB_cmc	54.482	0	99.99	normal	54.482	0.964
Spontaneous onset of labor	%	e_PCB	0	0	99.99	none	NA	NA
Spontaneous progression of labor	%	a_VB_shc	72.905	0	99.99	normal	72.905	8.228
Spontaneous progression of labor	%	b_VB_cmc	70.851	0	99.99	normal	70.851	1.74
Spontaneous progression of labor	%	c_ICB_shc	76.234	0	99.99	normal	76.234	4.691
Spontaneous progression of labor	%	d_ICB_cmc	80.889	0	99.99	normal	80.889	5.218
Spontaneous progression of labor	%	e_PCB	0	0	99.99	none	NA	NA
Childbirth without anesthesia	%	a_VB_shc	54.106	0	99.99	normal	54.106	12.227
Childbirth without anesthesia	%	b_VB_cmc	62.808	0	99.99	normal	62.808	1.093
Childbirth without anesthesia	%	c_ICB_shc	0	0	99.99	none	NA	NA
Childbirth without anesthesia	%	d_ICB_cmc	0	0	99.99	none	NA	NA
Childbirth without anesthesia	%	e_PCB	0	0	99.99	none	NA	NA
No iatrogenic obstetric wound	%	a_VB_shc	85.241	0	99.99	normal	85.241	11.334
No iatrogenic obstetric wound	%	b_VB_cmc	82.975	0	99.99	normal	82.975	2.786
No iatrogenic obstetric wound	%	c_ICB_shc	0	0	99.99	none	NA	NA
No iatrogenic obstetric wound	%	d_ICB_cmc	0	0	99.99	none	NA	NA
No iatrogenic obstetric wound	%	e_PCB	0	0	99.99	none	NA	NA
Spontaneous birth	%	a_VB_shc	86.504	0	99.99	normal	86.504	10.998
Spontaneous birth	%	b_VB_cmc	83.092	0	99.99	normal	83.092	1.504
Spontaneous birth	%	c_ICB_shc	0	0	99.99	none	NA	NA
Spontaneous birth	%	d_ICB_cmc	0	0	99.99	none	NA	NA
Spontaneous birth	%	e_PCB	0	0	99.99	none	NA	NA
Vaginal birth	%	a_VB_shc	100	0	100	none	NA	NA
Vaginal birth	%	b_VB_cmc	100	0	100	none	NA	NA
Vaginal birth	%	c_ICB_shc	0	0	100	none	NA	NA
Vaginal birth	%	d_ICB_cmc	0	0	100	none	NA	NA
Vaginal birth	%	e_PCB	0	0	100	none	NA	NA

Note: Adapted from the ValueDecisions' predictions template (9). Options: [a_VB_shc] Vaginal birth with standard hospital care; [b_VB_cmc] Vaginal birth with continuous midwifery care; [c_ICB_shc] Intrapartum cesarean birth with standard hospital care; [d_ICB_cmc] Intrapartum cesarean birth with continuous midwifery care; [e_PCB] Prelabor cesarean birth; NA: not applicable.

S1-13e. Preference matrix for physiological childbirth indicators, all scenarios

Expert	Indicator	Unit	Worst	Best	Shape of value function	Indicator levels	Values of indicator levels	Global weight of indicator
E1	Spontaneous onset of labor	%	0	99.99	interpolate	0.00/20.00/45.00/72.00/99.99	0/0.25/0.5/0.75/1	0.219
E1	Spontaneous progression of labor	%	0	99.99	interpolate	0.00/20.00/45.00/72.00/99.99	0/0.25/0.5/0.75/1	0.031
E1	Childbirth without anesthesia	%	0	99.99	interpolate	0.00/19.00/42.00/70.00/99.99	0/0.25/0.5/0.75/1	0.094
E1	No iatrogenic obstetric wound	%	0	99.99	interpolate	0.00/16.00/40.00/65.00/99.99	0/0.25/0.5/0.75/1	0.188
E1	Spontaneous birth	%	0	99.99	interpolate	0.00/16.00/40.00/65.00/99.99	0/0.25/0.5/0.75/1	0.156
E1	Vaginal birth	%	0	100	interpolate	0.00/16.00/40.00/65.00/100.00	0/0.25/0.5/0.75/1	0.313
E4	Spontaneous onset of labor	%	0	99.99	linear*	0.00/99.99	0/1	0.175
E4	Spontaneous progression of labor	%	0	99.99	linear*	0.00/99.99	0/1	0.125
E4	Childbirth without anesthesia	%	0	99.99	linear*	0.00/99.99	0/1	0.150
E4	No iatrogenic obstetric wound	%	0	99.99	linear*	0.00/99.99	0/1	0.100
E4	Spontaneous birth	%	0	99.99	linear*	0.00/99.99	0/1	0.200
E4	Vaginal birth	%	0	100	linear*	0.00/100.00	0/1	0.250
E5	Spontaneous onset of labor	%	0	99.99	interpolate	0.00/5.00/20.00/50.00/99.99	0/0.25/0.5/0.75/1	0.211
E5	Spontaneous progression of labor	%	0	99.99	interpolate	0.00/10.00/33.30/55.00/99.99	0/0.25/0.5/0.75/1	0.194
E5	Childbirth without anesthesia	%	0	99.99	interpolate	0.00/20.00/40.00/70.00/99.99	0/0.25/0.5/0.75/1	0.116
E5	No iatrogenic obstetric wound	%	0	99.99	interpolate	0.00/15.00/40.00/65.00/99.99	0/0.25/0.5/0.75/1	0.137
E5	Spontaneous birth	%	0	99.99	interpolate	0.00/10.00/30.00/50.00/99.99	0/0.25/0.5/0.75/1	0.168
E5	Vaginal birth	%	0	100	interpolate	0.00/10.00/33.30/66.60/100.00	0/0.25/0.5/0.75/1	0.175
E_pooled	Spontaneous onset of labor	%	0	99.99	interpolate	0.00/12.50/32.50/61.00/99.99	0/0.25/0.5/0.75/1	0.202
E_pooled	Spontaneous progression of labor	%	0	99.99	interpolate	0.00/15.00/39.15/63.50/99.99	0/0.25/0.5/0.75/1	0.117
E_pooled	Childbirth without anesthesia	%	0	99.99	interpolate	0.00/19.50/41.00/70.00/99.99	0/0.25/0.5/0.75/1	0.120
E_pooled	No iatrogenic obstetric wound	%	0	99.99	interpolate	0.00/15.50/40.00/65.00/99.99	0/0.25/0.5/0.75/1	0.142
E_pooled	Spontaneous birth	%	0	99.99	interpolate	0.00/13.00/35.00/57.50/99.99	0/0.25/0.5/0.75/1	0.175
E_pooled	Vaginal birth	%	0	100	interpolate	0.00/13.00/36.65/65.80/100.00	0/0.25/0.5/0.75/1	0.246

Note: Adapted from the ValueDecisions' preferences template (9). * Linear value functions were assumed, as expert 4 did not provide preferences for within-goal changes. For the weighted composite index of physiological childbirth indicators, value functions were pooled across experts 1 and 5.

S1-14. Main model: Outputs of the weighted composite index of physiological childbirth indicators with expert value functions and γ=0.2

S1-14a. Overall values of options for physiological childbirth indicators with expert value functions and γ=0.2, scenario 1

Expert	Option	Mean overall value	5% quantile of overall value	95% quantile of overall value	Minimum	Maximum
E1	a_VB_shc	0.854	0.816	0.887	0.758	0.911
E1	b_VB_cmc	0.9	0.876	0.921	0.847	0.939
E1	c_ICB_shc	0.001	0.001	0.001	0.001	0.001
E1	d_ICB_cmc	0.001	0.001	0.001	0.001	0.001
E1	e_PCB	0	0	0	0	0
E4	a_VB_shc	0.787	0.733	0.836	0.657	0.883
E4	b_VB_cmc	0.849	0.811	0.885	0.754	0.918
E4	c_ICB_shc	0.001	0.001	0.002	0.001	0.002
E4	d_ICB_cmc	0.002	0.001	0.002	0.001	0.002
E4	e_PCB	0	0	0	0	0
E5	a_VB_shc	0.855	0.814	0.89	0.738	0.921
E5	b_VB_cmc	0.896	0.867	0.923	0.824	0.946
E5	c_ICB_shc	0.009	0.008	0.009	0.008	0.009
E5	d_ICB_cmc	0.009	0.009	0.009	0.008	0.01
E5	e_PCB	0	0	0	0	0
E_pooled	a_VB_shc	0.852	0.813	0.888	0.75	0.913
E_pooled	b_VB_cmc	0.896	0.868	0.921	0.828	0.945
E_pooled	c_ICB_shc	0.002	0.002	0.002	0.002	0.003
E_pooled	d_ICB_cmc	0.002	0.002	0.003	0.002	0.003
E_pooled	e_PCB	0	0	0	0	0

Note: Overall values for each option and each expert using elicited expert value functions and a non-additive aggregation model with γ=0.2 for scenario 1: 0 (least physiological childbirth/"worst") to 1 (most physiological childbirth/"best"). Uncertainty in the predictions was accounted for by 2,000 Monte Carlo simulations. Options: [a_VB_shc] Vaginal birth with standard hospital care; [b_VB_cmc] Vaginal birth with continuous midwifery care; [c_ICB_shc] Intrapartum cesarean birth with standard hospital care; [d_ICB_cmc] Intrapartum cesarean birth with continuous midwifery care; [e_PCB] Prelabor cesarean birth.

S1-14b. Overall values of options for physiological childbirth indicators with expert value functions and γ=0.2, scenario 2

Expert	Option	Mean overall value	5% quantile of overall value	95% quantile of overall value	Minimum	Maximum
E1	a_VB_shc	0.778	0.745	0.81	0.702	0.833
E1	b_VB_cmc	0.835	0.812	0.855	0.774	0.872
E1	c_ICB_shc	0	0	0	0	0
E1	d_ICB_cmc	0	0	0	0	0
E1	e_PCB	0	0	0	0	0
E4	a_VB_shc	0.715	0.664	0.767	0.604	0.803
E4	b_VB_cmc	0.787	0.746	0.824	0.689	0.863
E4	c_ICB_shc	0.001	0.001	0.001	0.001	0.001
E4	d_ICB_cmc	0.001	0.001	0.001	0.001	0.001
E4	e_PCB	0	0	0	0	0
E5	a_VB_shc	0.813	0.772	0.85	0.683	0.876
E5	b_VB_cmc	0.863	0.832	0.89	0.774	0.916
E5	c_ICB_shc	0.007	0.007	0.008	0.007	0.008
E5	d_ICB_cmc	0.007	0.007	0.008	0.007	0.008
E5	e_PCB	0	0	0	0	0
E_pooled	a_VB_shc	0.795	0.758	0.832	0.702	0.858
E_pooled	b_VB_cmc	0.85	0.821	0.876	0.773	0.901
E_pooled	c_ICB_shc	0.002	0.002	0.002	0.002	0.002
E_pooled	d_ICB_cmc	0.002	0.002	0.002	0.002	0.002
E_pooled	e_PCB	0	0	0	0	0

Note: Overall values for each option and each expert using elicited expert value functions and a non-additive aggregation model with γ=0.2 for scenario 2: 0 (least physiological childbirth/"worst") to 1 (most physiological childbirth/"best"). Uncertainty in the predictions was accounted for by 2,000 Monte Carlo simulations. Options: [a_VB_shc] Vaginal birth with standard hospital care; [b_VB_cmc] Vaginal birth with continuous midwifery care; [c_ICB_shc] Intrapartum cesarean birth with standard hospital care; [d_ICB_cmc] Intrapartum cesarean birth with continuous midwifery care; [e_PCB] Prelabor cesarean birth.

S1-14c. Overall values of options for physiological childbirth indicators with expert value functions and γ=0.2, scenario 3

Expert	Option	Mean overall value	5% quantile of overall value	95% quantile of overall value	Minimum	Maximum
E1	a_VB_shc	0.866	0.828	0.899	0.779	0.924
E1	b_VB_cmc	0.909	0.886	0.93	0.857	0.95
E1	c_ICB_shc	0.001	0.001	0.001	0.001	0.001
E1	d_ICB_cmc	0.001	0.001	0.001	0.001	0.001
E1	e_PCB	0	0	0	0	0
E4	a_VB_shc	0.796	0.744	0.844	0.664	0.888
E4	b_VB_cmc	0.854	0.818	0.888	0.772	0.923
E4	c_ICB_shc	0.002	0.002	0.002	0.002	0.002
E4	d_ICB_cmc	0.002	0.002	0.002	0.002	0.002
E4	e_PCB	0	0	0	0	0
E5	a_VB_shc	0.862	0.826	0.896	0.765	0.923
E5	b_VB_cmc	0.901	0.877	0.924	0.848	0.947
E5	c_ICB_shc	0.009	0.009	0.009	0.009	0.01
E5	d_ICB_cmc	0.009	0.009	0.009	0.009	0.009
E5	e_PCB	0	0	0	0	0
E_pooled	a_VB_shc	0.86	0.822	0.894	0.761	0.923
E_pooled	b_VB_cmc	0.901	0.877	0.924	0.846	0.947
E_pooled	c_ICB_shc	0.003	0.003	0.003	0.002	0.003
E_pooled	d_ICB_cmc	0.003	0.003	0.003	0.003	0.003
E_pooled	e_PCB	0	0	0	0	0

Note: Overall values for each option and each expert using elicited expert value functions and a non-additive aggregation model with γ=0.2 for scenario 3: 0 (least physiological childbirth/"worst") to 1 (most physiological childbirth/"best"). Uncertainty in the predictions was accounted for by 2,000 Monte Carlo simulations. Options: [a_VB_shc] Vaginal birth with standard hospital care; [b_VB_cmc] Vaginal birth with continuous midwifery care; [c_ICB_shc] Intrapartum cesarean birth with standard hospital care; [d_ICB_cmc] Intrapartum cesarean birth with continuous midwifery care; [e_PCB] Prelabor cesarean birth.

S1-14d. Overall values of options for physiological childbirth indicators with expert value functions and γ=0.2, scenario 4

Expert	Option	Mean overall value	5% quantile of overall value	95% quantile of overall value	Minimum	Maximum
E1	a_VB_shc	0.837	0.797	0.872	0.754	0.902
E1	b_VB_cmc	0.864	0.858	0.871	0.846	0.879
E1	c_ICB_shc	0.001	0.001	0.001	0.001	0.001
E1	d_ICB_cmc	0.001	0.001	0.001	0.001	0.001
E1	e_PCB	0	0	0	0	0
E4	a_VB_shc	0.773	0.714	0.827	0.651	0.871
E4	b_VB_cmc	0.803	0.795	0.812	0.785	0.82
E4	c_ICB_shc	0.001	0.001	0.002	0.001	0.002
E4	d_ICB_cmc	0.002	0.001	0.002	0.001	0.002
E4	e_PCB	0	0	0	0	0
E5	a_VB_shc	0.853	0.811	0.888	0.756	0.915
E5	b_VB_cmc	0.871	0.865	0.877	0.859	0.882
E5	c_ICB_shc	0.009	0.009	0.009	0.008	0.009
E5	d_ICB_cmc	0.009	0.009	0.009	0.008	0.009
E5	e_PCB	0	0	0	0	0
E_pooled	a_VB_shc	0.845	0.803	0.882	0.752	0.908
E_pooled	b_VB_cmc	0.867	0.861	0.873	0.854	0.878
E_pooled	c_ICB_shc	0.002	0.002	0.002	0.002	0.002
E_pooled	d_ICB_cmc	0.002	0.002	0.003	0.002	0.003
E_pooled	e_PCB	0	0	0	0	0

Note: Overall values for each option and each expert using elicited expert value functions and a non-additive aggregation model with γ=0.2 for scenario 4: 0 (least physiological childbirth/"worst") to 1 (most physiological childbirth/"best"). Uncertainty in the predictions was accounted for by 2,000 Monte Carlo simulations. Options: [a_VB_shc] Vaginal birth with standard hospital care; [b_VB_cmc] Vaginal birth with continuous midwifery care; [c_ICB_shc] Intrapartum cesarean birth with standard hospital care; [d_ICB_cmc] Intrapartum cesarean birth with continuous midwifery care; [e_PCB] Prelabor cesarean birth.

S1-15. Sensitivity analyses: Outputs of the weighted composite index of physiological childbirth indicators with linear value functions and γ=0.2

S1-15a. Overall values of options for physiological childbirth indicators with linear value functions and γ=0.2, scenario 1

Expert	Option	Mean overall value	5% quantile of overall value	95% quantile of overall value	Minimum	Maximum
E1	a_VB_shc	0.821	0.772	0.865	0.705	0.898
E1	b_VB_cmc	0.878	0.845	0.906	0.807	0.929
E1	c_ICB_shc	0.001	0.001	0.001	0.001	0.001
E1	d_ICB_cmc	0.001	0.001	0.001	0.001	0.001
E1	e_PCB	0	0	0	0	0
E4	a_VB_shc	0.787	0.733	0.836	0.657	0.883
E4	b_VB_cmc	0.849	0.811	0.885	0.754	0.918
E4	c_ICB_shc	0.001	0.001	0.002	0.001	0.002
E4	d_ICB_cmc	0.002	0.001	0.002	0.001	0.002
E4	e_PCB	0	0	0	0	0
E5	a_VB_shc	0.761	0.705	0.813	0.625	0.87
E5	b_VB_cmc	0.824	0.778	0.868	0.72	0.9
E5	c_ICB_shc	0.007	0.006	0.007	0.005	0.008
E5	d_ICB_cmc	0.007	0.006	0.008	0.005	0.008
E5	e_PCB	0	0	0	0	0
E_pooled	a_VB_shc	0.789	0.739	0.837	0.672	0.875
E_pooled	b_VB_cmc	0.85	0.813	0.884	0.76	0.915
E_pooled	c_ICB_shc	0.002	0.002	0.002	0.002	0.002
E_pooled	d_ICB_cmc	0.002	0.002	0.002	0.002	0.002
E_pooled	e_PCB	0	0	0	0	0

Note: Overall values for each option and each expert using linear value functions and a non-additive aggregation model with γ=0.2 for scenario 1: 0 (least physiological childbirth/"worst") to 1 (most physiological childbirth/"best"). Uncertainty in the predictions was accounted for by 2,000 Monte Carlo simulations. Options: [a_VB_shc] Vaginal birth with standard hospital care; [b_VB_cmc] Vaginal birth with continuous midwifery care; [c_ICB_shc] Intrapartum cesarean birth with standard hospital care; [d_ICB_cmc] Intrapartum cesarean birth with continuous midwifery care; [e_PCB] Prelabor cesarean birth.

S1-15b. Overall values of options for physiological childbirth indicators with linear value functions and γ=0.2, scenario 2

Expert	Option	Mean overall value	5% quantile of overall value	95% quantile of overall value	Minimum	Maximum
E1	a_VB_shc	0.738	0.694	0.779	0.644	0.81
E1	b_VB_cmc	0.807	0.777	0.833	0.725	0.854
E1	c_ICB_shc	0	0	0	0	0
E1	d_ICB_cmc	0	0	0	0	0
E1	e_PCB	0	0	0	0	0
E4	a_VB_shc	0.715	0.664	0.767	0.604	0.803
E4	b_VB_cmc	0.787	0.746	0.824	0.689	0.863
E4	c_ICB_shc	0.001	0.001	0.001	0.001	0.001
E4	d_ICB_cmc	0.001	0.001	0.001	0.001	0.001
E4	e_PCB	0	0	0	0	0
E5	a_VB_shc	0.682	0.631	0.734	0.54	0.769
E5	b_VB_cmc	0.755	0.708	0.797	0.641	0.839
E5	c_ICB_shc	0.005	0.004	0.005	0.004	0.005
E5	d_ICB_cmc	0.005	0.004	0.005	0.004	0.006
E5	e_PCB	0	0	0	0	0
E_pooled	a_VB_shc	0.711	0.665	0.757	0.61	0.792
E_pooled	b_VB_cmc	0.783	0.746	0.816	0.689	0.849
E_pooled	c_ICB_shc	0.001	0.001	0.001	0.001	0.001
E_pooled	d_ICB_cmc	0.001	0.001	0.001	0.001	0.002
E_pooled	e_PCB	0	0	0	0	0

Note: Overall values for each option and each expert using linear value functions and a non-additive aggregation model with γ=0.2 for scenario 2: 0 (least physiological childbirth/"worst") to 1 (most physiological childbirth/"best"). Uncertainty in the predictions was accounted for by 2,000 Monte Carlo simulations. Options: [a_VB_shc] Vaginal birth with standard hospital care; [b_VB_cmc] Vaginal birth with continuous midwifery care; [c_ICB_shc] Intrapartum cesarean birth with standard hospital care; [d_ICB_cmc] Intrapartum cesarean birth with continuous midwifery care; [e_PCB] Prelabor cesarean birth.

S1-15c. Overall values of options for physiological childbirth indicators with linear value functions and γ=0.2, scenario 3

Expert	Option	Mean overall value	5% quantile of overall value	95% quantile of overall value	Minimum	Maximum
E1	a_VB_shc	0.833	0.786	0.877	0.728	0.909
E1	b_VB_cmc	0.887	0.857	0.916	0.816	0.941
E1	c_ICB_shc	0.001	0.001	0.001	0.001	0.001
E1	d_ICB_cmc	0.001	0.001	0.001	0.001	0.001
E1	e_PCB	0	0	0	0	0
E4	a_VB_shc	0.796	0.744	0.844	0.664	0.888
E4	b_VB_cmc	0.854	0.818	0.888	0.772	0.923
E4	c_ICB_shc	0.002	0.002	0.002	0.002	0.002
E4	d_ICB_cmc	0.002	0.002	0.002	0.002	0.002
E4	e_PCB	0	0	0	0	0
E5	a_VB_shc	0.781	0.73	0.831	0.666	0.869
E5	b_VB_cmc	0.84	0.803	0.875	0.759	0.912
E5	c_ICB_shc	0.008	0.007	0.008	0.007	0.009
E5	d_ICB_cmc	0.008	0.008	0.008	0.007	0.008
E5	e_PCB	0	0	0	0	0
E_pooled	a_VB_shc	0.803	0.755	0.848	0.687	0.889
E_pooled	b_VB_cmc	0.86	0.827	0.892	0.785	0.925
E_pooled	c_ICB_shc	0.002	0.002	0.002	0.002	0.003
E_pooled	d_ICB_cmc	0.002	0.002	0.002	0.002	0.002
E_pooled	e_PCB	0	0	0	0	0

Note: Overall values for each option and each expert using linear value functions and a non-additive aggregation model with γ=0.2 for scenario 3: 0 (least physiological childbirth/"worst") to 1 (most physiological childbirth/"best"). Uncertainty in the predictions was accounted for by 2,000 Monte Carlo simulations. Options: [a_VB_shc] Vaginal birth with standard hospital care; [b_VB_cmc] Vaginal birth with continuous midwifery care; [c_ICB_shc] Intrapartum cesarean birth with standard hospital care; [d_ICB_cmc] Intrapartum cesarean birth with continuous midwifery care; [e_PCB] Prelabor cesarean birth.

S1-15d. Overall values of options for physiological childbirth indicators with linear value functions and γ=0.2, scenario 4

Expert	Option	Mean overall value	5% quantile of overall value	95% quantile of overall value	Minimum	Maximum
E1	a_VB_shc	0.803	0.753	0.849	0.7	0.888
E1	b_VB_cmc	0.833	0.824	0.843	0.806	0.854
E1	c_ICB_shc	0.001	0.001	0.001	0	0.001
E1	d_ICB_cmc	0.001	0.001	0.001	0.001	0.001
E1	e_PCB	0	0	0	0	0
E4	a_VB_shc	0.773	0.714	0.827	0.651	0.871
E4	b_VB_cmc	0.803	0.795	0.812	0.785	0.82
E4	c_ICB_shc	0.001	0.001	0.002	0.001	0.002
E4	d_ICB_cmc	0.002	0.001	0.002	0.001	0.002
E4	e_PCB	0	0	0	0	0
E5	a_VB_shc	0.759	0.703	0.811	0.636	0.851
E5	b_VB_cmc	0.787	0.778	0.797	0.766	0.806
E5	c_ICB_shc	0.007	0.006	0.007	0.006	0.007
E5	d_ICB_cmc	0.007	0.007	0.008	0.006	0.008
E5	e_PCB	0	0	0	0	0
E_pooled	a_VB_shc	0.778	0.724	0.827	0.662	0.862
E_pooled	b_VB_cmc	0.808	0.799	0.817	0.79	0.825
E_pooled	c_ICB_shc	0.002	0.002	0.002	0.002	0.002
E_pooled	d_ICB_cmc	0.002	0.002	0.002	0.002	0.002
E_pooled	e_PCB	0	0	0	0	0

Note: Overall values for each option and each expert using linear value functions and a non-additive aggregation model with γ=0.2 for scenario 4: 0 (least physiological childbirth/"worst") to 1 (most physiological childbirth/"best"). Uncertainty in the predictions was accounted for by 2,000 Monte Carlo simulations. Options: [a_VB_shc] Vaginal birth with standard hospital care; [b_VB_cmc] Vaginal birth with continuous midwifery care; [c_ICB_shc] Intrapartum cesarean birth with standard hospital care; [d_ICB_cmc] Intrapartum cesarean birth with continuous midwifery care; [e_PCB] Prelabor cesarean birth.

S1-16. Sensitivity analyses: Outputs of the weighted composite index of physiological childbirth indicators with expert value functions and γ=1

S1-16a. Overall values of options for physiological childbirth indicators with expert value functions and γ=1, scenario 1

Expert	Option	Mean overall value	5% quantile of overall value	95% quantile of overall value	Minimum	Maximum
E1	a_VB_shc	0.862	0.827	0.893	0.779	0.917
E1	b_VB_cmc	0.903	0.881	0.924	0.853	0.942
E1	c_ICB_shc	0.154	0.149	0.159	0.142	0.164
E1	d_ICB_cmc	0.162	0.157	0.167	0.151	0.173
E1	e_PCB	0	0	0	0	0
E4	a_VB_shc	0.8	0.751	0.845	0.7	0.89
E4	b_VB_cmc	0.856	0.821	0.89	0.769	0.921
E4	c_ICB_shc	0.18	0.168	0.192	0.15	0.208
E4	d_ICB_cmc	0.192	0.178	0.206	0.156	0.225
E4	e_PCB	0	0	0	0	0
E5	a_VB_shc	0.861	0.824	0.893	0.763	0.923
E5	b_VB_cmc	0.899	0.872	0.924	0.831	0.947
E5	c_ICB_shc	0.321	0.31	0.331	0.285	0.344
E5	d_ICB_cmc	0.329	0.318	0.341	0.286	0.357
E5	e_PCB	0	0	0	0	0
E_pooled	a_VB_shc	0.859	0.824	0.892	0.778	0.916
E_pooled	b_VB_cmc	0.9	0.874	0.923	0.837	0.946
E_pooled	c_ICB_shc	0.231	0.22	0.24	0.203	0.25
E_pooled	d_ICB_cmc	0.241	0.231	0.251	0.209	0.263
E_pooled	e_PCB	0	0	0	0	0

Note: Overall values for each option and each expert using elicited expert value functions and an additive aggregation model with γ=1 for scenario 1: 0 (least physiological childbirth/"worst") to 1 (most physiological childbirth/"best"). Uncertainty in the predictions was accounted for by 2,000 Monte Carlo simulations. Options: [a_VB_shc] Vaginal birth with standard hospital care; [b_VB_cmc] Vaginal birth with continuous midwifery care; [c_ICB_shc] Intrapartum cesarean birth with standard hospital care; [d_ICB_cmc] Intrapartum cesarean birth with continuous midwifery care; [e_PCB] Prelabor cesarean birth.

S1-16b. Overall values of options for physiological childbirth indicators with expert value functions and γ=1, scenario 2

Expert	Option	Mean overall value	5% quantile of overall value	95% quantile of overall value	Minimum	Maximum
E1	a_VB_shc	0.802	0.769	0.834	0.727	0.857
E1	b_VB_cmc	0.85	0.829	0.871	0.789	0.888
E1	c_ICB_shc	0.094	0.089	0.099	0.086	0.104
E1	d_ICB_cmc	0.1	0.097	0.104	0.093	0.108
E1	e_PCB	0	0	0	0	0
E4	a_VB_shc	0.743	0.694	0.791	0.646	0.827
E4	b_VB_cmc	0.804	0.767	0.84	0.717	0.878
E4	c_ICB_shc	0.13	0.118	0.142	0.108	0.156
E4	d_ICB_cmc	0.136	0.122	0.151	0.108	0.166
E4	e_PCB	0	0	0	0	0
E5	a_VB_shc	0.822	0.785	0.856	0.721	0.883
E5	b_VB_cmc	0.868	0.838	0.894	0.786	0.919
E5	c_ICB_shc	0.275	0.265	0.286	0.247	0.298
E5	d_ICB_cmc	0.281	0.268	0.294	0.244	0.308
E5	e_PCB	0	0	0	0	0
E_pooled	a_VB_shc	0.81	0.775	0.844	0.736	0.872
E_pooled	b_VB_cmc	0.859	0.832	0.883	0.789	0.907
E_pooled	c_ICB_shc	0.178	0.167	0.187	0.157	0.197
E_pooled	d_ICB_cmc	0.185	0.173	0.196	0.159	0.206
E_pooled	e_PCB	0	0	0	0	0

Note: Overall values for each option and each expert using elicited expert value functions and an additive aggregation model with γ=1 for scenario 2: 0 (least physiological childbirth/"worst") to 1 (most physiological childbirth/"best"). Uncertainty in the predictions was accounted for by 2,000 Monte Carlo simulations. Options: [a_VB_shc] Vaginal birth with standard hospital care; [b_VB_cmc] Vaginal birth with continuous midwifery care; [c_ICB_shc] Intrapartum cesarean birth with standard hospital care; [d_ICB_cmc] Intrapartum cesarean birth with continuous midwifery care; [e_PCB] Prelabor cesarean birth.

S1-16c. Overall values of options for physiological childbirth indicators with expert value functions and γ=1, scenario 3

Expert	Option	Mean overall value	5% quantile of overall value	95% quantile of overall value	Minimum	Maximum
E1	a_VB_shc	0.873	0.84	0.903	0.798	0.927
E1	b_VB_cmc	0.912	0.891	0.933	0.863	0.951
E1	c_ICB_shc	0.181	0.176	0.185	0.171	0.192
E1	d_ICB_cmc	0.186	0.185	0.187	0.184	0.188
E1	e_PCB	0	0	0	0	0
E4	a_VB_shc	0.81	0.767	0.852	0.714	0.891
E4	b_VB_cmc	0.861	0.829	0.892	0.788	0.925
E4	c_ICB_shc	0.212	0.201	0.222	0.191	0.236
E4	d_ICB_cmc	0.216	0.211	0.22	0.206	0.225
E4	e_PCB	0	0	0	0	0
E5	a_VB_shc	0.869	0.838	0.9	0.798	0.924
E5	b_VB_cmc	0.904	0.882	0.926	0.856	0.948
E5	c_ICB_shc	0.343	0.334	0.352	0.325	0.363
E5	d_ICB_cmc	0.345	0.341	0.349	0.337	0.353
E5	e_PCB	0	0	0	0	0
E_pooled	a_VB_shc	0.868	0.836	0.898	0.795	0.925
E_pooled	b_VB_cmc	0.905	0.883	0.926	0.855	0.949
E_pooled	c_ICB_shc	0.256	0.249	0.263	0.24	0.273
E_pooled	d_ICB_cmc	0.259	0.256	0.262	0.253	0.265
E_pooled	e_PCB	0	0	0	0	0

Note: Overall values for each option and each expert using elicited expert value functions and an additive aggregation model with γ=1 for scenario 3: 0 (least physiological childbirth/"worst") to 1 (most physiological childbirth/"best"). Uncertainty in the predictions was accounted for by 2,000 Monte Carlo simulations. Options: [a_VB_shc] Vaginal birth with standard hospital care; [b_VB_cmc] Vaginal birth with continuous midwifery care; [c_ICB_shc] Intrapartum cesarean birth with standard hospital care; [d_ICB_cmc] Intrapartum cesarean birth with continuous midwifery care; [e_PCB] Prelabor cesarean birth.

S1-16d. Overall values of options for physiological childbirth indicators with expert value functions and γ=1, scenario 4

Expert	Option	Mean overall value	5% quantile of overall value	95% quantile of overall value	Minimum	Maximum
E1	a_VB_shc	0.848	0.813	0.881	0.771	0.91
E1	b_VB_cmc	0.87	0.863	0.876	0.852	0.885
E1	c_ICB_shc	0.146	0.141	0.15	0.135	0.156
E1	d_ICB_cmc	0.154	0.15	0.158	0.147	0.163
E1	e_PCB	0	0	0	0	0
E4	a_VB_shc	0.789	0.74	0.837	0.681	0.879
E4	b_VB_cmc	0.811	0.803	0.819	0.794	0.828
E4	c_ICB_shc	0.185	0.174	0.195	0.162	0.205
E4	d_ICB_cmc	0.196	0.185	0.208	0.173	0.219
E4	e_PCB	0	0	0	0	0
E5	a_VB_shc	0.86	0.826	0.892	0.782	0.917
E5	b_VB_cmc	0.874	0.868	0.88	0.862	0.885
E5	c_ICB_shc	0.327	0.319	0.336	0.308	0.344
E5	d_ICB_cmc	0.336	0.326	0.346	0.315	0.356
E5	e_PCB	0	0	0	0	0
E_pooled	a_VB_shc	0.854	0.819	0.887	0.773	0.911
E_pooled	b_VB_cmc	0.871	0.865	0.877	0.859	0.882
E_pooled	c_ICB_shc	0.231	0.224	0.238	0.214	0.246
E_pooled	d_ICB_cmc	0.241	0.233	0.248	0.225	0.256
E_pooled	e_PCB	0	0	0	0	0

Note: Overall values for each option and each expert using elicited expert value functions and an additive aggregation model with γ=1 for scenario 4: 0 (least physiological childbirth/"worst") to 1 (most physiological childbirth/"best"). Uncertainty in the predictions was accounted for by 2,000 Monte Carlo simulations. Options: [a_VB_shc] Vaginal birth with standard hospital care; [b_VB_cmc] Vaginal birth with continuous midwifery care; [c_ICB_shc] Intrapartum cesarean birth with standard hospital care; [d_ICB_cmc] Intrapartum cesarean birth with continuous midwifery care; [e_PCB] Prelabor cesarean birth.

S1-17. Clinical indicator scoring system for resource use in intrapartum care, inspired by (10)

Indicator	Description	Score
Gestational age	≥ 37+0 weeks	1
	34+0 - 36+6 weeks	2
	≤ 33+6 weeks	3
Multiple pregnancy	Twins	2
	≥ Triplets	5
Medical condition	Condition requiring medical oversight[a]	5
Labor interventions	Induction of labor	2
	Augmentation of labor	2
	Intrapartum epidural/spinal anesthesia	3
Childbirth mode	Spontaneous vaginal birth	1
	Instrumental vaginal birth, breech vaginal birth	2
	Elective/prelabor cesarean birth	3
	Emergency/intrapartum cesarean birth	5
Perineal/vaginal/cervical tear[b]	Intact perineum	1
	Perineal tear 1st/2nd degree, episiotomy, vaginal tear	2
	Perineal tear 3rd/4th degree (obstetric anal sphincter injury), cervical tear, other obstetric birth canal trauma	3
Postpartum hemorrhage	Blood loss > 500ml after vaginal birth or > 1000ml after cesarean birth	2
Apgar score at 5 minutes[c]	≥ 8	1
	5 - 7	2
	< 5	3
Birth weight[c]	Birth weight ≥ 2500g	1
	Birth weight 1500g - 2499g	2
	Birth weight < 1500g	3
Congenital anomaly	Congenital anomaly	3
Perinatal death	Infant is stillborn or dies in the early postnatal period	5
Emergency procedures	Blood transfusion	5
	Emergency procedure in the 3rd stage of labor or immediately postpartum[d]	5
Intensive care needs	Maternal intensive care unit admission	5
	Neonatal intensive care unit admission	5

Note: The minimum score attainable for women who met the study's inclusion and exclusion criteria was 5, and the maximum score was 54. [a] Diabetes mellitus, hypertensive disorder, respiratory, uro-/nephrological, neurological, coagulation disorder, acute mental health condition, alcohol/illicit drug problem; Score regardless of number of conditions. [b] Must also be assessed for caesarean births. [c] For multiple births, each baby must be assessed. [d] General anesthesia in 3rd stage of labor, procedure due to retained placenta (tissue), surgical revision of the birth canal, hysterectomy after vaginal birth; Score regardless of number of interventions.

S1-18. Characteristics of experts who provided performance estimates for the goals on psychosocial care experience and physical strain for care providers

Characteristic [m]	Statistic	N = 5
Age in years [0]	range (mean; median)	34.00-40.00 (37.40; 38.00)
Self-reported female gender [0]	n (%)	5 (100.00)
Number of children [0]	range (mean; median)	0.00-2.00 (1.20; 1.00)
Self-reported obesity [0]	n (%)	1 (20.00)
Professional experience in years [0]	range (mean; median)	9.00-17.00 (12.00; 11.00)
Number of clients with obesity per year [1]	range (mean; median)	10.00-180.00 (60.00; 25.00)
Self-reported field of expertise[a]		Advanced midwifery practice<br>Applied continuous midwifery care<br>Choice of birth<br>Maternal obesity<br>Medical obstetrics<br>Peripartum mental health
Self-reported level of uncertainty in estimating the psychosocial experience of care[b] [0]	range (mean; median)	25-50% (40%; 50%)
Self-reported level of uncertainty in estimating the physical strain for care providers[b] [0]	range (mean; median)	25-75% (45%; 50%)

Note: [m]: number of missing values; n: number of observations; %: percentage. [a] Multiple answers possible. [b] "extremely uncertain" = 95% uncertain; "highly uncertain" = 75% uncertain; "fairly (un)certain" = 50% (un)certain; "highly certain" = 25% uncertain; "extremely certain" = 5% uncertain (11, 12).

S1-19. Characteristics of real stakeholders who provided value functions and weights for the main MCDA

Characteristic [m] / Question	Statistic	N = 3
Age in years [0]	set	35, 40, 43
Self-reported female gender [0]	n (%)	3 (100)
Children [0]	n (%)	1 (33)
Self-reported obesity [0]	n (%)	2 (67)
Professional experience in years[a] [0]	set	16, 17
Stakeholder role (in random order) [0]		Women with BMI of 34.5 kg/m^2<br>Specialist midwife<br>Leading senior obstetrician
How much influence did you have in making the decision about your own childbirth care?[b,*] [0]	Answer scale	Woman with BMI of 34.5 kg/m^2: 4=substantial influence
How much influence do you have in making the decision about childbirth care for women with obesity?[a,*] [0]	Answer scale	Specialist midwife: 2=rather small influence<br>Leading senior obstetrician: 3=rather large influence
To what extent does the decision regarding childbirth care for women with obesity affect you personally?[a,**] [0]	Answer scale	Woman with BMI of 34.5 kg/m^2: 4=substantially affected<br>Specialist midwife: 2=rather slightly affected<br>Leading senior obstetrician: 3=rather substantially affected

Note: [m]: number of missing values; n: number of observations; %: percentage. [a] Care providers only. [b] Woman with obesity only. Answer scales: * 0=no influence; 1=very small influence; 2=rather small influence; 3=rather large influence; 4=substantial influence. ** 0=not affected; 1=very slightly affected; 2=rather slightly affected; 3=rather substantially affected; 4=substantially affected.

S2. MCDA inputs

S2-1. Options' goal performance predictions

S2-1a. Prediction matrix, scenario 1

Goal	Attribute	Unit	Option	Prediction	Minimum[a]	Maximum[a]	Uncertainty distribution	Uncertainty distribution parameter 1[b]	Uncertainty distribution parameter 2[c]	Uncertainty distribution parameter 3[d]
A1. Low maternal complication rates	Weighted composite index	Value	a_VB_shc	0.816	0.3	1	normal	0.816	0.045	NA
A1. Low maternal complication rates	Weighted composite index	Value	b_VB_cmc	0.841	0.3	1	normal	0.841	0.033	NA
A1. Low maternal complication rates	Weighted composite index	Value	c_ICB_shc	0.914	0.3	1	normal	0.914	0.015	NA
A1. Low maternal complication rates	Weighted composite index	Value	d_ICB_cmc	0.912	0.3	1	normal	0.912	0.023	NA
A1. Low maternal complication rates	Weighted composite index	Value	e_PCB	0.942	0.3	1	normal	0.942	0.013	NA
A2. Low neonatal complication rates	Weighted composite index	Value	a_VB_shc	0.860	0.3	1	normal	0.860	0.018	NA
A2. Low neonatal complication rates	Weighted composite index	Value	b_VB_cmc	0.847	0.3	1	normal	0.847	0.030	NA
A2. Low neonatal complication rates	Weighted composite index	Value	c_ICB_shc	0.885	0.3	1	normal	0.885	0.011	NA
A2. Low neonatal complication rates	Weighted composite index	Value	d_ICB_cmc	0.931	0.3	1	normal	0.931	0.010	NA
A2. Low neonatal complication rates	Weighted composite index	Value	e_PCB	0.912	0.3	1	normal	0.912	0.008	NA
B1. Physiological labor and childbirth processes	Weighted composite index	Value	a_VB_shc	0.852	0	1	normal	0.852	0.019	NA
B1. Physiological labor and childbirth processes	Weighted composite index	Value	b_VB_cmc	0.896	0	1	normal	0.896	0.014	NA
B1. Physiological labor and childbirth processes	Weighted composite index	Value	c_ICB_shc	0.002	0	1	normal	0.002	0.000	NA
B1. Physiological labor and childbirth processes	Weighted composite index	Value	d_ICB_cmc	0.002	0	1	normal	0.002	0.000	NA
B1. Physiological labor and childbirth processes	Weighted composite index	Value	e_PCB	0.000	0	1	normal	0.000	0.000	NA
B2. Positive initiation of breastfeeding after childbirth	Exclusive breastfeeding	%	a_VB_shc	85.248	50	95	normal	85.248	2.469	NA
B2. Positive initiation of breastfeeding after childbirth	Exclusive breastfeeding	%	b_VB_cmc	90.317	50	95	normal	90.317	1.152	NA
B2. Positive initiation of breastfeeding after childbirth	Exclusive breastfeeding	%	c_ICB_shc	77.925	50	95	normal	77.925	3.488	NA
B2. Positive initiation of breastfeeding after childbirth	Exclusive breastfeeding	%	d_ICB_cmc	78.942	50	95	normal	78.942	2.058	NA
B2. Positive initiation of breastfeeding after childbirth	Exclusive breastfeeding	%	e_PCB	75.424	50	95	normal	75.424	3.578	NA
C1. Positive psychosocial experience of care	Expert assessment	Scale	a_VB_shc	60	0	100	triangular	0	95	60
C1. Positive psychosocial experience of care	Expert assessment	Scale	b_VB_cmc	80	0	100	triangular	10	100	80
C1. Positive psychosocial experience of care	Expert assessment	Scale	c_ICB_shc	45	0	100	triangular	0	85	45
C1. Positive psychosocial experience of care	Expert assessment	Scale	d_ICB_cmc	65	0	100	triangular	5	95	65
C1. Positive psychosocial experience of care	Expert assessment	Scale	e_PCB	60	0	100	triangular	5	100	60
D1. Low physical strain for care providers	Expert assessment	Scale	a_VB_shc	5	1	10	triangular	2	9	5
D1. Low physical strain for care providers	Expert assessment	Scale	b_VB_cmc	6	1	10	triangular	2	10	6
D1. Low physical strain for care providers	Expert assessment	Scale	c_ICB_shc	6	1	10	triangular	3	10	6
D1. Low physical strain for care providers	Expert assessment	Scale	d_ICB_cmc	7	1	10	triangular	3	10	7
D1. Low physical strain for care providers	Expert assessment	Scale	e_PCB	5	1	10	triangular	1	9	5

E1. Low resource use in care setting	Clinical indicator score	Score	a_VB_shc	9.050	5	36	normal	9.050	0.744	NA
E1. Low resource use in care setting	Clinical indicator score	Score	b_VB_cmc	8.060	5	36	normal	8.060	0.750	NA
E1. Low resource use in care setting	Clinical indicator score	Score	c_ICB_shc	13.853	5	36	normal	13.853	0.443	NA
E1. Low resource use in care setting	Clinical indicator score	Score	d_ICB_cmc	14.068	5	36	normal	14.068	0.529	NA
E1. Low resource use in care setting	Clinical indicator score	Score	e_PCB	7.437	5	36	normal	7.437	0.611	NA
F1. Low direct costs to the healthcare system	SwissDRG cost weight	Cost weight	a_VB_shc	0.582	0.553	1.405	normal	0.582	0.012	NA
F1. Low direct costs to the healthcare system	SwissDRG cost weight	Cost weight	b_VB_cmc	0.586	0.553	1.405	normal	0.586	0.012	NA
F1. Low direct costs to the healthcare system	SwissDRG cost weight	Cost weight	c_ICB_shc	1.046	0.553	1.405	normal	1.046	0.007	NA
F1. Low direct costs to the healthcare system	SwissDRG cost weight	Cost weight	d_ICB_cmc	1.047	0.553	1.405	normal	1.047	0.008	NA
F1. Low direct costs to the healthcare system	SwissDRG cost weight	Cost weight	e_PCB	0.817	0.553	1.405	normal	0.817	0.010	NA

Note: Adapted from the ValueDecisions' predictions template (9). Options: [a_VB_shc] Vaginal birth with standard hospital care; [b_VB_cmc] Vaginal birth with continuous midwifery care; [c_ICB_shc] Intrapartum cesarean birth with standard hospital care; [d_ICB_cmc] Intrapartum cesarean birth with continuous midwifery care; [e_PCB] Prelabor cesarean birth; NA: not applicable. [a] Minimum/maximum across all options. [b] Normal distribution: expectation value (mean, average); Triangular distribution: minimum. [c] Normal distribution: standard deviation; Triangular distribution: maximum. [d] Normal distribution: not applicable; Triangular distribution: mode.

S2-1b. Performance predictions with uncertainty, scenario 1

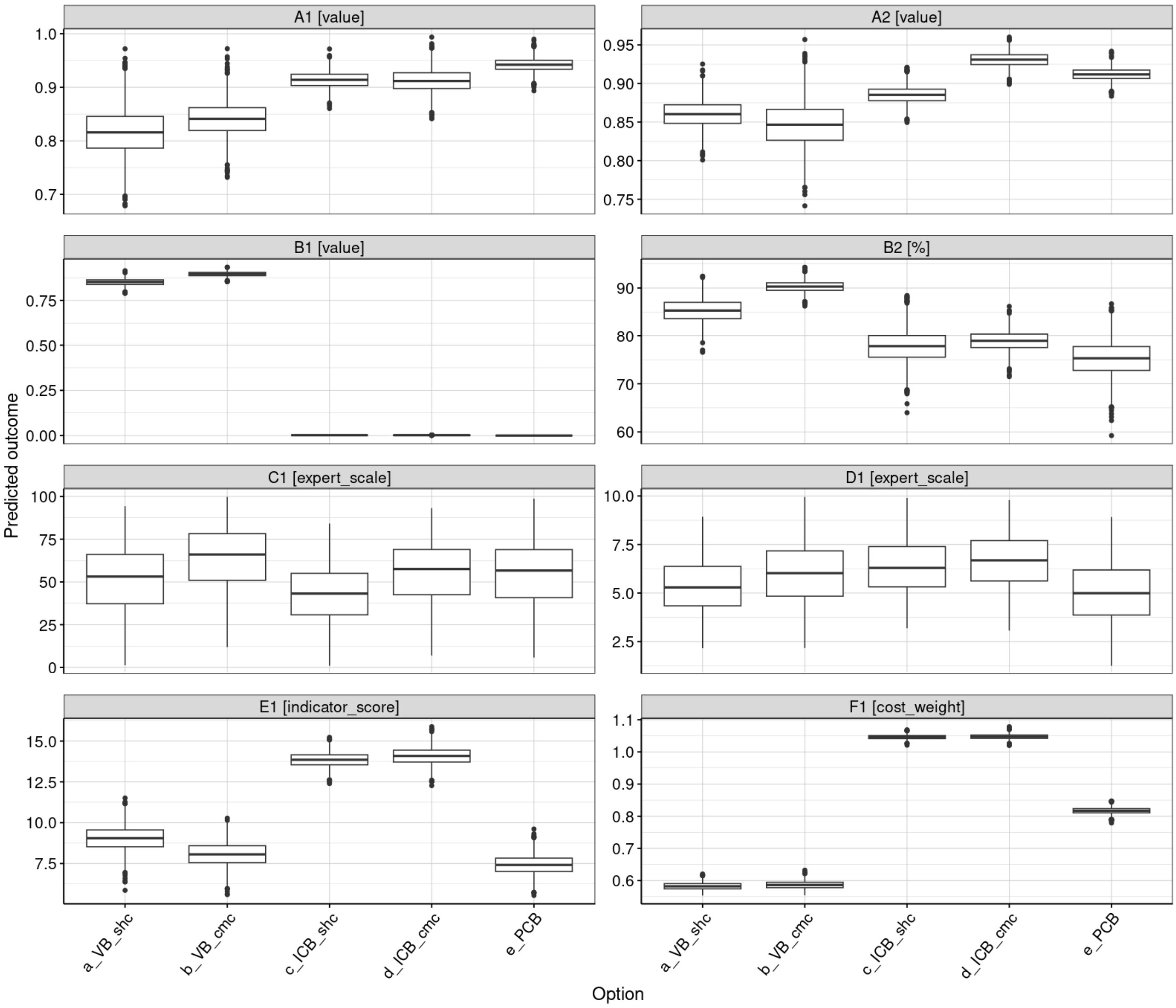

Note: Graph by ValueDecisions (9). Performance predictions for scenario 1. Uncertainty in the predictions was accounted for by 2,000 Monte Carlo simulations. Goals/attributes (units): A1: Low maternal complication rates/weighted composite index (value); A2: Low neonatal complication rates/weighted composite index (value); B1: Physiological labor and childbirth processes/weighted composite index (value); B2: Positive initiation of bonding and breastfeeding after childbirth/exclusive breastfeeding at hospital discharge (%); C1: Positive psychosocial experience of care interactions and events/expert assessment (scale); D1: Low physical strain for care providers/expert assessment (scale); E1: Low resource use in care setting/clinical indicator score (score); F1: Low direct costs to the healthcare system/SwissDRG cost weight (cost weight). Options: [a_VB_shc] Vaginal birth with standard hospital care; [b_VB_cmc] Vaginal birth with continuous midwifery care; [c_ICB_shc] Intrapartum cesarean birth with standard hospital care; [d_ICB_cmc] Intrapartum cesarean birth with continuous midwifery care; [e_PCB] Prelabor cesarean birth.

S2-1c. Prediction matrix, scenario 2

Goal	Attribute	Unit	Option	Prediction	Minimum[a]	Maximum[a]	Uncertainty distribution	Uncertainty distribution parameter 1[b]	Uncertainty distribution parameter 2[c]	Uncertainty distribution parameter 3[d]
A1. Low maternal complication rates	Weighted composite index	Value	a_VB_shc	0.767	0.3	1	normal	0.767	0.057	NA
A1. Low maternal complication rates	Weighted composite index	Value	b_VB_cmc	0.797	0.3	1	normal	0.797	0.041	NA
A1. Low maternal complication rates	Weighted composite index	Value	c_ICB_shc	0.894	0.3	1	normal	0.894	0.021	NA
A1. Low maternal complication rates	Weighted composite index	Value	d_ICB_cmc	0.874	0.3	1	normal	0.874	0.040	NA
A1. Low maternal complication rates	Weighted composite index	Value	e_PCB	0.924	0.3	1	normal	0.924	0.020	NA
A2. Low neonatal complication rates	Weighted composite index	Value	a_VB_shc	0.813	0.3	1	normal	0.813	0.025	NA
A2. Low neonatal complication rates	Weighted composite index	Value	b_VB_cmc	0.788	0.3	1	normal	0.788	0.051	NA
A2. Low neonatal complication rates	Weighted composite index	Value	c_ICB_shc	0.817	0.3	1	normal	0.817	0.020	NA
A2. Low neonatal complication rates	Weighted composite index	Value	d_ICB_cmc	0.903	0.3	1	normal	0.903	0.014	NA
A2. Low neonatal complication rates	Weighted composite index	Value	e_PCB	0.849	0.3	1	normal	0.849	0.018	NA
B1. Physiological labor and childbirth processes	Weighted composite index	Value	a_VB_shc	0.795	0	1	normal	0.795	0.019	NA
B1. Physiological labor and childbirth processes	Weighted composite index	Value	b_VB_cmc	0.850	0	1	normal	0.850	0.014	NA
B1. Physiological labor and childbirth processes	Weighted composite index	Value	c_ICB_shc	0.002	0	1	normal	0.002	0.000	NA
B1. Physiological labor and childbirth processes	Weighted composite index	Value	d_ICB_cmc	0.002	0	1	normal	0.002	0.000	NA
B1. Physiological labor and childbirth processes	Weighted composite index	Value	e_PCB	0	0	1	normal	0	0.000	NA
B2. Positive initiation of breastfeeding after childbirth	Exclusive breastfeeding	%	a_VB_shc	80.961	50	95	normal	80.961	3.093	NA
B2. Positive initiation of breastfeeding after childbirth	Exclusive breastfeeding	%	b_VB_cmc	87.321	50	95	normal	87.321	1.656	NA
B2. Positive initiation of breastfeeding after childbirth	Exclusive breastfeeding	%	c_ICB_shc	72.392	50	95	normal	72.392	4.051	NA
B2. Positive initiation of breastfeeding after childbirth	Exclusive breastfeeding	%	d_ICB_cmc	74.113	50	95	normal	74.113	3.526	NA
B2. Positive initiation of breastfeeding after childbirth	Exclusive breastfeeding	%	e_PCB	69.440	50	95	normal	69.440	4.216	NA
C1. Positive psychosocial experience of care	Expert assessment	Scale	a_VB_shc	60	0	100	triangular	0	95	60
C1. Positive psychosocial experience of care	Expert assessment	Scale	b_VB_cmc	80	0	100	triangular	10	100	80
C1. Positive psychosocial experience of care	Expert assessment	Scale	c_ICB_shc	45	0	100	triangular	0	85	45
C1. Positive psychosocial experience of care	Expert assessment	Scale	d_ICB_cmc	65	0	100	triangular	5	95	65
C1. Positive psychosocial experience of care	Expert assessment	Scale	e_PCB	60	0	100	triangular	5	100	60
D1. Low physical strain for care providers	Expert assessment	Scale	a_VB_shc	5	1	10	triangular	2	9	5
D1. Low physical strain for care providers	Expert assessment	Scale	b_VB_cmc	6	1	10	triangular	2	10	6
D1. Low physical strain for care providers	Expert assessment	Scale	c_ICB_shc	6	1	10	triangular	3	10	6
D1. Low physical strain for care providers	Expert assessment	Scale	d_ICB_cmc	7	1	10	triangular	3	10	7
D1. Low physical strain for care providers	Expert assessment	Scale	e_PCB	5	1	10	triangular	1	9	5
E1. Low resource use in care setting	Clinical indicator score	Score	a_VB_shc	14.656	5	36	normal	14.656	0.798	NA
E1. Low resource use in care setting	Clinical indicator score	Score	b_VB_cmc	13.724	5	36	normal	13.724	0.738	NA
E1. Low resource use in care setting	Clinical indicator score	Score	c_ICB_shc	19.471	5	36	normal	19.471	0.427	NA
E1. Low resource use in care setting	Clinical indicator score	Score	d_ICB_cmc	19.746	5	36	normal	19.746	0.353	NA
E1. Low resource use in care setting	Clinical indicator score	Score	e_PCB	13.120	5	36	normal	13.120	0.674	NA

F1. Low direct costs to the healthcare system	SwissDRG cost weight	Cost weight	a_VB_shc	0.622	0.553	1.405	normal	0.622	0.013	NA
F1. Low direct costs to the healthcare system	SwissDRG cost weight	Cost weight	b_VB_cmc	0.626	0.553	1.405	normal	0.626	0.012	NA
F1. Low direct costs to the healthcare system	SwissDRG cost weight	Cost weight	c_ICB_shc	1.086	0.553	1.405	normal	1.086	0.007	NA
F1. Low direct costs to the healthcare system	SwissDRG cost weight	Cost weight	d_ICB_cmc	1.088	0.553	1.405	normal	1.088	0.007	NA
F1. Low direct costs to the healthcare system	SwissDRG cost weight	Cost weight	e_PCB	0.859	0.553	1.405	normal	0.859	0.011	NA

Note: Adapted from the ValueDecisions' predictions template (9). Options: [a_VB_shc] Vaginal birth with standard hospital care; [b_VB_cmc] Vaginal birth with continuous midwifery care; [c_ICB_shc] Intrapartum cesarean birth with standard hospital care; [d_ICB_cmc] Intrapartum cesarean birth with continuous midwifery care; [e_PCB] Prelabor cesarean birth; NA: not applicable. [a] Minimum/maximum across all options. [b] Normal distribution: expectation value (mean, average); Triangular distribution: minimum. [c] Normal distribution: standard deviation; Triangular distribution: maximum. [d] Normal distribution: not applicable; Triangular distribution: mode.

S2-1d. Performance predictions with uncertainty, scenario 2

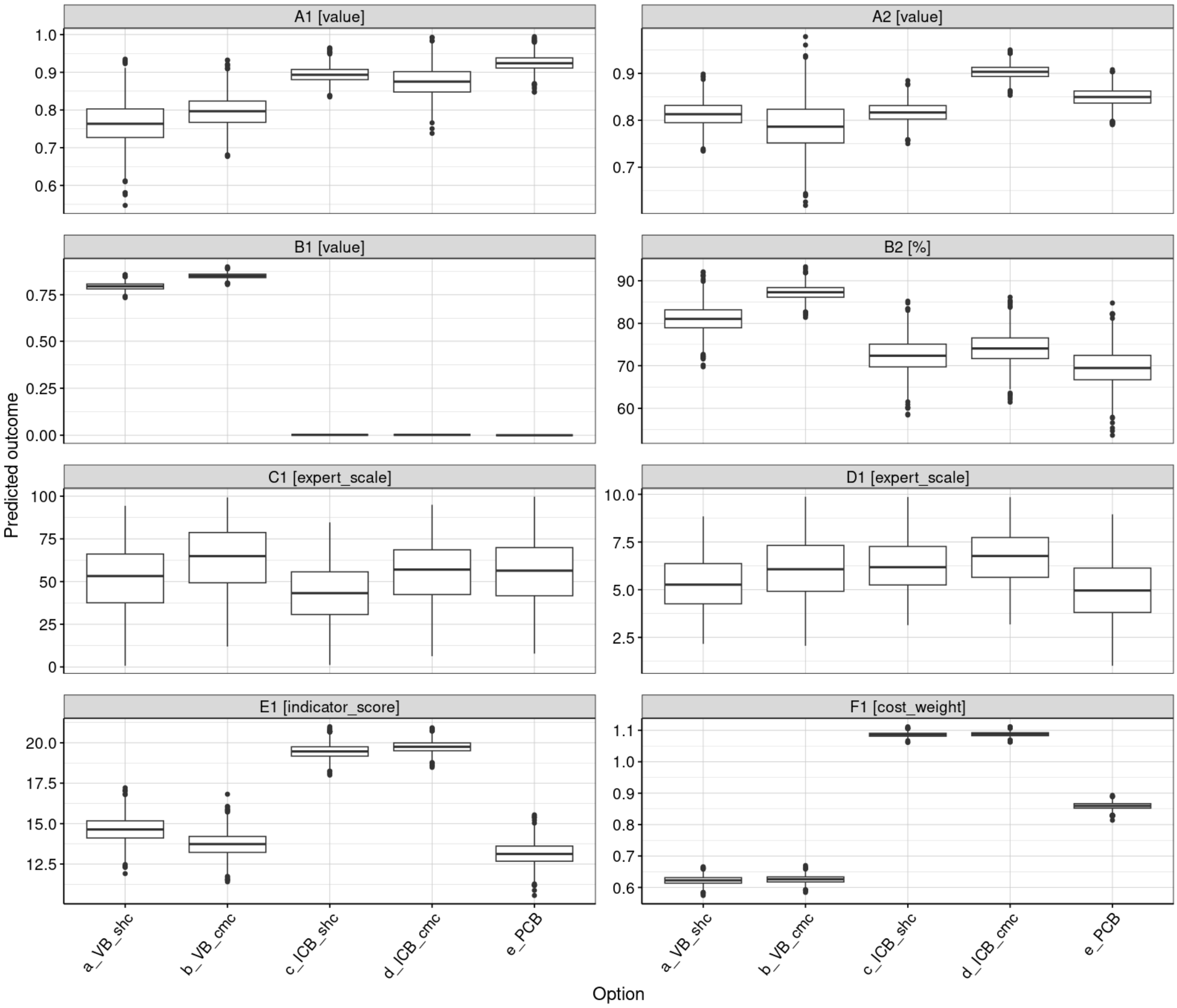

Note: Graph by ValueDecisions (9). Performance predictions for scenario 2. Uncertainty in the predictions was accounted for by 2,000 Monte Carlo simulations. Goals/attributes (units): A1: Low maternal complication rates/weighted composite index (value); A2: Low neonatal complication rates/weighted composite index (value); B1: Physiological labor and childbirth processes/weighted composite index (value); B2: Positive initiation of bonding and breastfeeding after childbirth/exclusive breastfeeding at hospital discharge (%); C1: Positive psychosocial experience of care interactions and events/expert assessment (scale); D1: Low physical strain for care providers/expert assessment (scale); E1: Low resource use in care setting/clinical indicator score (score); F1: Low direct costs to the healthcare system/SwissDRG cost weight (cost weight). Options: [a_VB_shc] Vaginal birth with standard hospital care; [b_VB_cmc] Vaginal birth with continuous midwifery care; [c_ICB_shc] Intrapartum cesarean birth with standard hospital care; [d_ICB_cmc] Intrapartum cesarean birth with continuous midwifery care; [e_PCB] Prelabor cesarean birth.

S2-1e. Prediction matrix, scenario 3

Goal	Attribute	Unit	Option	Prediction	Minimum[a]	Maximum[a]	Uncertainty distribution	Uncertainty distribution parameter 1[b]	Uncertainty distribution parameter 2[c]	Uncertainty distribution parameter 3[d]
A1. Low maternal complication rates	Weighted composite index	Value	a_VB_shc	0.819	0.3	1	normal	0.819	0.049	NA
A1. Low maternal complication rates	Weighted composite index	Value	b_VB_cmc	0.855	0.3	1	normal	0.855	0.026	NA
A1. Low maternal complication rates	Weighted composite index	Value	c_ICB_shc	0.926	0.3	1	normal	0.926	0.015	NA
A1. Low maternal complication rates	Weighted composite index	Value	d_ICB_cmc	0.926	0.3	1	normal	0.926	0.016	NA
A1. Low maternal complication rates	Weighted composite index	Value	e_PCB	0.945	0.3	1	normal	0.945	0.015	NA
A2. Low neonatal complication rates	Weighted composite index	Value	a_VB_shc	0.875	0.3	1	normal	0.875	0.015	NA
A2. Low neonatal complication rates	Weighted composite index	Value	b_VB_cmc	0.854	0.3	1	normal	0.854	0.026	NA
A2. Low neonatal complication rates	Weighted composite index	Value	c_ICB_shc	0.881	0.3	1	normal	0.881	0.013	NA
A2. Low neonatal complication rates	Weighted composite index	Value	d_ICB_cmc	0.929	0.3	1	normal	0.929	0.009	NA
A2. Low neonatal complication rates	Weighted composite index	Value	e_PCB	0.907	0.3	1	normal	0.907	0.009	NA
B1. Physiological labor and childbirth processes	Weighted composite index	Value	a_VB_shc	0.860	0	1	normal	0.860	0.018	NA
B1. Physiological labor and childbirth processes	Weighted composite index	Value	b_VB_cmc	0.901	0	1	normal	0.901	0.012	NA
B1. Physiological labor and childbirth processes	Weighted composite index	Value	c_ICB_shc	0.003	0	1	normal	0.003	0.000	NA
B1. Physiological labor and childbirth processes	Weighted composite index	Value	d_ICB_cmc	0.003	0	1	normal	0.003	0.000	NA
B1. Physiological labor and childbirth processes	Weighted composite index	Value	e_PCB	0	0	1	normal	0	0.000	NA
B2. Positive initiation of breastfeeding after childbirth	Exclusive breastfeeding	%	a_VB_shc	86.159	50	95	normal	86.159	2.278	NA
B2. Positive initiation of breastfeeding after childbirth	Exclusive breastfeeding	%	b_VB_cmc	90.854	50	95	normal	90.854	0.560	NA
B2. Positive initiation of breastfeeding after childbirth	Exclusive breastfeeding	%	c_ICB_shc	79.253	50	95	normal	79.253	3.197	NA
B2. Positive initiation of breastfeeding after childbirth	Exclusive breastfeeding	%	d_ICB_cmc	80.902	50	95	normal	80.902	1.048	NA
B2. Positive initiation of breastfeeding after childbirth	Exclusive breastfeeding	%	e_PCB	76.926	50	95	normal	76.926	3.408	NA
C1. Positive psychosocial experience of care	Expert assessment	Scale	a_VB_shc	60	0	100	triangular	0	95	60
C1. Positive psychosocial experience of care	Expert assessment	Scale	b_VB_cmc	80	0	100	triangular	10	100	80
C1. Positive psychosocial experience of care	Expert assessment	Scale	c_ICB_shc	45	0	100	triangular	0	85	45
C1. Positive psychosocial experience of care	Expert assessment	Scale	d_ICB_cmc	65	0	100	triangular	5	95	65
C1. Positive psychosocial experience of care	Expert assessment	Scale	e_PCB	60	0	100	triangular	5	100	60
D1. Low physical strain for care providers	Expert assessment	Scale	a_VB_shc	5	1	10	triangular	2	9	5
D1. Low physical strain for care providers	Expert assessment	Scale	b_VB_cmc	6	1	10	triangular	2	10	6
D1. Low physical strain for care providers	Expert assessment	Scale	c_ICB_shc	6	1	10	triangular	3	10	6
D1. Low physical strain for care providers	Expert assessment	Scale	d_ICB_cmc	7	1	10	triangular	3	10	7
D1. Low physical strain for care providers	Expert assessment	Scale	e_PCB	5	1	10	triangular	1	9	5
E1. Low resource use in care setting	Clinical indicator score	Score	a_VB_shc	9.064	5	36	normal	9.064	0.804	NA
E1. Low resource use in care setting	Clinical indicator score	Score	b_VB_cmc	8.253	5	36	normal	8.253	0.554	NA
E1. Low resource use in care setting	Clinical indicator score	Score	c_ICB_shc	13.670	5	36	normal	13.670	0.431	NA
E1. Low resource use in care setting	Clinical indicator score	Score	d_ICB_cmc	14.156	5	36	normal	14.156	0.097	NA
E1. Low resource use in care setting	Clinical indicator score	Score	e_PCB	7.356	5	36	normal	7.356	0.534	NA

F1. Low direct costs to the healthcare system	SwissDRG cost weight	Cost weight	a_VB_shc	0.570	0.553	1.405	normal	0.570	0.013	NA
F1. Low direct costs to the healthcare system	SwissDRG cost weight	Cost weight	b_VB_cmc	0.576	0.553	1.405	normal	0.576	0.008	NA
F1. Low direct costs to the healthcare system	SwissDRG cost weight	Cost weight	c_ICB_shc	1.031	0.553	1.405	normal	1.031	0.007	NA
F1. Low direct costs to the healthcare system	SwissDRG cost weight	Cost weight	d_ICB_cmc	1.036	0.553	1.405	normal	1.036	0.002	NA
F1. Low direct costs to the healthcare system	SwissDRG cost weight	Cost weight	e_PCB	0.803	0.553	1.405	normal	0.803	0.009	NA

Note: Adapted from the ValueDecisions' predictions template (9). Options: [a_VB_shc] Vaginal birth with standard hospital care; [b_VB_cmc] Vaginal birth with continuous midwifery care; [c_ICB_shc] Intrapartum cesarean birth with standard hospital care; [d_ICB_cmc] Intrapartum cesarean birth with continuous midwifery care; [e_PCB] Prelabor cesarean birth; NA: not applicable. [a] Minimum/maximum across all options. [b] Normal distribution: expectation value (mean, average); Triangular distribution: minimum. [c] Normal distribution: standard deviation; Triangular distribution: maximum. [d] Normal distribution: not applicable; Triangular distribution: mode.

S2-1f. Performance predictions with uncertainty, scenario 3

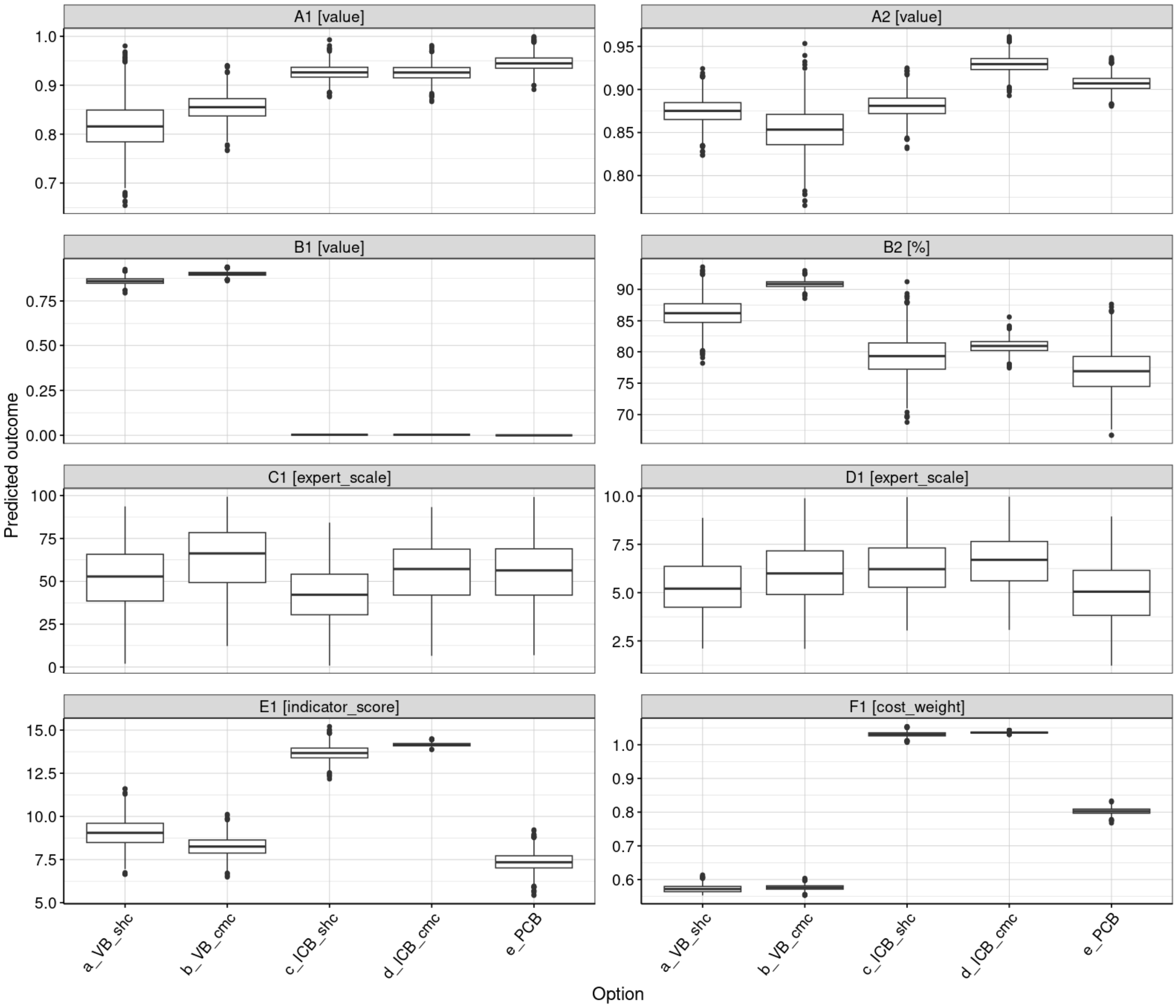

Note: Graph by ValueDecisions (9). Performance predictions for scenario 3. Uncertainty in the predictions was accounted for by 2,000 Monte Carlo simulations. Goals/attributes (units): A1: Low maternal complication rates/weighted composite index (value); A2: Low neonatal complication rates/weighted composite index (value); B1: Physiological labor and childbirth processes/weighted composite index (value); B2: Positive initiation of bonding and breastfeeding after childbirth/exclusive breastfeeding at hospital discharge (%); C1: Positive psychosocial experience of care interactions and events/expert assessment (scale); D1: Low physical strain for care providers/expert assessment (scale); E1: Low resource use in care setting/clinical indicator score (score); F1: Low direct costs to the healthcare system/SwissDRG cost weight (cost weight). Options: [a_VB_shc] Vaginal birth with standard hospital care; [b_VB_cmc] Vaginal birth with continuous midwifery care; [c_ICB_shc] Intrapartum cesarean birth with standard hospital care; [d_ICB_cmc] Intrapartum cesarean birth with continuous midwifery care; [e_PCB] Prelabor cesarean birth.

S2-1g. Prediction matrix, scenario 4

Goal	Attribute	Unit	Option	Prediction	Minimum[a]	Maximum[a]	Uncertainty distribution	Uncertainty distribution parameter 1[b]	Uncertainty distribution parameter 2[c]	Uncertainty distribution parameter 3[d]
A1. Low maternal complication rates	Weighted composite index	Value	a_VB_shc	0.810	0.3	1	normal	0.810	0.044	NA
A1. Low maternal complication rates	Weighted composite index	Value	b_VB_cmc	0.823	0.3	1	normal	0.823	0.015	NA
A1. Low maternal complication rates	Weighted composite index	Value	c_ICB_shc	0.917	0.3	1	normal	0.917	0.014	NA
A1. Low maternal complication rates	Weighted composite index	Value	d_ICB_cmc	0.924	0.3	1	normal	0.924	0.013	NA
A1. Low maternal complication rates	Weighted composite index	Value	e_PCB	0.943	0.3	1	normal	0.943	0.013	NA
A2. Low neonatal complication rates	Weighted composite index	Value	a_VB_shc	0.856	0.3	1	normal	0.856	0.019	NA
A2. Low neonatal complication rates	Weighted composite index	Value	b_VB_cmc	0.802	0.3	1	normal	0.802	0.015	NA
A2. Low neonatal complication rates	Weighted composite index	Value	c_ICB_shc	0.852	0.3	1	normal	0.852	0.020	NA
A2. Low neonatal complication rates	Weighted composite index	Value	d_ICB_cmc	0.920	0.3	1	normal	0.920	0.014	NA
A2. Low neonatal complication rates	Weighted composite index	Value	e_PCB	0.885	0.3	1	normal	0.885	0.015	NA
B1. Physiological labor and childbirth processes	Weighted composite index	Value	a_VB_shc	0.845	0	1	normal	0.845	0.020	NA
B1. Physiological labor and childbirth processes	Weighted composite index	Value	b_VB_cmc	0.867	0	1	normal	0.867	0.003	NA
B1. Physiological labor and childbirth processes	Weighted composite index	Value	c_ICB_shc	0.002	0	1	normal	0.002	0.000	NA
B1. Physiological labor and childbirth processes	Weighted composite index	Value	d_ICB_cmc	0.002	0	1	normal	0.002	0.000	NA
B1. Physiological labor and childbirth processes	Weighted composite index	Value	e_PCB	0	0	1	normal	0	0.000	NA
B2. Positive initiation of breastfeeding after childbirth	Exclusive breastfeeding	%	a_VB_shc	83.056	50	95	normal	83.056	3.015	NA
B2. Positive initiation of breastfeeding after childbirth	Exclusive breastfeeding	%	b_VB_cmc	88.957	50	95	normal	88.957	0.682	NA
B2. Positive initiation of breastfeeding after childbirth	Exclusive breastfeeding	%	c_ICB_shc	75.958	50	95	normal	75.958	3.006	NA
B2. Positive initiation of breastfeeding after childbirth	Exclusive breastfeeding	%	d_ICB_cmc	77.483	50	95	normal	77.483	0.714	NA
B2. Positive initiation of breastfeeding after childbirth	Exclusive breastfeeding	%	e_PCB	72.743	50	95	normal	72.743	3.892	NA
C1. Positive psychosocial experience of care	Expert assessment	Scale	a_VB_shc	60	0	100	triangular	0	95	60
C1. Positive psychosocial experience of care	Expert assessment	Scale	b_VB_cmc	80	0	100	triangular	10	100	80
C1. Positive psychosocial experience of care	Expert assessment	Scale	c_ICB_shc	45	0	100	triangular	0	85	45
C1. Positive psychosocial experience of care	Expert assessment	Scale	d_ICB_cmc	65	0	100	triangular	5	95	65
C1. Positive psychosocial experience of care	Expert assessment	Scale	e_PCB	60	0	100	triangular	5	100	60
D1. Low physical strain for care providers	Expert assessment	Scale	a_VB_shc	5	1	10	triangular	2	9	5
D1. Low physical strain for care providers	Expert assessment	Scale	b_VB_cmc	6	1	10	triangular	2	10	6
D1. Low physical strain for care providers	Expert assessment	Scale	c_ICB_shc	6	1	10	triangular	3	10	6
D1. Low physical strain for care providers	Expert assessment	Scale	d_ICB_cmc	7	1	10	triangular	3	10	7
D1. Low physical strain for care providers	Expert assessment	Scale	e_PCB	5	1	10	triangular	1	9	5
E1. Low resource use in care setting	Clinical indicator score	Score	a_VB_shc	13.932	5	36	normal	13.932	0.757	NA
E1. Low resource use in care setting	Clinical indicator score	Score	b_VB_cmc	13.498	5	36	normal	13.498	0.074	NA
E1. Low resource use in care setting	Clinical indicator score	Score	c_ICB_shc	18.606	5	36	normal	18.606	0.469	NA
E1. Low resource use in care setting	Clinical indicator score	Score	d_ICB_cmc	18.641	5	36	normal	18.641	0.637	NA
E1. Low resource use in care setting	Clinical indicator score	Score	e_PCB	12.308	5	36	normal	12.308	0.597	NA

F1. Low direct costs to the healthcare system	SwissDRG cost weight	Cost weight	a_VB_shc	0.633	0.553	1.405	normal	0.633	0.012	NA
F1. Low direct costs to the healthcare system	SwissDRG cost weight	Cost weight	b_VB_cmc	0.647	0.553	1.405	normal	0.647	0.003	NA
F1. Low direct costs to the healthcare system	SwissDRG cost weight	Cost weight	c_ICB_shc	1.095	0.553	1.405	normal	1.095	0.008	NA
F1. Low direct costs to the healthcare system	SwissDRG cost weight	Cost weight	d_ICB_cmc	1.093	0.553	1.405	normal	1.093	0.010	NA
F1. Low direct costs to the healthcare system	SwissDRG cost weight	Cost weight	e_PCB	0.867	0.553	1.405	normal	0.867	0.010	NA

Note: Adapted from the ValueDecisions' predictions template (9). Options: [a_VB_shc] Vaginal birth with standard hospital care; [b_VB_cmc] Vaginal birth with continuous midwifery care; [c_ICB_shc] Intrapartum cesarean birth with standard hospital care; [d_ICB_cmc] Intrapartum cesarean birth with continuous midwifery care; [e_PCB] Prelabor cesarean birth; NA: not applicable. [a] Minimum/maximum across all options. [b] Normal distribution: expectation value (mean, average); Triangular distribution: minimum. [c] Normal distribution: standard deviation; Triangular distribution: maximum. [d] Normal distribution: not applicable; Triangular distribution: mode.

S2-1h. Performance predictions with uncertainty, scenario 4

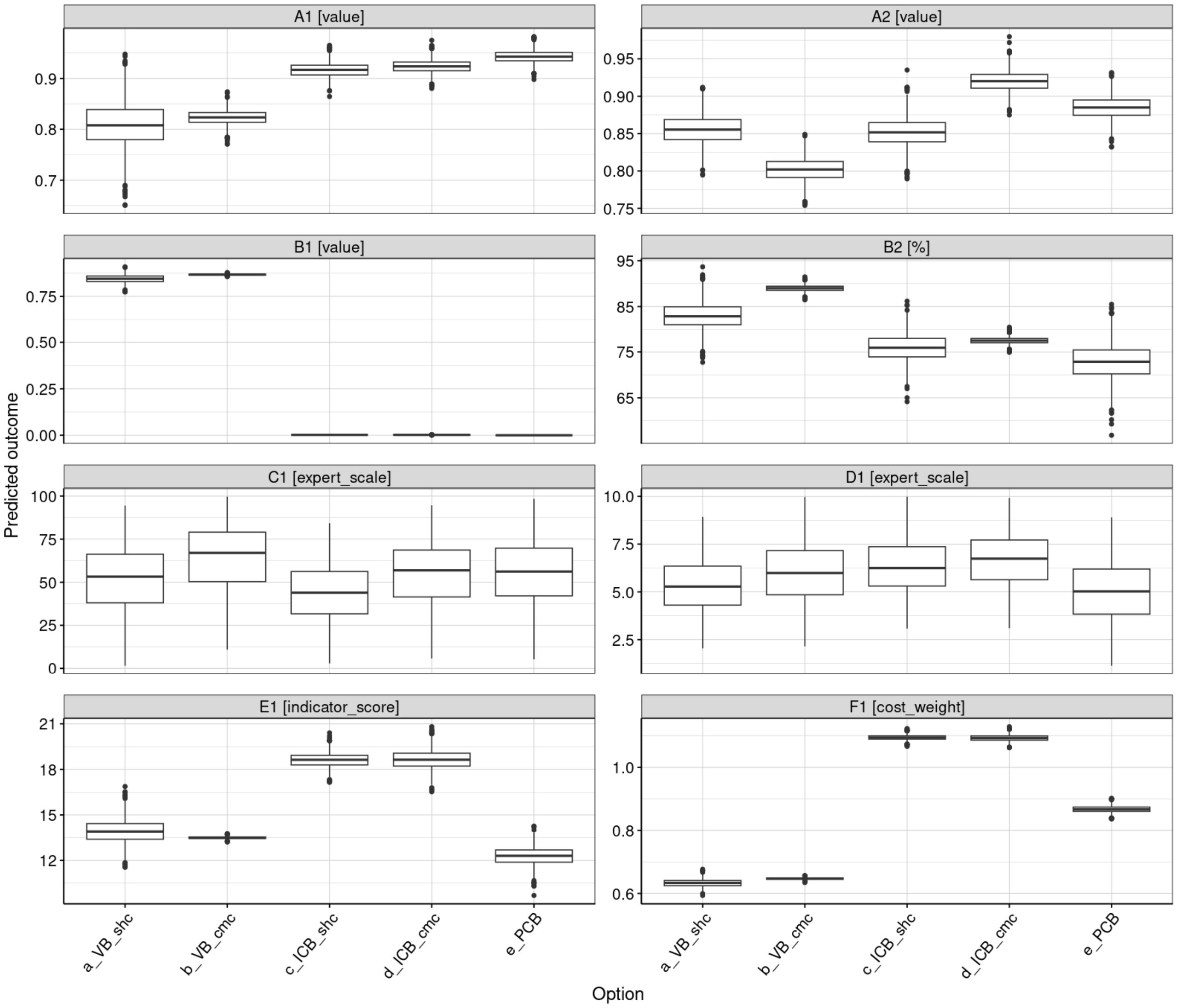

Note: Graph by ValueDecisions (9). Performance predictions for scenario 4. Uncertainty in the predictions was accounted for by 2,000 Monte Carlo simulations. Goals/attributes (units): A1: Low maternal complication rates/weighted composite index (value); A2: Low neonatal complication rates/weighted composite index (value); B1: Physiological labor and childbirth processes/weighted composite index (value); B2: Positive initiation of bonding and breastfeeding after childbirth/exclusive breastfeeding at hospital discharge (%); C1: Positive psychosocial experience of care interactions and events/expert assessment (scale); D1: Low physical strain for care providers/expert assessment (scale); E1: Low resource use in care setting/clinical indicator score (score); F1: Low direct costs to the healthcare system/SwissDRG cost weight (cost weight). Options: [a_VB_shc] Vaginal birth with standard hospital care; [b_VB_cmc] Vaginal birth with continuous midwifery care; [c_ICB_shc] Intrapartum cesarean birth with standard hospital care; [d_ICB_cmc] Intrapartum cesarean birth with continuous midwifery care; [e_PCB] Prelabor cesarean birth.

S2-2. Stakeholder preferences

S2-2a. Preference matrix, all scenarios

Stakeholder	Goal	Attribute	Unit	Worst	Best	Shape of value function	Attribute levels	Values of attribute levels	Global weight
Biomedical safety enthusiast	A1. Low maternal complication rates	Weighted composite index	Value	0.3	1	linear[a]	0.3/1	0/1	0.45
Biomedical safety enthusiast	A2. Low neonatal complication rates	Weighted composite index	Value	0.3	1	linear[a]	0.3/1	0/1	0.45
Biomedical safety enthusiast	B1. Physiological labor and childbirth processes	Weighted composite index	Value	0	1	linear[a]	0/1	0/1	0
Biomedical safety enthusiast	B2. Positive initiation of breastfeeding after childbirth	Exclusive breastfeeding	%	50	95	linear[b]	50/95	0/1	0
Biomedical safety enthusiast	C1. Positive psychosocial experience of care interactions and events	Expert assessment	Scale	0	100	linear[b]	0/100	0/1	0
Biomedical safety enthusiast	D1. Low physical strain for care providers	Expert assessment	Scale	10	1	linear[b]	10/1	0/1	0.1
Biomedical safety enthusiast	E1. Low resource use in care setting	Clinical indicator score	Score	36	5	linear[b]	36/5	0/1	0
Biomedical safety enthusiast	F1. Low direct costs to the healthcare system	SwissDRG cost weight	Cost weight	1.405	0.553	linear[b]	1.405/0.553	0/1	0
Natural birth proponent	A1. Low maternal complication rates	Weighted composite index	Value	0.3	1	linear[a]	0.3/1	0/1	0.1
Natural birth proponent	A2. Low neonatal complication rates	Weighted composite index	Value	0.3	1	linear[a]	0.3/1	0/1	0.1
Natural birth proponent	B1. Physiological labor and childbirth processes	Weighted composite index	Value	0	1	linear[a]	0/1	0/1	0.4
Natural birth proponent	B2. Positive initiation of breastfeeding after childbirth	Exclusive breastfeeding	%	50	95	linear[b]	50/95	0/1	0.25
Natural birth proponent	C1. Positive psychosocial experience of care interactions and events	Expert assessment	Scale	0	100	linear[b]	0/100	0/1	0.15
Natural birth proponent	D1. Low physical strain for care providers	Expert assessment	Scale	10	1	linear[b]	10/1	0/1	0
Natural birth proponent	E1. Low resource use in care setting	Clinical indicator score	Score	36	5	linear[b]	36/5	0/1	0
Natural birth proponent	F1. Low direct costs to the healthcare system	SwissDRG cost weight	Cost weight	1.405	0.553	linear[b]	1.405/0.553	0/1	0
Psychosocial experience advocate	A1. Low maternal complication rates	Weighted composite index	Value	0.3	1	linear[a]	0.3/1	0/1	0.1
Psychosocial experience advocate	A2. Low neonatal complication rates	Weighted composite index	Value	0.3	1	linear[a]	0.3/1	0/1	0.2
Psychosocial experience advocate	B1. Physiological labor and childbirth processes	Weighted composite index	Value	0	1	linear[a]	0/1	0/1	0
Psychosocial experience advocate	B2. Positive initiation of breastfeeding after childbirth	Exclusive breastfeeding	%	50	95	linear[b]	50/95	0/1	0
Psychosocial experience advocate	C1. Positive psychosocial experience of care interactions and events	Expert assessment	Scale	0	100	linear[b]	0/100	0/1	0.7
Psychosocial experience advocate	D1. Low physical strain for care providers	Expert assessment	Scale	10	1	linear[b]	10/1	0/1	0
Psychosocial experience advocate	E1. Low resource use in care setting	Clinical indicator score	Score	36	5	linear[b]	36/5	0/1	0
Psychosocial experience advocate	F1. Low direct costs to the healthcare system	SwissDRG cost weight	Cost weight	1.405	0.553	linear[b]	1.405/0.553	0/1	0
Resource utilization pragmatist	A1. Low maternal complication rates	Weighted composite index	Value	0.3	1	linear[a]	0.3/1	0/1	0.25
Resource utilization pragmatist	A2. Low neonatal complication rates	Weighted composite index	Value	0.3	1	linear[a]	0.3/1	0/1	0.25
Resource utilization pragmatist	B1. Physiological labor and childbirth processes	Weighted composite index	Value	0	1	linear[a]	0/1	0/1	0
Resource utilization pragmatist	B2. Positive initiation of breastfeeding after childbirth	Exclusive breastfeeding	%	50	95	linear[b]	50/95	0/1	0
Resource utilization pragmatist	C1. Positive psychosocial experience of care interactions and events	Expert assessment	Scale	0	100	linear[b]	0/100	0/1	0
Resource utilization pragmatist	D1. Low physical strain for care providers	Expert assessment	Scale	10	1	linear[b]	10/1	0/1	0.1
Resource utilization pragmatist	E1. Low resource use in care setting	Clinical indicator score	Score	36	5	linear[b]	36/5	0/1	0.3
Resource utilization pragmatist	F1. Low direct costs to the healthcare system	SwissDRG cost weight	Cost weight	1.405	0.553	linear[b]	1.405/0.553	0/1	0.1

Real stakeholder 1	A1. Low maternal complication rates	Weighted composite index	Value	0.3	1	linear[a]	0.3/1	0/1	0.142
Real stakeholder 1	A2. Low neonatal complication rates	Weighted composite index	Value	0.3	1	linear[a]	0.3/1	0/1	0.158
Real stakeholder 1	B1. Physiological labor and childbirth processes	Weighted composite index	Value	0	1	linear[a]	0/1	0/1	0.111
Real stakeholder 1	B2. Positive initiation of breastfeeding after childbirth	Exclusive breastfeeding	%	50	95	interpolate	50/55/65/77/95	0/0.25/0.5/0.75/1	0.123
Real stakeholder 1	C1. Positive psychosocial experience of care interactions and events	Expert assessment	Scale	0	100	interpolate	0/12/30/55/100	0/0.25/0.5/0.75/1	0.333
Real stakeholder 1	D1. Low physical strain for care providers	Expert assessment	Scale	10	1	interpolate	10/8.8/7.3/4.6/1	0/0.25/0.5/0.75/1	0.067
Real stakeholder 1	E1. Low resource use in care setting	Clinical indicator score	Score	36	5	interpolate	36/32/25/16/5	0/0.25/0.5/0.75/1	0.033
Real stakeholder 1	F1. Low direct costs to the healthcare system	SwissDRG cost weight	Cost weight	1.405	0.553	interpolate	1.405/1.309/1.149/0.915/0.553	0/0.25/0.5/0.75/1	0.033
Real stakeholder 2	A1. Low maternal complication rates	Weighted composite index	Value	0.3	1	linear[a]	0.3/1	0/1	0.127
Real stakeholder 2	A2. Low neonatal complication rates	Weighted composite index	Value	0.3	1	linear[a]	0.3/1	0/1	0.120
Real stakeholder 2	B1. Physiological labor and childbirth processes	Weighted composite index	Value	0	1	linear[a]	0/1	0/1	0.074
Real stakeholder 2	B2. Positive initiation of breastfeeding after childbirth	Exclusive breastfeeding	%	50	95	interpolate	50/59.56/70.81/82.63/95	0/0.25/0.5/0.75/1	0.123
Real stakeholder 2	C1. Positive psychosocial experience of care interactions and events	Expert assessment	Scale	0	100	interpolate	0/20/45/70/100	0/0.25/0.5/0.75/1	0.235
Real stakeholder 2	D1. Low physical strain for care providers	Expert assessment	Scale	10	1	interpolate	10/8.43/6.18/3.70/1	0/0.25/0.5/0.75/1	0.210
Real stakeholder 2	E1. Low resource use in care setting	Clinical indicator score	Score	36	5	interpolate	36/30.56/22.83/14.30/5	0/0.25/0.5/0.75/1	0.062
Real stakeholder 2	F1. Low direct costs to the healthcare system	SwissDRG cost weight	Cost weight	1.405	0.553	interpolate	1.405/1.192/0.979/0.766/0.553	0/0.25/0.5/0.75/1	0.049
Real stakeholder 3	A1. Low maternal complication rates	Weighted composite index	Value	0.3	1	linear[a]	0.3/1	0/1	0.128
Real stakeholder 3	A2. Low neonatal complication rates	Weighted composite index	Value	0.3	1	linear[a]	0.3/1	0/1	0.151
Real stakeholder 3	B1. Physiological labor and childbirth processes	Weighted composite index	Value	0	1	linear[a]	0/1	0/1	0.116
Real stakeholder 3	B2. Positive initiation of breastfeeding after childbirth	Exclusive breastfeeding	%	50	95	interpolate	50/61.25/72.5/83.75/95	0/0.25/0.5/0.75/1	0.144
Real stakeholder 3	C1. Positive psychosocial experience of care interactions and events	Expert assessment	Scale	0	100	interpolate	0/13/36/76/100	0/0.25/0.5/0.75/1	0.209
Real stakeholder 3	D1. Low physical strain for care providers	Expert assessment	Scale	10	1	interpolate	10/9/7/5/1	0/0.25/0.5/0.75/1	0.140
Real stakeholder 3	E1. Low resource use in care setting	Clinical indicator score	Score	36	5	interpolate	36/28.25/20.5/12.75/5	0/0.25/0.5/0.75/1	0.084
Real stakeholder 3	F1. Low direct costs to the healthcare system	SwissDRG cost weight	Cost weight	1.405	0.553	interpolate	1.405/1.192/0.979/0.766/0.553	0/0.25/0.5/0.75/1	0.028

Note: Adapted from the ValueDecisions' preferences template (9). [a] Linear value functions were assumed for the weighted composite indices, as expert-derived non-linear value functions had already been integrated to construct them. [b] Linear value functions were assumed for the hypothetical stakeholders' preferences for within-goal changes.

S2-2b. Value functions of real stakeholders

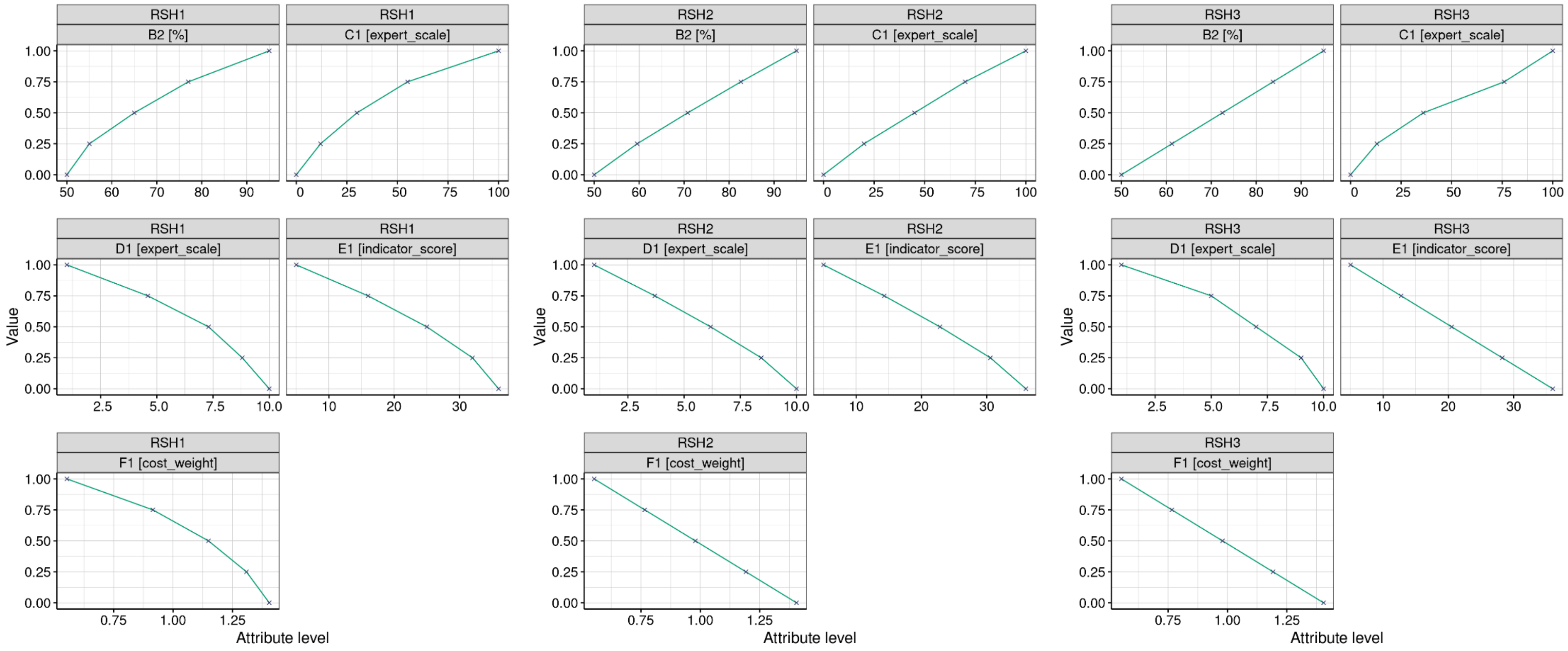

Note: Graphs by ValueDecisions (9). Value functions plots of the real stakeholders. RSH1: Real stakeholder 1; RSH2: Real stakeholder 2; RSH3: Real stakeholder 3. Goals/attributes (units): B2: Positive initiation of bonding and breastfeeding after childbirth/exclusive breastfeeding at hospital discharge (%); C1: Positive psychosocial experience of care interactions and events/expert assessment (scale); D1: Low physical strain for care providers/expert assessment (scale); E1: Low resource use in care setting/clinical indicator score (score); F1: Low direct costs to the healthcare system/SwissDRG cost weight (cost weight). For the goals operationalized by weighted composite indices (A1 Low maternal complication rates, A2 Low neonatal complication rates, and B1 Physiological labor and childbirth processes), we assumed linear value functions in the main model, as expert-derived non-linear value functions had already been integrated to construct them.

S3. MCDA results

S3-1. Main model: Overall values of childbirth options with stakeholder value functions and γ=0.2

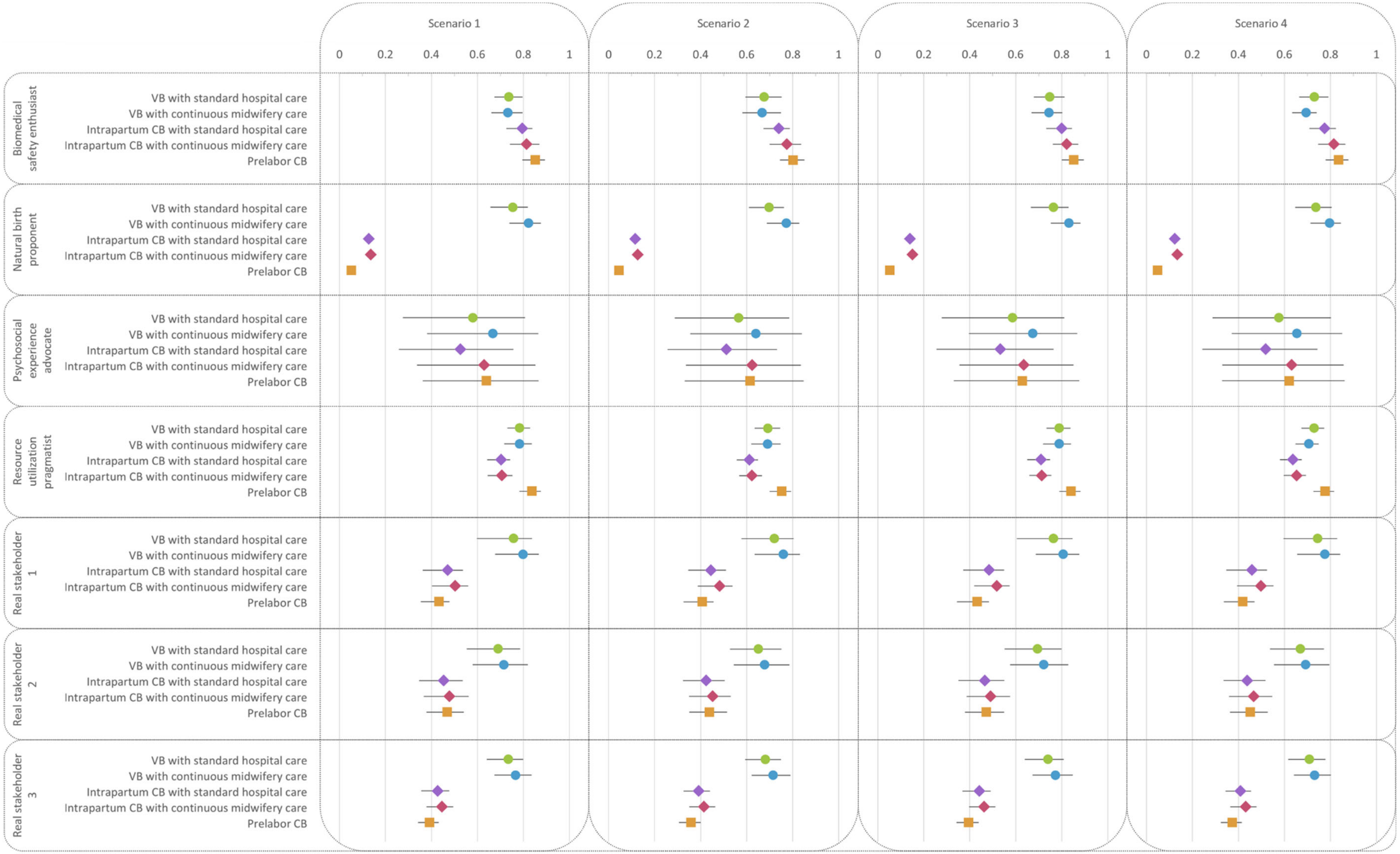

Note: Mean overall values for each option and each stakeholder in each scenario using stakeholder value functions and a non-additive aggregation model with γ=0.2. Values normalized to the interval [0, 1], with higher values indicating better overall performance aligned with individual preferences. Uncertainty in the predictions was accounted for by 2,000 Monte Carlo simulations. Error bars indicate the 5% and 95% quantiles of the overall values. Scenarios: (1) Women with no cesarean birth history or comorbidities; (2) Women with no cesarean birth history but comorbidities; (3) Women with a cesarean birth history but no comorbidities; (4) Women with a cesarean birth history and comorbidities. Childbirth options: ● Vaginal birth (VB) with standard hospital care; ● Vaginal birth (VB) with continuous midwifery care; ◆ Intrapartum cesarean birth (CB) with standard hospital care; ◆ Intrapartum cesarean birth (CB) with continuous midwifery care; ■ Prelabor cesarean birth (CB).

S3-1a. Overall values of childbirth options with stakeholder value functions and γ=0.2, scenario 1

Stakeholder	Option	Mean overall value	5% quantile of overall value	95% quantile of overall value	Minimum	Maximum
Biomedical safety enthusiast	a_VB_shc	0.736	0.674	0.797	0.602	0.865
Biomedical safety enthusiast	b_VB_cmc	0.732	0.661	0.797	0.541	0.877
Biomedical safety enthusiast	c_ICB_shc	0.794	0.726	0.839	0.624	0.875
Biomedical safety enthusiast	d_ICB_cmc	0.813	0.741	0.869	0.642	0.905
Biomedical safety enthusiast	e_PCB	0.851	0.796	0.893	0.753	0.915
Natural birth proponent	a_VB_shc	0.753	0.657	0.819	0.536	0.862
Natural birth proponent	b_VB_cmc	0.822	0.739	0.876	0.654	0.909
Natural birth proponent	c_ICB_shc	0.127	0.104	0.145	0.075	0.159
Natural birth proponent	d_ICB_cmc	0.136	0.114	0.152	0.094	0.163
Natural birth proponent	e_PCB	0.051	0.041	0.059	0.033	0.067
Psychosocial experience advocate	a_VB_shc	0.58	0.276	0.807	0.065	0.908
Psychosocial experience advocate	b_VB_cmc	0.666	0.381	0.865	0.209	0.933
Psychosocial experience advocate	c_ICB_shc	0.525	0.258	0.757	0.064	0.843
Psychosocial experience advocate	d_ICB_cmc	0.628	0.337	0.852	0.155	0.93
Psychosocial experience advocate	e_PCB	0.638	0.362	0.866	0.153	0.954
Resource utilization pragmatist	a_VB_shc	0.783	0.730	0.829	0.682	0.878
Resource utilization pragmatist	b_VB_cmc	0.783	0.716	0.837	0.603	0.888
Resource utilization pragmatist	c_ICB_shc	0.702	0.642	0.742	0.549	0.764
Resource utilization pragmatist	d_ICB_cmc	0.706	0.645	0.752	0.562	0.777
Resource utilization pragmatist	e_PCB	0.836	0.782	0.876	0.745	0.899
Real stakeholder 1	a_VB_shc	0.756	0.598	0.838	0.326	0.89
Real stakeholder 1	b_VB_cmc	0.798	0.677	0.867	0.497	0.907
Real stakeholder 1	c_ICB_shc	0.47	0.362	0.537	0.19	0.572
Real stakeholder 1	d_ICB_cmc	0.503	0.403	0.56	0.282	0.586
Real stakeholder 1	e_PCB	0.432	0.354	0.479	0.243	0.508
Real stakeholder 2	a_VB_shc	0.689	0.554	0.786	0.354	0.852
Real stakeholder 2	b_VB_cmc	0.714	0.579	0.819	0.362	0.878
Real stakeholder 2	c_ICB_shc	0.453	0.346	0.536	0.235	0.602
Real stakeholder 2	d_ICB_cmc	0.477	0.367	0.562	0.245	0.607
Real stakeholder 2	e_PCB	0.468	0.379	0.541	0.291	0.598
Real stakeholder 3	a_VB_shc	0.734	0.64	0.799	0.438	0.852
Real stakeholder 3	b_VB_cmc	0.765	0.674	0.836	0.501	0.88
Real stakeholder 3	c_ICB_shc	0.427	0.356	0.478	0.248	0.522
Real stakeholder 3	d_ICB_cmc	0.445	0.379	0.495	0.291	0.527
Real stakeholder 3	e_PCB	0.391	0.342	0.432	0.28	0.47

Note: Overall values for each option and each stakeholder using stakeholder value functions and a non-additive aggregation model with γ=0.2 for scenario 1. Values normalized to the interval [0, 1], with higher values indicating better overall performance aligned with individual preferences. Uncertainty in the predictions was accounted for by 2,000 Monte Carlo simulations. Options: [a_VB_shc] Vaginal birth with standard hospital care; [b_VB_cmc] Vaginal birth with continuous midwifery care; [c_ICB_shc] Intrapartum cesarean birth with standard hospital care; [d_ICB_cmc] Intrapartum cesarean birth with continuous midwifery care; [e_PCB] Prelabor cesarean birth.

S3-1b. Overall values of childbirth options with stakeholder value functions and γ=0.2, scenario 2

Stakeholder	Option	Mean overall value	5% quantile of overall value	95% quantile of overall value	Minimum	Maximum
Biomedical safety enthusiast	a_VB_shc	0.675	0.595	0.752	0.516	0.832
Biomedical safety enthusiast	b_VB_cmc	0.667	0.582	0.749	0.458	0.84
Biomedical safety enthusiast	c_ICB_shc	0.739	0.673	0.788	0.55	0.845
Biomedical safety enthusiast	d_ICB_cmc	0.774	0.700	0.837	0.608	0.899
Biomedical safety enthusiast	e_PCB	0.801	0.745	0.851	0.702	0.887
Natural birth proponent	a_VB_shc	0.697	0.61	0.762	0.522	0.814
Natural birth proponent	b_VB_cmc	0.772	0.688	0.829	0.623	0.861
Natural birth proponent	c_ICB_shc	0.115	0.093	0.133	0.063	0.152
Natural birth proponent	d_ICB_cmc	0.126	0.104	0.144	0.088	0.156
Natural birth proponent	e_PCB	0.044	0.033	0.053	0.02	0.061
Psychosocial experience advocate	a_VB_shc	0.565	0.287	0.785	0.095	0.873
Psychosocial experience advocate	b_VB_cmc	0.639	0.355	0.84	0.215	0.916
Psychosocial experience advocate	c_ICB_shc	0.511	0.256	0.732	0.051	0.822
Psychosocial experience advocate	d_ICB_cmc	0.623	0.336	0.836	0.193	0.909
Psychosocial experience advocate	e_PCB	0.614	0.331	0.848	0.159	0.941
Resource utilization pragmatist	a_VB_shc	0.692	0.635	0.746	0.57	0.79
Resource utilization pragmatist	b_VB_cmc	0.69	0.620	0.748	0.514	0.804
Resource utilization pragmatist	c_ICB_shc	0.611	0.557	0.649	0.46	0.674
Resource utilization pragmatist	d_ICB_cmc	0.622	0.568	0.666	0.486	0.704
Resource utilization pragmatist	e_PCB	0.751	0.700	0.794	0.664	0.817
Real stakeholder 1	a_VB_shc	0.720	0.578	0.804	0.369	0.848
Real stakeholder 1	b_VB_cmc	0.759	0.635	0.832	0.513	0.88
Real stakeholder 1	c_ICB_shc	0.444	0.346	0.51	0.17	0.545
Real stakeholder 1	d_ICB_cmc	0.482	0.387	0.538	0.297	0.561
Real stakeholder 1	e_PCB	0.406	0.325	0.456	0.22	0.485
Real stakeholder 2	a_VB_shc	0.65	0.527	0.751	0.364	0.828
Real stakeholder 2	b_VB_cmc	0.677	0.544	0.786	0.36	0.858
Real stakeholder 2	c_ICB_shc	0.423	0.323	0.505	0.216	0.563
Real stakeholder 2	d_ICB_cmc	0.451	0.349	0.531	0.215	0.581
Real stakeholder 2	e_PCB	0.437	0.35	0.514	0.237	0.567
Real stakeholder 3	a_VB_shc	0.681	0.594	0.749	0.443	0.804
Real stakeholder 3	b_VB_cmc	0.714	0.622	0.79	0.479	0.844
Real stakeholder 3	c_ICB_shc	0.391	0.325	0.441	0.225	0.48
Real stakeholder 3	d_ICB_cmc	0.414	0.350	0.463	0.258	0.495
Real stakeholder 3	e_PCB	0.357	0.306	0.400	0.227	0.435

Note: Overall values for each option and each stakeholder using stakeholder value functions and a non-additive aggregation model with γ=0.2 for scenario 2. Values normalized to the interval [0, 1], with higher values indicating better overall performance aligned with individual preferences. Uncertainty in the predictions was accounted for by 2,000 Monte Carlo simulations. Options: [a_VB_shc] Vaginal birth with standard hospital care; [b_VB_cmc] Vaginal birth with continuous midwifery care; [c_ICB_shc] Intrapartum cesarean birth with standard hospital care; [d_ICB_cmc] Intrapartum cesarean birth with continuous midwifery care; [e_PCB] Prelabor cesarean birth.

S3-1c. Overall values of childbirth options with stakeholder value functions and γ=0.2, scenario 3

Stakeholder	Option	Mean overall value	5% quantile of overall value	95% quantile of overall value	Minimum	Maximum
Biomedical safety enthusiast	a_VB_shc	0.747	0.679	0.812	0.598	0.879
Biomedical safety enthusiast	b_VB_cmc	0.744	0.669	0.803	0.569	0.856
Biomedical safety enthusiast	c_ICB_shc	0.799	0.733	0.845	0.655	0.873
Biomedical safety enthusiast	d_ICB_cmc	0.821	0.761	0.872	0.675	0.902
Biomedical safety enthusiast	e_PCB	0.852	0.800	0.896	0.75	0.922
Natural birth proponent	a_VB_shc	0.764	0.666	0.83	0.47	0.866
Natural birth proponent	b_VB_cmc	0.831	0.753	0.882	0.67	0.906
Natural birth proponent	c_ICB_shc	0.139	0.114	0.158	0.082	0.172
Natural birth proponent	d_ICB_cmc	0.15	0.129	0.165	0.105	0.172
Natural birth proponent	e_PCB	0.051	0.041	0.060	0.032	0.066
Psychosocial experience advocate	a_VB_shc	0.585	0.278	0.811	0.032	0.9
Psychosocial experience advocate	b_VB_cmc	0.673	0.396	0.868	0.214	0.938
Psychosocial experience advocate	c_ICB_shc	0.532	0.255	0.765	0.068	0.845
Psychosocial experience advocate	d_ICB_cmc	0.634	0.355	0.851	0.158	0.923
Psychosocial experience advocate	e_PCB	0.628	0.330	0.876	0.177	0.954
Resource utilization pragmatist	a_VB_shc	0.789	0.734	0.839	0.676	0.88
Resource utilization pragmatist	b_VB_cmc	0.789	0.719	0.841	0.618	0.873
Resource utilization pragmatist	c_ICB_shc	0.709	0.650	0.749	0.576	0.769
Resource utilization pragmatist	d_ICB_cmc	0.712	0.659	0.755	0.583	0.773
Resource utilization pragmatist	e_PCB	0.840	0.790	0.882	0.743	0.908
Real stakeholder 1	a_VB_shc	0.763	0.605	0.847	0.251	0.889
Real stakeholder 1	b_VB_cmc	0.806	0.688	0.876	0.557	0.913
Real stakeholder 1	c_ICB_shc	0.483	0.371	0.549	0.208	0.582
Real stakeholder 1	d_ICB_cmc	0.517	0.419	0.573	0.293	0.596
Real stakeholder 1	e_PCB	0.431	0.344	0.483	0.261	0.504
Real stakeholder 2	a_VB_shc	0.694	0.552	0.799	0.329	0.857
Real stakeholder 2	b_VB_cmc	0.721	0.576	0.829	0.417	0.898
Real stakeholder 2	c_ICB_shc	0.465	0.351	0.551	0.185	0.611
Real stakeholder 2	d_ICB_cmc	0.49	0.387	0.575	0.275	0.619
Real stakeholder 2	e_PCB	0.471	0.379	0.550	0.275	0.592
Real stakeholder 3	a_VB_shc	0.740	0.640	0.809	0.393	0.859
Real stakeholder 3	b_VB_cmc	0.772	0.673	0.848	0.551	0.895
Real stakeholder 3	c_ICB_shc	0.441	0.368	0.491	0.23	0.532
Real stakeholder 3	d_ICB_cmc	0.461	0.397	0.511	0.316	0.543
Real stakeholder 3	e_PCB	0.394	0.342	0.439	0.275	0.469

Note: Overall values for each option and each stakeholder using stakeholder value functions and a non-additive aggregation model with γ=0.2 for scenario 3. Values normalized to the interval [0, 1], with higher values indicating better overall performance aligned with individual preferences. Uncertainty in the predictions was accounted for by 2,000 Monte Carlo simulations. Options: [a_VB_shc] Vaginal birth with standard hospital care; [b_VB_cmc] Vaginal birth with continuous midwifery care; [c_ICB_shc] Intrapartum cesarean birth with standard hospital care; [d_ICB_cmc] Intrapartum cesarean birth with continuous midwifery care; [e_PCB] Prelabor cesarean birth.

S3-1d. Overall values of childbirth options with stakeholder value functions and γ=0.2, scenario 4

Stakeholder	Option	Mean overall value	5% quantile of overall value	95% quantile of overall value	Minimum	Maximum
Biomedical safety enthusiast	a_VB_shc	0.729	0.664	0.791	0.607	0.845
Biomedical safety enthusiast	b_VB_cmc	0.694	0.634	0.740	0.555	0.779
Biomedical safety enthusiast	c_ICB_shc	0.774	0.709	0.824	0.613	0.861
Biomedical safety enthusiast	d_ICB_cmc	0.814	0.747	0.865	0.672	0.898
Biomedical safety enthusiast	e_PCB	0.835	0.779	0.878	0.726	0.905
Natural birth proponent	a_VB_shc	0.736	0.647	0.805	0.49	0.855
Natural birth proponent	b_VB_cmc	0.796	0.713	0.845	0.644	0.859
Natural birth proponent	c_ICB_shc	0.123	0.100	0.140	0.063	0.152
Natural birth proponent	d_ICB_cmc	0.134	0.112	0.149	0.093	0.157
Natural birth proponent	e_PCB	0.048	0.038	0.056	0.027	0.064
Psychosocial experience advocate	a_VB_shc	0.575	0.288	0.802	0.06	0.892
Psychosocial experience advocate	b_VB_cmc	0.653	0.371	0.851	0.214	0.907
Psychosocial experience advocate	c_ICB_shc	0.518	0.243	0.743	0.032	0.832
Psychosocial experience advocate	d_ICB_cmc	0.631	0.330	0.857	0.17	0.93
Psychosocial experience advocate	e_PCB	0.620	0.329	0.862	0.158	0.942
Resource utilization pragmatist	a_VB_shc	0.728	0.674	0.773	0.619	0.807
Resource utilization pragmatist	b_VB_cmc	0.706	0.648	0.749	0.565	0.768
Resource utilization pragmatist	c_ICB_shc	0.636	0.581	0.675	0.505	0.704
Resource utilization pragmatist	d_ICB_cmc	0.652	0.597	0.694	0.529	0.716
Resource utilization pragmatist	e_PCB	0.776	0.726	0.816	0.678	0.836
Real stakeholder 1	a_VB_shc	0.744	0.596	0.829	0.288	0.886
Real stakeholder 1	b_VB_cmc	0.775	0.656	0.842	0.531	0.869
Real stakeholder 1	c_ICB_shc	0.458	0.347	0.524	0.137	0.55
Real stakeholder 1	d_ICB_cmc	0.497	0.394	0.552	0.299	0.574
Real stakeholder 1	e_PCB	0.418	0.336	0.470	0.227	0.492
Real stakeholder 2	a_VB_shc	0.669	0.537	0.772	0.279	0.865
Real stakeholder 2	b_VB_cmc	0.691	0.555	0.796	0.404	0.842
Real stakeholder 2	c_ICB_shc	0.437	0.335	0.518	0.19	0.564
Real stakeholder 2	d_ICB_cmc	0.466	0.359	0.547	0.25	0.59
Real stakeholder 2	e_PCB	0.450	0.364	0.527	0.26	0.573
Real stakeholder 3	a_VB_shc	0.708	0.617	0.778	0.367	0.854
Real stakeholder 3	b_VB_cmc	0.731	0.642	0.802	0.526	0.838
Real stakeholder 3	c_ICB_shc	0.408	0.344	0.455	0.197	0.487
Real stakeholder 3	d_ICB_cmc	0.431	0.365	0.479	0.29	0.508
Real stakeholder 3	e_PCB	0.372	0.323	0.415	0.242	0.443

Note: Overall values for each option and each stakeholder using stakeholder value functions and a non-additive aggregation model with γ=0.2 for scenario 4. Values normalized to the interval [0, 1], with higher values indicating better overall performance aligned with individual preferences. Uncertainty in the predictions was accounted for by 2,000 Monte Carlo simulations. Options: [a_VB_shc] Vaginal birth with standard hospital care; [b_VB_cmc] Vaginal birth with continuous midwifery care; [c_ICB_shc] Intrapartum cesarean birth with standard hospital care; [d_ICB_cmc] Intrapartum cesarean birth with continuous midwifery care; [e_PCB] Prelabor cesarean birth.

S3-2. Cost-benefit visualizations

S3-2a. Cost-benefit visualizations, scenario 1

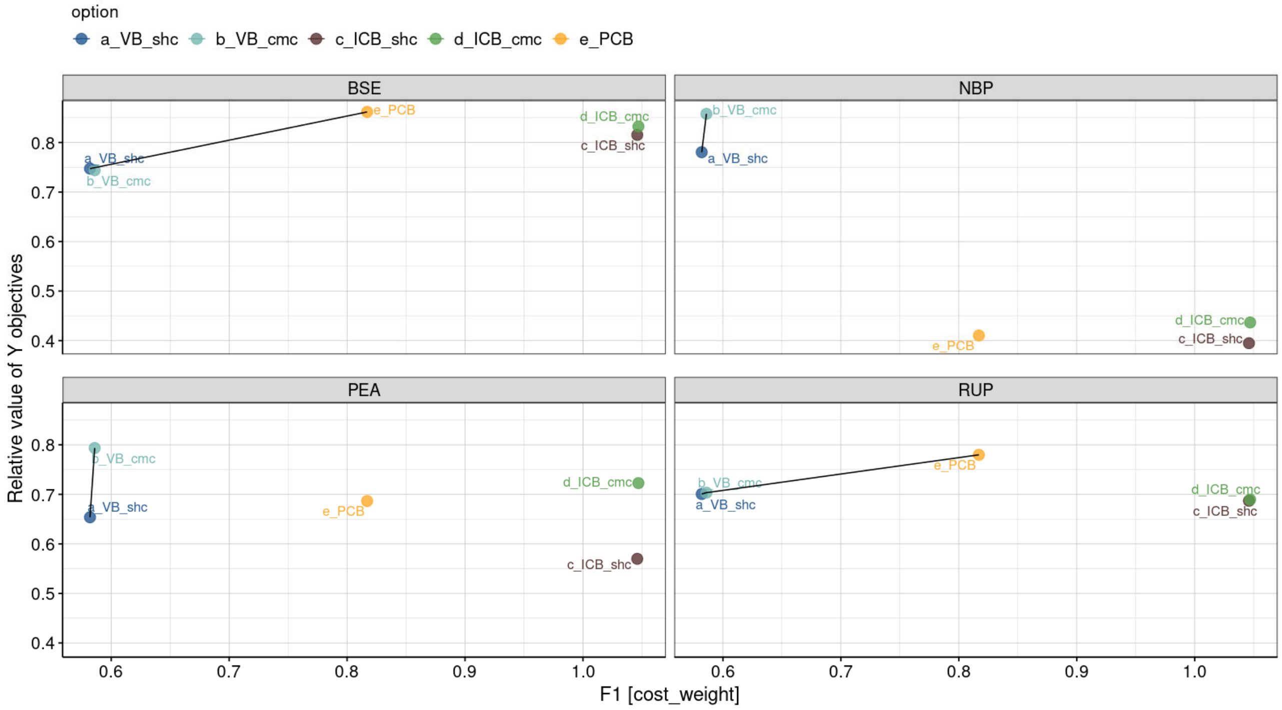

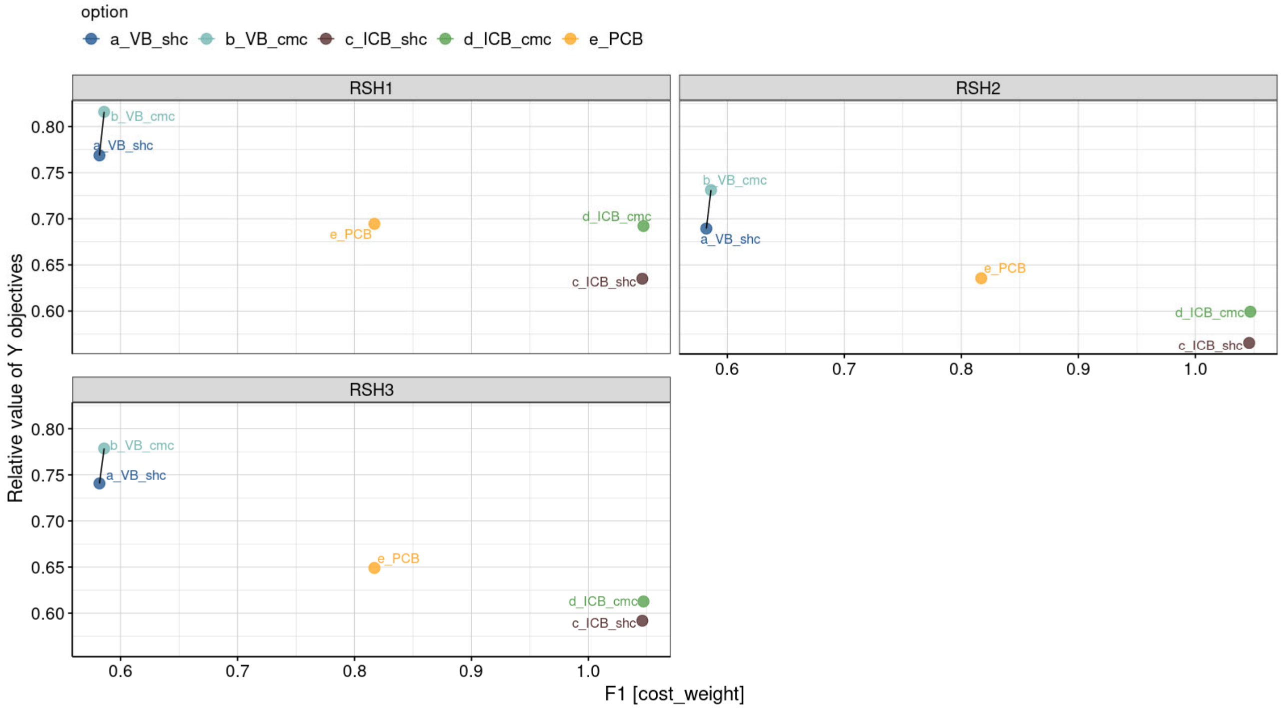

Note: Graphs by ValueDecisions (9). Cost-benefit visualizations with efficient frontiers for scenario 1. X-axis: Predicted average SwissDRG cost weight (attribute F1); y-axis: Relative value aggregated over all other goals (A1-E1). Options: [a_VB_shc] Vaginal birth with standard hospital care; [b_VB_cmc] Vaginal birth with continuous midwifery care; [c_ICB_shc] Intrapartum cesarean birth with standard hospital care; [d_ICB_cmc] Intrapartum cesarean birth with continuous midwifery care; [e_PCB] Prelabor cesarean birth. BSE: Hypothetical stakeholder biomedical safety enthusiast; NBP: Hypothetical stakeholder natural birth proponent; PEA: Hypothetical stakeholder psychosocial experience advocate; RUP: Hypothetical stakeholder resource utilization pragmatist. RSH1: Real stakeholder 1; RSH2: Real stakeholder 2; RSH3: Real stakeholder 3.

S3-2b. Cost-benefit visualizations, scenario 2

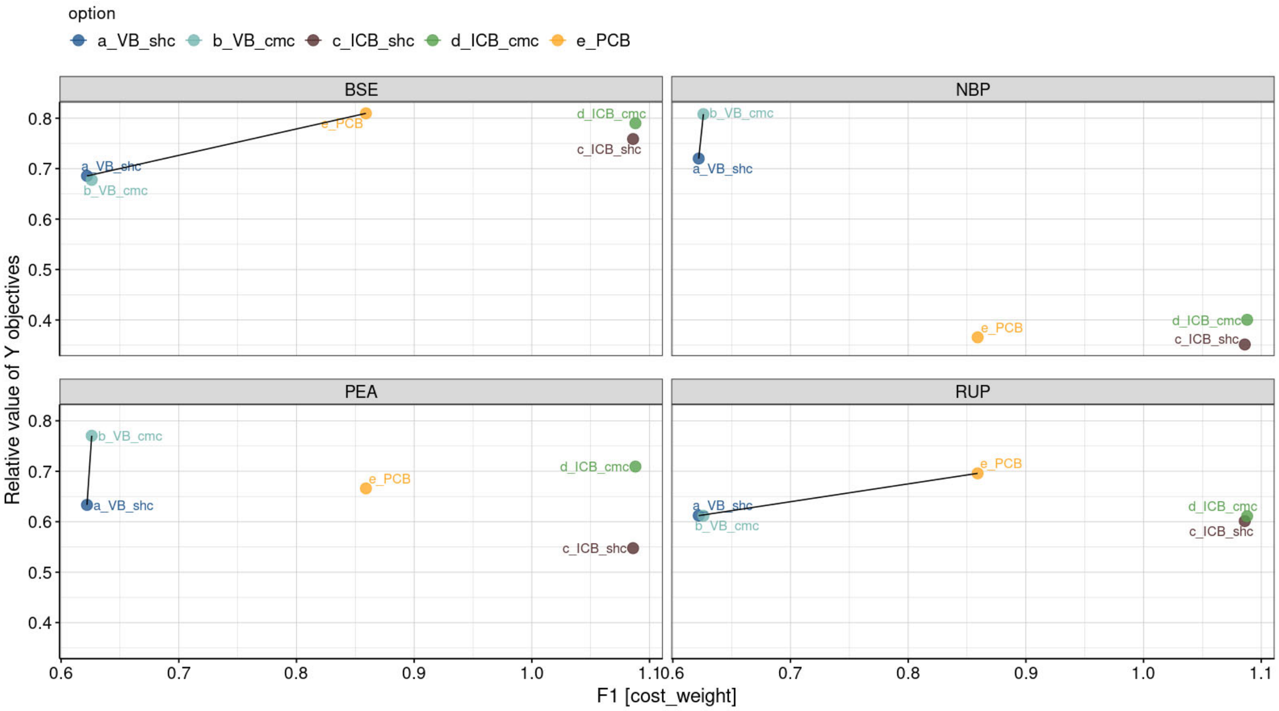

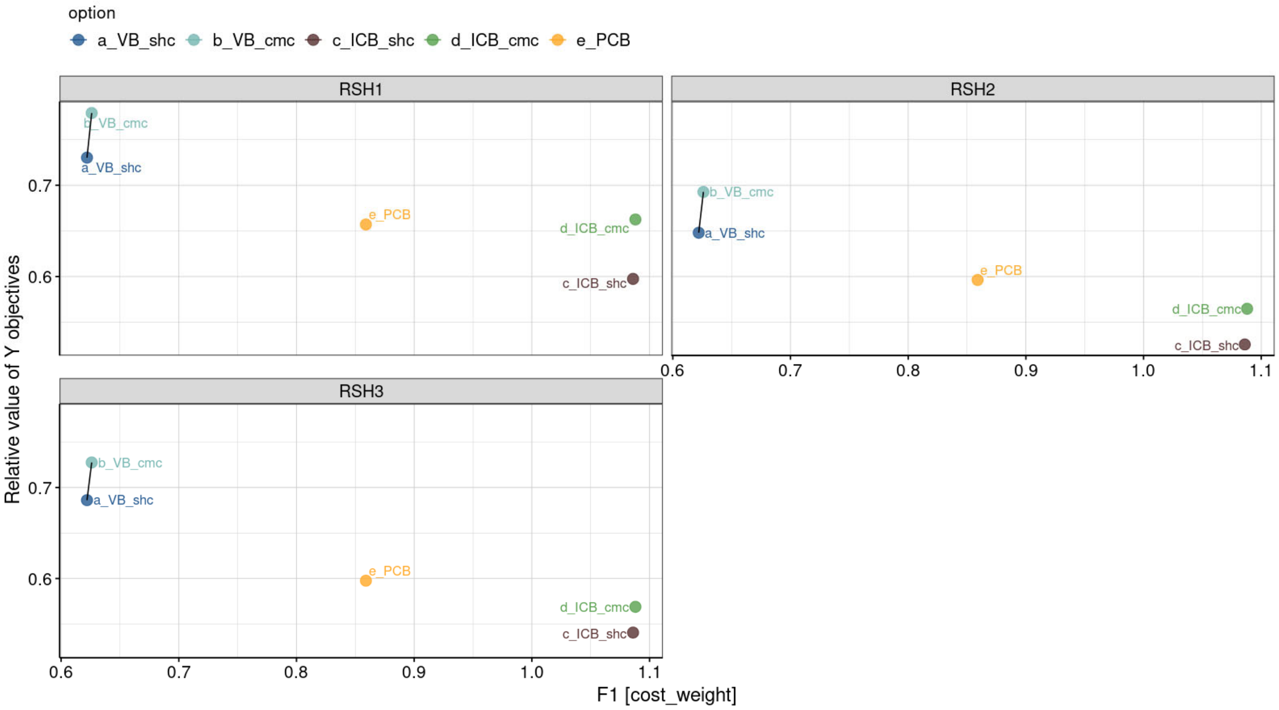

Note: Graphs by ValueDecisions (9). Cost-benefit visualizations with efficient frontiers for scenario 2. X-axis: Predicted average SwissDRG cost weight (attribute F1); y-axis: Relative value aggregated over all other goals (A1-E1). Options: [a_VB_shc] Vaginal birth with standard hospital care; [b_VB_cmc] Vaginal birth with continuous midwifery care; [c_ICB_shc] Intrapartum cesarean birth with standard hospital care; [d_ICB_cmc] Intrapartum cesarean birth with continuous midwifery care; [e_PCB] Prelabor cesarean birth. BSE: Hypothetical stakeholder biomedical safety enthusiast; NBP: Hypothetical stakeholder natural birth proponent; PEA: Hypothetical stakeholder psychosocial experience advocate; RUP: Hypothetical stakeholder resource utilization pragmatist. RSH1: Real stakeholder 1; RSH2: Real stakeholder 2; RSH3: Real stakeholder 3.

S3-2c. Cost-benefit visualizations, scenario 3

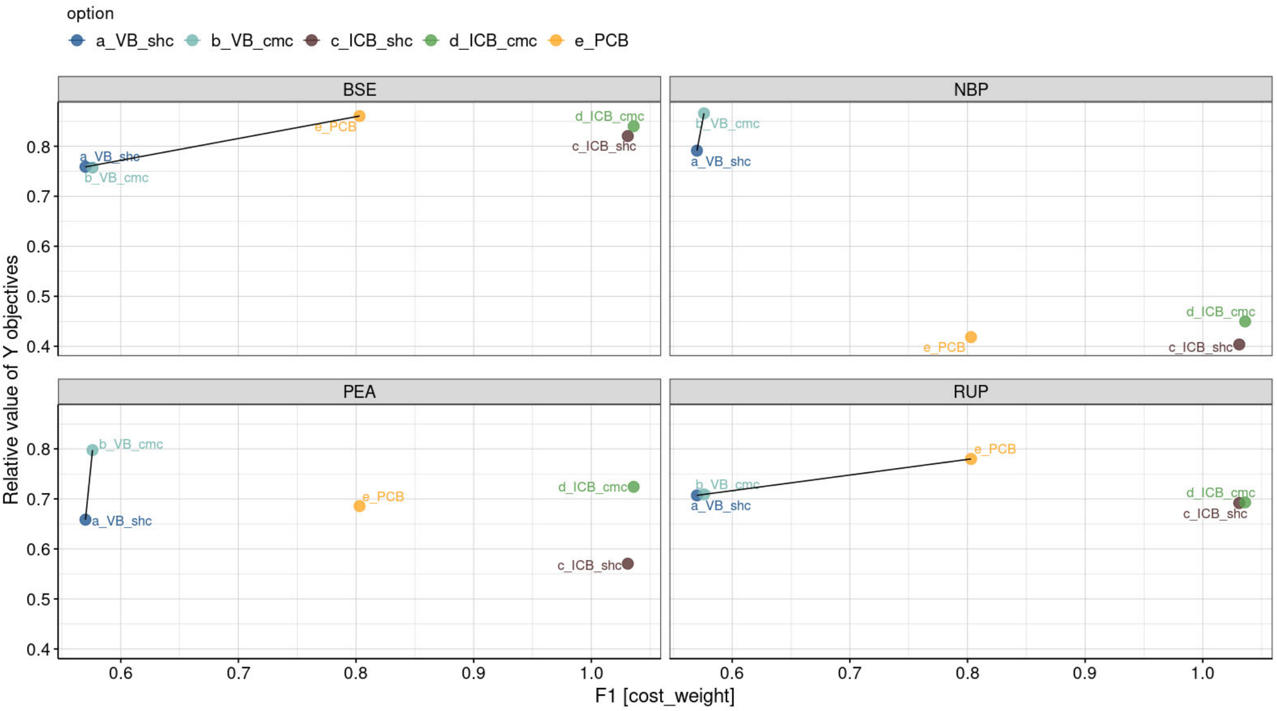

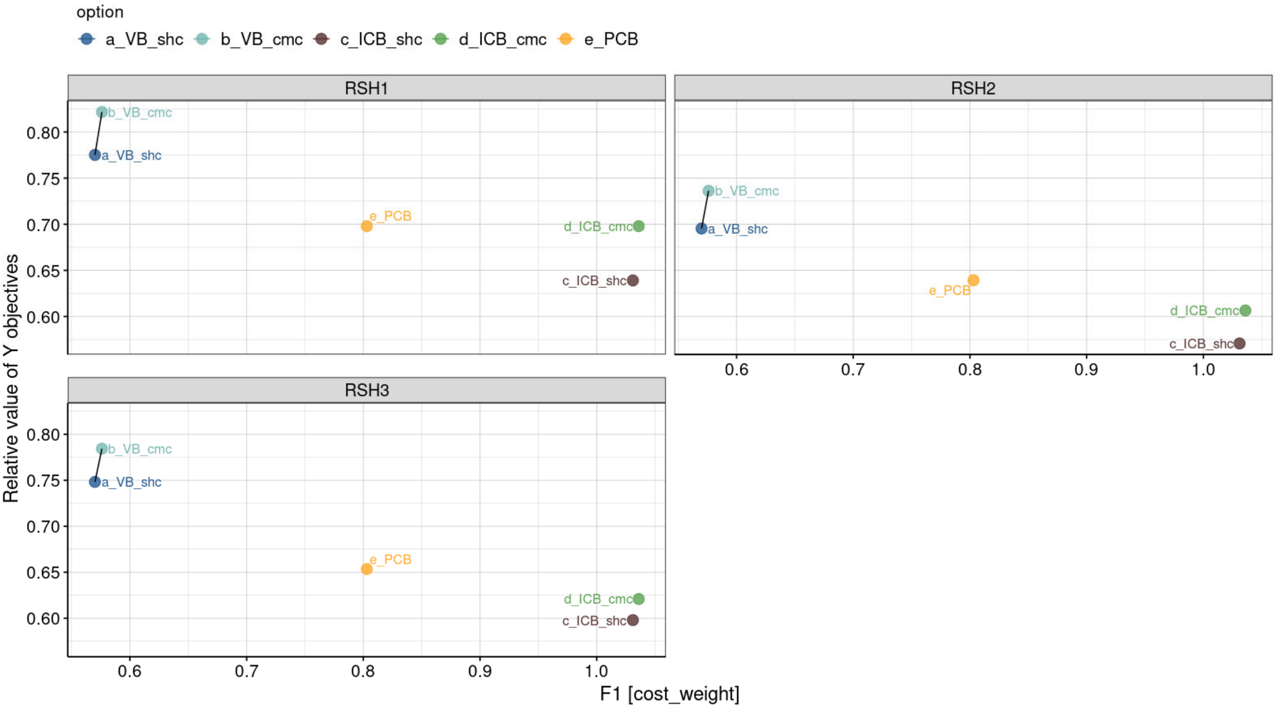

Note: Graphs by ValueDecisions (9). Cost-benefit visualizations with efficient frontiers for scenario 3. X-axis: Predicted average SwissDRG cost weight (attribute F1); y-axis: Relative value aggregated over all other goals (A1-E1). Options: [a_VB_shc] Vaginal birth with standard hospital care; [b_VB_cmc] Vaginal birth with continuous midwifery care; [c_ICB_shc] Intrapartum cesarean birth with standard hospital care; [d_ICB_cmc] Intrapartum cesarean birth with continuous midwifery care; [e_PCB] Prelabor cesarean birth. BSE: Hypothetical stakeholder biomedical safety enthusiast; NBP: Hypothetical stakeholder natural birth proponent; PEA: Hypothetical stakeholder psychosocial experience advocate; RUP: Hypothetical stakeholder resource utilization pragmatist. RSH1: Real stakeholder 1; RSH2: Real stakeholder 2; RSH3: Real stakeholder 3.

S3-2d. Cost-benefit visualizations, scenario 4

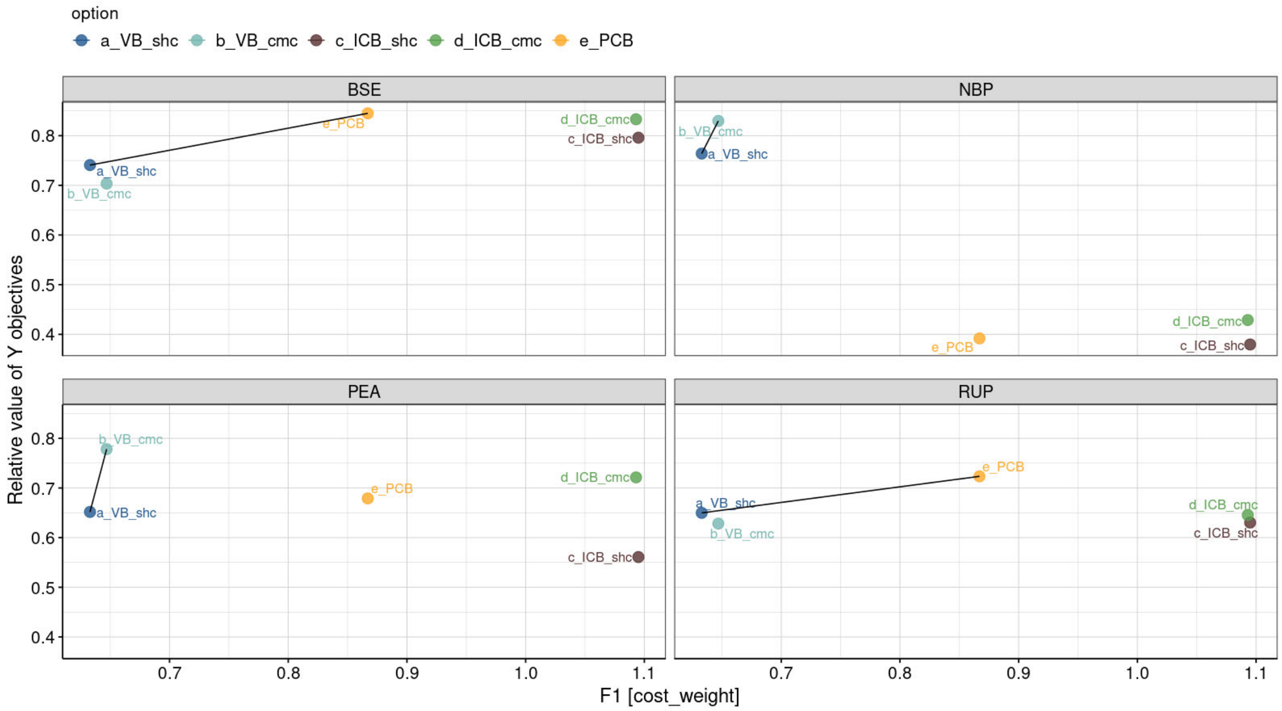

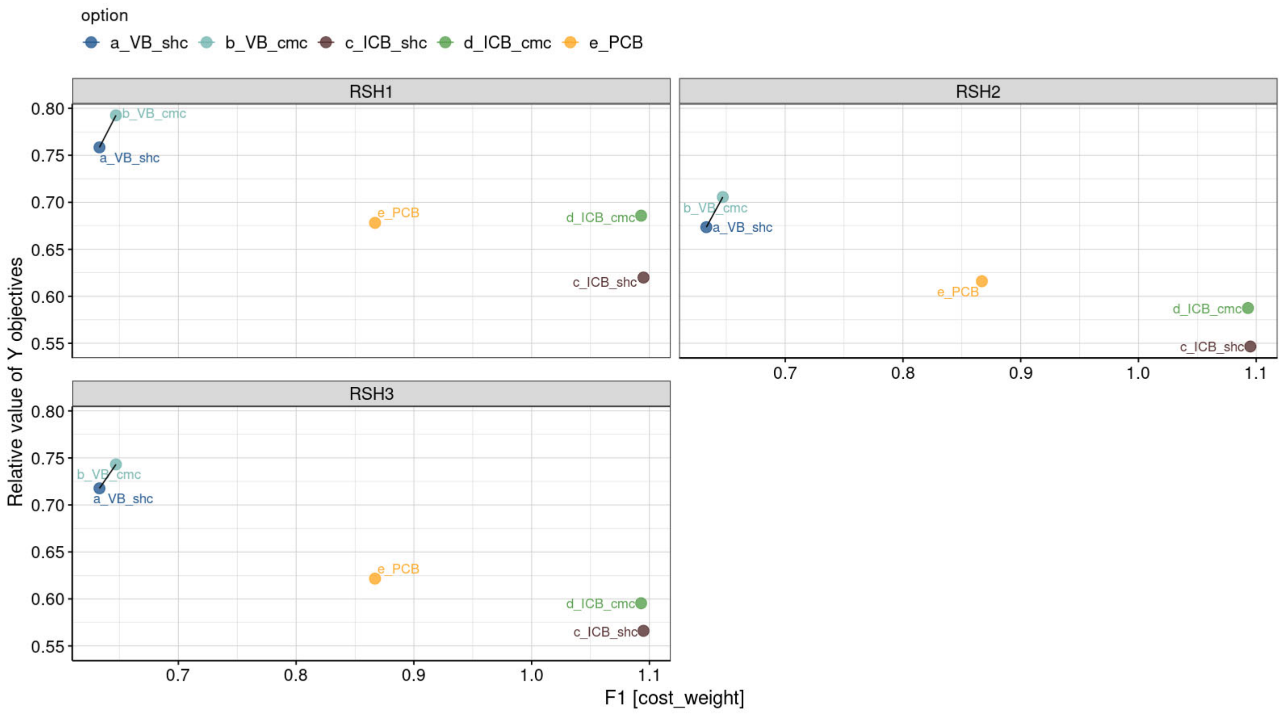

Note: Graphs by ValueDecisions (9). Cost-benefit visualizations with efficient frontiers for scenario 4. X-axis: Predicted average SwissDRG cost weight (attribute F1); y-axis: Relative value aggregated over all other goals (A1-E1). Options: [a_VB_shc] Vaginal birth with standard hospital care; [b_VB_cmc] Vaginal birth with continuous midwifery care; [c_ICB_shc] Intrapartum cesarean birth with standard hospital care; [d_ICB_cmc] Intrapartum cesarean birth with continuous midwifery care; [e_PCB] Prelabor cesarean birth. BSE: Hypothetical stakeholder biomedical safety enthusiast; NBP: Hypothetical stakeholder natural birth proponent; PEA: Hypothetical stakeholder psychosocial experience advocate; RUP: Hypothetical stakeholder resource utilization pragmatist. RSH1: Real stakeholder 1; RSH2: Real stakeholder 2; RSH3: Real stakeholder 3.

S3-3. Sensitivity analyses: Real stakeholder weights

S3-3a. Sensitivity analyses of real stakeholder weights, scenario 1

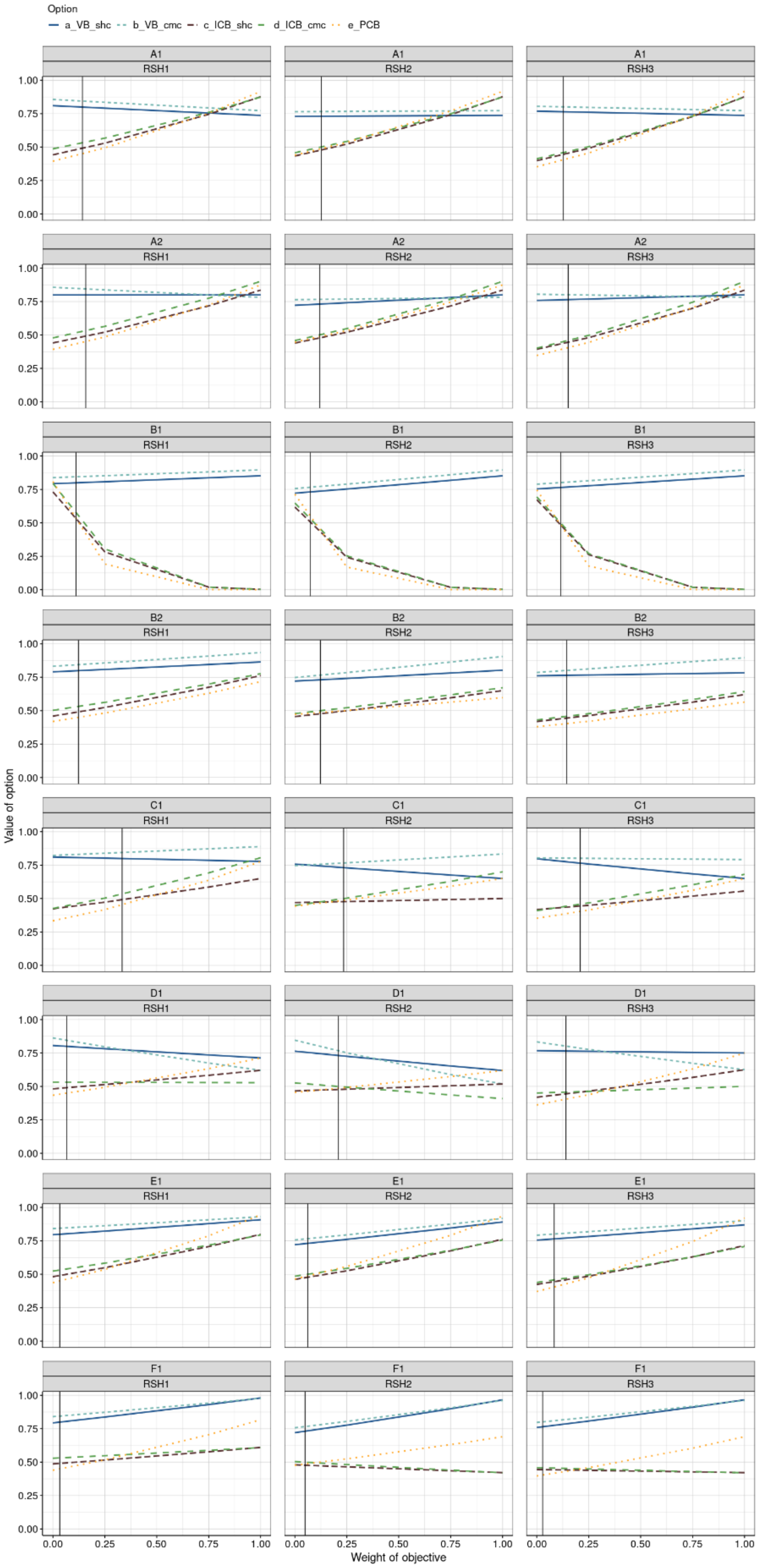

Note: Graph by ValueDecisions (9). Sensitivity analyses of real stakeholder weights for scenario 1. Options: [a_VB_shc] Vaginal birth with standard hospital care; [b_VB_cmc] Vaginal birth with continuous midwifery care; [c_ICB_shc] Intrapartum cesarean birth with standard hospital care; [d_ICB_cmc] Intrapartum cesarean birth with continuous midwifery care; [e_PCB] Prelabor cesarean birth. Goals/attributes: A1: Low maternal complication rates/weighted composite index; A2: Low neonatal complication rates/weighted composite index; B1: Physiological labor and childbirth processes/weighted composite index; B2: Positive initiation of bonding and breastfeeding after childbirth/exclusive breastfeeding at hospital discharge; C1: Positive psychosocial experience of care interactions and events/expert assessment; D1: Low physical strain for care providers/expert assessment; E1: Low resource use in care setting/clinical indicator score; F1: Low direct costs to the healthcare system/SwissDRG cost weight. RSH1: Real stakeholder 1; RSH2: Real stakeholder 2; RSH3: Real stakeholder 3.

S3-3b. Sensitivity analyses of real stakeholder weights, scenario 2

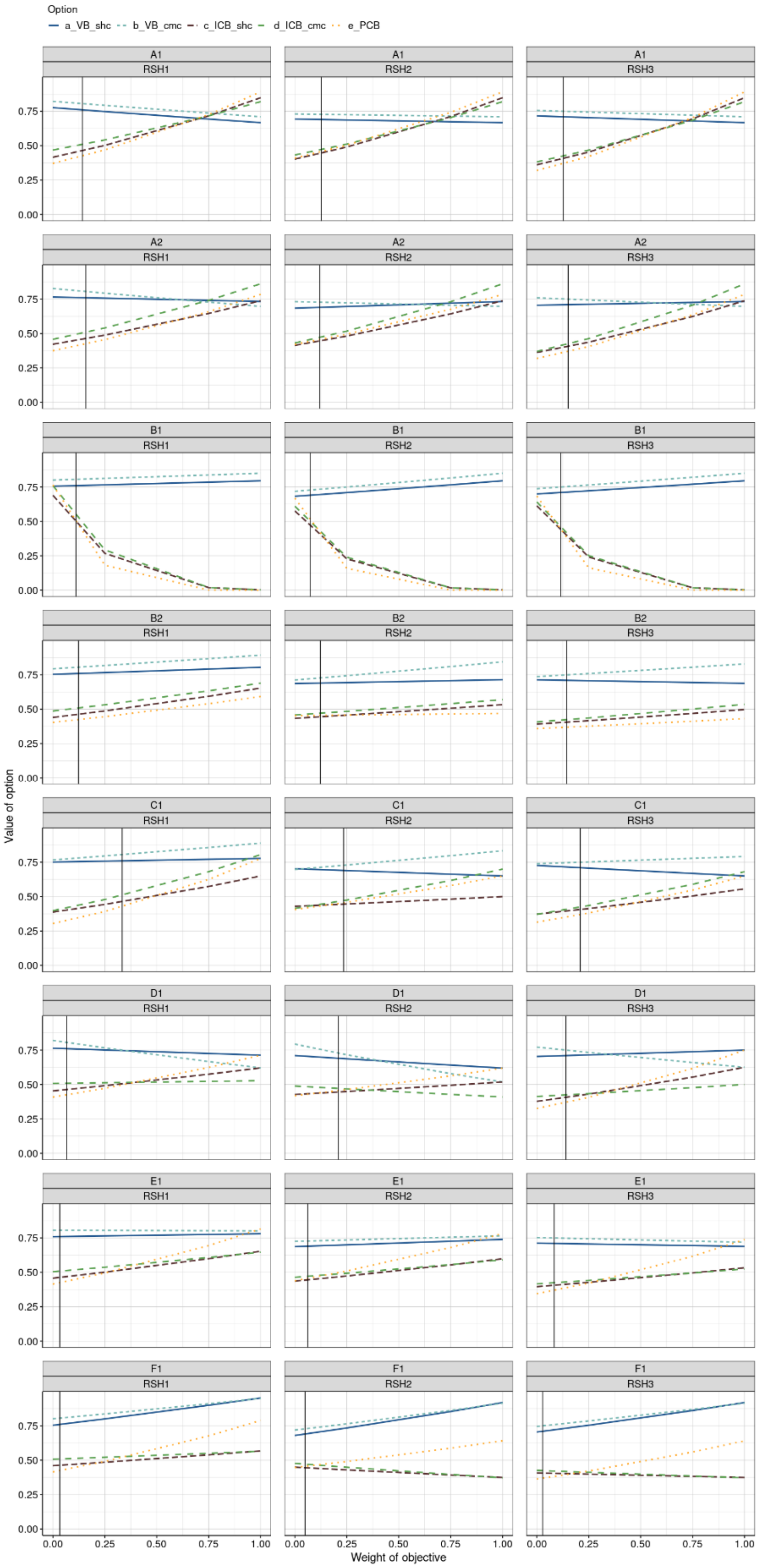

Note: Graph by ValueDecisions (9). Sensitivity analyses of real stakeholder weights for scenario 2. Options: [a_VB_shc] Vaginal birth with standard hospital care; [b_VB_cmc] Vaginal birth with continuous midwifery care; [c_ICB_shc] Intrapartum cesarean birth with standard hospital care; [d_ICB_cmc] Intrapartum cesarean birth with continuous midwifery care; [e_PCB] Prelabor cesarean birth. Goals/attributes: A1: Low maternal complication rates/weighted composite index; A2: Low neonatal complication rates/weighted composite index; B1: Physiological labor and childbirth processes/weighted composite index; B2: Positive initiation of bonding and breastfeeding after childbirth/exclusive breastfeeding at hospital discharge; C1: Positive psychosocial experience of care interactions and events/expert assessment; D1: Low physical strain for care providers/expert assessment; E1: Low resource use in care setting/clinical indicator score; F1: Low direct costs to the healthcare system/SwissDRG cost weight. RSH1: Real stakeholder 1; RSH2: Real stakeholder 2; RSH3: Real stakeholder 3.

S3-3c. Sensitivity analyses of real stakeholder weights, scenario 3

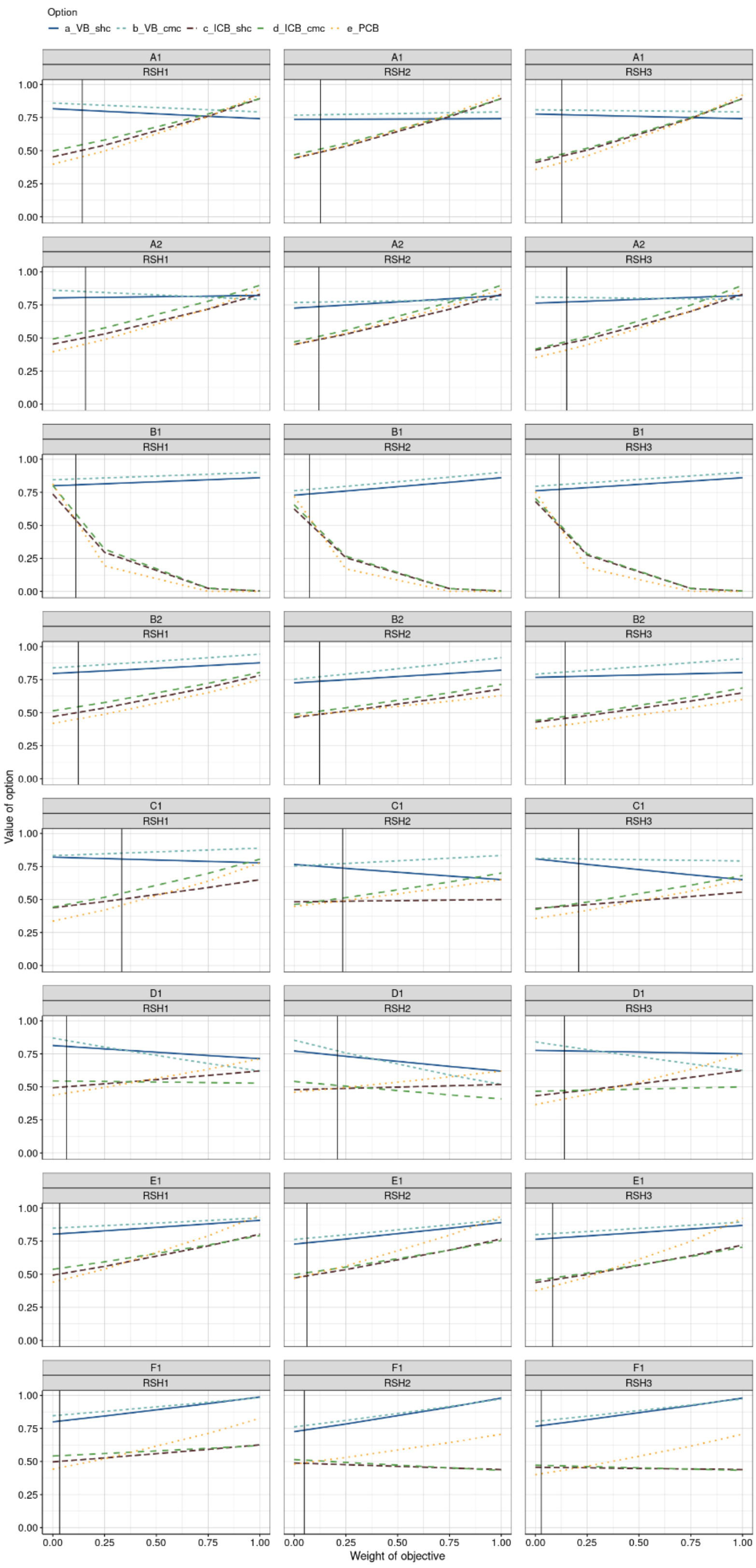

Note: Graph by ValueDecisions (9). Sensitivity analyses of real stakeholder weights for scenario 3. Options: [a_VB_shc] Vaginal birth with standard hospital care; [b_VB_cmc] Vaginal birth with continuous midwifery care; [c_ICB_shc] Intrapartum cesarean birth with standard hospital care; [d_ICB_cmc] Intrapartum cesarean birth with continuous midwifery care; [e_PCB] Prelabor cesarean birth. Goals/attributes: A1: Low maternal complication rates/weighted composite index; A2: Low neonatal complication rates/weighted composite index; B1: Physiological labor and childbirth processes/weighted composite index; B2: Positive initiation of bonding and breastfeeding after childbirth/exclusive breastfeeding at hospital discharge; C1: Positive psychosocial experience of care interactions and events/expert assessment; D1: Low physical strain for care providers/expert assessment; E1: Low resource use in care setting/clinical indicator score; F1: Low direct costs to the healthcare system/SwissDRG cost weight. RSH1: Real stakeholder 1; RSH2: Real stakeholder 2; RSH3: Real stakeholder 3.

S3-3d. Sensitivity analyses of real stakeholder weights, scenario 4

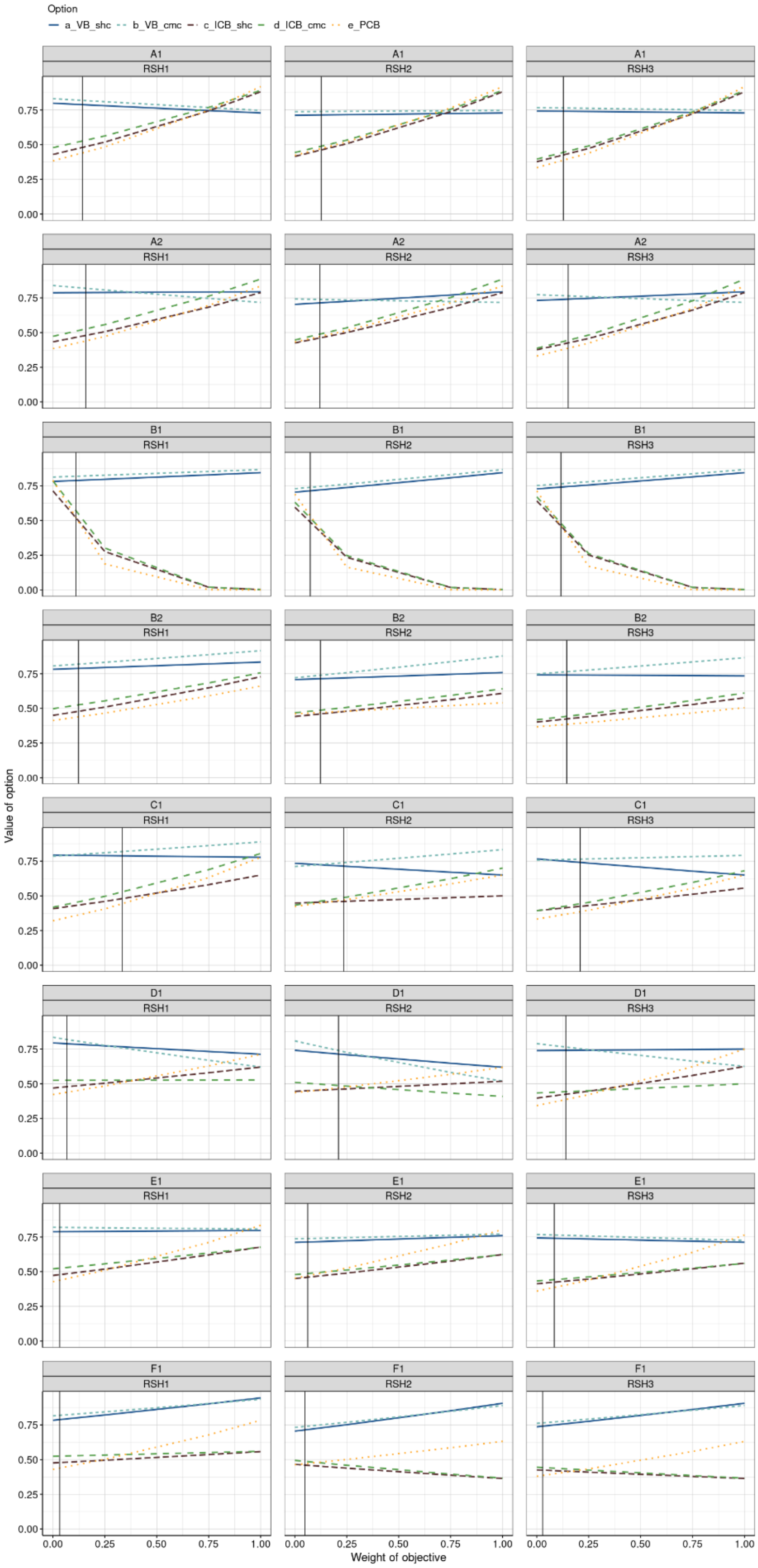

Note: Graph by ValueDecisions (9). Sensitivity analyses of real stakeholder weights for scenario 4. Options: [a_VB_shc] Vaginal birth with standard hospital care; [b_VB_cmc] Vaginal birth with continuous midwifery care; [c_ICB_shc] Intrapartum cesarean birth with standard hospital care; [d_ICB_cmc] Intrapartum cesarean birth with continuous midwifery care; [e_PCB] Prelabor cesarean birth. Goals/attributes: A1: Low maternal complication rates/weighted composite index; A2: Low neonatal complication rates/weighted composite index; B1: Physiological labor and childbirth processes/weighted composite index; B2: Positive initiation of bonding and breastfeeding after childbirth/exclusive breastfeeding at hospital discharge; C1: Positive psychosocial experience of care interactions and events/expert assessment; D1: Low physical strain for care providers/expert assessment; E1: Low resource use in care setting/clinical indicator score; F1: Low direct costs to the healthcare system/SwissDRG cost weight. RSH1: Real stakeholder 1; RSH2: Real stakeholder 2; RSH3: Real stakeholder 3.

S3-4. Sensitivity analyses: Overall values of childbirth options with value functions curvature *c*=2 and γ=0.2

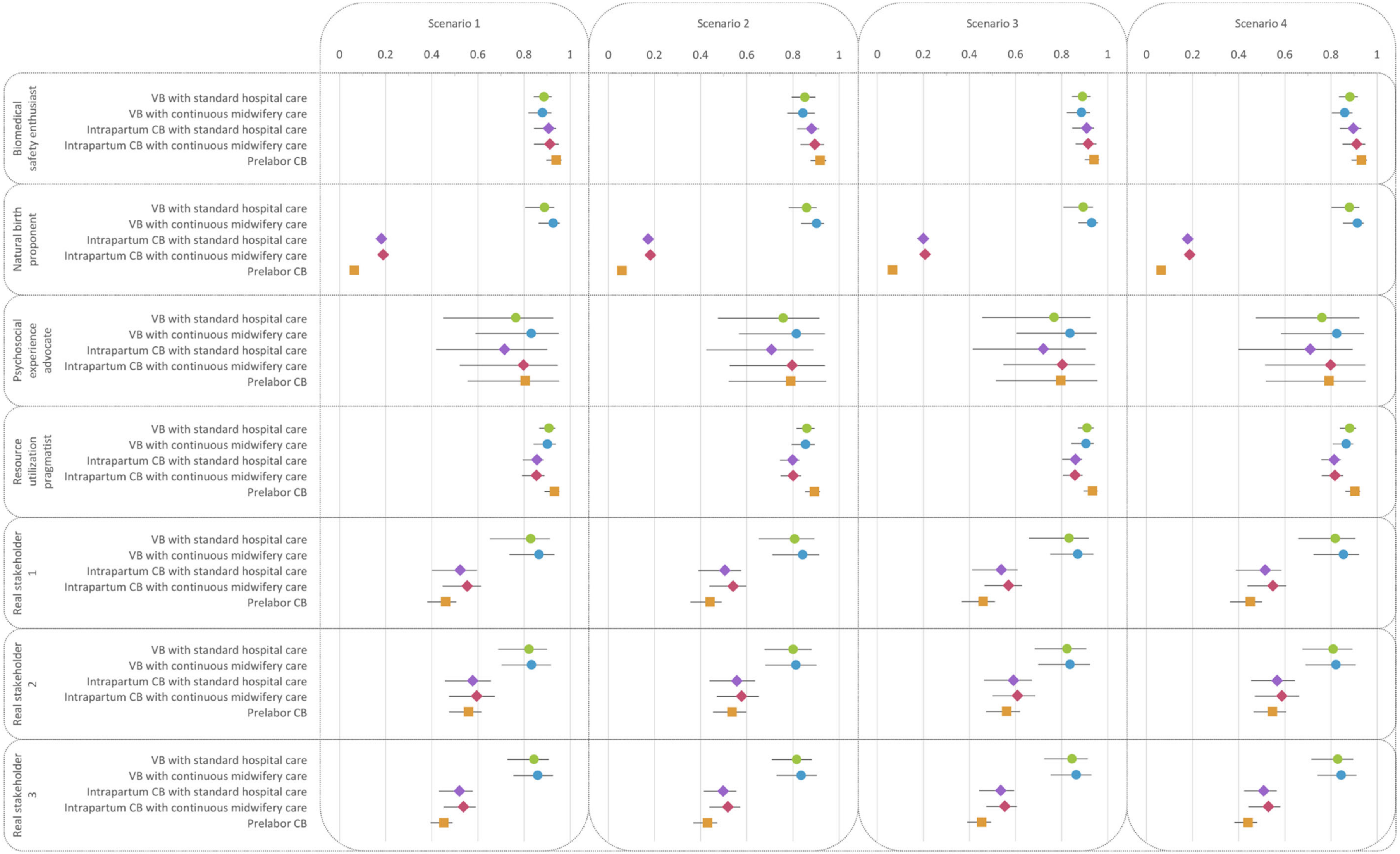

Note: Mean overall values for each option and each stakeholder in each scenario using value functions' curvature *c*=2 and a non-additive aggregation model with γ=0.2. Values normalized to the interval [0, 1], with higher values indicating better overall performance aligned with individual preferences. Uncertainty in the predictions was accounted for by 2,000 Monte Carlo simulations. Error bars indicate the 5% and 95% quantiles of the overall values. Scenarios: (1) Women with no cesarean birth history or comorbidities; (2) Women with no cesarean birth history but comorbidities; (3) Women with a cesarean birth history but no comorbidities; (4) Women with a cesarean birth history and comorbidities. Childbirth options: ● Vaginal birth (VB) with standard hospital care; ● Vaginal birth (VB) with continuous midwifery care; ◆ Intrapartum cesarean birth (CB) with standard hospital care; ◆ Intrapartum cesarean birth (CB) with continuous midwifery care; ■ Prelabor cesarean birth (CB).

S3-4a. Overall values of childbirth options with value functions curvature *c*=2 and γ=0.2, scenario 1

Stakeholder	Option	Mean overall value	5% quantile of overall value	95% quantile of overall value	Minimum	Maximum
Biomedical safety enthusiast	a_VB_shc	0.885	0.842	0.92	0.783	0.95
Biomedical safety enthusiast	b_VB_cmc	0.879	0.818	0.919	0.683	0.955
Biomedical safety enthusiast	c_ICB_shc	0.906	0.843	0.938	0.719	0.953
Biomedical safety enthusiast	d_ICB_cmc	0.911	0.843	0.95	0.729	0.966
Biomedical safety enthusiast	e_PCB	0.938	0.896	0.962	0.857	0.971
Natural birth proponent	a_VB_shc	0.888	0.804	0.931	0.635	0.95
Natural birth proponent	b_VB_cmc	0.925	0.863	0.955	0.779	0.968
Natural birth proponent	c_ICB_shc	0.181	0.157	0.196	0.113	0.205
Natural birth proponent	d_ICB_cmc	0.189	0.167	0.203	0.139	0.211
Natural birth proponent	e_PCB	0.064	0.056	0.07	0.046	0.074
Psychosocial experience advocate	a_VB_shc	0.764	0.449	0.926	0.107	0.968
Psychosocial experience advocate	b_VB_cmc	0.83	0.59	0.95	0.354	0.976
Psychosocial experience advocate	c_ICB_shc	0.715	0.419	0.901	0.104	0.942
Psychosocial experience advocate	d_ICB_cmc	0.797	0.522	0.946	0.254	0.977
Psychosocial experience advocate	e_PCB	0.805	0.555	0.952	0.251	0.985
Resource utilization pragmatist	a_VB_shc	0.907	0.866	0.934	0.821	0.956
Resource utilization pragmatist	b_VB_cmc	0.901	0.841	0.937	0.701	0.959
Resource utilization pragmatist	c_ICB_shc	0.855	0.794	0.886	0.676	0.897
Resource utilization pragmatist	d_ICB_cmc	0.853	0.791	0.889	0.684	0.902
Resource utilization pragmatist	e_PCB	0.931	0.889	0.954	0.851	0.963
Real stakeholder 1	a_VB_shc	0.828	0.652	0.912	0.36	0.945
Real stakeholder 1	b_VB_cmc	0.864	0.736	0.932	0.536	0.955
Real stakeholder 1	c_ICB_shc	0.523	0.401	0.596	0.217	0.626
Real stakeholder 1	d_ICB_cmc	0.553	0.447	0.613	0.315	0.634
Real stakeholder 1	e_PCB	0.459	0.38	0.507	0.26	0.529
Real stakeholder 2	a_VB_shc	0.821	0.688	0.9	0.442	0.939
Real stakeholder 2	b_VB_cmc	0.832	0.703	0.917	0.457	0.951
Real stakeholder 2	c_ICB_shc	0.577	0.457	0.657	0.315	0.702
Real stakeholder 2	d_ICB_cmc	0.594	0.475	0.674	0.33	0.706
Real stakeholder 2	e_PCB	0.558	0.475	0.615	0.374	0.645
Real stakeholder 3	a_VB_shc	0.842	0.728	0.907	0.497	0.939
Real stakeholder 3	b_VB_cmc	0.859	0.754	0.925	0.548	0.95
Real stakeholder 3	c_ICB_shc	0.52	0.431	0.578	0.304	0.612
Real stakeholder 3	d_ICB_cmc	0.537	0.451	0.591	0.341	0.615
Real stakeholder 3	e_PCB	0.451	0.395	0.49	0.319	0.51

Note: Overall values for each option and each stakeholder using value functions' curvature *c*=2 and a non-additive aggregation model with γ=0.2 for scenario 1. Values normalized to the interval [0, 1], with higher values indicating better overall performance aligned with individual preferences. Uncertainty in the predictions was accounted for by 2,000 Monte Carlo simulations. Options: [a_VB_shc] Vaginal birth with standard hospital care; [b_VB_cmc] Vaginal birth with continuous midwifery care; [c_ICB_shc] Intrapartum cesarean birth with standard hospital care; [d_ICB_cmc] Intrapartum cesarean birth with continuous midwifery care; [e_PCB] Prelabor cesarean birth.

S3-4b. Overall values of childbirth options with value functions curvature *c*=2 and γ=0.2, scenario 2

Stakeholder	Option	Mean overall value	5% quantile of overall value	95% quantile of overall value	Minimum	Maximum
Biomedical safety enthusiast	a_VB_shc	0.851	0.795	0.898	0.72	0.937
Biomedical safety enthusiast	b_VB_cmc	0.843	0.776	0.895	0.65	0.937
Biomedical safety enthusiast	c_ICB_shc	0.88	0.819	0.914	0.666	0.941
Biomedical safety enthusiast	d_ICB_cmc	0.894	0.833	0.936	0.714	0.964
Biomedical safety enthusiast	e_PCB	0.917	0.877	0.944	0.837	0.96
Natural birth proponent	a_VB_shc	0.859	0.782	0.903	0.658	0.927
Natural birth proponent	b_VB_cmc	0.902	0.836	0.935	0.769	0.948
Natural birth proponent	c_ICB_shc	0.172	0.149	0.189	0.103	0.201
Natural birth proponent	d_ICB_cmc	0.182	0.160	0.196	0.139	0.203
Natural birth proponent	e_PCB	0.059	0.049	0.066	0.032	0.071
Psychosocial experience advocate	a_VB_shc	0.757	0.475	0.915	0.167	0.953
Psychosocial experience advocate	b_VB_cmc	0.815	0.566	0.939	0.37	0.97
Psychosocial experience advocate	c_ICB_shc	0.707	0.425	0.889	0.084	0.933
Psychosocial experience advocate	d_ICB_cmc	0.796	0.526	0.939	0.32	0.968
Psychosocial experience advocate	e_PCB	0.79	0.522	0.944	0.266	0.978
Resource utilization pragmatist	a_VB_shc	0.86	0.816	0.894	0.76	0.916
Resource utilization pragmatist	b_VB_cmc	0.855	0.795	0.895	0.687	0.921
Resource utilization pragmatist	c_ICB_shc	0.798	0.744	0.829	0.608	0.843
Resource utilization pragmatist	d_ICB_cmc	0.801	0.747	0.836	0.638	0.855
Resource utilization pragmatist	e_PCB	0.892	0.853	0.918	0.816	0.928
Real stakeholder 1	a_VB_shc	0.807	0.652	0.893	0.419	0.927
Real stakeholder 1	b_VB_cmc	0.842	0.711	0.914	0.58	0.944
Real stakeholder 1	c_ICB_shc	0.505	0.390	0.576	0.198	0.61
Real stakeholder 1	d_ICB_cmc	0.540	0.438	0.598	0.333	0.62
Real stakeholder 1	e_PCB	0.440	0.355	0.492	0.242	0.514
Real stakeholder 2	a_VB_shc	0.800	0.677	0.882	0.478	0.927
Real stakeholder 2	b_VB_cmc	0.812	0.681	0.902	0.468	0.942
Real stakeholder 2	c_ICB_shc	0.556	0.439	0.636	0.303	0.677
Real stakeholder 2	d_ICB_cmc	0.577	0.470	0.653	0.298	0.687
Real stakeholder 2	e_PCB	0.536	0.454	0.598	0.319	0.629
Real stakeholder 3	a_VB_shc	0.816	0.709	0.883	0.529	0.919
Real stakeholder 3	b_VB_cmc	0.835	0.729	0.904	0.558	0.938
Real stakeholder 3	c_ICB_shc	0.497	0.414	0.555	0.288	0.588
Real stakeholder 3	d_ICB_cmc	0.518	0.438	0.571	0.317	0.595
Real stakeholder 3	e_PCB	0.429	0.369	0.472	0.274	0.495

Note: Overall values for each option and each stakeholder using value functions' curvature *c*=2 and a non-additive aggregation model with γ=0.2 for scenario 2. Values normalized to the interval [0, 1], with higher values indicating better overall performance aligned with individual preferences. Uncertainty in the predictions was accounted for by 2,000 Monte Carlo simulations. Options: [a_VB_shc] Vaginal birth with standard hospital care; [b_VB_cmc] Vaginal birth with continuous midwifery care; [c_ICB_shc] Intrapartum cesarean birth with standard hospital care; [d_ICB_cmc] Intrapartum cesarean birth with continuous midwifery care; [e_PCB] Prelabor cesarean birth.

S3-4c. Overall values of childbirth options with value functions curvature *c*=2 and γ=0.2, scenario 3

Stakeholder	Option	Mean overall value	5% quantile of overall value	95% quantile of overall value	Minimum	Maximum
Biomedical safety enthusiast	a_VB_shc	0.89	0.845	0.927	0.78	0.955
Biomedical safety enthusiast	b_VB_cmc	0.885	0.823	0.923	0.709	0.945
Biomedical safety enthusiast	c_ICB_shc	0.908	0.847	0.941	0.751	0.953
Biomedical safety enthusiast	d_ICB_cmc	0.915	0.861	0.951	0.753	0.964
Biomedical safety enthusiast	e_PCB	0.939	0.901	0.963	0.849	0.973
Natural birth proponent	a_VB_shc	0.893	0.808	0.936	0.564	0.951
Natural birth proponent	b_VB_cmc	0.93	0.872	0.958	0.787	0.967
Natural birth proponent	c_ICB_shc	0.199	0.172	0.214	0.126	0.222
Natural birth proponent	d_ICB_cmc	0.207	0.187	0.218	0.154	0.222
Natural birth proponent	e_PCB	0.065	0.056	0.07	0.046	0.073
Psychosocial experience advocate	a_VB_shc	0.767	0.455	0.927	0.052	0.965
Psychosocial experience advocate	b_VB_cmc	0.836	0.605	0.952	0.359	0.979
Psychosocial experience advocate	c_ICB_shc	0.72	0.414	0.905	0.11	0.943
Psychosocial experience advocate	d_ICB_cmc	0.802	0.547	0.945	0.258	0.974
Psychosocial experience advocate	e_PCB	0.796	0.515	0.956	0.289	0.984
Resource utilization pragmatist	a_VB_shc	0.909	0.870	0.939	0.82	0.956
Resource utilization pragmatist	b_VB_cmc	0.905	0.842	0.939	0.726	0.953
Resource utilization pragmatist	c_ICB_shc	0.859	0.802	0.889	0.707	0.9
Resource utilization pragmatist	d_ICB_cmc	0.857	0.806	0.891	0.702	0.9
Resource utilization pragmatist	e_PCB	0.933	0.895	0.957	0.845	0.967
Real stakeholder 1	a_VB_shc	0.831	0.659	0.918	0.283	0.948
Real stakeholder 1	b_VB_cmc	0.869	0.750	0.938	0.597	0.961
Real stakeholder 1	c_ICB_shc	0.537	0.411	0.609	0.237	0.641
Real stakeholder 1	d_ICB_cmc	0.569	0.465	0.628	0.325	0.648
Real stakeholder 1	e_PCB	0.459	0.367	0.511	0.277	0.528
Real stakeholder 2	a_VB_shc	0.823	0.683	0.907	0.402	0.94
Real stakeholder 2	b_VB_cmc	0.836	0.699	0.923	0.519	0.961
Real stakeholder 2	c_ICB_shc	0.59	0.463	0.672	0.253	0.711
Real stakeholder 2	d_ICB_cmc	0.608	0.501	0.686	0.364	0.714
Real stakeholder 2	e_PCB	0.56	0.472	0.620	0.351	0.644
Real stakeholder 3	a_VB_shc	0.844	0.724	0.914	0.447	0.942
Real stakeholder 3	b_VB_cmc	0.863	0.752	0.93	0.61	0.96
Real stakeholder 3	c_ICB_shc	0.535	0.441	0.594	0.279	0.625
Real stakeholder 3	d_ICB_cmc	0.553	0.473	0.607	0.365	0.628
Real stakeholder 3	e_PCB	0.452	0.390	0.493	0.307	0.511

Note: Overall values for each option and each stakeholder using value functions' curvature *c*=2 and a non-additive aggregation model with γ=0.2 for scenario 3. Values normalized to the interval [0, 1], with higher values indicating better overall performance aligned with individual preferences. Uncertainty in the predictions was accounted for by 2,000 Monte Carlo simulations. Options: [a_VB_shc] Vaginal birth with standard hospital care; [b_VB_cmc] Vaginal birth with continuous midwifery care; [c_ICB_shc] Intrapartum cesarean birth with standard hospital care; [d_ICB_cmc] Intrapartum cesarean birth with continuous midwifery care; [e_PCB] Prelabor cesarean birth.

S3-4d. Overall values of childbirth options with value functions curvature *c*=2 and γ=0.2, scenario 4

Stakeholder	Option	Mean overall value	5% quantile of overall value	95% quantile of overall value	Minimum	Maximum
Biomedical safety enthusiast	a_VB_shc	0.881	0.835	0.917	0.784	0.942
Biomedical safety enthusiast	b_VB_cmc	0.859	0.804	0.893	0.695	0.913
Biomedical safety enthusiast	c_ICB_shc	0.897	0.838	0.931	0.719	0.949
Biomedical safety enthusiast	d_ICB_cmc	0.911	0.850	0.948	0.748	0.964
Biomedical safety enthusiast	e_PCB	0.931	0.889	0.956	0.847	0.967
Natural birth proponent	a_VB_shc	0.879	0.803	0.924	0.607	0.947
Natural birth proponent	b_VB_cmc	0.914	0.852	0.942	0.777	0.948
Natural birth proponent	c_ICB_shc	0.178	0.153	0.193	0.096	0.201
Natural birth proponent	d_ICB_cmc	0.187	0.165	0.201	0.14	0.21
Natural birth proponent	e_PCB	0.062	0.053	0.068	0.04	0.073
Psychosocial experience advocate	a_VB_shc	0.760	0.473	0.924	0.1	0.961
Psychosocial experience advocate	b_VB_cmc	0.824	0.583	0.943	0.363	0.964
Psychosocial experience advocate	c_ICB_shc	0.710	0.400	0.894	0.05	0.937
Psychosocial experience advocate	d_ICB_cmc	0.798	0.514	0.948	0.277	0.977
Psychosocial experience advocate	e_PCB	0.791	0.517	0.950	0.261	0.98
Resource utilization pragmatist	a_VB_shc	0.880	0.838	0.908	0.787	0.926
Resource utilization pragmatist	b_VB_cmc	0.865	0.808	0.897	0.7	0.906
Resource utilization pragmatist	c_ICB_shc	0.813	0.758	0.843	0.653	0.861
Resource utilization pragmatist	d_ICB_cmc	0.817	0.760	0.853	0.667	0.866
Resource utilization pragmatist	e_PCB	0.903	0.862	0.927	0.821	0.937
Real stakeholder 1	a_VB_shc	0.818	0.658	0.906	0.324	0.948
Real stakeholder 1	b_VB_cmc	0.853	0.725	0.921	0.585	0.941
Real stakeholder 1	c_ICB_shc	0.514	0.388	0.585	0.159	0.613
Real stakeholder 1	d_ICB_cmc	0.548	0.437	0.606	0.33	0.624
Real stakeholder 1	e_PCB	0.449	0.362	0.501	0.245	0.518
Real stakeholder 2	a_VB_shc	0.809	0.676	0.893	0.361	0.945
Real stakeholder 2	b_VB_cmc	0.821	0.690	0.907	0.519	0.933
Real stakeholder 2	c_ICB_shc	0.566	0.453	0.644	0.257	0.678
Real stakeholder 2	d_ICB_cmc	0.586	0.470	0.662	0.334	0.69
Real stakeholder 2	e_PCB	0.545	0.464	0.606	0.345	0.631
Real stakeholder 3	a_VB_shc	0.828	0.715	0.897	0.428	0.942
Real stakeholder 3	b_VB_cmc	0.844	0.742	0.911	0.604	0.931
Real stakeholder 3	c_ICB_shc	0.508	0.423	0.565	0.25	0.589
Real stakeholder 3	d_ICB_cmc	0.528	0.442	0.581	0.354	0.601
Real stakeholder 3	e_PCB	0.439	0.381	0.481	0.29	0.499

Note: Overall values for each option and each stakeholder using value functions' curvature *c*=2 and a non-additive aggregation model with γ=0.2 for scenario 4. Values normalized to the interval [0, 1], with higher values indicating better overall performance aligned with individual preferences. Uncertainty in the predictions was accounted for by 2,000 Monte Carlo simulations. Options: [a_VB_shc] Vaginal birth with standard hospital care; [b_VB_cmc] Vaginal birth with continuous midwifery care; [c_ICB_shc] Intrapartum cesarean birth with standard hospital care; [d_ICB_cmc] Intrapartum cesarean birth with continuous midwifery care; [e_PCB] Prelabor cesarean birth.

S3-5. Sensitivity analyses: Overall values of childbirth options with linear value functions and γ=0.2

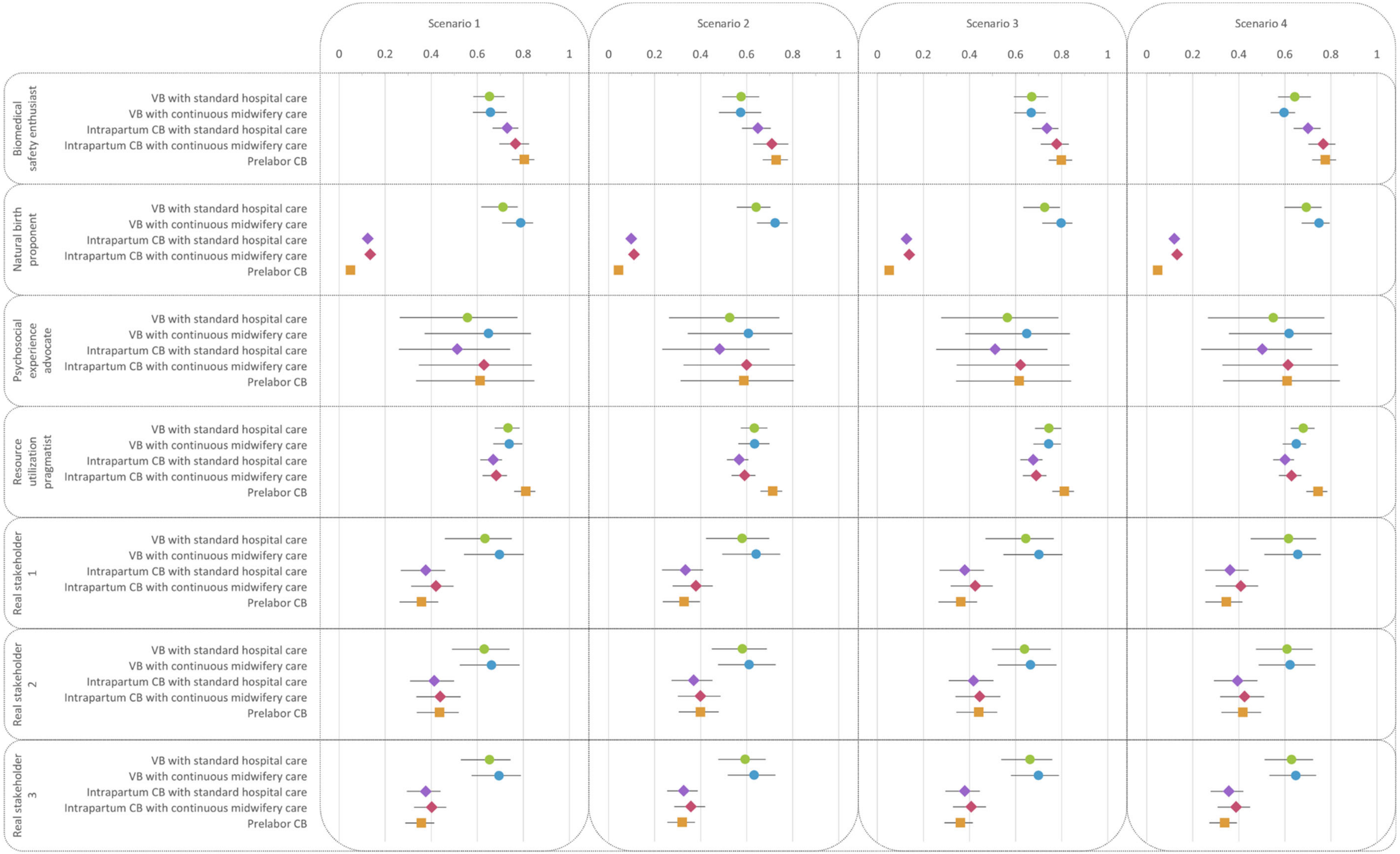

Note: Mean overall values for each option and each stakeholder in each scenario using linear value functions and a non-additive aggregation model with γ=0.2. Values normalized to the interval [0, 1], with higher values indicating better overall performance aligned with individual preferences. Uncertainty in the predictions was accounted for by 2,000 Monte Carlo simulations. Error bars indicate the 5% and 95% quantiles of the overall values. Scenarios: (1) Women with no cesarean birth history or comorbidities; (2) Women with no cesarean birth history but comorbidities; (3) Women with a cesarean birth history but no comorbidities; (4) Women with a cesarean birth history and comorbidities. Childbirth options: ● Vaginal birth (VB) with standard hospital care; ● Vaginal birth (VB) with continuous midwifery care; ◆ Intrapartum cesarean birth (CB) with standard hospital care; ◆ Intrapartum cesarean birth (CB) with continuous midwifery care; ■ Prelabor cesarean birth (CB).

S3-5a. Overall values of childbirth options with linear value functions and γ=0.2, scenario 1

Stakeholder	Option	Mean overall value	5% quantile of overall value	95% quantile of overall value	Minimum	Maximum
Biomedical safety enthusiast	a_VB_shc	0.652	0.583	0.719	0.503	0.801
Biomedical safety enthusiast	b_VB_cmc	0.657	0.581	0.728	0.496	0.809
Biomedical safety enthusiast	c_ICB_shc	0.729	0.666	0.778	0.572	0.816
Biomedical safety enthusiast	d_ICB_cmc	0.765	0.696	0.825	0.603	0.869
Biomedical safety enthusiast	e_PCB	0.803	0.750	0.848	0.706	0.89
Natural birth proponent	a_VB_shc	0.711	0.617	0.776	0.463	0.83
Natural birth proponent	b_VB_cmc	0.788	0.708	0.842	0.617	0.874
Natural birth proponent	c_ICB_shc	0.124	0.102	0.142	0.072	0.157
Natural birth proponent	d_ICB_cmc	0.135	0.115	0.149	0.095	0.157
Natural birth proponent	e_PCB	0.049	0.039	0.058	0.03	0.064
Psychosocial experience advocate	a_VB_shc	0.557	0.264	0.775	0.025	0.87
Psychosocial experience advocate	b_VB_cmc	0.648	0.371	0.834	0.191	0.905
Psychosocial experience advocate	c_ICB_shc	0.512	0.259	0.742	0.079	0.824
Psychosocial experience advocate	d_ICB_cmc	0.628	0.346	0.837	0.166	0.907
Psychosocial experience advocate	e_PCB	0.611	0.334	0.848	0.17	0.942
Resource utilization pragmatist	a_VB_shc	0.733	0.676	0.784	0.615	0.842
Resource utilization pragmatist	b_VB_cmc	0.738	0.67	0.796	0.58	0.841
Resource utilization pragmatist	c_ICB_shc	0.669	0.613	0.708	0.53	0.732
Resource utilization pragmatist	d_ICB_cmc	0.682	0.623	0.729	0.532	0.766
Resource utilization pragmatist	e_PCB	0.81	0.760	0.852	0.712	0.879
Real stakeholder 1	a_VB_shc	0.633	0.459	0.75	0.202	0.818
Real stakeholder 1	b_VB_cmc	0.696	0.543	0.802	0.417	0.859
Real stakeholder 1	c_ICB_shc	0.376	0.268	0.461	0.166	0.509
Real stakeholder 1	d_ICB_cmc	0.42	0.313	0.497	0.222	0.537
Real stakeholder 1	e_PCB	0.357	0.263	0.431	0.185	0.475
Real stakeholder 2	a_VB_shc	0.629	0.490	0.74	0.305	0.819
Real stakeholder 2	b_VB_cmc	0.661	0.524	0.784	0.347	0.843
Real stakeholder 2	c_ICB_shc	0.412	0.308	0.499	0.173	0.561
Real stakeholder 2	d_ICB_cmc	0.438	0.335	0.527	0.236	0.592
Real stakeholder 2	e_PCB	0.435	0.338	0.52	0.234	0.574
Real stakeholder 3	a_VB_shc	0.652	0.529	0.745	0.346	0.813
Real stakeholder 3	b_VB_cmc	0.694	0.575	0.789	0.441	0.847
Real stakeholder 3	c_ICB_shc	0.375	0.294	0.441	0.195	0.492
Real stakeholder 3	d_ICB_cmc	0.402	0.325	0.466	0.247	0.509
Real stakeholder 3	e_PCB	0.356	0.288	0.414	0.216	0.452

Note: Overall values for each option and each stakeholder using linear value functions and a non-additive aggregation model with γ=0.2 for scenario 1. Values normalized to the interval [0, 1], with higher values indicating better overall performance aligned with individual preferences. Uncertainty in the predictions was accounted for by 2,000 Monte Carlo simulations. Options: [a_VB_shc] Vaginal birth with standard hospital care; [b_VB_cmc] Vaginal birth with continuous midwifery care; [c_ICB_shc] Intrapartum cesarean birth with standard hospital care; [d_ICB_cmc] Intrapartum cesarean birth with continuous midwifery care; [e_PCB] Prelabor cesarean birth.

S3-5b. Overall values of childbirth options with linear value functions and γ=0.2, scenario 2

Stakeholder	Option	Mean overall value	5% quantile of overall value	95% quantile of overall value	Minimum	Maximum
Biomedical safety enthusiast	a_VB_shc	0.575	0.494	0.655	0.416	0.743
Biomedical safety enthusiast	b_VB_cmc	0.573	0.480	0.663	0.395	0.814
Biomedical safety enthusiast	c_ICB_shc	0.648	0.580	0.705	0.503	0.753
Biomedical safety enthusiast	d_ICB_cmc	0.709	0.629	0.782	0.53	0.851
Biomedical safety enthusiast	e_PCB	0.727	0.670	0.779	0.61	0.824
Natural birth proponent	a_VB_shc	0.64	0.557	0.704	0.431	0.751
Natural birth proponent	b_VB_cmc	0.723	0.645	0.778	0.584	0.809
Natural birth proponent	c_ICB_shc	0.098	0.079	0.115	0.057	0.131
Natural birth proponent	d_ICB_cmc	0.109	0.090	0.124	0.073	0.134
Natural birth proponent	e_PCB	0.042	0.032	0.051	0.023	0.058
Psychosocial experience advocate	a_VB_shc	0.525	0.263	0.743	0.049	0.829
Psychosocial experience advocate	b_VB_cmc	0.607	0.344	0.799	0.195	0.856
Psychosocial experience advocate	c_ICB_shc	0.482	0.233	0.699	0.054	0.79
Psychosocial experience advocate	d_ICB_cmc	0.599	0.326	0.810	0.145	0.885
Psychosocial experience advocate	e_PCB	0.587	0.313	0.805	0.153	0.897
Resource utilization pragmatist	a_VB_shc	0.633	0.574	0.69	0.497	0.732
Resource utilization pragmatist	b_VB_cmc	0.634	0.563	0.699	0.482	0.787
Resource utilization pragmatist	c_ICB_shc	0.567	0.515	0.608	0.452	0.638
Resource utilization pragmatist	d_ICB_cmc	0.591	0.534	0.638	0.462	0.68
Resource utilization pragmatist	e_PCB	0.712	0.660	0.755	0.619	0.784
Real stakeholder 1	a_VB_shc	0.58	0.423	0.698	0.219	0.759
Real stakeholder 1	b_VB_cmc	0.641	0.494	0.746	0.362	0.791
Real stakeholder 1	c_ICB_shc	0.333	0.232	0.410	0.129	0.459
Real stakeholder 1	d_ICB_cmc	0.379	0.278	0.453	0.196	0.503
Real stakeholder 1	e_PCB	0.327	0.235	0.397	0.173	0.439
Real stakeholder 2	a_VB_shc	0.581	0.448	0.688	0.283	0.753
Real stakeholder 2	b_VB_cmc	0.61	0.475	0.726	0.292	0.8
Real stakeholder 2	c_ICB_shc	0.369	0.273	0.451	0.192	0.52
Real stakeholder 2	d_ICB_cmc	0.398	0.301	0.486	0.207	0.557
Real stakeholder 2	e_PCB	0.398	0.305	0.479	0.245	0.536
Real stakeholder 3	a_VB_shc	0.593	0.476	0.683	0.32	0.738
Real stakeholder 3	b_VB_cmc	0.632	0.518	0.725	0.369	0.786
Real stakeholder 3	c_ICB_shc	0.326	0.254	0.387	0.187	0.437
Real stakeholder 3	d_ICB_cmc	0.357	0.285	0.420	0.221	0.471
Real stakeholder 3	e_PCB	0.319	0.255	0.375	0.211	0.416

Note: Overall values for each option and each stakeholder using linear value functions and a non-additive aggregation model with γ=0.2 for scenario 2. Values normalized to the interval [0, 1], with higher values indicating better overall performance aligned with individual preferences. Uncertainty in the predictions was accounted for by 2,000 Monte Carlo simulations. Options: [a_VB_shc] Vaginal birth with standard hospital care; [b_VB_cmc] Vaginal birth with continuous midwifery care; [c_ICB_shc] Intrapartum cesarean birth with standard hospital care; [d_ICB_cmc] Intrapartum cesarean birth with continuous midwifery care; [e_PCB] Prelabor cesarean birth.

S3-5c. Overall values of childbirth options with linear value functions and γ=0.2, scenario 3

Stakeholder	Option	Mean overall value	5% quantile of overall value	95% quantile of overall value	Minimum	Maximum
Biomedical safety enthusiast	a_VB_shc	0.669	0.593	0.742	0.52	0.805
Biomedical safety enthusiast	b_VB_cmc	0.667	0.594	0.731	0.518	0.793
Biomedical safety enthusiast	c_ICB_shc	0.735	0.672	0.786	0.579	0.828
Biomedical safety enthusiast	d_ICB_cmc	0.777	0.709	0.831	0.623	0.872
Biomedical safety enthusiast	e_PCB	0.798	0.744	0.845	0.691	0.887
Natural birth proponent	a_VB_shc	0.726	0.634	0.792	0.503	0.829
Natural birth proponent	b_VB_cmc	0.797	0.716	0.847	0.637	0.876
Natural birth proponent	c_ICB_shc	0.126	0.104	0.143	0.076	0.155
Natural birth proponent	d_ICB_cmc	0.138	0.117	0.152	0.098	0.161
Natural birth proponent	e_PCB	0.05	0.04	0.058	0.028	0.064
Psychosocial experience advocate	a_VB_shc	0.564	0.277	0.786	0.064	0.855
Psychosocial experience advocate	b_VB_cmc	0.648	0.382	0.836	0.205	0.907
Psychosocial experience advocate	c_ICB_shc	0.511	0.256	0.739	0.059	0.835
Psychosocial experience advocate	d_ICB_cmc	0.621	0.344	0.834	0.171	0.915
Psychosocial experience advocate	e_PCB	0.614	0.342	0.841	0.144	0.936
Resource utilization pragmatist	a_VB_shc	0.744	0.684	0.798	0.621	0.844
Resource utilization pragmatist	b_VB_cmc	0.743	0.677	0.797	0.594	0.843
Resource utilization pragmatist	c_ICB_shc	0.676	0.621	0.717	0.535	0.746
Resource utilization pragmatist	d_ICB_cmc	0.689	0.631	0.734	0.559	0.76
Resource utilization pragmatist	e_PCB	0.811	0.759	0.853	0.708	0.882
Real stakeholder 1	a_VB_shc	0.643	0.469	0.766	0.275	0.818
Real stakeholder 1	b_VB_cmc	0.701	0.547	0.803	0.426	0.862
Real stakeholder 1	c_ICB_shc	0.379	0.27	0.463	0.141	0.511
Real stakeholder 1	d_ICB_cmc	0.424	0.318	0.501	0.225	0.546
Real stakeholder 1	e_PCB	0.361	0.265	0.433	0.171	0.47
Real stakeholder 2	a_VB_shc	0.638	0.497	0.753	0.345	0.815
Real stakeholder 2	b_VB_cmc	0.664	0.522	0.777	0.389	0.853
Real stakeholder 2	c_ICB_shc	0.417	0.309	0.504	0.188	0.564
Real stakeholder 2	d_ICB_cmc	0.444	0.339	0.534	0.235	0.595
Real stakeholder 2	e_PCB	0.439	0.343	0.52	0.235	0.579
Real stakeholder 3	a_VB_shc	0.662	0.538	0.759	0.398	0.81
Real stakeholder 3	b_VB_cmc	0.698	0.58	0.788	0.471	0.851
Real stakeholder 3	c_ICB_shc	0.379	0.295	0.445	0.195	0.49
Real stakeholder 3	d_ICB_cmc	0.407	0.328	0.472	0.255	0.516
Real stakeholder 3	e_PCB	0.359	0.291	0.414	0.212	0.452

Note: Overall values for each option and each stakeholder using linear value functions and a non-additive aggregation model with γ=0.2 for scenario 3. Values normalized to the interval [0, 1], with higher values indicating better overall performance aligned with individual preferences. Uncertainty in the predictions was accounted for by 2,000 Monte Carlo simulations. Options: [a_VB_shc] Vaginal birth with standard hospital care; [b_VB_cmc] Vaginal birth with continuous midwifery care; [c_ICB_shc] Intrapartum cesarean birth with standard hospital care; [d_ICB_cmc] Intrapartum cesarean birth with continuous midwifery care; [e_PCB] Prelabor cesarean birth.

S3-5d. Overall values of childbirth options with linear value functions and γ=0.2, scenario 4

Stakeholder	Option	Mean overall value	5% quantile of overall value	95% quantile of overall value	Minimum	Maximum
Biomedical safety enthusiast	a_VB_shc	0.643	0.571	0.713	0.509	0.774
Biomedical safety enthusiast	b_VB_cmc	0.596	0.538	0.645	0.446	0.684
Biomedical safety enthusiast	c_ICB_shc	0.700	0.638	0.755	0.546	0.794
Biomedical safety enthusiast	d_ICB_cmc	0.766	0.702	0.820	0.6	0.899
Biomedical safety enthusiast	e_PCB	0.775	0.719	0.823	0.65	0.868
Natural birth proponent	a_VB_shc	0.692	0.598	0.760	0.444	0.816
Natural birth proponent	b_VB_cmc	0.748	0.673	0.795	0.611	0.817
Natural birth proponent	c_ICB_shc	0.119	0.098	0.136	0.069	0.146
Natural birth proponent	d_ICB_cmc	0.131	0.111	0.145	0.096	0.152
Natural birth proponent	e_PCB	0.047	0.036	0.055	0.025	0.062
Psychosocial experience advocate	a_VB_shc	0.549	0.266	0.772	0.039	0.868
Psychosocial experience advocate	b_VB_cmc	0.618	0.357	0.804	0.205	0.869
Psychosocial experience advocate	c_ICB_shc	0.501	0.237	0.719	0.04	0.805
Psychosocial experience advocate	d_ICB_cmc	0.613	0.329	0.831	0.192	0.925
Psychosocial experience advocate	e_PCB	0.609	0.332	0.839	0.157	0.922
Resource utilization pragmatist	a_VB_shc	0.679	0.625	0.729	0.562	0.763
Resource utilization pragmatist	b_VB_cmc	0.649	0.590	0.692	0.494	0.717
Resource utilization pragmatist	c_ICB_shc	0.600	0.549	0.640	0.467	0.665
Resource utilization pragmatist	d_ICB_cmc	0.629	0.574	0.672	0.49	0.714
Resource utilization pragmatist	e_PCB	0.744	0.694	0.785	0.65	0.812
Real stakeholder 1	a_VB_shc	0.615	0.451	0.736	0.222	0.802
Real stakeholder 1	b_VB_cmc	0.656	0.511	0.756	0.392	0.812
Real stakeholder 1	c_ICB_shc	0.361	0.254	0.443	0.103	0.485
Real stakeholder 1	d_ICB_cmc	0.408	0.300	0.484	0.231	0.522
Real stakeholder 1	e_PCB	0.345	0.255	0.416	0.17	0.453
Real stakeholder 2	a_VB_shc	0.609	0.474	0.721	0.318	0.796
Real stakeholder 2	b_VB_cmc	0.622	0.486	0.733	0.317	0.812
Real stakeholder 2	c_ICB_shc	0.394	0.292	0.481	0.116	0.538
Real stakeholder 2	d_ICB_cmc	0.424	0.319	0.510	0.219	0.568
Real stakeholder 2	e_PCB	0.417	0.325	0.497	0.211	0.548
Real stakeholder 3	a_VB_shc	0.628	0.512	0.723	0.351	0.78
Real stakeholder 3	b_VB_cmc	0.647	0.533	0.736	0.399	0.797
Real stakeholder 3	c_ICB_shc	0.356	0.278	0.420	0.135	0.461
Real stakeholder 3	d_ICB_cmc	0.387	0.308	0.449	0.233	0.489
Real stakeholder 3	e_PCB	0.338	0.272	0.392	0.194	0.43

Note: Overall values for each option and each stakeholder using linear value functions and a non-additive aggregation model with γ=0.2 for scenario 4. Values normalized to the interval [0, 1], with higher values indicating better overall performance aligned with individual preferences. Uncertainty in the predictions was accounted for by 2,000 Monte Carlo simulations. Options: [a_VB_shc] Vaginal birth with standard hospital care; [b_VB_cmc] Vaginal birth with continuous midwifery care; [c_ICB_shc] Intrapartum cesarean birth with standard hospital care; [d_ICB_cmc] Intrapartum cesarean birth with continuous midwifery care; [e_PCB] Prelabor cesarean birth.

S3-6. Sensitivity analyses: Overall values of childbirth options with stakeholder value functions and γ=1

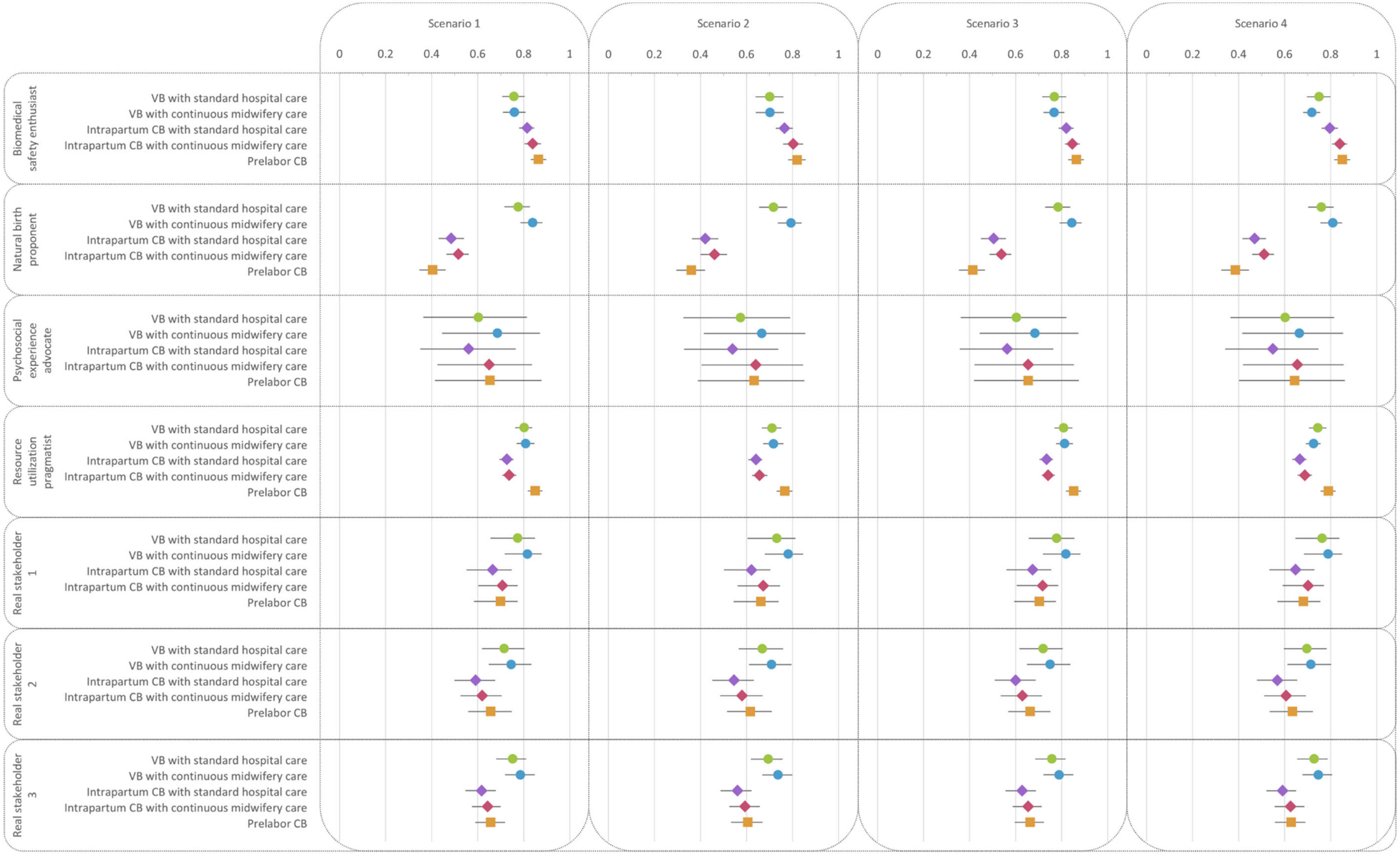

Note: Mean overall values for each option and each stakeholder in each scenario using stakeholder value functions and an additive aggregation model with γ=1. Values normalized to the interval [0, 1], with higher values indicating better overall performance aligned with individual preferences. Uncertainty in the predictions was accounted for by 2,000 Monte Carlo simulations. Error bars indicate the 5% and 95% quantiles of the overall values. Scenarios: (1) Women with no cesarean birth history or comorbidities; (2) Women with no cesarean birth history but comorbidities; (3) Women with a cesarean birth history but no comorbidities; (4) Women with a cesarean birth history and comorbidities. Childbirth options: ● Vaginal birth (VB) with standard hospital care; ● Vaginal birth (VB) with continuous midwifery care; ◆ Intrapartum cesarean birth (CB) with standard hospital care; ◆ Intrapartum cesarean birth (CB) with continuous midwifery care; ■ Prelabor cesarean birth (CB).

S3-6a. Overall values of childbirth options with stakeholder value functions and γ=1, scenario 1

Stakeholder	Option	Mean overall value	5% quantile of overall value	95% quantile of overall value	Minimum	Maximum
Biomedical safety enthusiast	a_VB_shc	0.756	0.706	0.804	0.67	0.847
Biomedical safety enthusiast	b_VB_cmc	0.759	0.710	0.807	0.658	0.876
Biomedical safety enthusiast	c_ICB_shc	0.814	0.780	0.846	0.755	0.876
Biomedical safety enthusiast	d_ICB_cmc	0.838	0.803	0.875	0.77	0.913
Biomedical safety enthusiast	e_PCB	0.863	0.830	0.898	0.806	0.927
Natural birth proponent	a_VB_shc	0.775	0.716	0.827	0.662	0.896
Natural birth proponent	b_VB_cmc	0.838	0.786	0.881	0.737	0.904
Natural birth proponent	c_ICB_shc	0.484	0.431	0.540	0.383	0.591
Natural birth proponent	d_ICB_cmc	0.516	0.465	0.561	0.421	0.591
Natural birth proponent	e_PCB	0.404	0.346	0.461	0.289	0.5
Psychosocial experience advocate	a_VB_shc	0.602	0.364	0.814	0.248	0.915
Psychosocial experience advocate	b_VB_cmc	0.685	0.445	0.870	0.306	0.949
Psychosocial experience advocate	c_ICB_shc	0.56	0.351	0.765	0.266	0.854
Psychosocial experience advocate	d_ICB_cmc	0.649	0.425	0.836	0.32	0.922
Psychosocial experience advocate	e_PCB	0.652	0.415	0.877	0.314	0.963
Resource utilization pragmatist	a_VB_shc	0.801	0.763	0.837	0.733	0.871
Resource utilization pragmatist	b_VB_cmc	0.808	0.769	0.847	0.735	0.884
Resource utilization pragmatist	c_ICB_shc	0.726	0.695	0.755	0.673	0.78
Resource utilization pragmatist	d_ICB_cmc	0.736	0.706	0.767	0.681	0.798
Resource utilization pragmatist	e_PCB	0.849	0.817	0.881	0.792	0.902
Real stakeholder 1	a_VB_shc	0.773	0.655	0.849	0.548	0.899
Real stakeholder 1	b_VB_cmc	0.815	0.717	0.878	0.616	0.909
Real stakeholder 1	c_ICB_shc	0.664	0.551	0.748	0.455	0.79
Real stakeholder 1	d_ICB_cmc	0.707	0.602	0.774	0.528	0.8
Real stakeholder 1	e_PCB	0.698	0.584	0.774	0.509	0.801
Real stakeholder 2	a_VB_shc	0.714	0.619	0.803	0.527	0.863
Real stakeholder 2	b_VB_cmc	0.745	0.648	0.833	0.567	0.895
Real stakeholder 2	c_ICB_shc	0.59	0.499	0.676	0.404	0.744
Real stakeholder 2	d_ICB_cmc	0.619	0.525	0.704	0.448	0.758
Real stakeholder 2	e_PCB	0.655	0.559	0.748	0.469	0.795
Real stakeholder 3	a_VB_shc	0.751	0.681	0.811	0.595	0.867
Real stakeholder 3	b_VB_cmc	0.785	0.719	0.848	0.647	0.894
Real stakeholder 3	c_ICB_shc	0.617	0.547	0.678	0.459	0.731
Real stakeholder 3	d_ICB_cmc	0.642	0.575	0.700	0.514	0.737
Real stakeholder 3	e_PCB	0.655	0.589	0.719	0.515	0.758

Note: Overall values for each option and each stakeholder using stakeholder value functions and an additive aggregation model with γ=1 for scenario 1. Values normalized to the interval [0, 1], with higher values indicating better overall performance aligned with individual preferences. Uncertainty in the predictions was accounted for by 2,000 Monte Carlo simulations. Options: [a_VB_shc] Vaginal birth with standard hospital care; [b_VB_cmc] Vaginal birth with continuous midwifery care; [c_ICB_shc] Intrapartum cesarean birth with standard hospital care; [d_ICB_cmc] Intrapartum cesarean birth with continuous midwifery care; [e_PCB] Prelabor cesarean birth.

S3-6b. Overall values of childbirth options with stakeholder value functions and γ=1, scenario 2

Stakeholder	Option	Mean overall value	5% quantile of overall value	95% quantile of overall value	Minimum	Maximum
Biomedical safety enthusiast	a_VB_shc	0.700	0.639	0.760	0.587	0.825
Biomedical safety enthusiast	b_VB_cmc	0.701	0.640	0.762	0.579	0.81
Biomedical safety enthusiast	c_ICB_shc	0.764	0.727	0.801	0.69	0.847
Biomedical safety enthusiast	d_ICB_cmc	0.802	0.759	0.846	0.714	0.887
Biomedical safety enthusiast	e_PCB	0.819	0.781	0.857	0.751	0.888
Natural birth proponent	a_VB_shc	0.716	0.655	0.776	0.602	0.831
Natural birth proponent	b_VB_cmc	0.793	0.736	0.839	0.697	0.872
Natural birth proponent	c_ICB_shc	0.420	0.362	0.478	0.29	0.54
Natural birth proponent	d_ICB_cmc	0.460	0.400	0.516	0.322	0.56
Natural birth proponent	e_PCB	0.359	0.295	0.420	0.235	0.483
Psychosocial experience advocate	a_VB_shc	0.573	0.326	0.790	0.222	0.88
Psychosocial experience advocate	b_VB_cmc	0.665	0.415	0.856	0.292	0.921
Psychosocial experience advocate	c_ICB_shc	0.538	0.329	0.738	0.238	0.826
Psychosocial experience advocate	d_ICB_cmc	0.639	0.405	0.846	0.306	0.92
Psychosocial experience advocate	e_PCB	0.632	0.389	0.851	0.288	0.937
Resource utilization pragmatist	a_VB_shc	0.710	0.666	0.751	0.638	0.793
Resource utilization pragmatist	b_VB_cmc	0.716	0.672	0.761	0.628	0.794
Resource utilization pragmatist	c_ICB_shc	0.640	0.608	0.669	0.577	0.693
Resource utilization pragmatist	d_ICB_cmc	0.656	0.624	0.690	0.594	0.717
Resource utilization pragmatist	e_PCB	0.765	0.731	0.799	0.705	0.822
Real stakeholder 1	a_VB_shc	0.731	0.605	0.813	0.508	0.852
Real stakeholder 1	b_VB_cmc	0.781	0.680	0.846	0.597	0.883
Real stakeholder 1	c_ICB_shc	0.621	0.501	0.703	0.406	0.75
Real stakeholder 1	d_ICB_cmc	0.672	0.561	0.746	0.471	0.792
Real stakeholder 1	e_PCB	0.661	0.544	0.739	0.441	0.779
Real stakeholder 2	a_VB_shc	0.668	0.565	0.759	0.475	0.833
Real stakeholder 2	b_VB_cmc	0.708	0.611	0.796	0.504	0.856
Real stakeholder 2	c_ICB_shc	0.545	0.451	0.632	0.379	0.71
Real stakeholder 2	d_ICB_cmc	0.580	0.485	0.670	0.395	0.736
Real stakeholder 2	e_PCB	0.616	0.516	0.710	0.414	0.783
Real stakeholder 3	a_VB_shc	0.694	0.619	0.757	0.541	0.811
Real stakeholder 3	b_VB_cmc	0.736	0.669	0.799	0.581	0.851
Real stakeholder 3	c_ICB_shc	0.56	0.487	0.622	0.42	0.682
Real stakeholder 3	d_ICB_cmc	0.593	0.525	0.658	0.449	0.717
Real stakeholder 3	e_PCB	0.605	0.533	0.670	0.443	0.725

Note: Overall values for each option and each stakeholder using stakeholder value functions and an additive aggregation model with γ=1 for scenario 2. Values normalized to the interval [0, 1], with higher values indicating better overall performance aligned with individual preferences. Uncertainty in the predictions was accounted for by 2,000 Monte Carlo simulations. Options: [a_VB_shc] Vaginal birth with standard hospital care; [b_VB_cmc] Vaginal birth with continuous midwifery care; [c_ICB_shc] Intrapartum cesarean birth with standard hospital care; [d_ICB_cmc] Intrapartum cesarean birth with continuous midwifery care; [e_PCB] Prelabor cesarean birth.

S3-6c. Overall values of childbirth options with stakeholder value functions and γ=1, scenario 3

Stakeholder	Option	Mean overall value	5% quantile of overall value	95% quantile of overall value	Minimum	Maximum
Biomedical safety enthusiast	a_VB_shc	0.768	0.716	0.820	0.667	0.867
Biomedical safety enthusiast	b_VB_cmc	0.767	0.721	0.812	0.679	0.864
Biomedical safety enthusiast	c_ICB_shc	0.82	0.787	0.853	0.762	0.878
Biomedical safety enthusiast	d_ICB_cmc	0.846	0.815	0.879	0.786	0.913
Biomedical safety enthusiast	e_PCB	0.863	0.829	0.897	0.796	0.922
Natural birth proponent	a_VB_shc	0.784	0.729	0.837	0.669	0.877
Natural birth proponent	b_VB_cmc	0.844	0.792	0.887	0.76	0.912
Natural birth proponent	c_ICB_shc	0.504	0.449	0.558	0.393	0.598
Natural birth proponent	d_ICB_cmc	0.538	0.486	0.581	0.457	0.602
Natural birth proponent	e_PCB	0.413	0.353	0.467	0.307	0.522
Psychosocial experience advocate	a_VB_shc	0.603	0.361	0.821	0.262	0.907
Psychosocial experience advocate	b_VB_cmc	0.683	0.444	0.873	0.315	0.938
Psychosocial experience advocate	c_ICB_shc	0.562	0.357	0.765	0.26	0.835
Psychosocial experience advocate	d_ICB_cmc	0.654	0.421	0.854	0.307	0.924
Psychosocial experience advocate	e_PCB	0.654	0.419	0.875	0.315	0.961
Resource utilization pragmatist	a_VB_shc	0.808	0.769	0.847	0.729	0.884
Resource utilization pragmatist	b_VB_cmc	0.812	0.777	0.849	0.741	0.887
Resource utilization pragmatist	c_ICB_shc	0.734	0.704	0.762	0.686	0.787
Resource utilization pragmatist	d_ICB_cmc	0.741	0.714	0.771	0.694	0.794
Resource utilization pragmatist	e_PCB	0.852	0.819	0.884	0.795	0.904
Real stakeholder 1	a_VB_shc	0.778	0.657	0.856	0.564	0.886
Real stakeholder 1	b_VB_cmc	0.818	0.719	0.882	0.62	0.91
Real stakeholder 1	c_ICB_shc	0.673	0.560	0.755	0.454	0.785
Real stakeholder 1	d_ICB_cmc	0.717	0.605	0.785	0.514	0.811
Real stakeholder 1	e_PCB	0.703	0.594	0.776	0.504	0.816
Real stakeholder 2	a_VB_shc	0.719	0.617	0.805	0.553	0.861
Real stakeholder 2	b_VB_cmc	0.749	0.649	0.839	0.573	0.891
Real stakeholder 2	c_ICB_shc	0.600	0.509	0.687	0.423	0.741
Real stakeholder 2	d_ICB_cmc	0.629	0.535	0.715	0.438	0.766
Real stakeholder 2	e_PCB	0.662	0.568	0.752	0.478	0.818
Real stakeholder 3	a_VB_shc	0.757	0.685	0.818	0.623	0.856
Real stakeholder 3	b_VB_cmc	0.789	0.721	0.852	0.655	0.891
Real stakeholder 3	c_ICB_shc	0.628	0.556	0.689	0.473	0.725
Real stakeholder 3	d_ICB_cmc	0.654	0.588	0.714	0.504	0.75
Real stakeholder 3	e_PCB	0.662	0.596	0.723	0.524	0.775

Note: Overall values for each option and each stakeholder using stakeholder value functions and an additive aggregation model with γ=1 for scenario 3. Values normalized to the interval [0, 1], with higher values indicating better overall performance aligned with individual preferences. Uncertainty in the predictions was accounted for by 2,000 Monte Carlo simulations. Options: [a_VB_shc] Vaginal birth with standard hospital care; [b_VB_cmc] Vaginal birth with continuous midwifery care; [c_ICB_shc] Intrapartum cesarean birth with standard hospital care; [d_ICB_cmc] Intrapartum cesarean birth with continuous midwifery care; [e_PCB] Prelabor cesarean birth.

S3-6d. Overall values of childbirth options with stakeholder value functions and γ=1, scenario 4

Stakeholder	Option	Mean overall value	5% quantile of overall value	95% quantile of overall value	Minimum	Maximum
Biomedical safety enthusiast	a_VB_shc	0.749	0.697	0.799	0.6	0.872
Biomedical safety enthusiast	b_VB_cmc	0.717	0.681	0.754	0.652	0.784
Biomedical safety enthusiast	c_ICB_shc	0.796	0.761	0.831	0.725	0.86
Biomedical safety enthusiast	d_ICB_cmc	0.839	0.806	0.872	0.784	0.913
Biomedical safety enthusiast	e_PCB	0.850	0.815	0.885	0.78	0.916
Natural birth proponent	a_VB_shc	0.759	0.702	0.813	0.653	0.864
Natural birth proponent	b_VB_cmc	0.809	0.755	0.850	0.726	0.867
Natural birth proponent	c_ICB_shc	0.469	0.417	0.519	0.374	0.552
Natural birth proponent	d_ICB_cmc	0.510	0.459	0.554	0.432	0.575
Natural birth proponent	e_PCB	0.385	0.324	0.445	0.274	0.485
Psychosocial experience advocate	a_VB_shc	0.601	0.365	0.815	0.243	0.898
Psychosocial experience advocate	b_VB_cmc	0.663	0.416	0.854	0.312	0.922
Psychosocial experience advocate	c_ICB_shc	0.548	0.342	0.747	0.269	0.83
Psychosocial experience advocate	d_ICB_cmc	0.654	0.419	0.856	0.307	0.929
Psychosocial experience advocate	e_PCB	0.643	0.402	0.862	0.307	0.95
Resource utilization pragmatist	a_VB_shc	0.744	0.706	0.781	0.639	0.836
Resource utilization pragmatist	b_VB_cmc	0.725	0.693	0.757	0.677	0.78
Resource utilization pragmatist	c_ICB_shc	0.665	0.634	0.695	0.607	0.716
Resource utilization pragmatist	d_ICB_cmc	0.687	0.657	0.718	0.637	0.742
Resource utilization pragmatist	e_PCB	0.789	0.757	0.822	0.727	0.848
Real stakeholder 1	a_VB_shc	0.762	0.647	0.838	0.518	0.878
Real stakeholder 1	b_VB_cmc	0.788	0.684	0.850	0.605	0.886
Real stakeholder 1	c_ICB_shc	0.647	0.534	0.729	0.442	0.759
Real stakeholder 1	d_ICB_cmc	0.701	0.591	0.771	0.485	0.802
Real stakeholder 1	e_PCB	0.681	0.569	0.755	0.486	0.8
Real stakeholder 2	a_VB_shc	0.696	0.597	0.783	0.494	0.847
Real stakeholder 2	b_VB_cmc	0.713	0.612	0.802	0.529	0.852
Real stakeholder 2	c_ICB_shc	0.568	0.480	0.654	0.393	0.706
Real stakeholder 2	d_ICB_cmc	0.606	0.511	0.692	0.422	0.753
Real stakeholder 2	e_PCB	0.633	0.535	0.723	0.435	0.786
Real stakeholder 3	a_VB_shc	0.727	0.654	0.787	0.555	0.836
Real stakeholder 3	b_VB_cmc	0.746	0.677	0.807	0.611	0.853
Real stakeholder 3	c_ICB_shc	0.590	0.521	0.650	0.445	0.686
Real stakeholder 3	d_ICB_cmc	0.625	0.557	0.686	0.477	0.73
Real stakeholder 3	e_PCB	0.627	0.558	0.690	0.472	0.747

Note: Overall values for each option and each stakeholder using stakeholder value functions and an additive aggregation model with γ=1 for scenario 4. Values normalized to the interval [0, 1], with higher values indicating better overall performance aligned with individual preferences. Uncertainty in the predictions was accounted for by 2,000 Monte Carlo simulations. Options: [a_VB_shc] Vaginal birth with standard hospital care; [b_VB_cmc] Vaginal birth with continuous midwifery care; [c_ICB_shc] Intrapartum cesarean birth with standard hospital care; [d_ICB_cmc] Intrapartum cesarean birth with continuous midwifery care; [e_PCB] Prelabor cesarean birth.

References

1. Swiss Society of Gynecology and Obstetrics (Schweizerische Gesellschaft für Gynäkologie und Geburtshilfe [SGGG]). Guideline sectio caesarea. Accessed May 22, 2025, https://www.sggg.ch/fileadmin/user_upload/Dokumente/3_Fachinformationen/2_Guidelines/De/Guideline_Sectio_Caesarea_2015.pdf
2. Association of the Scientific Medical Societies (Arbeitsgemeinschaft der Wissenschaftlichen Medizinischen Fachgesellschaften [AWMF]). Sectio caesarea. Accessed May 22, 2025, https://www.awmf.org/uploads/tx_szleitlinien/015-084l_S3_Sectio-caesarea_2020-06_1_02.pdf
3. Mylonas I, Friese K. Indications for and risks of elective cesarean section. Dtsch Arztebl Int. 2015;112(29-30):489-95. doi:10.3238/arztebl.2015.0489
4. Sandall J, Soltani H, Gates S, Shennan A, Devane D. Midwife-led continuity models versus other models of care for childbearing women. *Cochrane Database Syst Rev*. 2016;(4). doi:10.1002/14651858.CD004667.pub5
5. Sandall J. The contribution of continuity of midwifery care to high quality maternity care. Accessed June 30, 2023, https://pre.rcm.org.uk/media/2265/continuity-of-care.pdf
6. Homer CS. Models of maternity care: evidence for midwifery continuity of care. *Med J Aust*. 2016;205(8):370-374. doi:10.5694/mja16.00844
7. Rooks JP. The midwifery model of care. *J Nurse Midwifery*. 1999;44(4):370-374. doi:10.1016/S0091-2182(99)00060-9
8. Sevisa (Arbeitsgemeinschaft Schweizerischer Frauenkliniken [ASF]). A national Swiss hospital in-patient database for obstetric and gynecological hospital admissions. Accessed October 26, 2023, https://sevisa.ch/
9. Haag F, Aubert AH, Lienert J. ValueDecisions, a web app to support decisions with conflicting objectives, multiple stakeholders, and uncertainty. *Environ Model Softw*. 2022;150:105361. doi:10.1016/j.envsoft.2022.105361
10. Ball JA, Washbrook M, The Royal College of Midwives. Working with Birthrate Plus®. Accessed March 14, 2025, https://www.rcm.org.uk/media/2375/working-with-birthrate-plus.pdf
11. Akter S, Bennett J. Preference uncertainty in stated preference studies: facts and artefacts. *Appl Econ*. 2013;45(15):2107-2115. doi:10.1080/00036846.2012.654914
12. Zheng J, Egger C, Lienert J. A scenario-based MCDA framework for wastewater infrastructure planning under uncertainty. SUPPLEMENTARY MATERIAL. *J Environ Manage*. 2016;183:895-908. doi:10.1016/j.jenvman.2016.09.027
